# Supplementary material for: Large-scale multitrait genome-wide association analyses identify hundreds of glaucoma risk loci
Source: Nat Genet. 2023 Jun 29;55(7):1116–25. doi: 10.1038/s41588-023-01428-5 (PMC10335935; doi:10.1038/s41588-023-01428-5)
Supplement: Supplementary file 1 — Reporting Summary [file 41588_2023_1428_MOESM1_ESM.pdf]

nature portfolio

Corresponding author(s): Xikun Han

Last updated by author(s): Jan 20, 2023

Reporting Summary

Nature Portfolio wishes to improve the reproducibility of the work that we publish. This form provides structure for consistency and transparency in reporting. For further information on Nature Portfolio policies, see our Editorial Policies and the Editorial Policy Checklist.

Statistics

For all statistical analyses, confirm that the following items are present in the figure legend, table legend, main text, or Methods section.

n/a	Confirmed	
☐	☒	The exact sample size (*n*) for each experimental group/condition, given as a discrete number and unit of measurement
☐	☒	A statement on whether measurements were taken from distinct samples or whether the same sample was measured repeatedly
☐	☒	The statistical test(s) used AND whether they are one- or two-sided<br>*Only common tests should be described solely by name; describe more complex techniques in the Methods section.*
☐	☒	A description of all covariates tested
☐	☒	A description of any assumptions or corrections, such as tests of normality and adjustment for multiple comparisons
☐	☒	A full description of the statistical parameters including central tendency (e.g. means) or other basic estimates (e.g. regression coefficient) AND variation (e.g. standard deviation) or associated estimates of uncertainty (e.g. confidence intervals)
☐	☒	For null hypothesis testing, the test statistic (e.g. *F*, *t*, *r*) with confidence intervals, effect sizes, degrees of freedom and *P* value noted<br>*Give P values as exact values whenever suitable.*
☒	☐	For Bayesian analysis, information on the choice of priors and Markov chain Monte Carlo settings
☒	☐	For hierarchical and complex designs, identification of the appropriate level for tests and full reporting of outcomes
☐	☒	Estimates of effect sizes (e.g. Cohen's *d*, Pearson's *r*), indicating how they were calculated

Our web collection on statistics for biologists contains articles on many of the points above.

Software and code

Policy information about availability of computer code

Data collection	In this study, we included genetic and phenotypic data from UK Biobank (UKB), Canadian Longitudinal Study on Aging (CLSA), Mass General Brigham Biobank, and our previously published GWAS for POAG, VCDR, and IOP. The detailed information for each study is described in the methods section.
Data analysis	In this study, we performed various genetic analysis, including genome-wide association study and cross-ancestry meta-analysis, multi-trait GWAS (MTAG) analysis, colocalization analysis, Mendelian randomization analysis, proteome-wide association study, etc. The detailed information is described in the methods section.<br>The following software packages were used for data analyses: BOLT-LMM software (version 2.3): https://data.broadinstitute.org/alkesgroup/BOLT-LMM; eCAVIAR: https://github.com/fhormoz/caviar; LOCUSZOOM: http://locuszoom.sph.umich.edu/ ; LD score regression software: https://github.com/bulik/ldsc; METAL software (2011-03-25 release): http://csg.sph.umich.edu/abecasis/Metal/; MTAG (v1.0.8): Multi-Trait Analysis of GWAS https://github.com/omeed-maghzian/mtag; PLINK software (v2.00): http://www.cog-genomics.org/plink2; R: https://cran.r-project.org/; REGENIE software (v1.0.6.2): https://rgcgithub.github.io/regenie/overview/.

For manuscripts utilizing custom algorithms or software that are central to the research but not yet described in published literature, software must be made available to editors and reviewers. We strongly encourage code deposition in a community repository (e.g. GitHub). See the Nature Portfolio guidelines for submitting code & software for further information.

Data

Policy information about availability of data

All manuscripts must include a data availability statement. This statement should provide the following information, where applicable:

- Accession codes, unique identifiers, or web links for publicly available datasets
- A description of any restrictions on data availability
- For clinical datasets or third party data, please ensure that the statement adheres to our policy

UK Biobank data are available through the UK Biobank Access Management System https://www.ukbiobank.ac.uk/.
Data are available from the Canadian Longitudinal Study on Aging (www.clsa-elcv.ca) for researchers who meet the criteria for access to de-identified CLSA data (https://www.clsa-elcv.ca/researchers/data-support-documentation).
The GWAS summary statistics from this study are available for research use at https://xikunhan.github.io/site/publication/.
Eye Genotype Expression data are are available at Gene Expression Omnibus (GEO) under accession code GSE115828.
The variant-level data for the 23andMe replication dataset are fully disclosed in the manuscript. Individual-level data are not publicly available due to participant confidentiality, and in accordance with the IRB-approved protocol under which the study was conducted.

Field-specific reporting

Please select the one below that is the best fit for your research. If you are not sure, read the appropriate sections before making your selection.

☒ Life sciences ☐ Behavioural & social sciences ☐ Ecological, evolutionary & environmental sciences

For a reference copy of the document with all sections, see nature.com/documents/nr-reporting-summary-flat.pdf

Life sciences study design

All studies must disclose on these points even when the disclosure is negative.

Sample size	We assembled the largest possible sample size to maximize the number of novel loci.
Data exclusions	This is described in the methods. Some samples were excluded based on genetic control procedures and genetic ancestry to ensure homogeneity.
Replication	The identified genetic loci were replicated in a large independent cohort from 23andMe, Inc (total sample size>2.8 million). For drug target discovery, we performed genetic analysis from multi-datasets to provide multi-level evidence. All data sets were analyzed independently to replicate genetic loci.
Randomization	Samples were from collected study cohorts and were not randomized (randomization is not applicable here). In our genetic association analysis, we adjust for age, sex, and genetic principal components to control covariates.
Blinding	Genotyping and quality control for the genetic data was conducted without knowledge of the phenotypes.

Reporting for specific materials, systems and methods

We require information from authors about some types of materials, experimental systems and methods used in many studies. Here, indicate whether each material, system or method listed is relevant to your study. If you are not sure if a list item applies to your research, read the appropriate section before selecting a response.

Materials & experimental systems

n/a	Involved in the study	
☒	☐	Antibodies
☒	☐	Eukaryotic cell lines
☒	☐	Palaeontology and archaeology
☒	☐	Animals and other organisms
☐	☒	Human research participants
☒	☐	Clinical data
☒	☐	Dual use research of concern

Methods

n/a	Involved in the study	
☒	☐	ChIP-seq
☒	☐	Flow cytometry
☒	☐	MRI-based neuroimaging

Human research participants

Policy information about studies involving human research participants

Population characteristics	. In this study, we included genetic and phenotypic data from UK Biobank (UKB), Canadian Longitudinal Study on Aging

Population characteristics	(CLSA), Mass General Brigham Biobank, and our previously published GWAS for POAG, VCDR, and IOP. Each study was described in full in the methods section.
Recruitment	Described in full in the methods section.
Ethics oversight	Described in full in the methods section.

Note that full information on the approval of the study protocol must also be provided in the manuscript.
