# Supplementary figures and images for: Large-scale multitrait genome-wide association analyses identify hundreds of glaucoma risk loci
Source: Nat Genet. 2023 Jun 29;55(7):1116–25. doi: 10.1038/s41588-023-01428-5 (PMC10335935; doi:10.1038/s41588-023-01428-5)

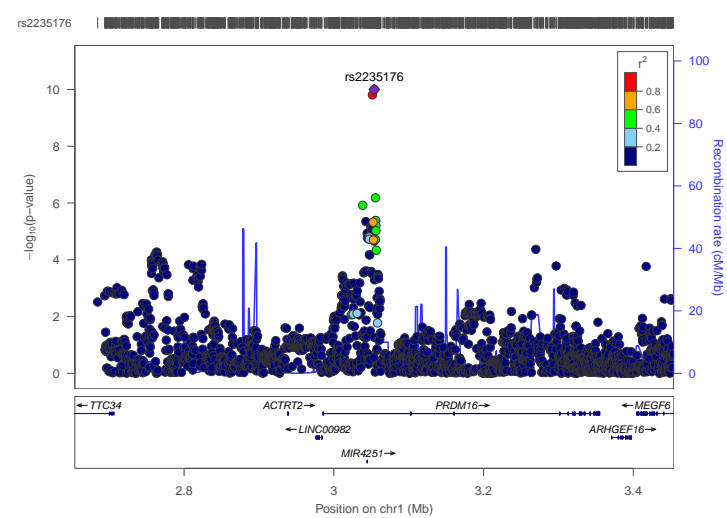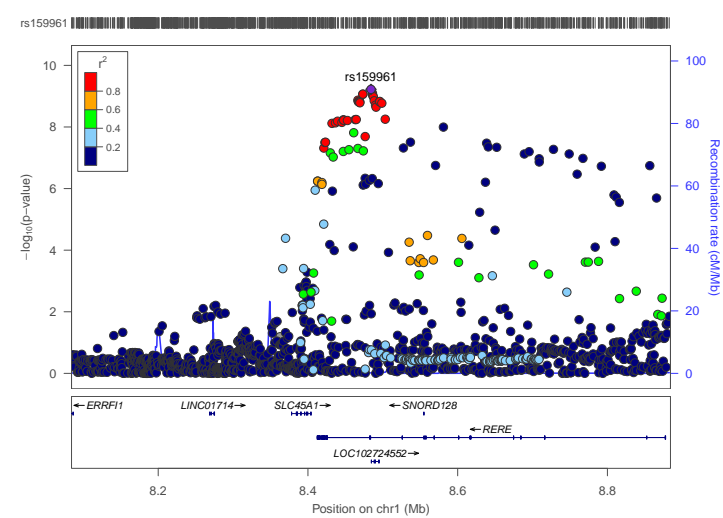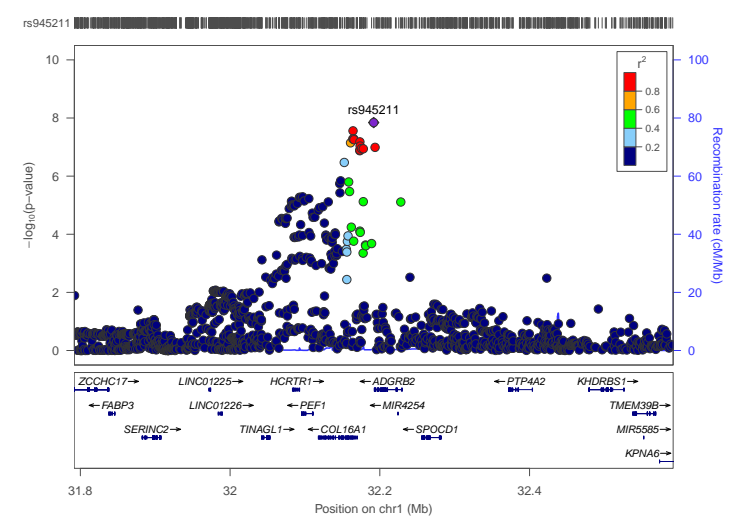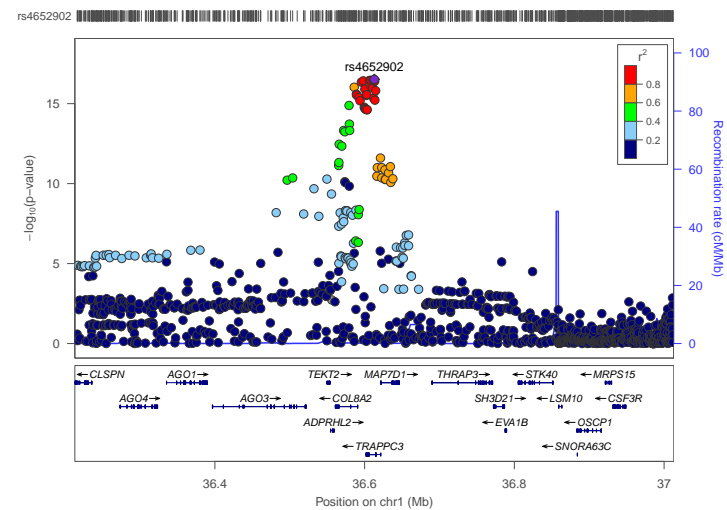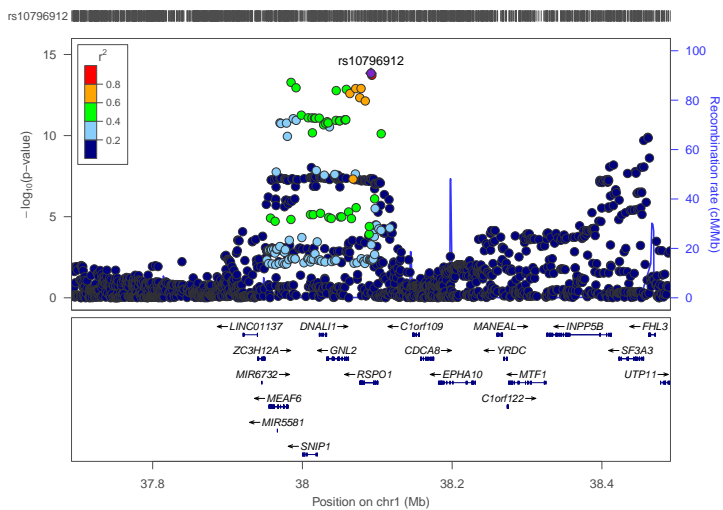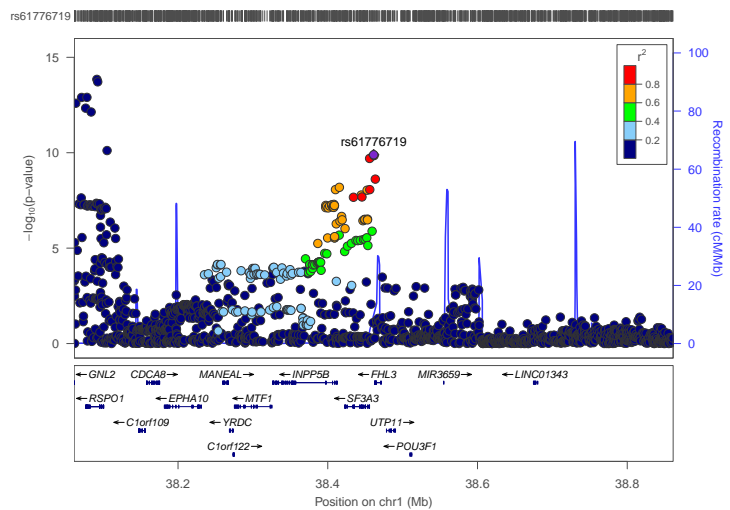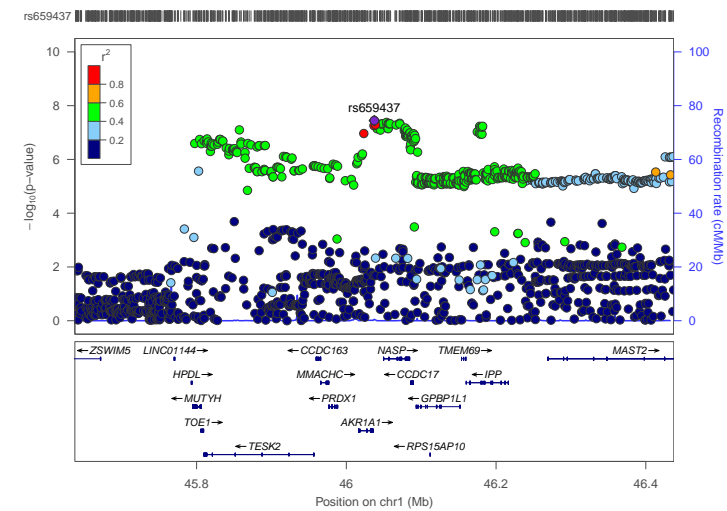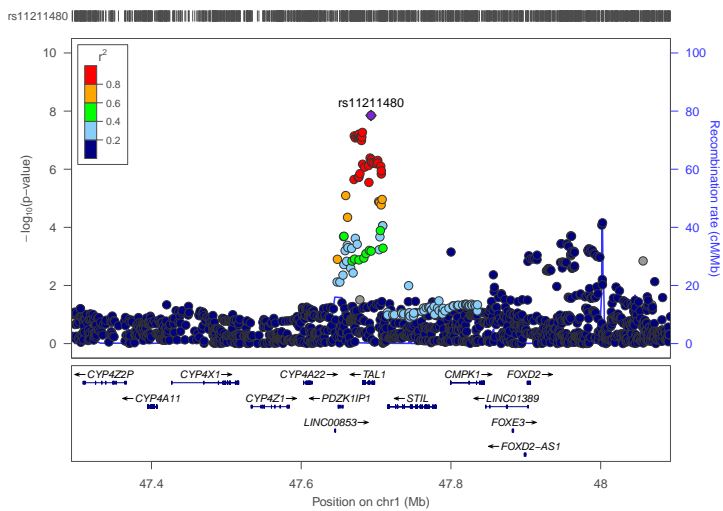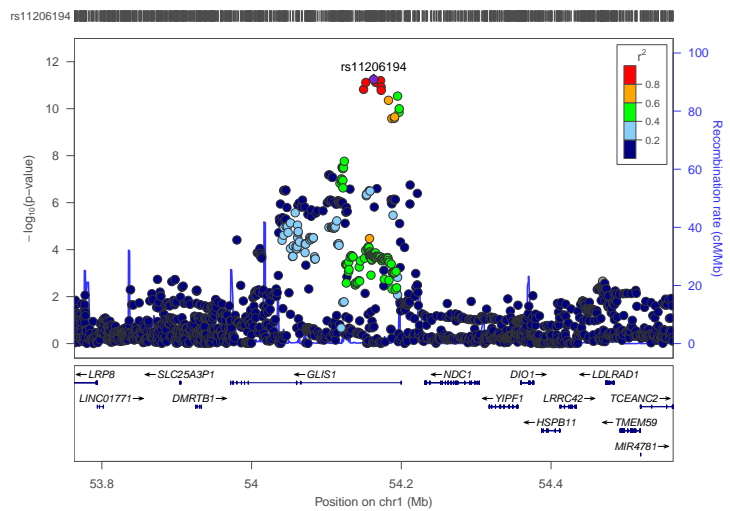

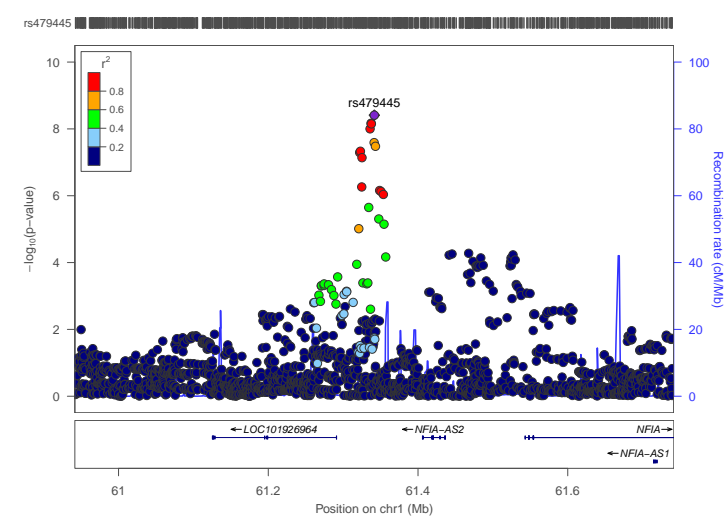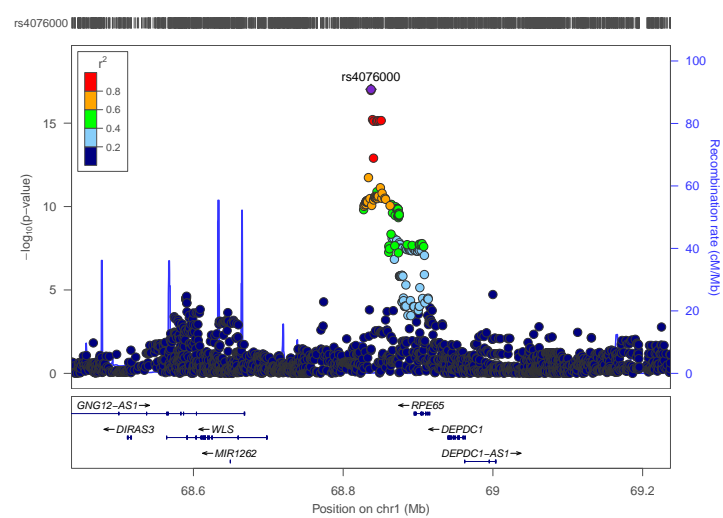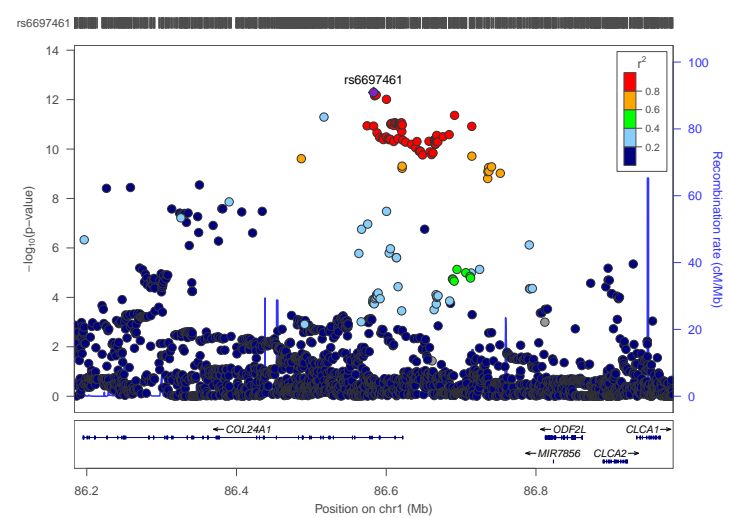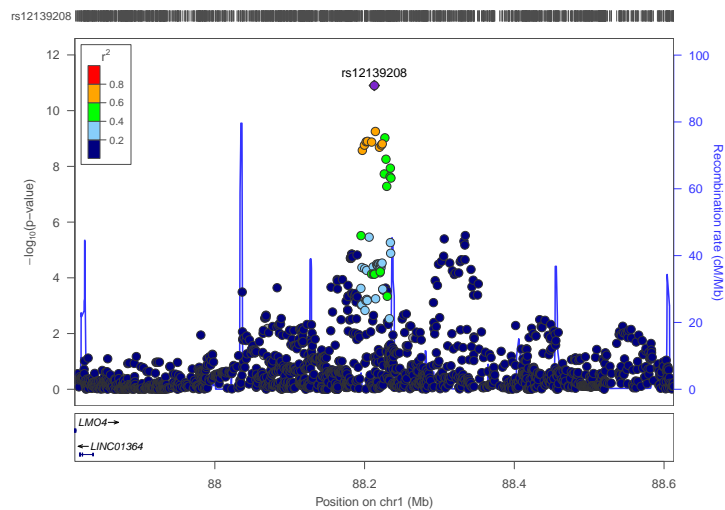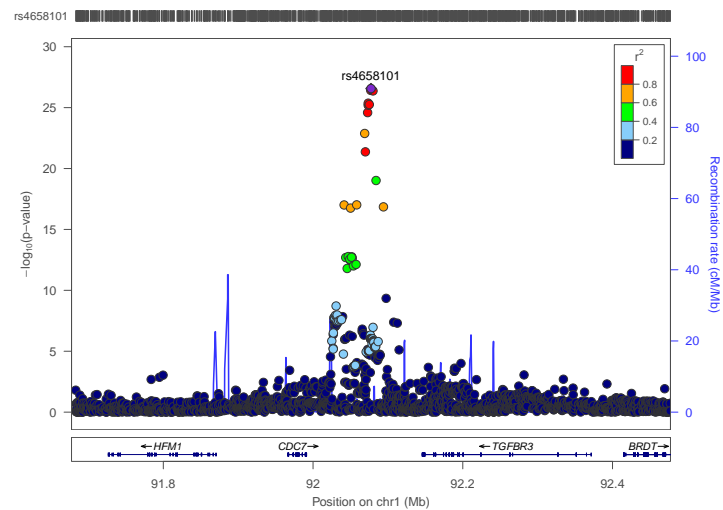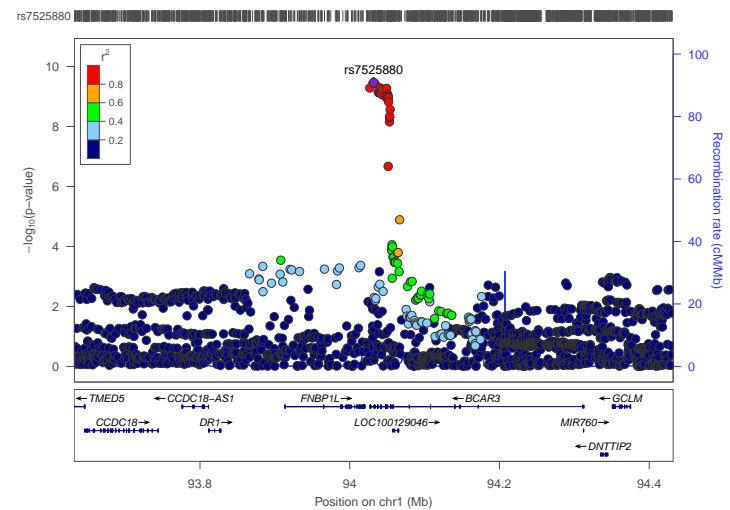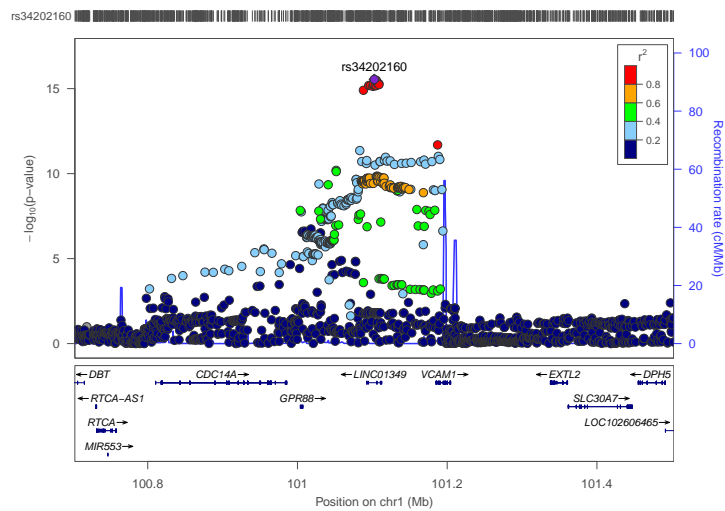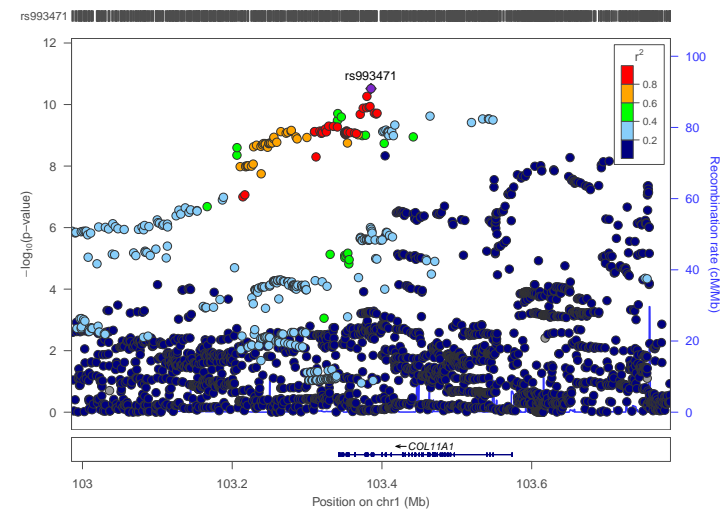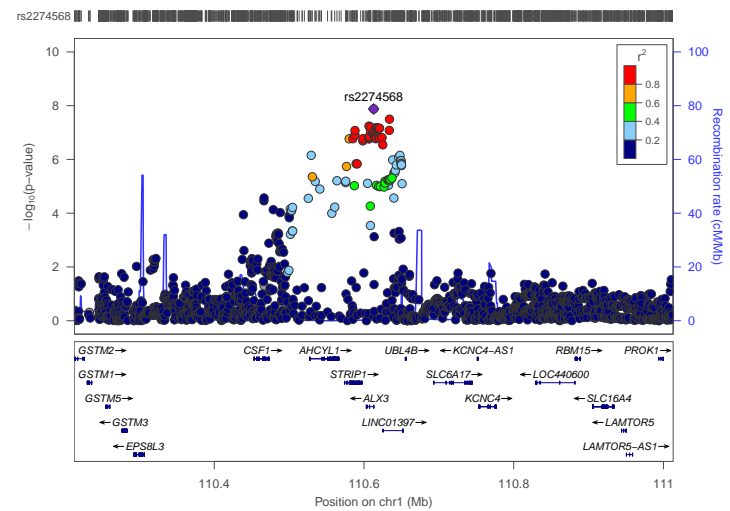

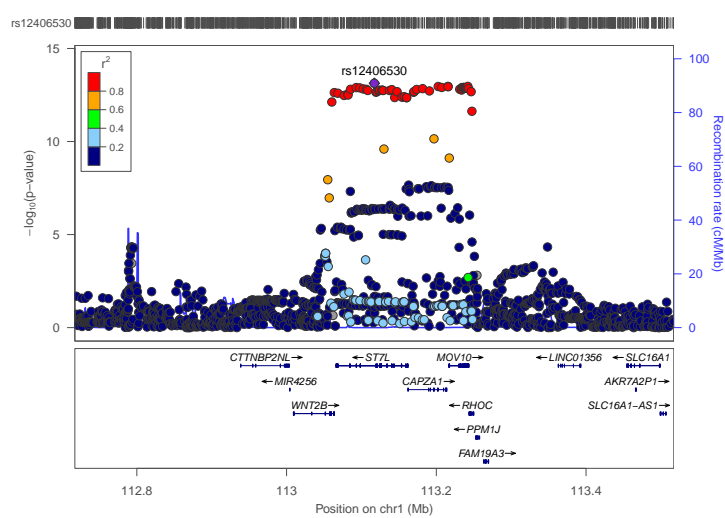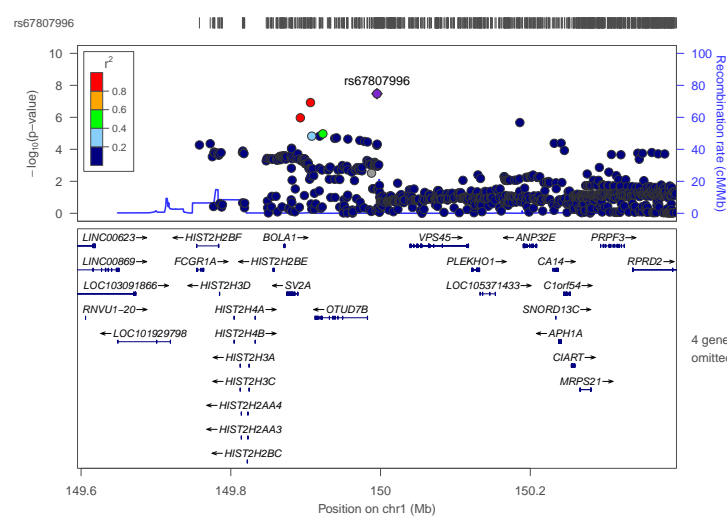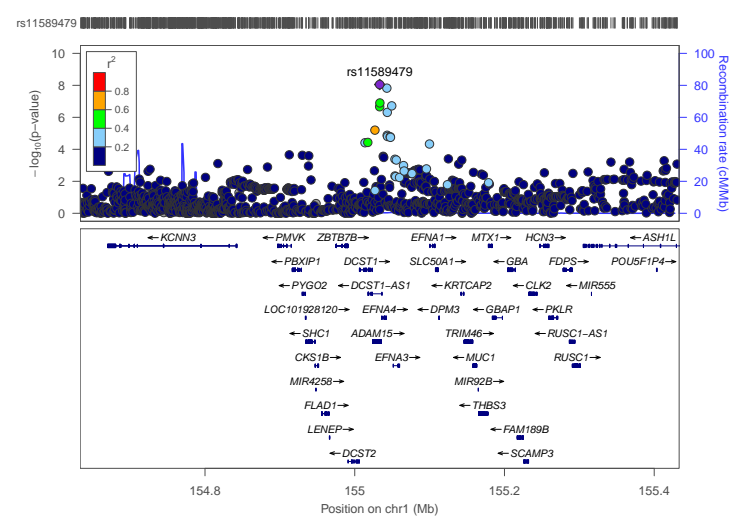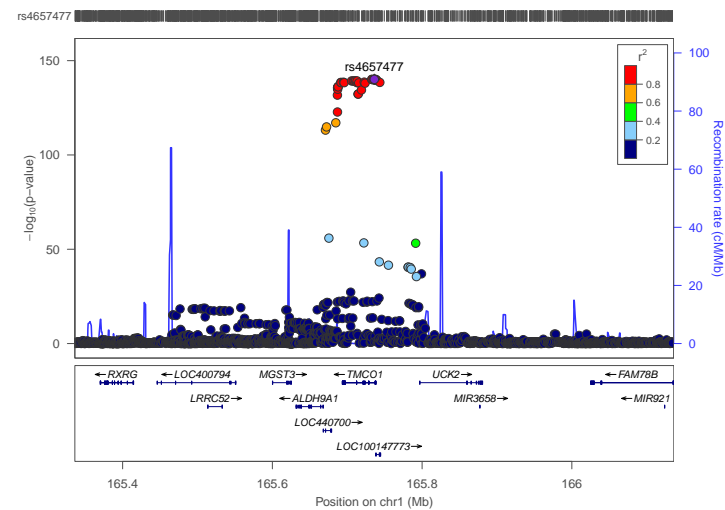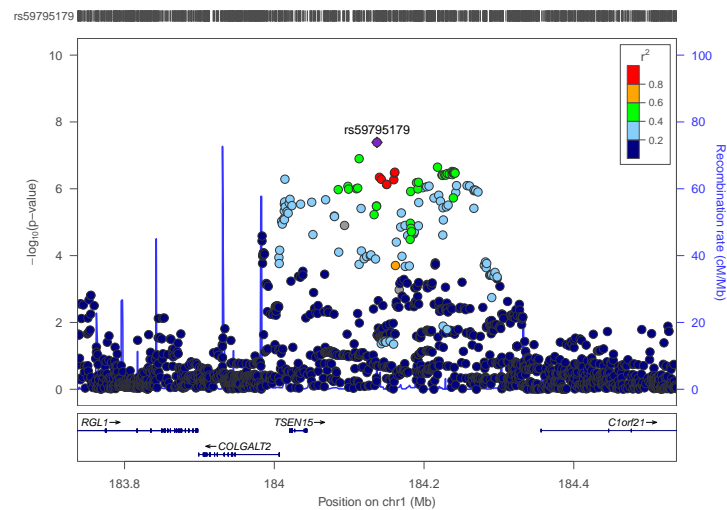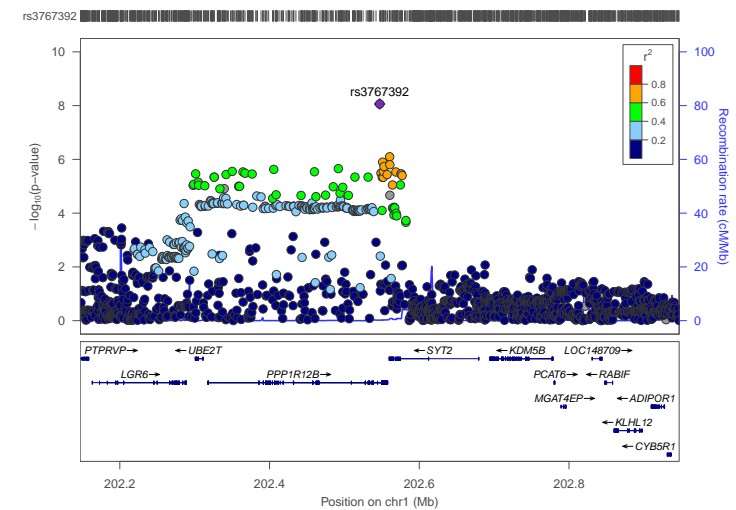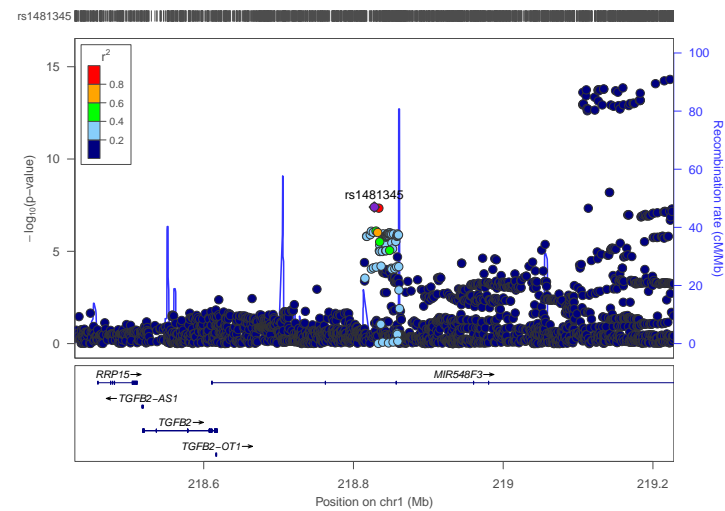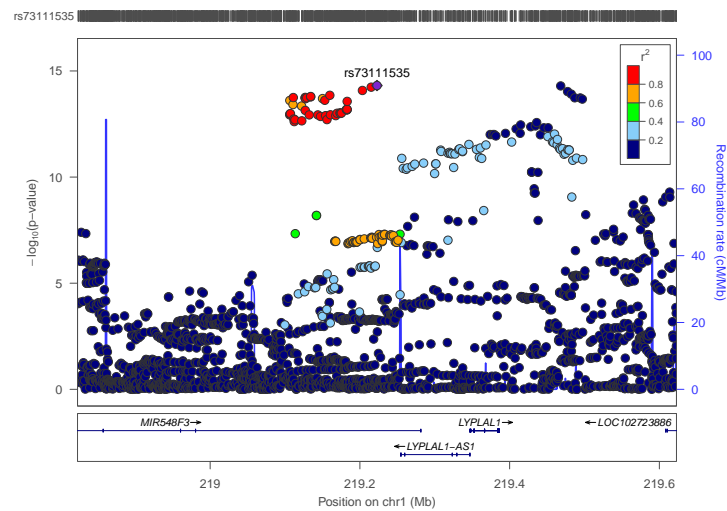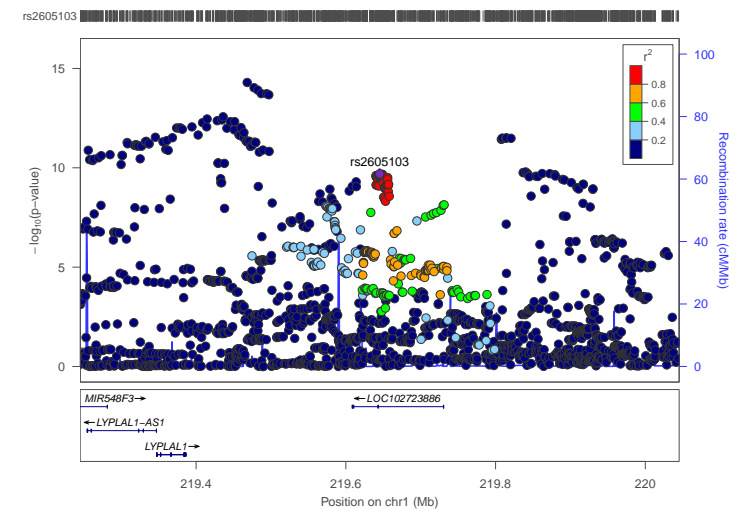

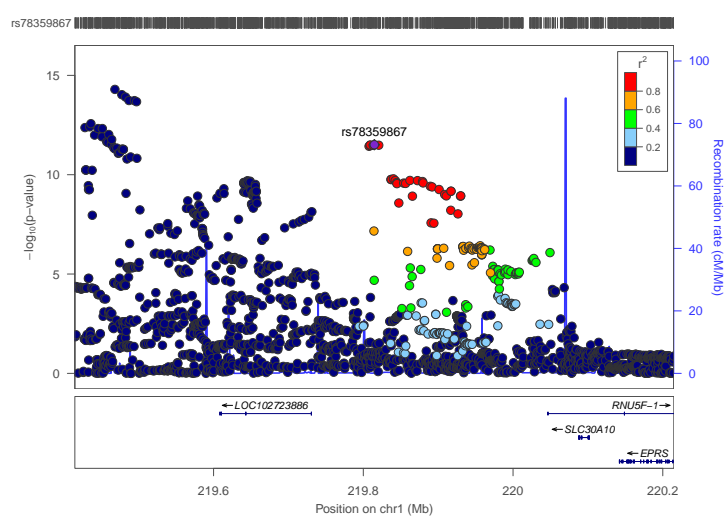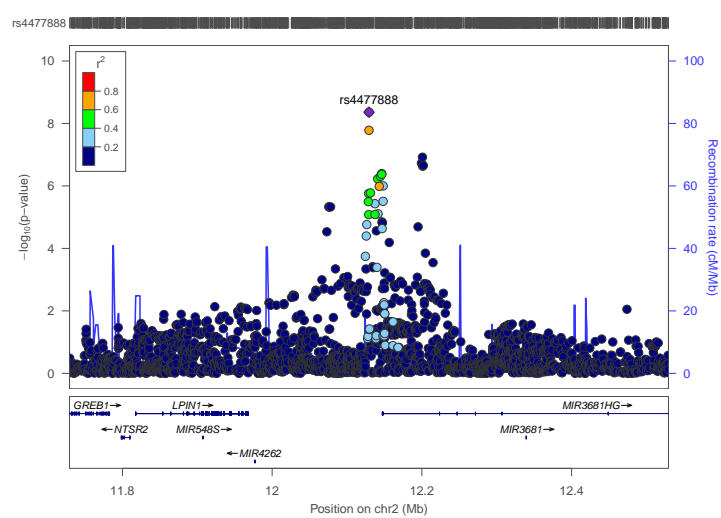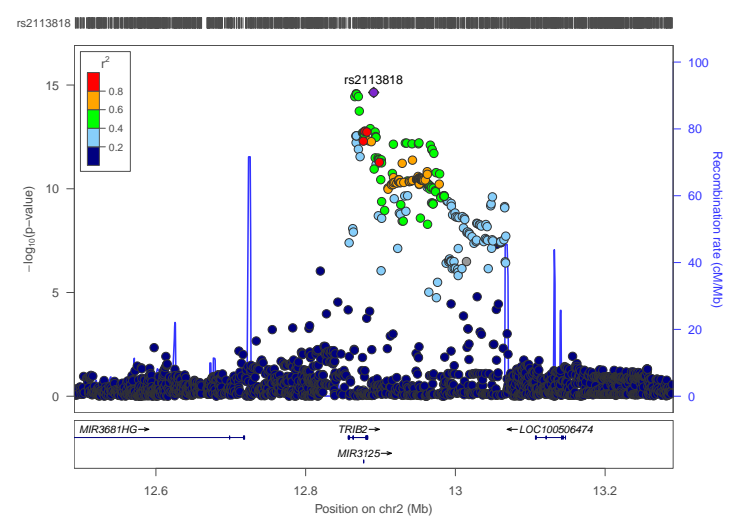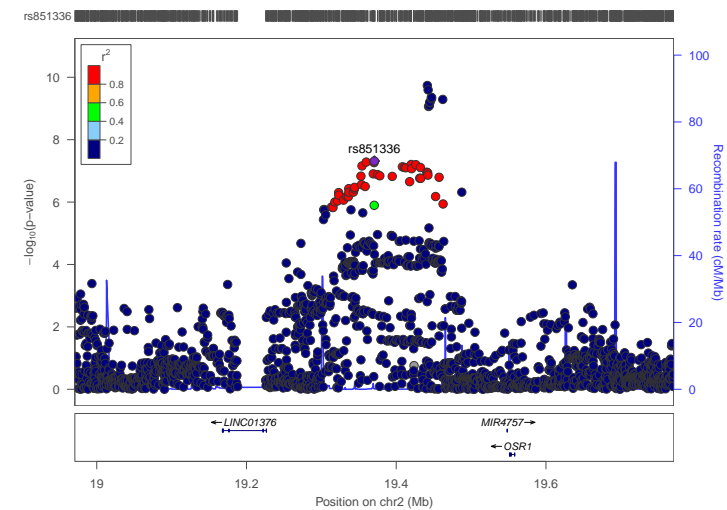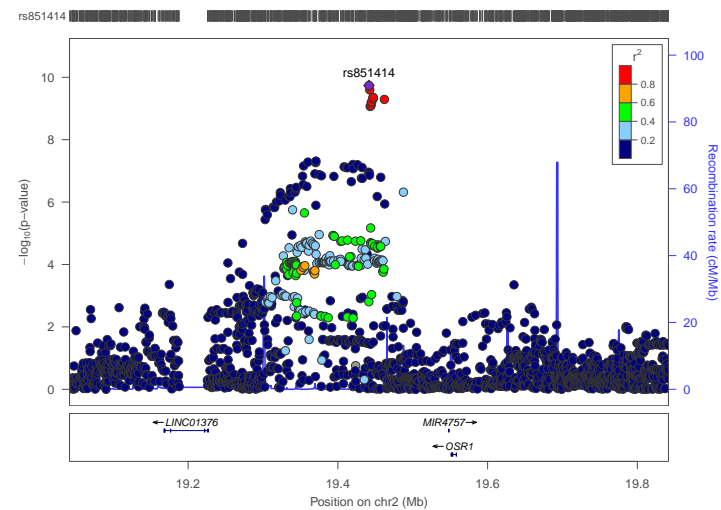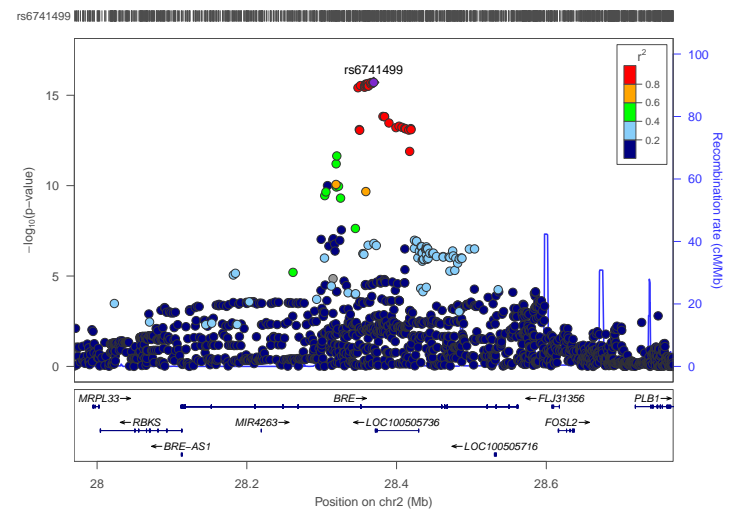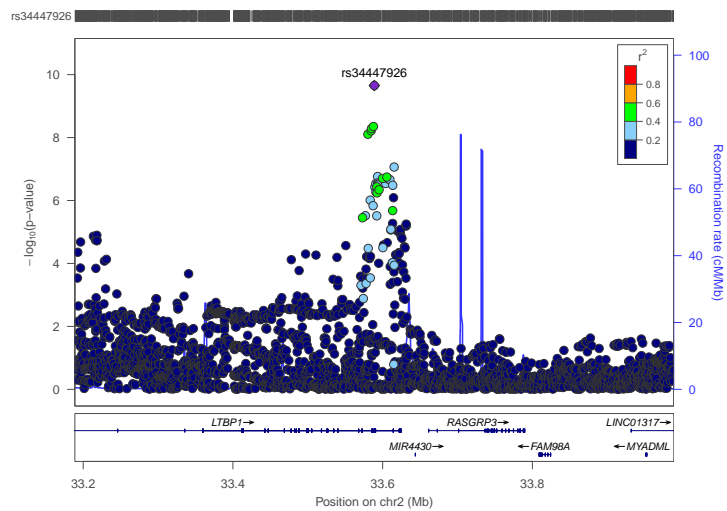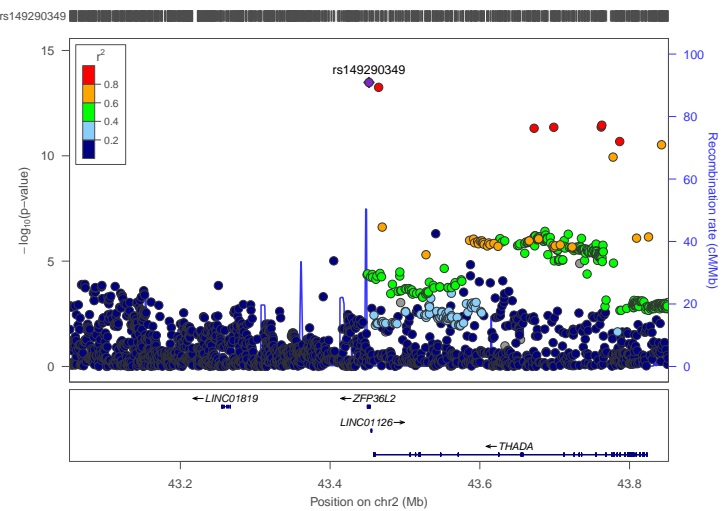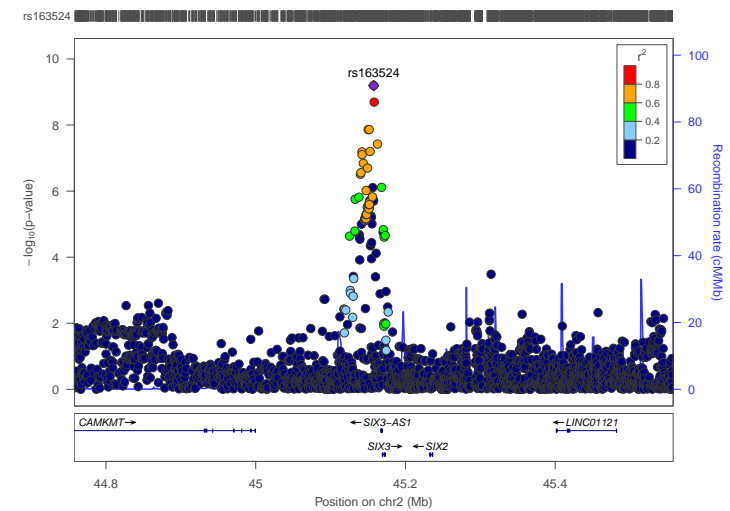

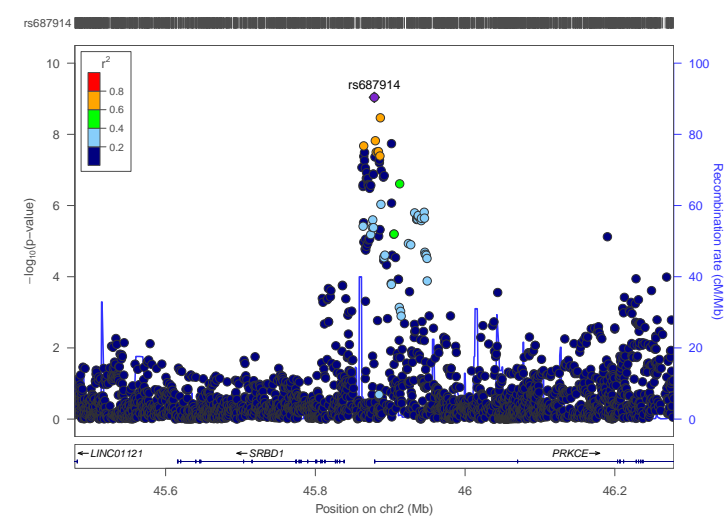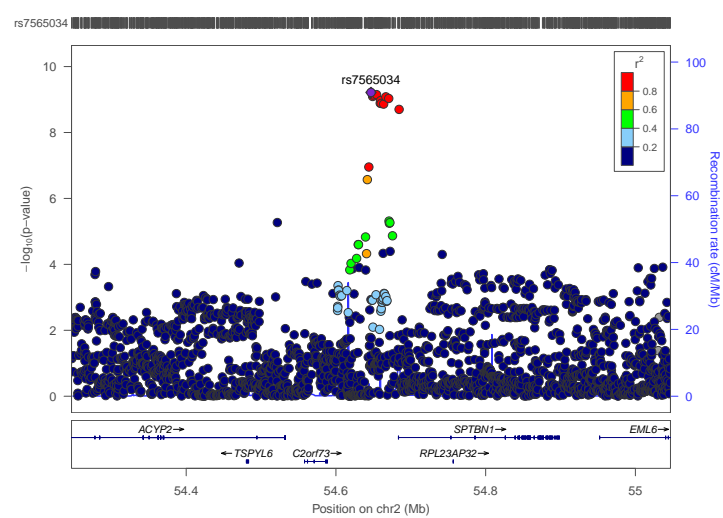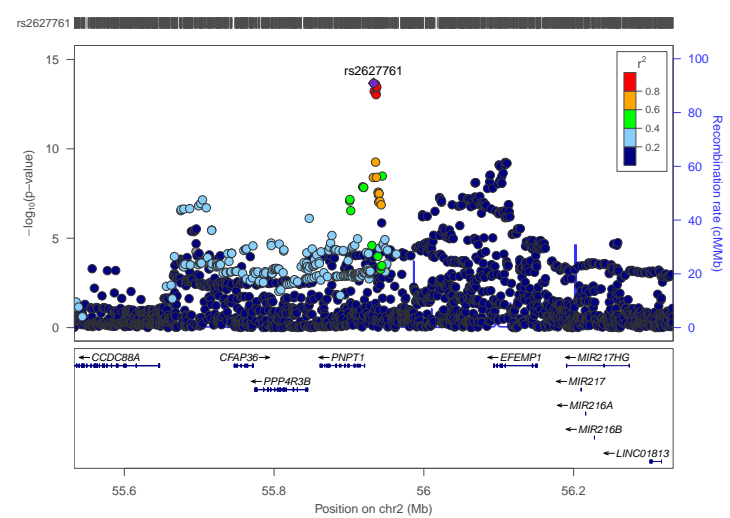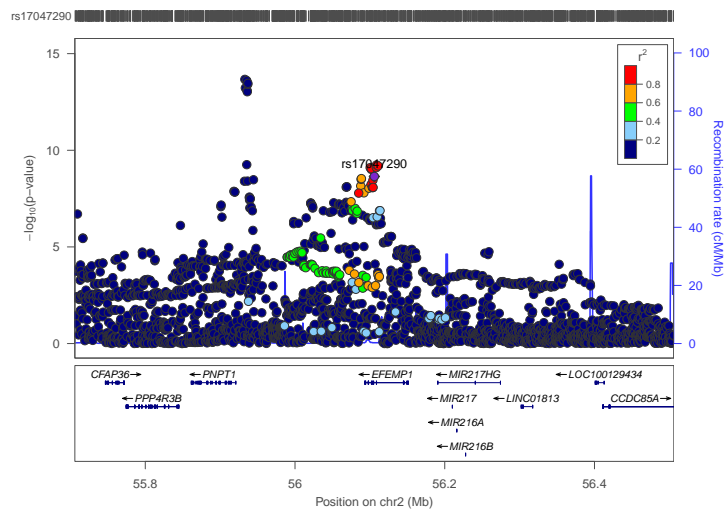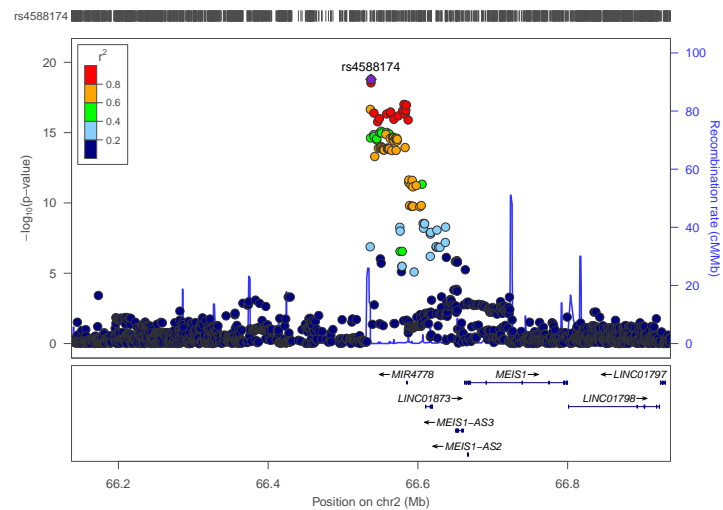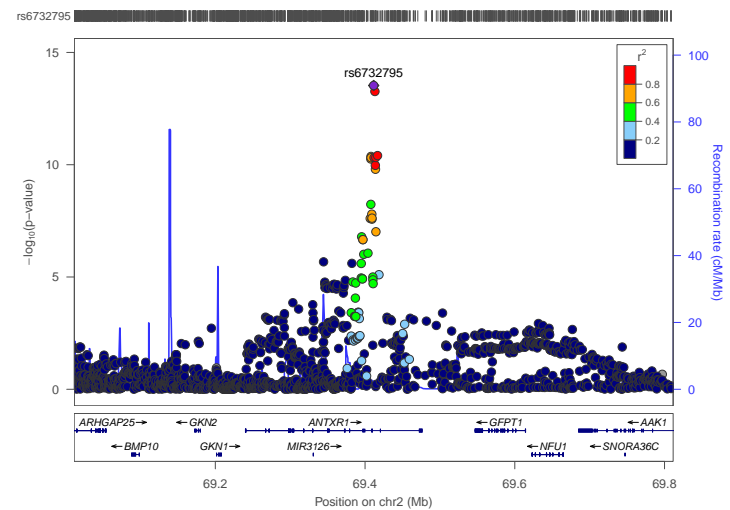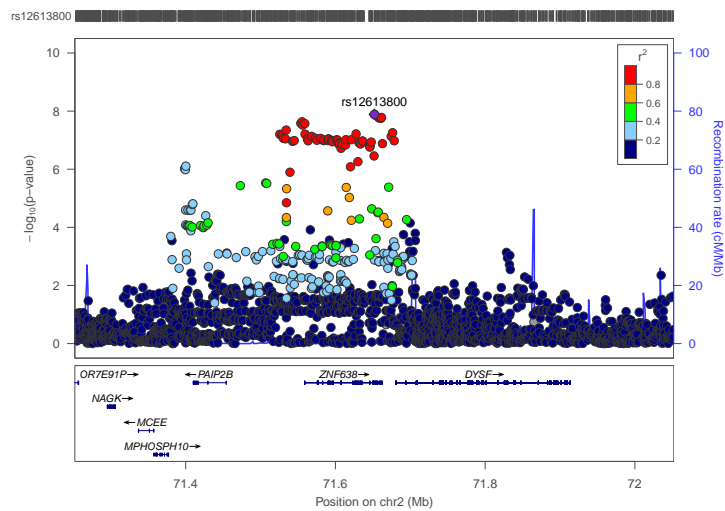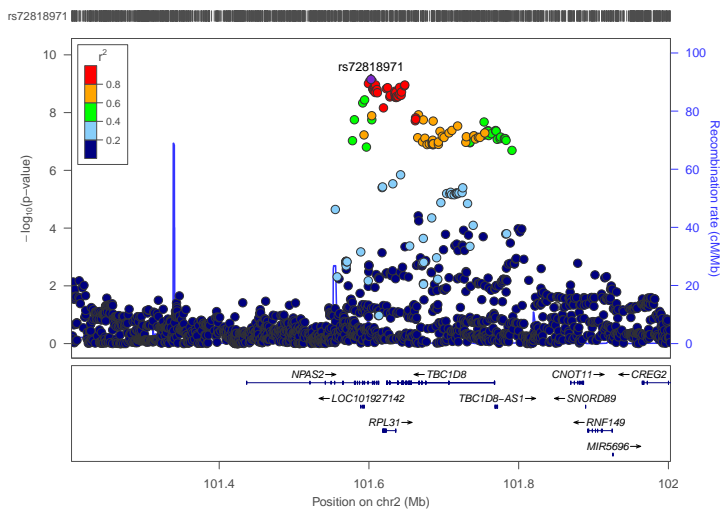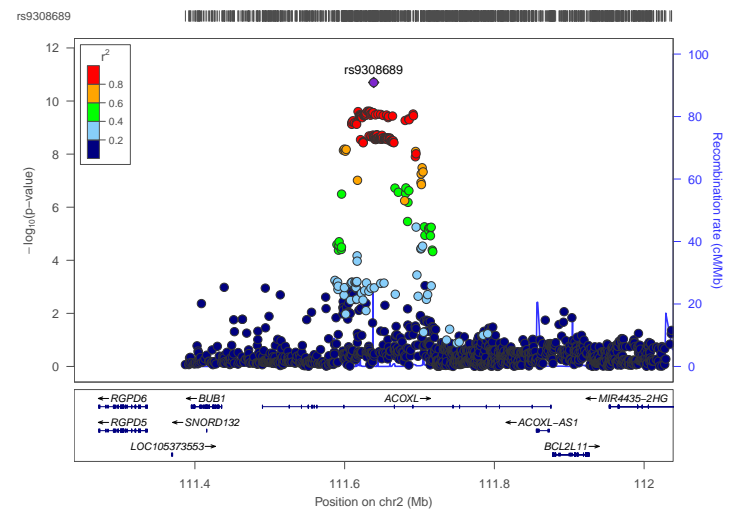

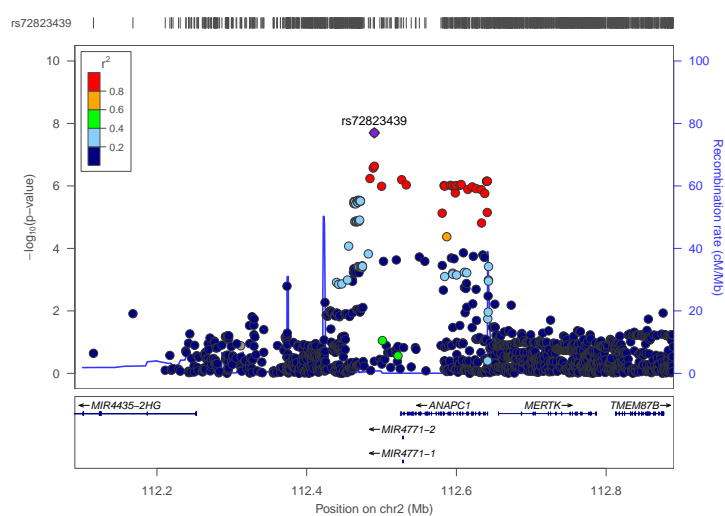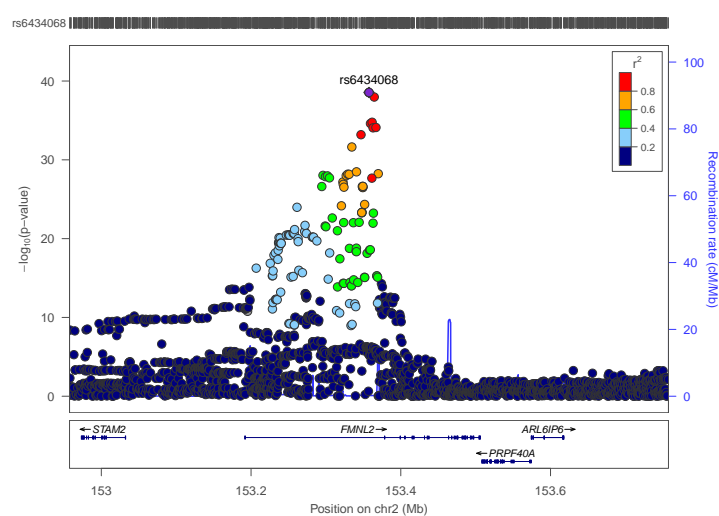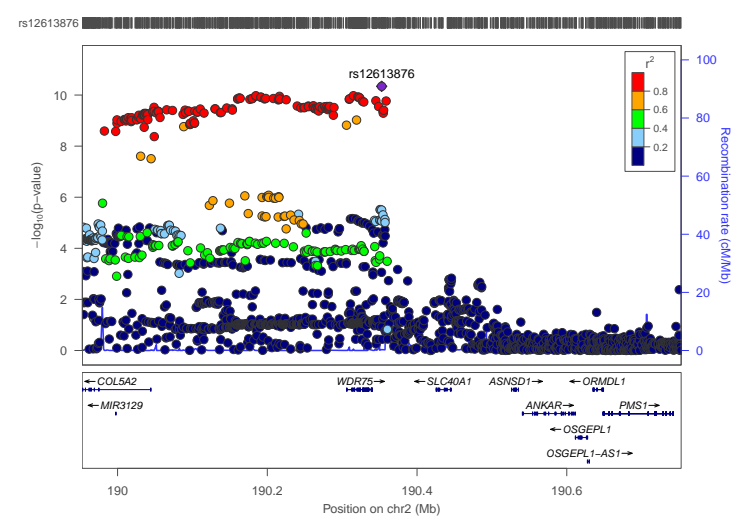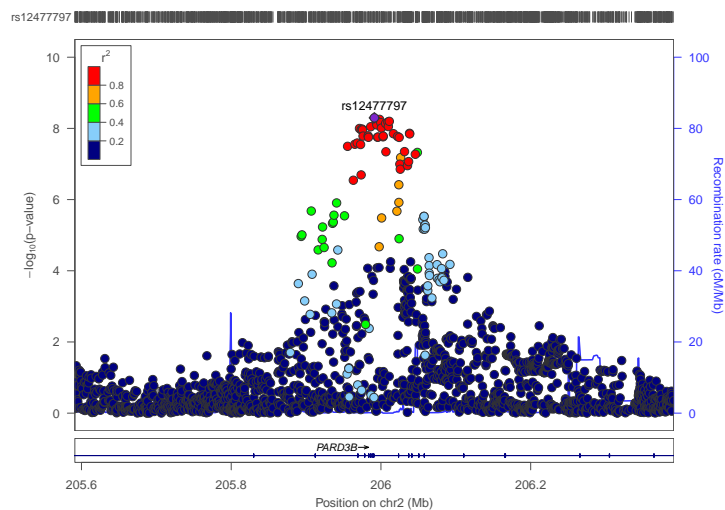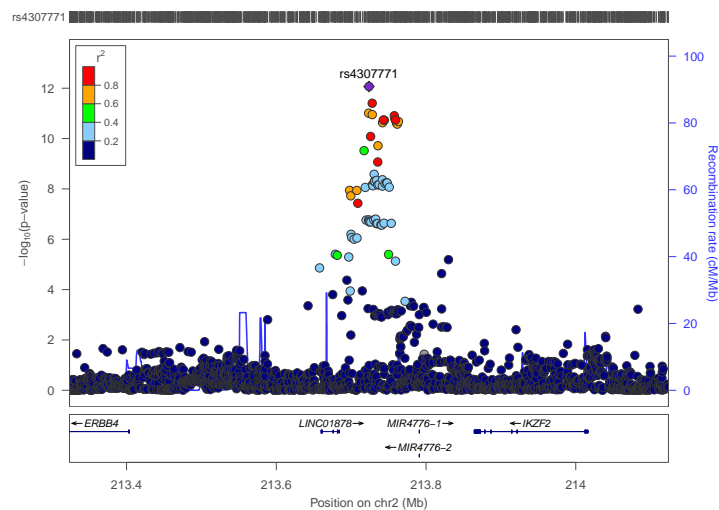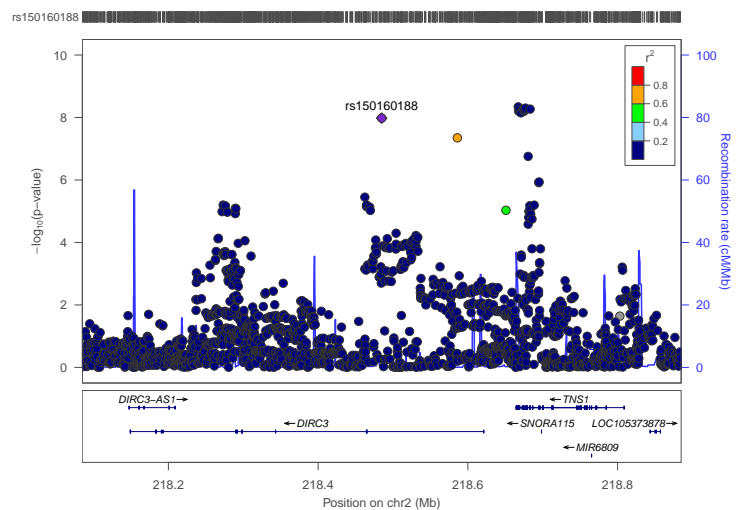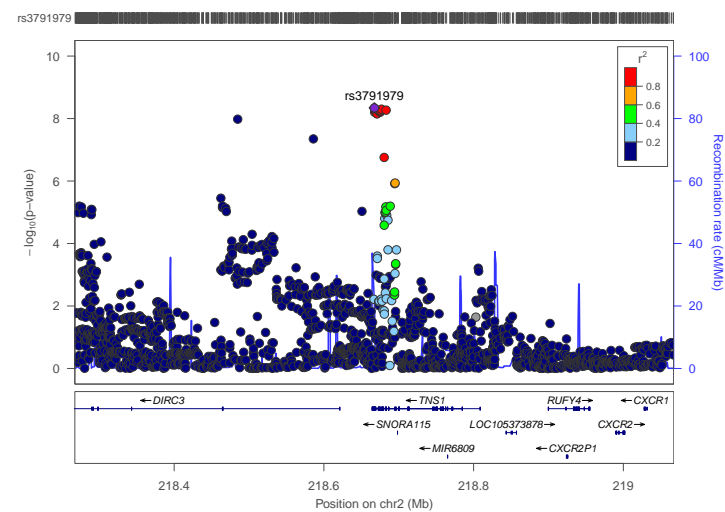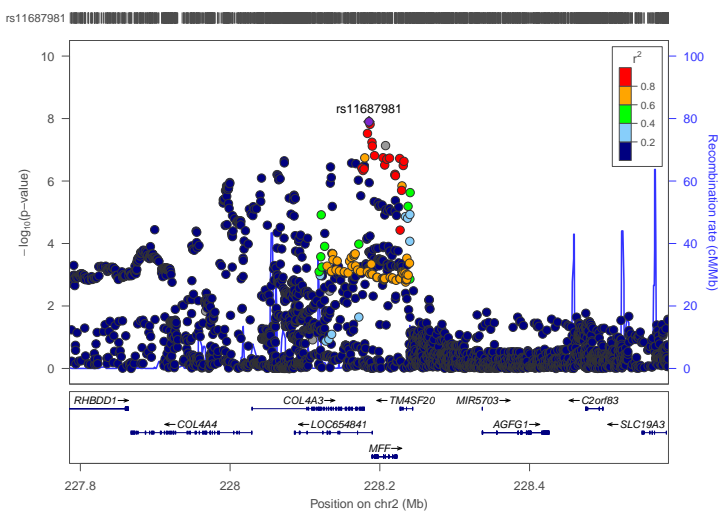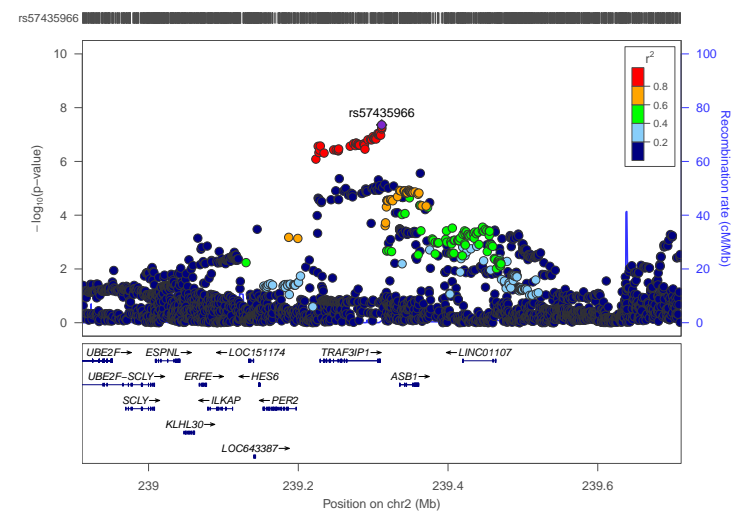

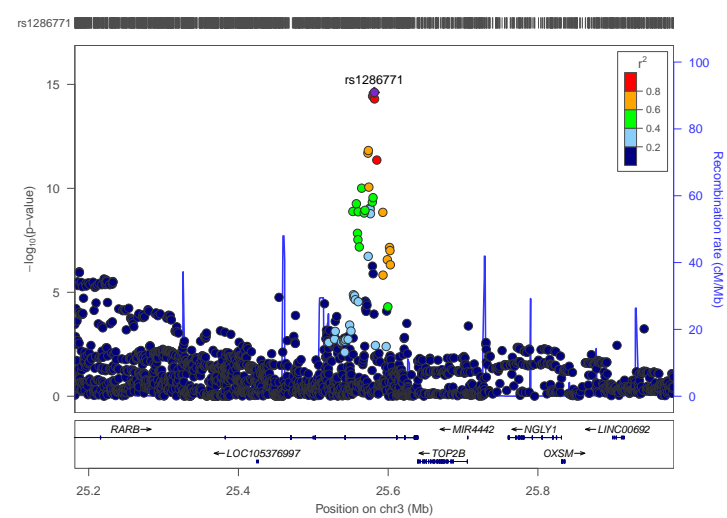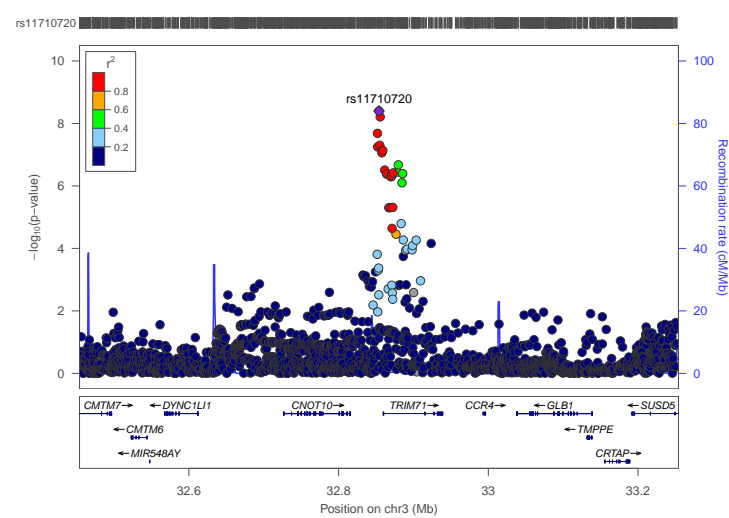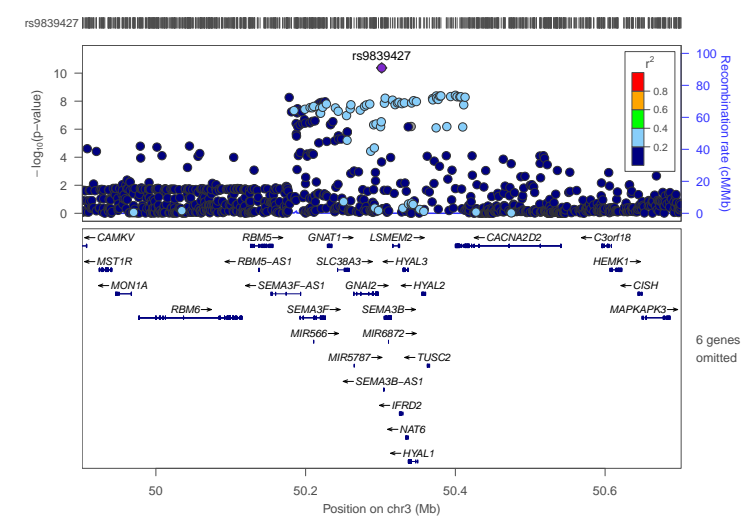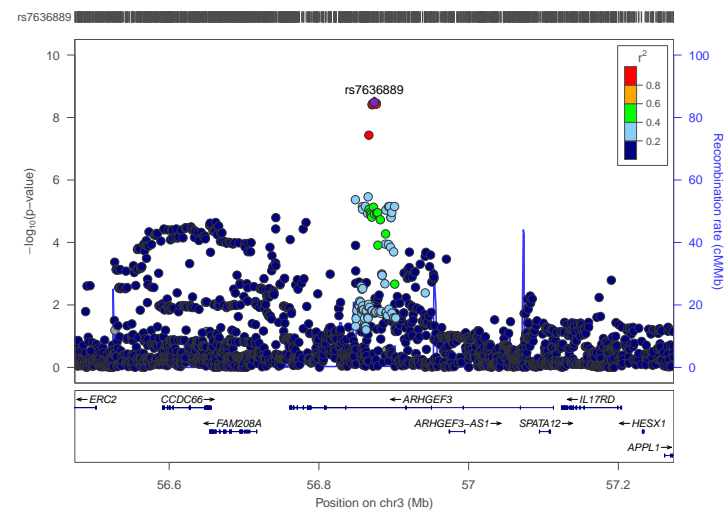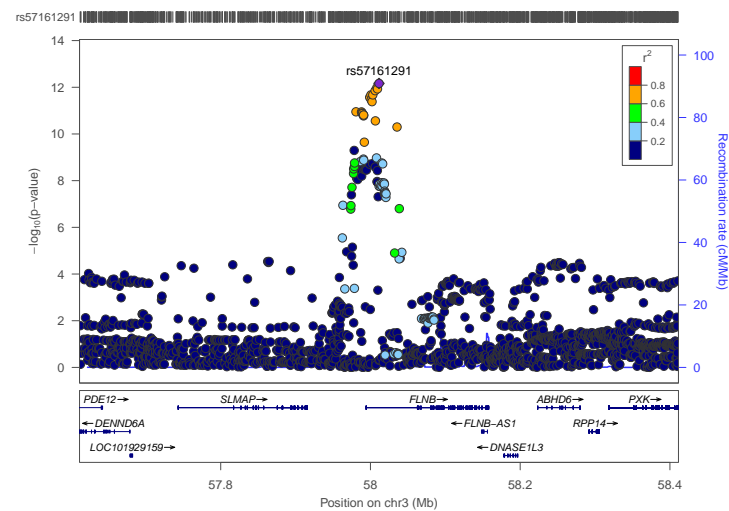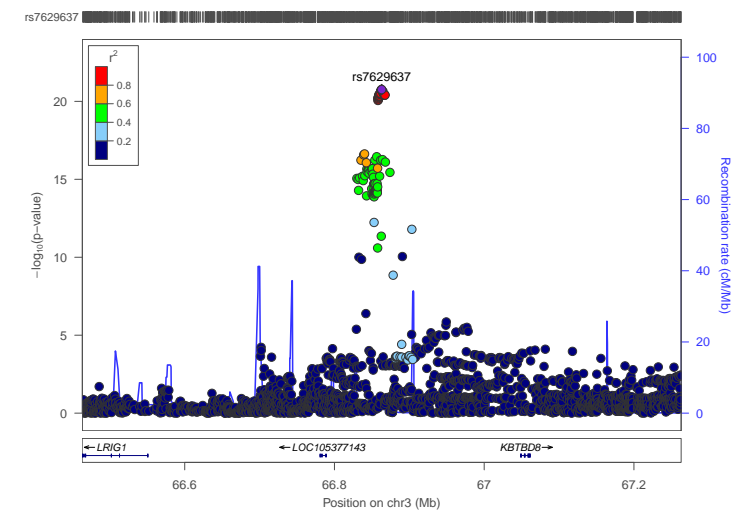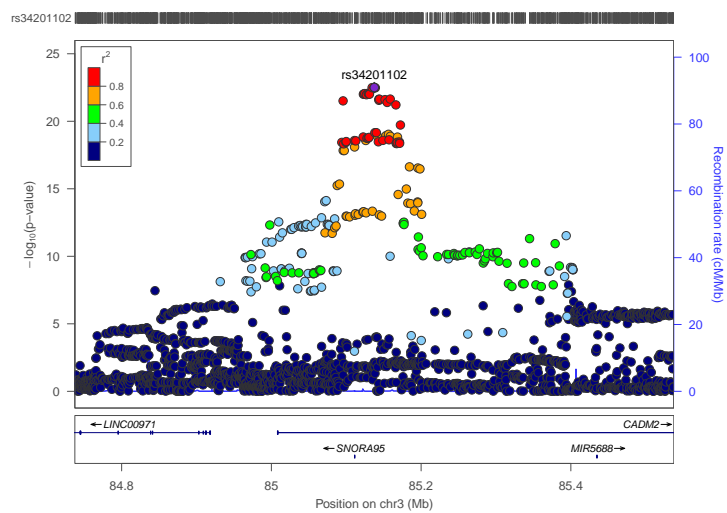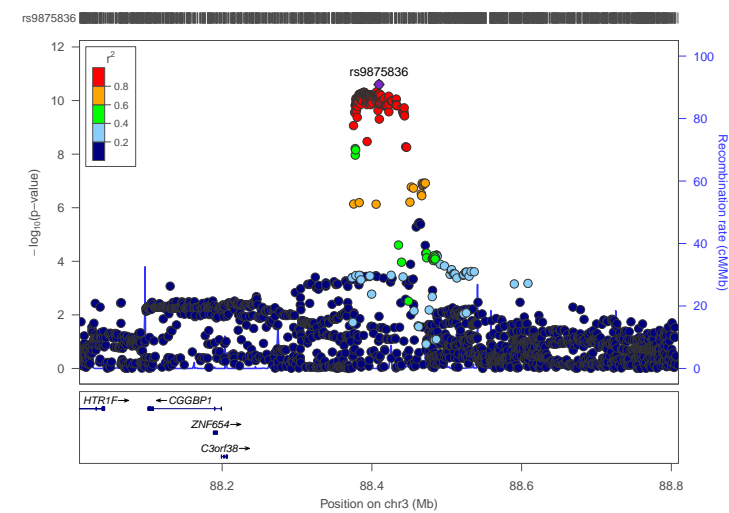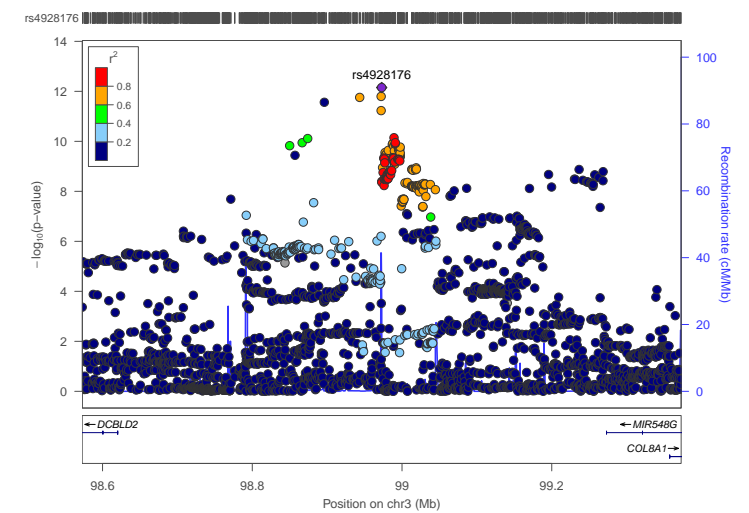

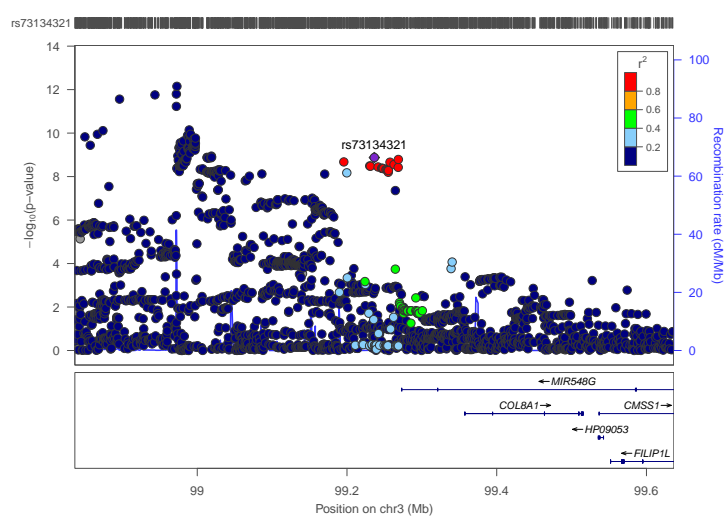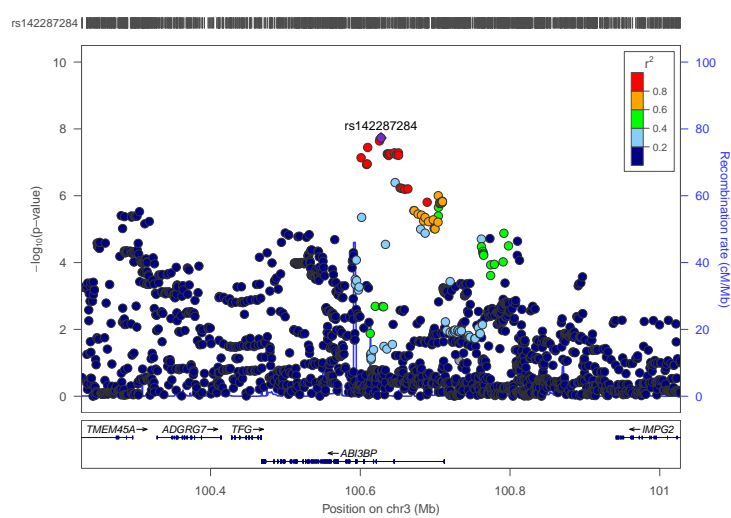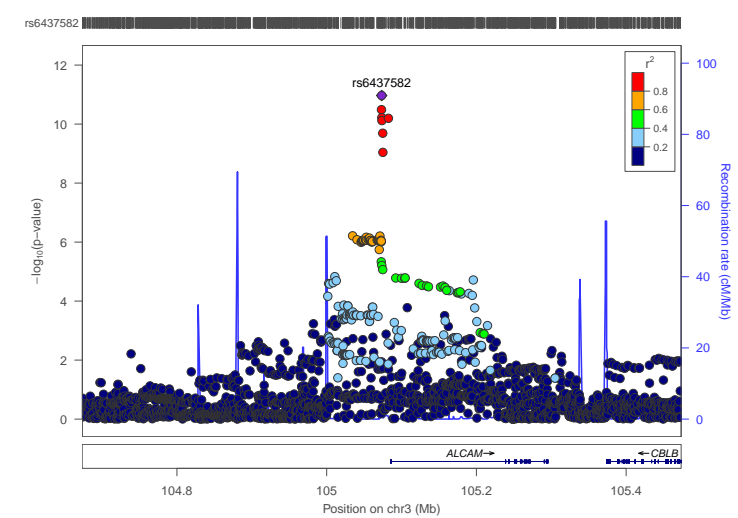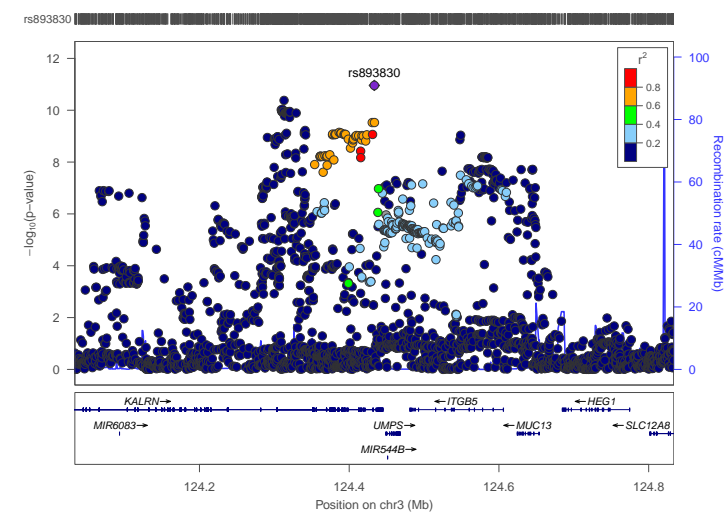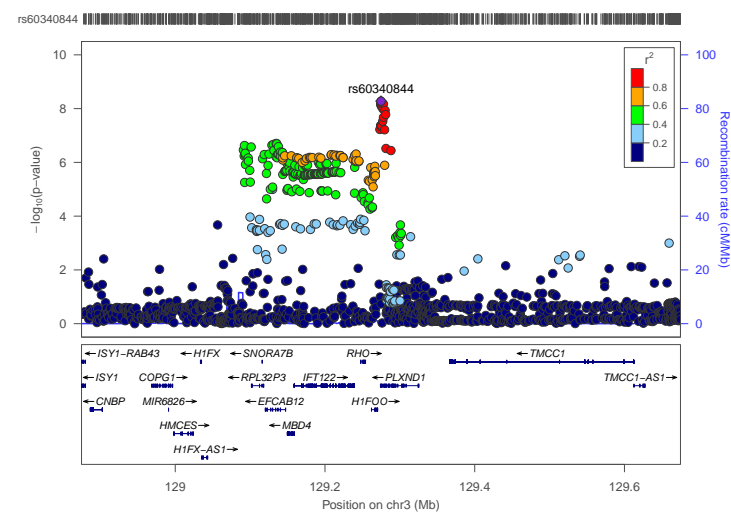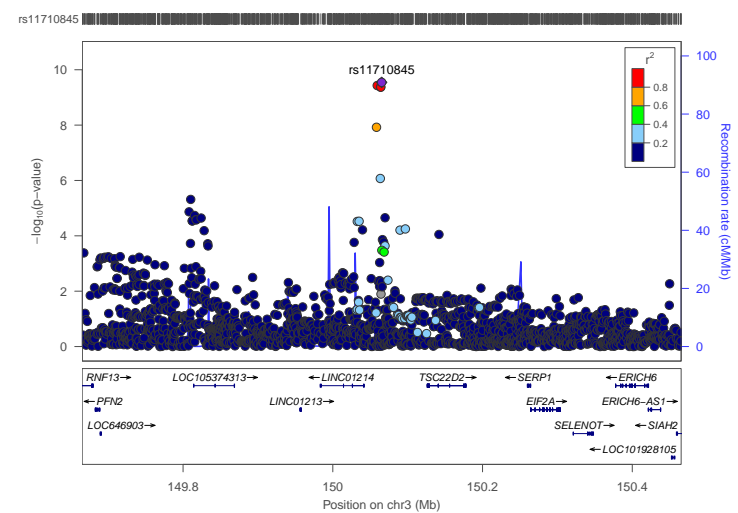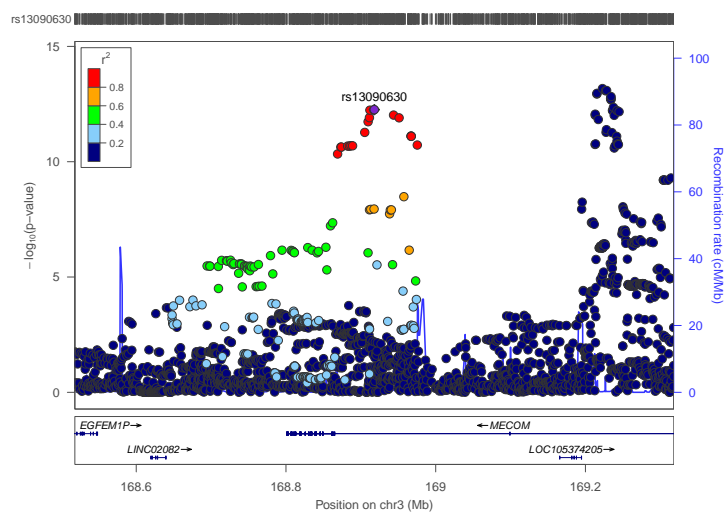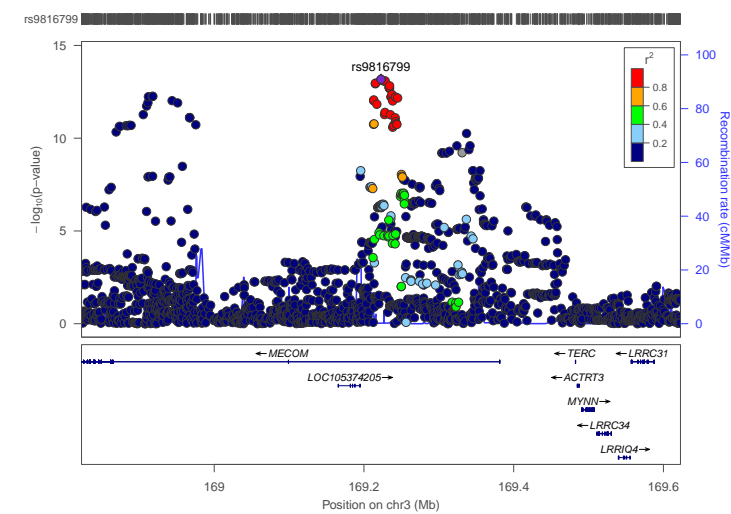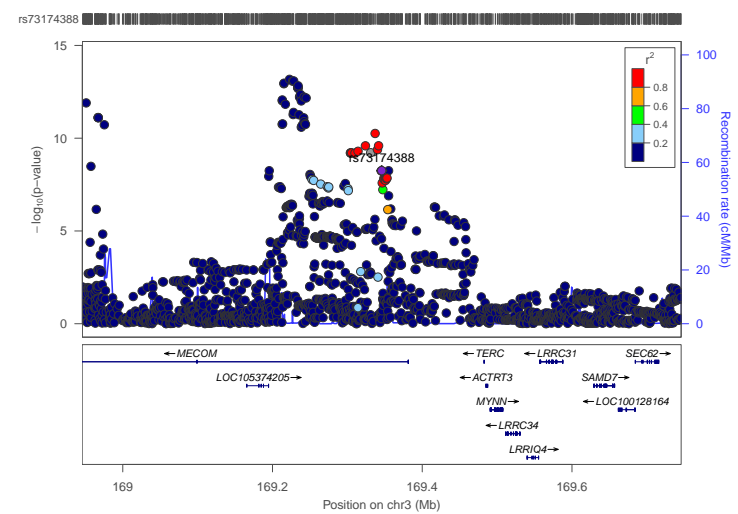

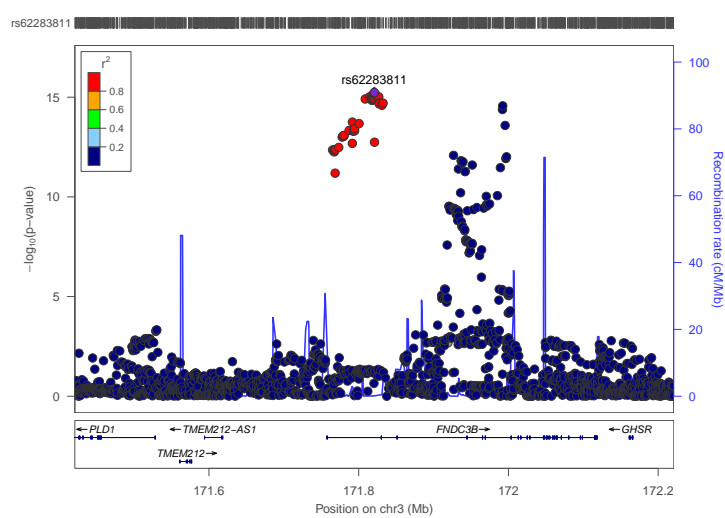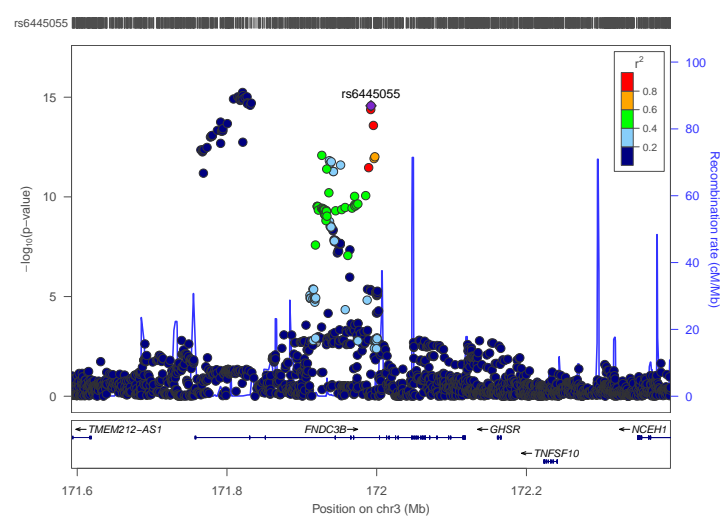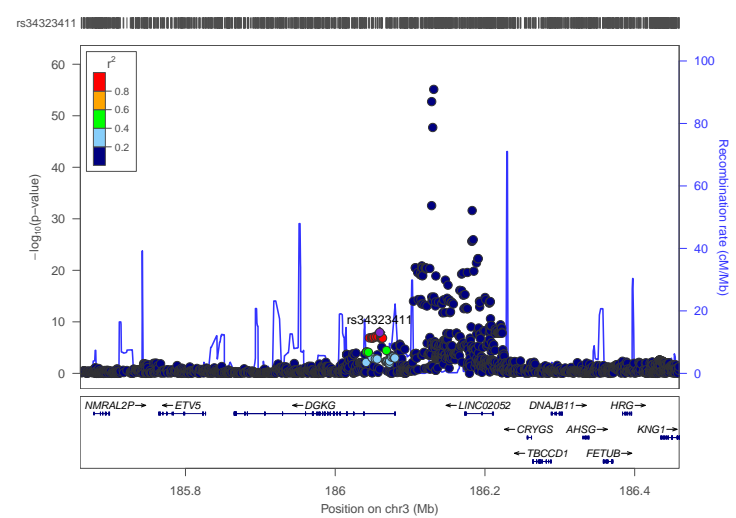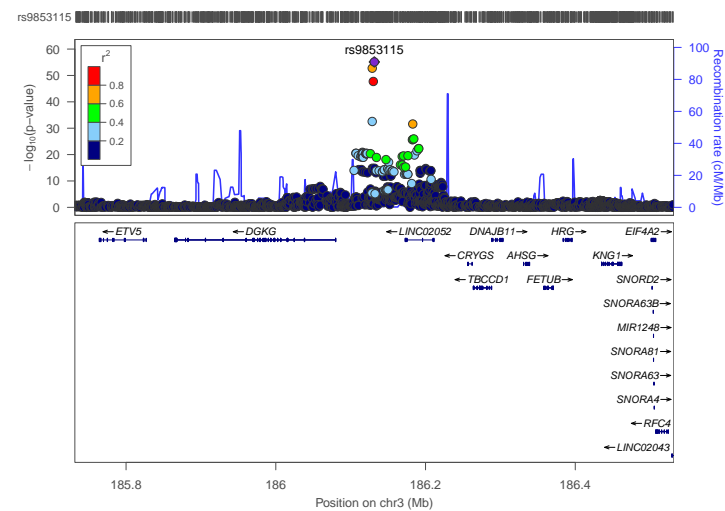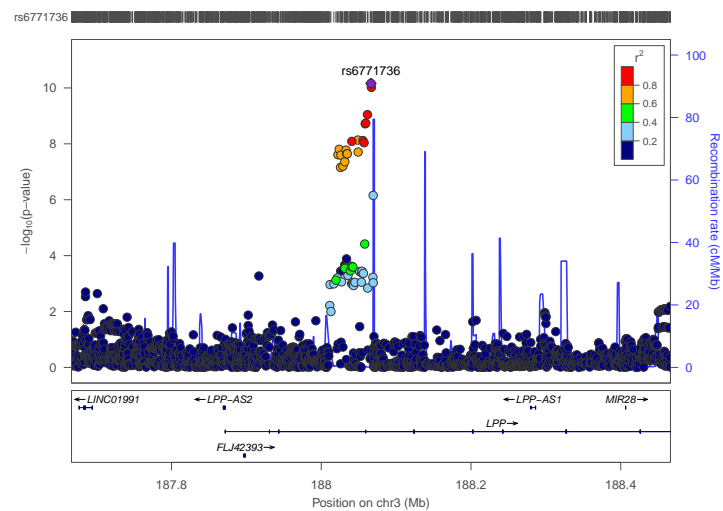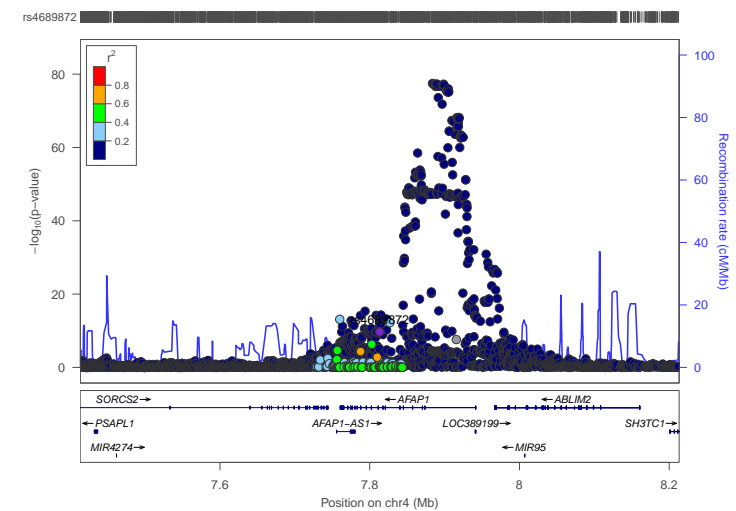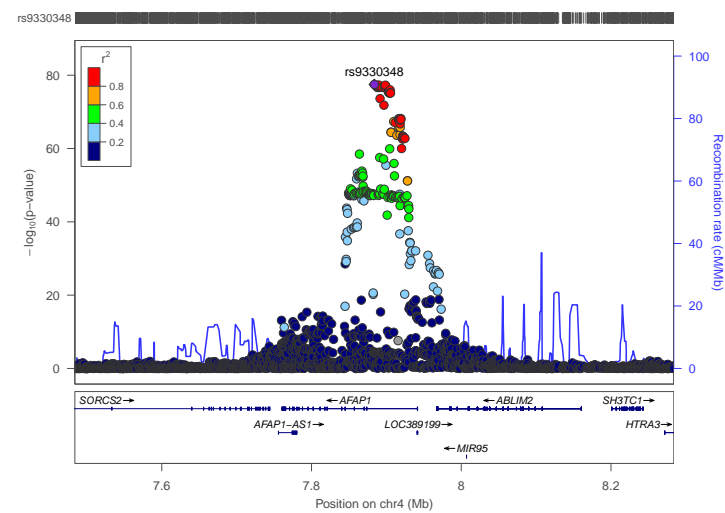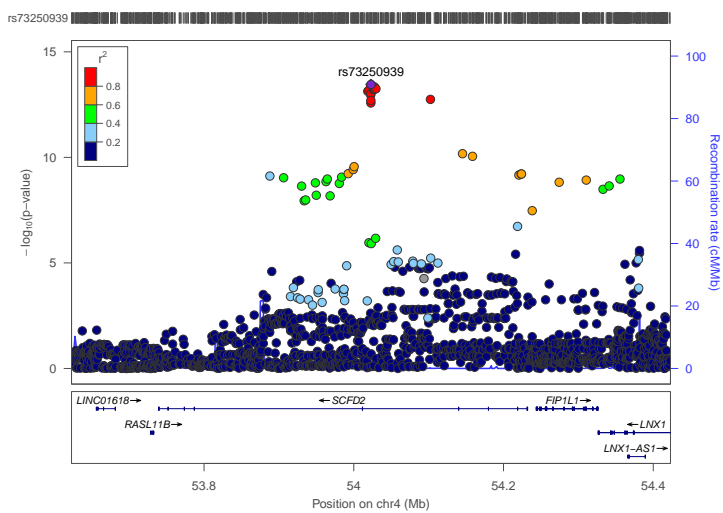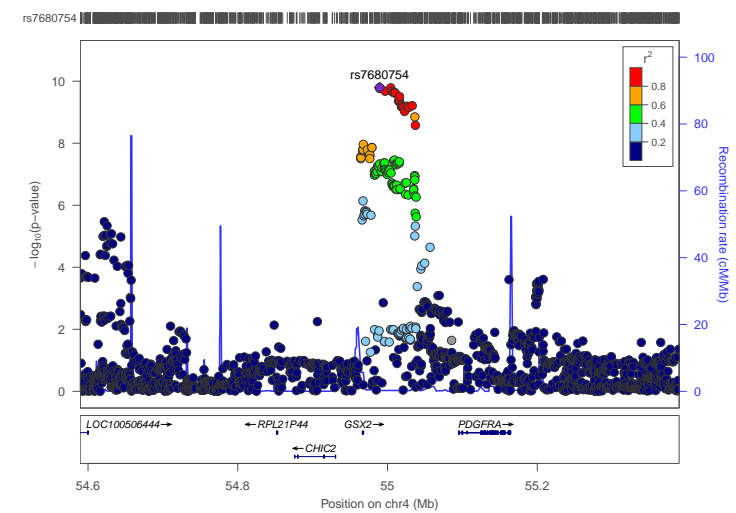

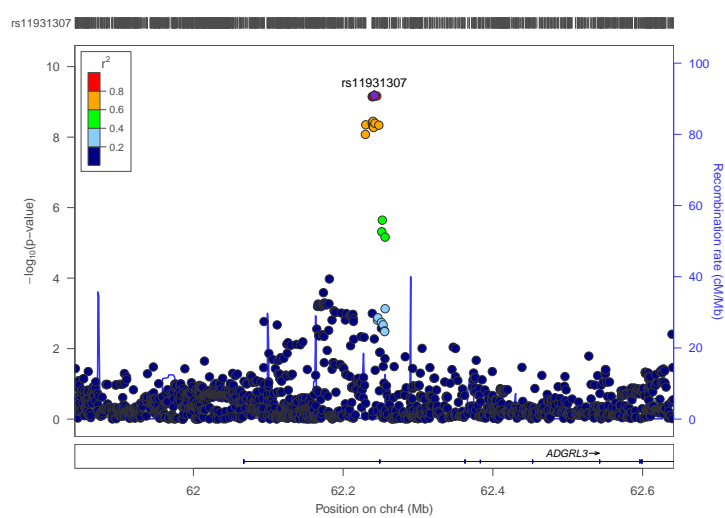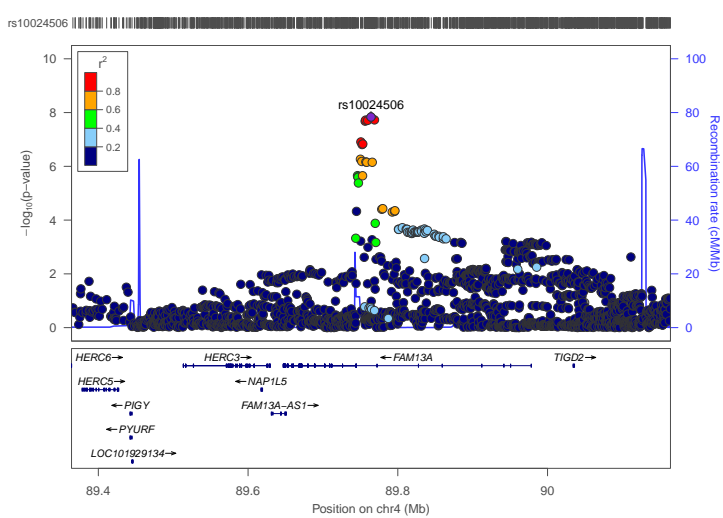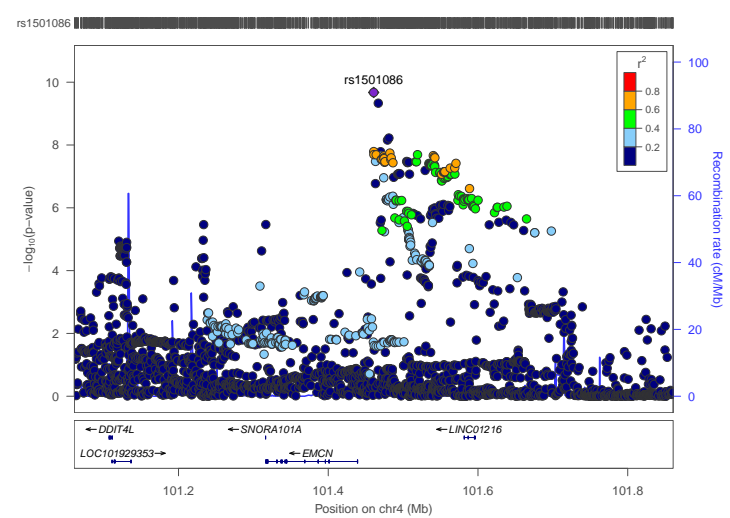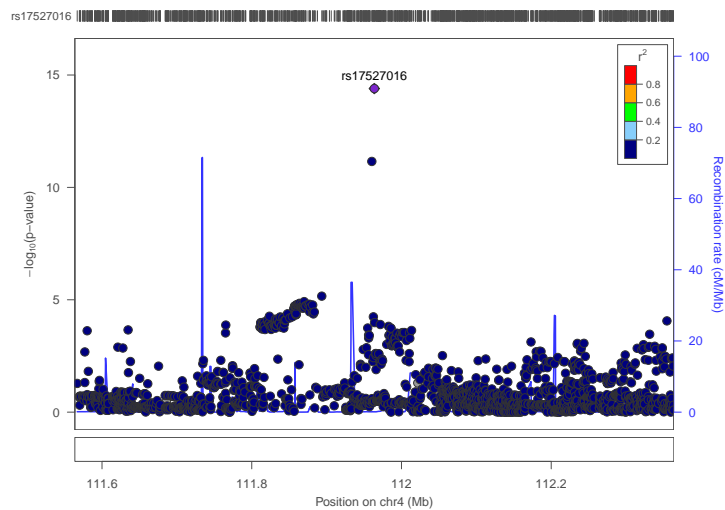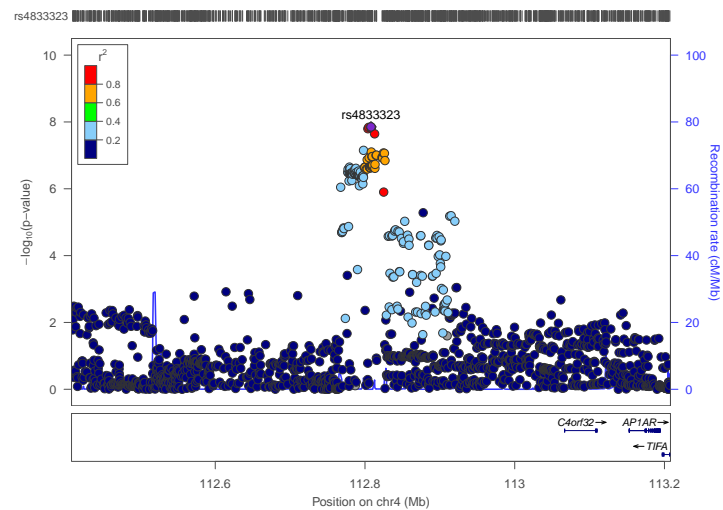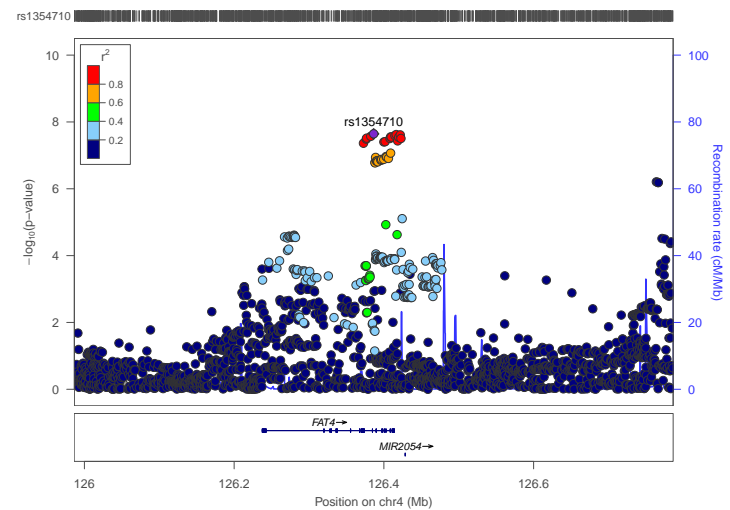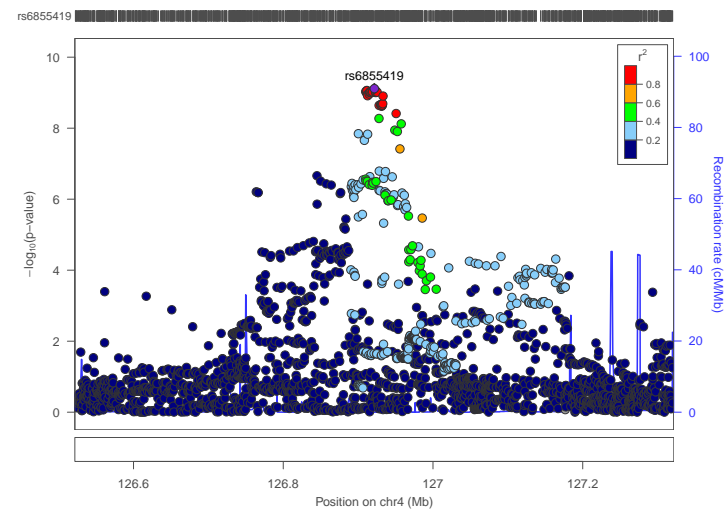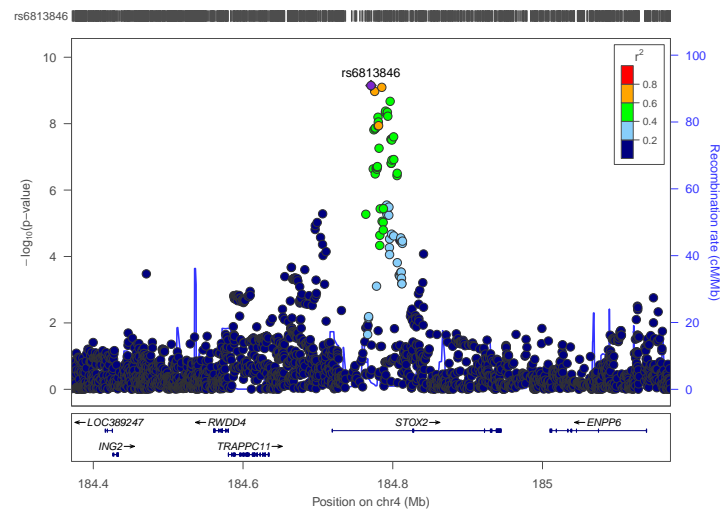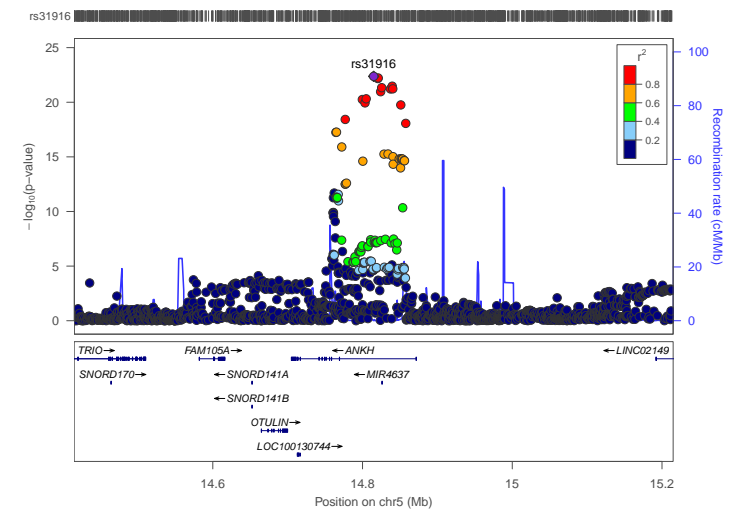

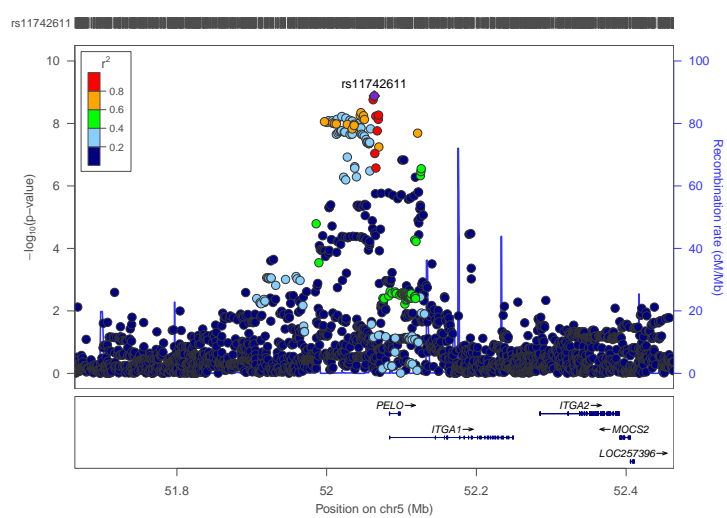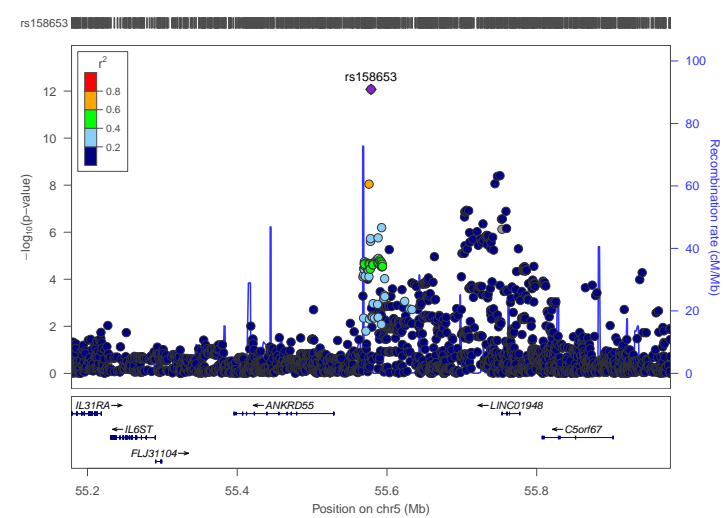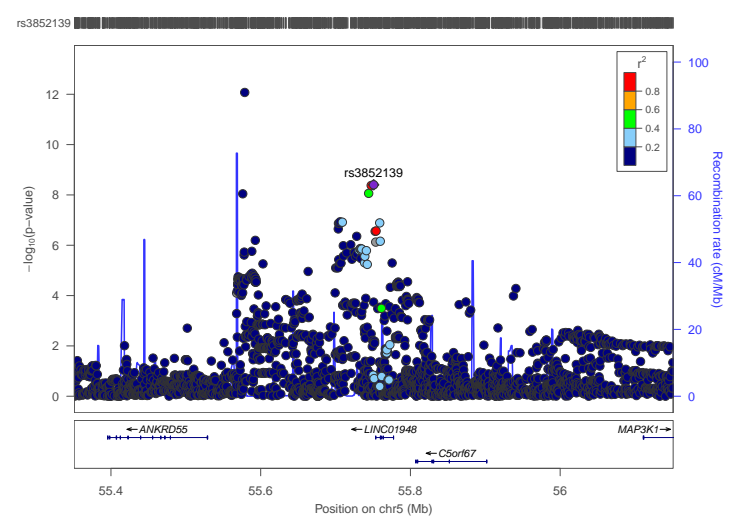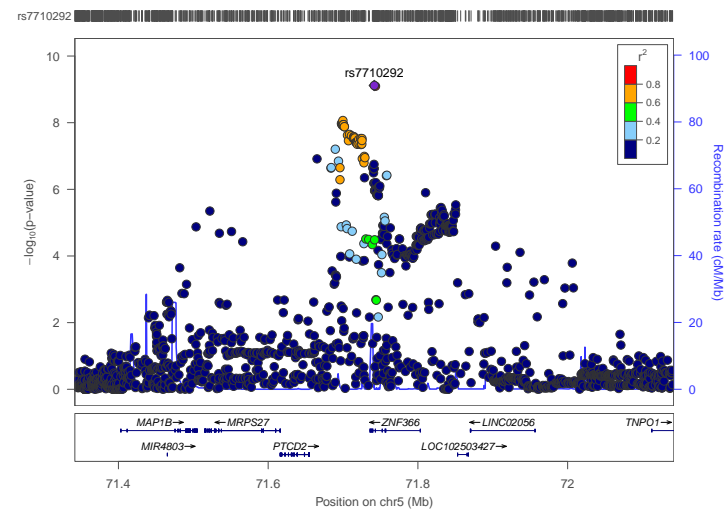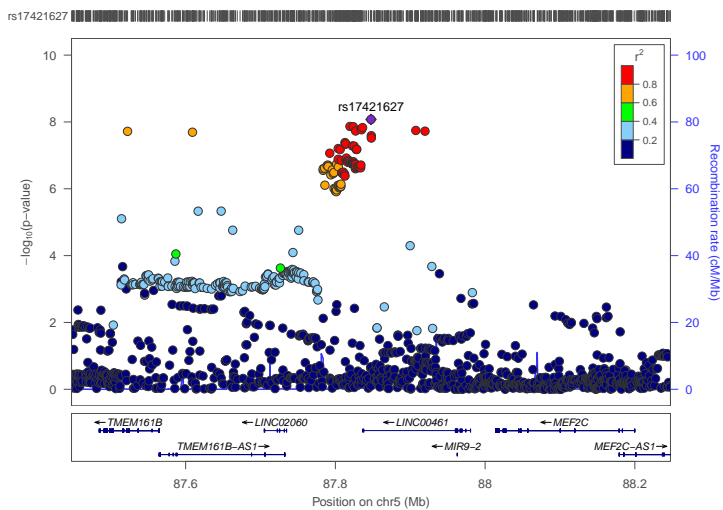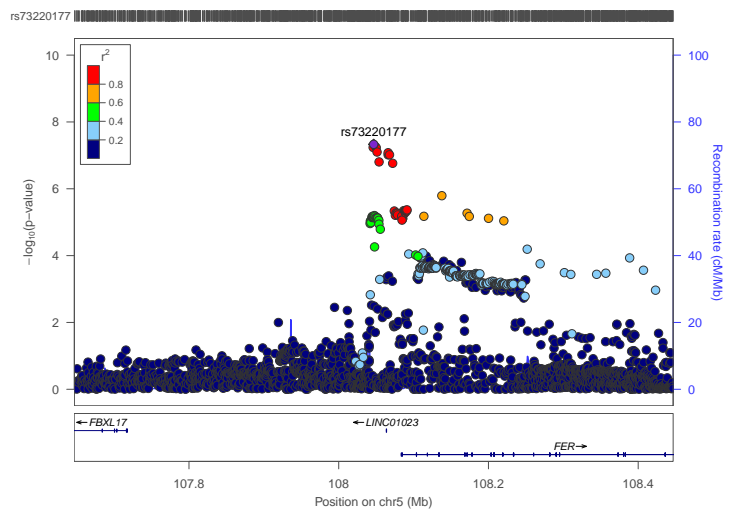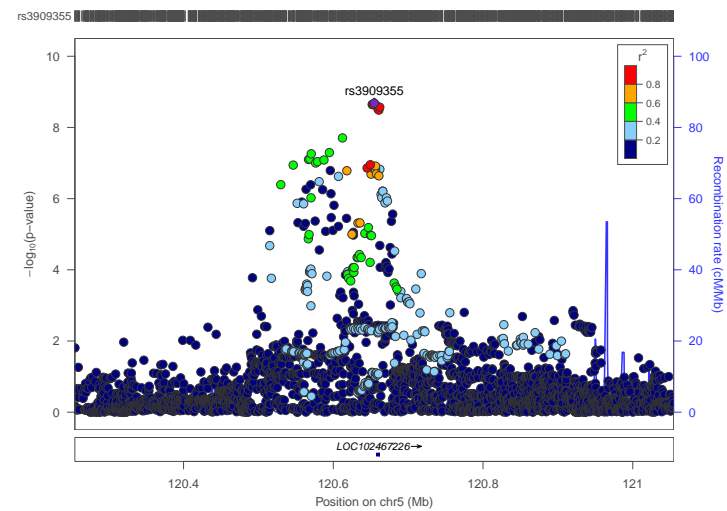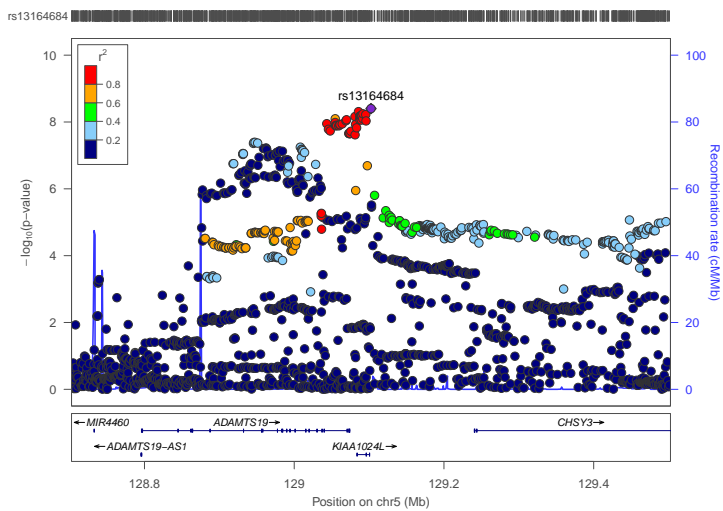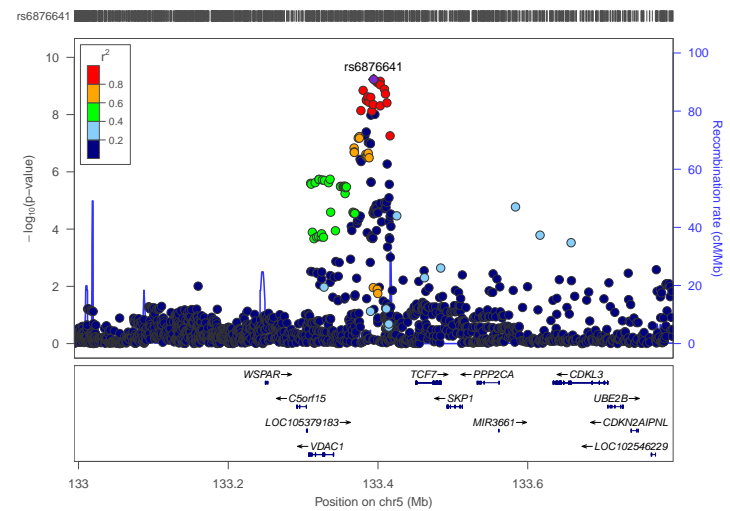

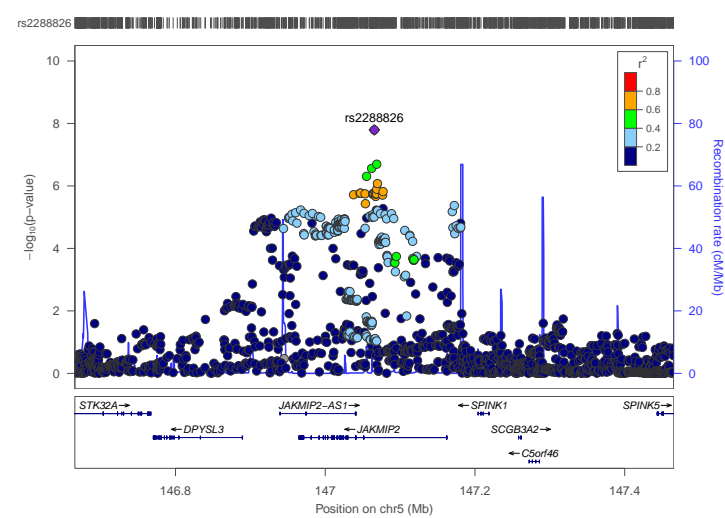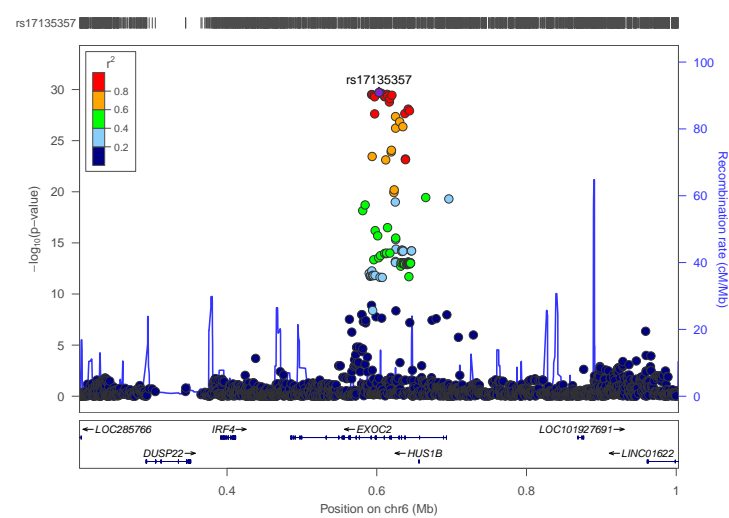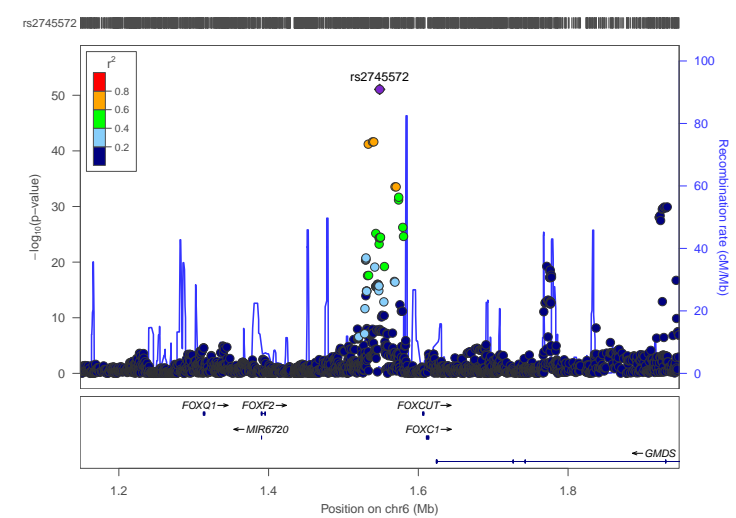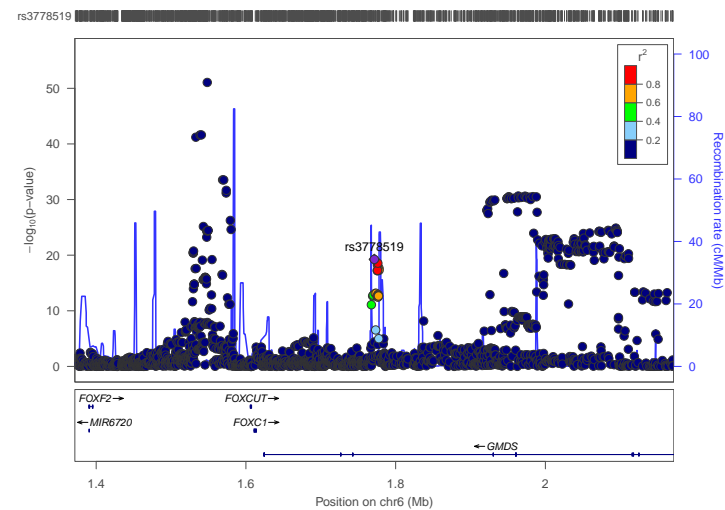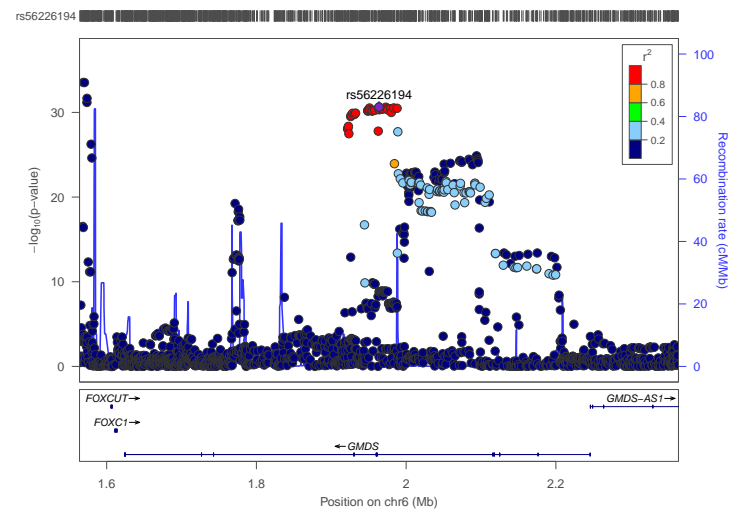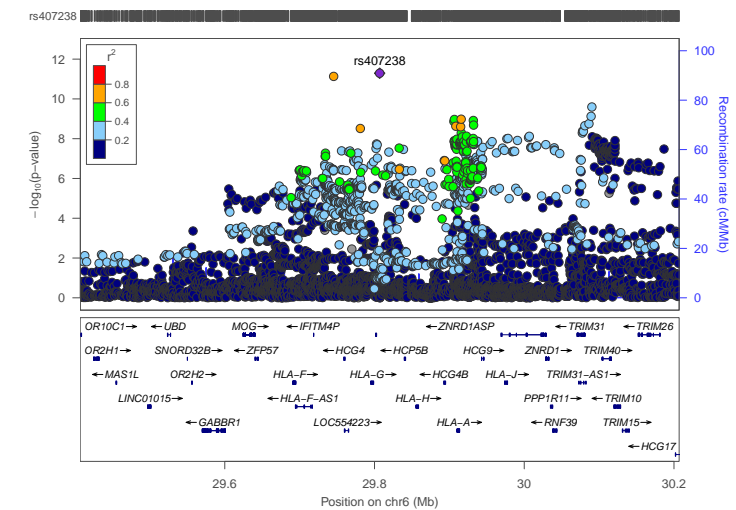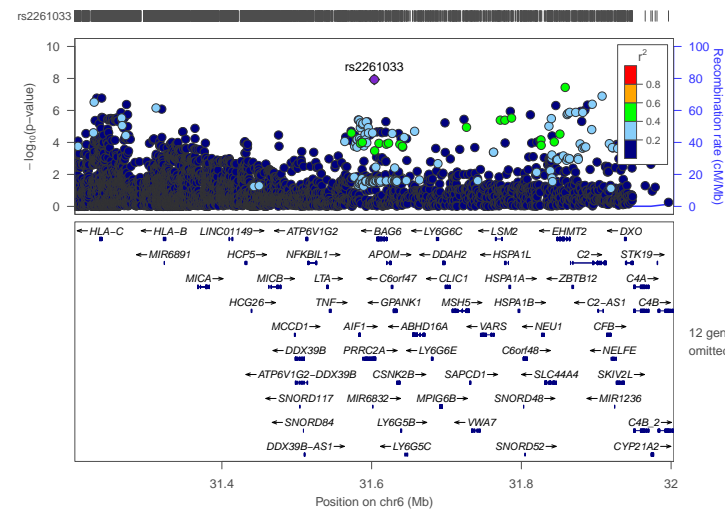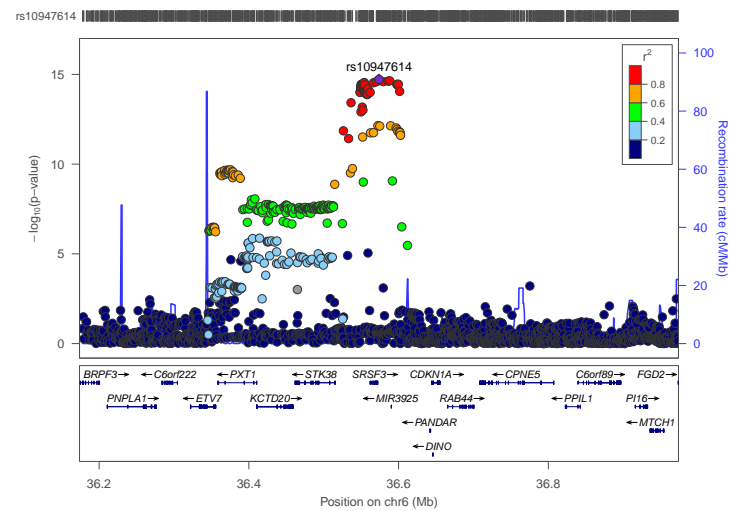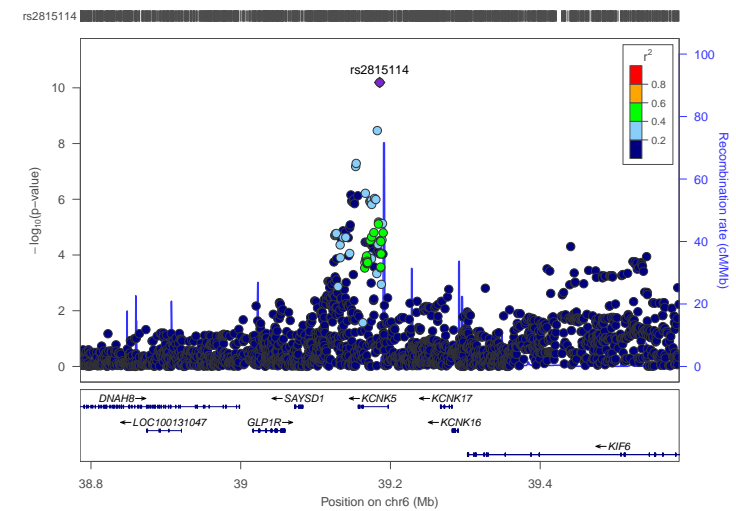

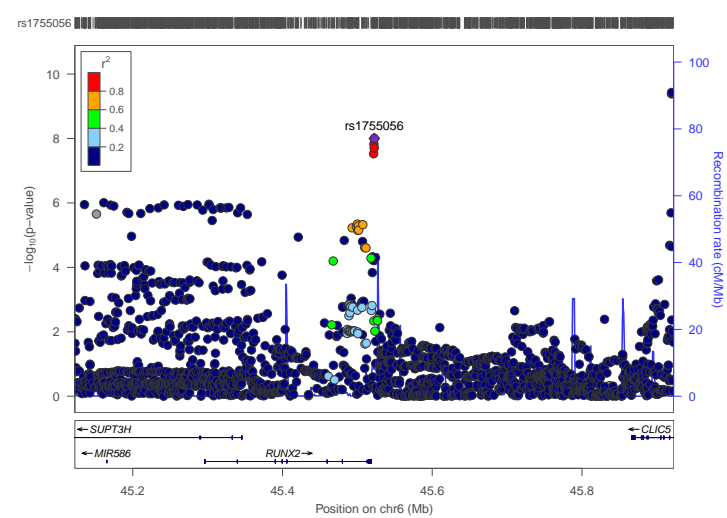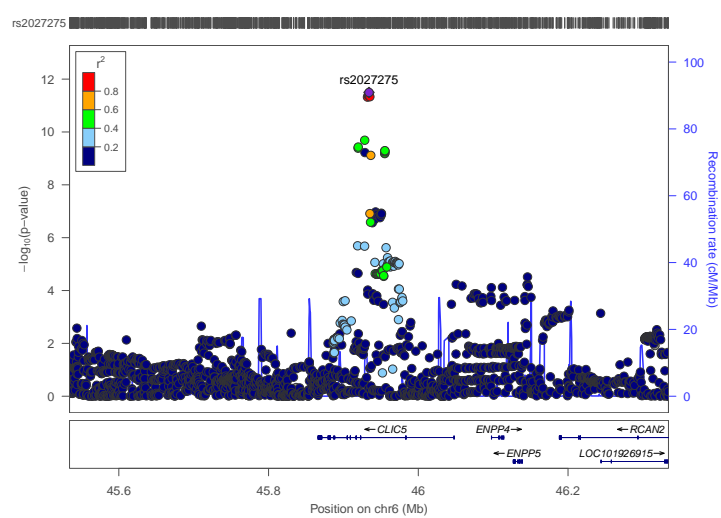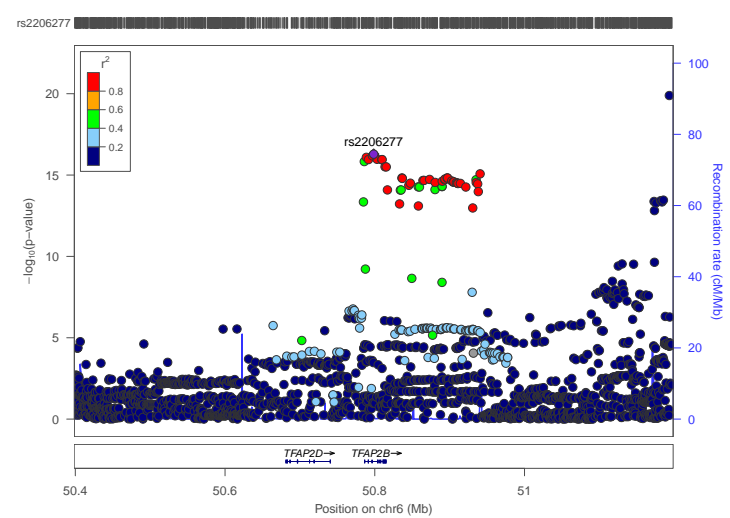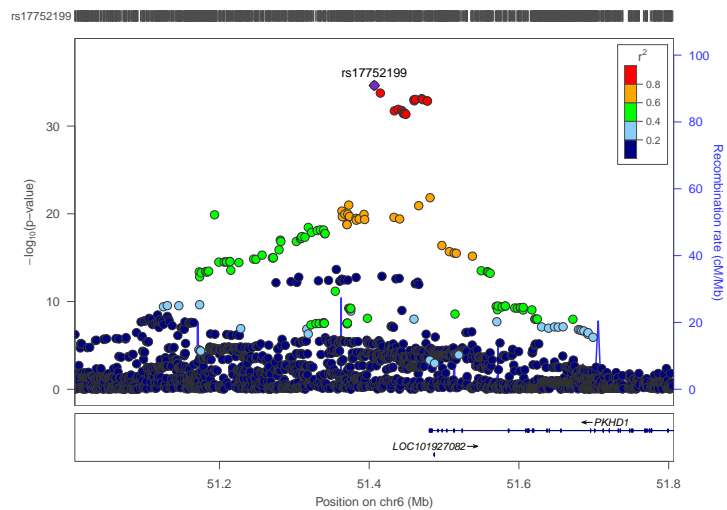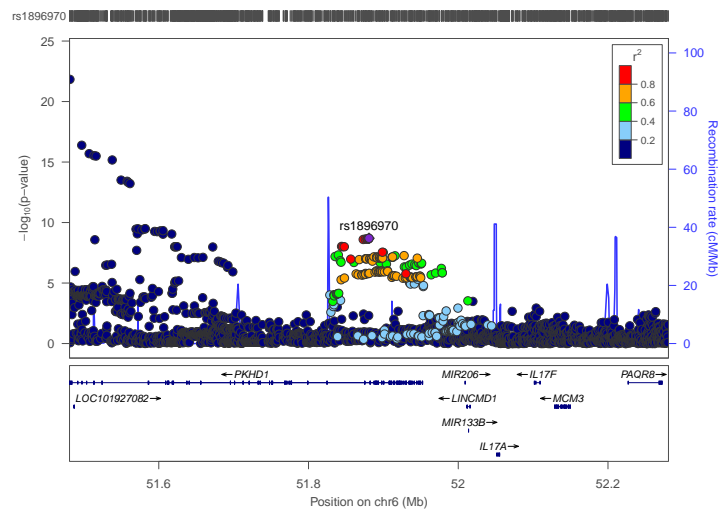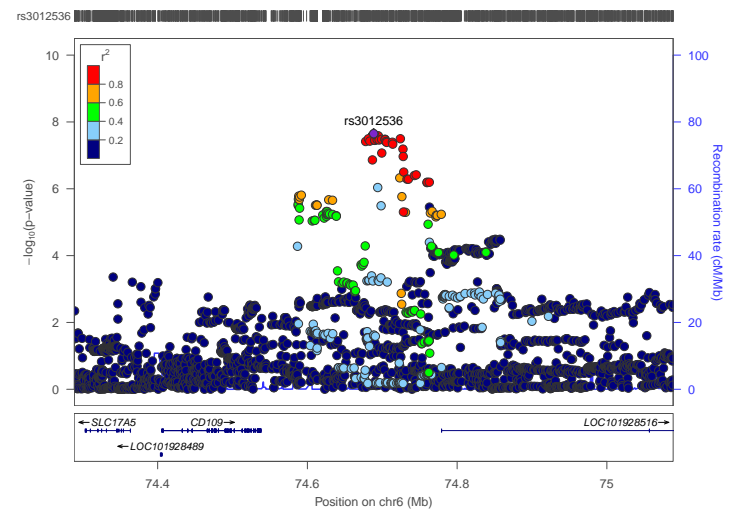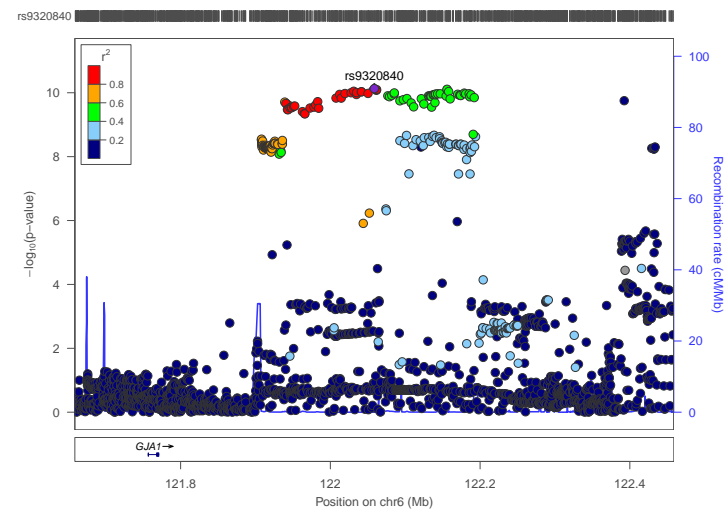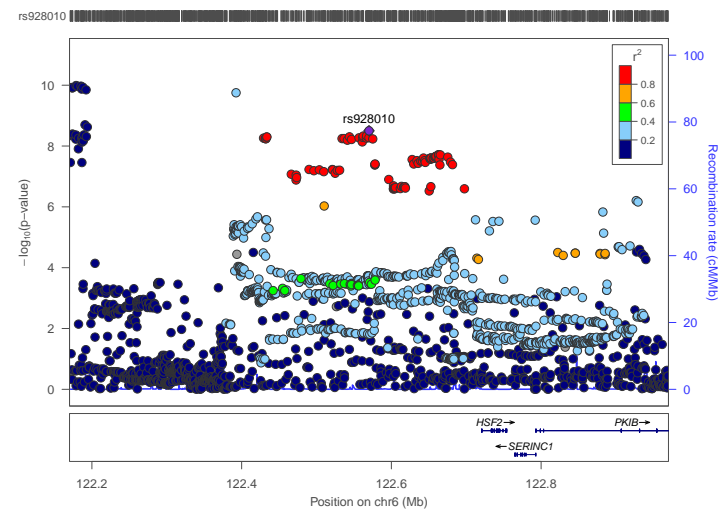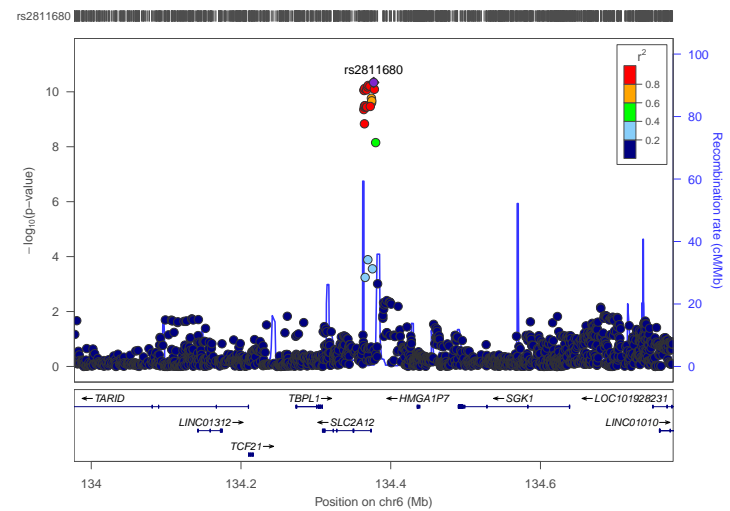

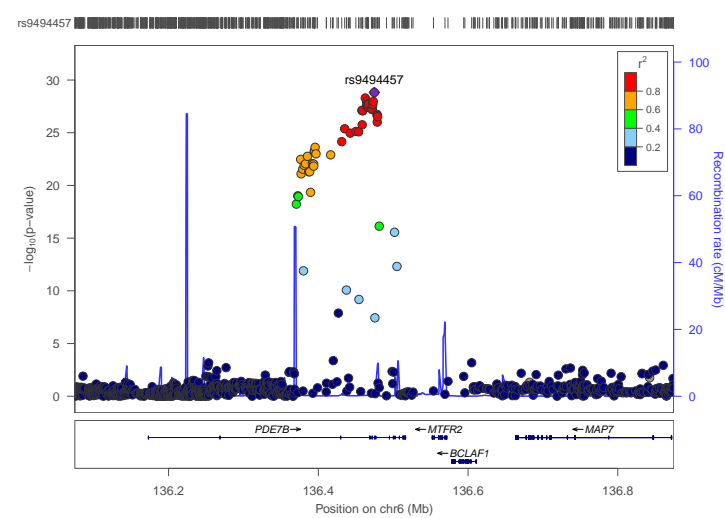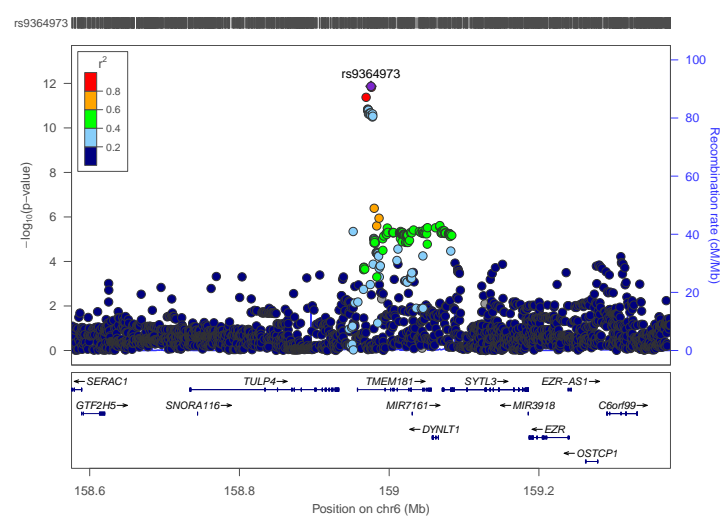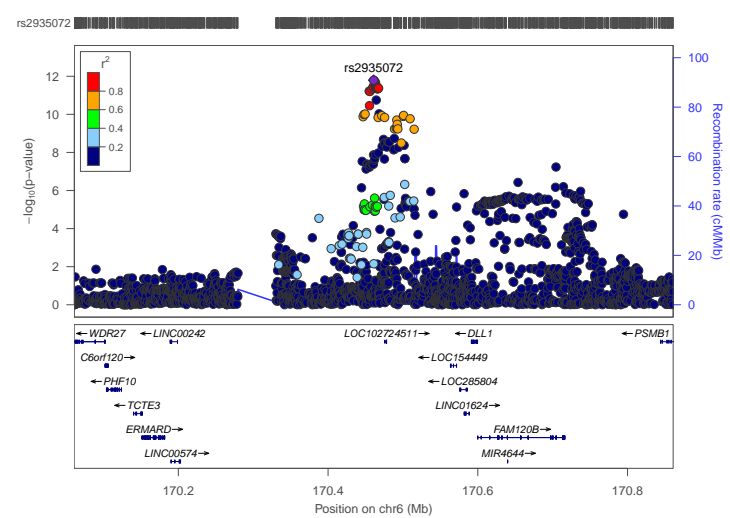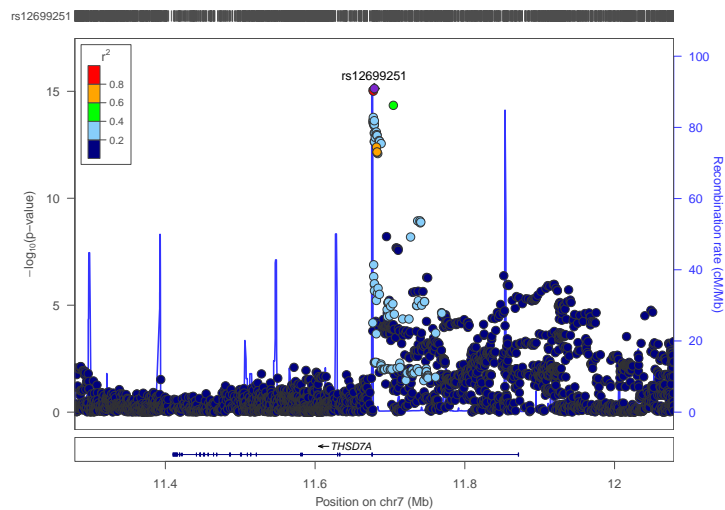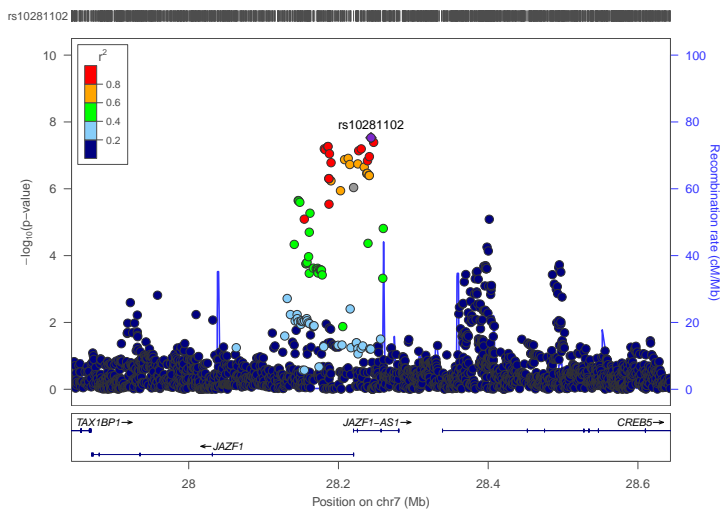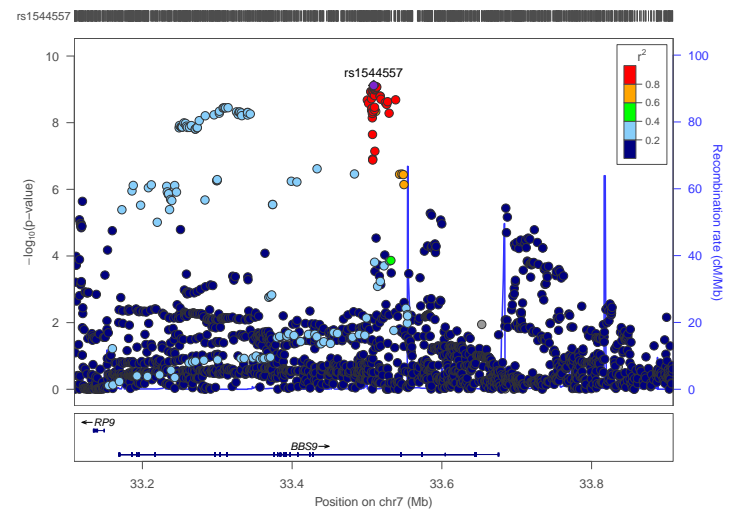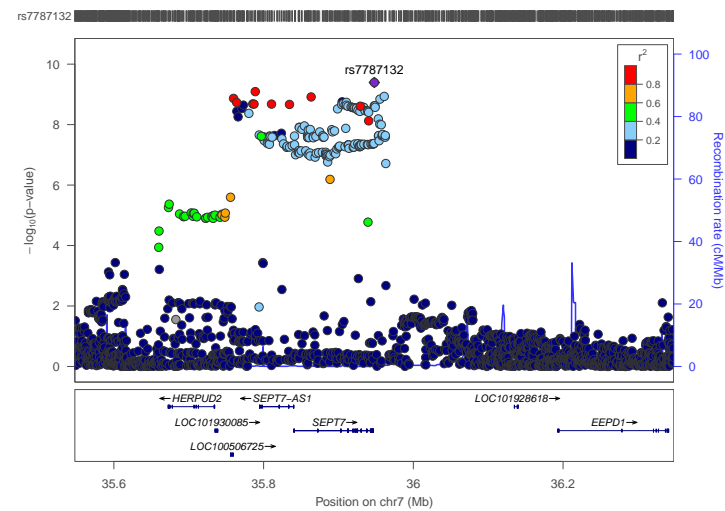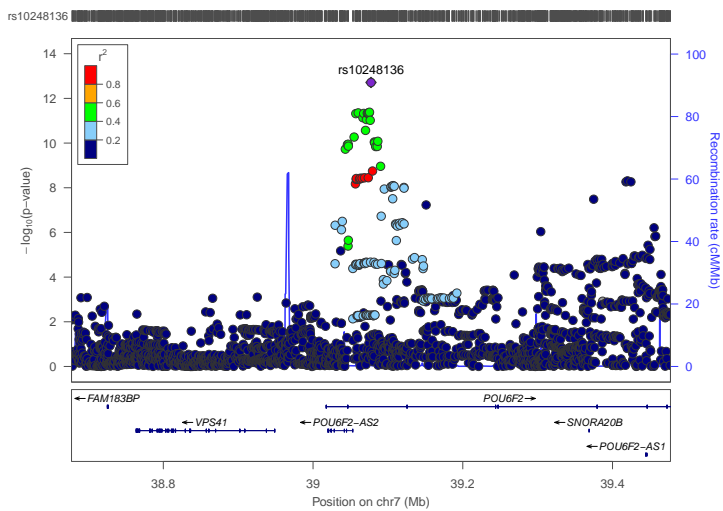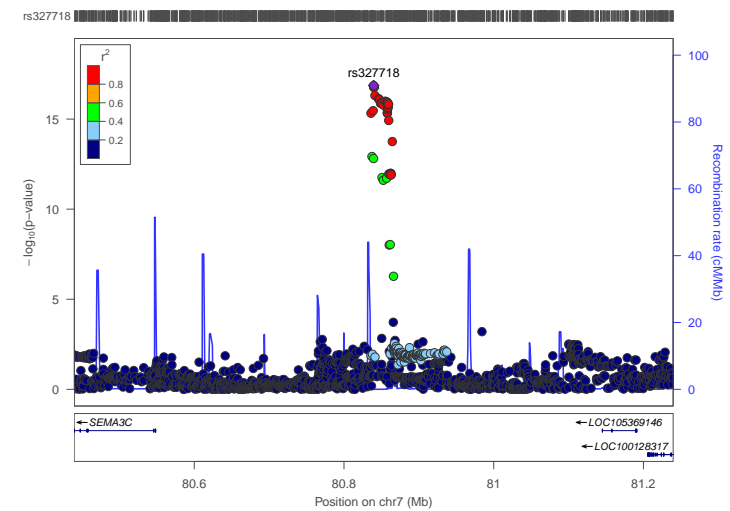

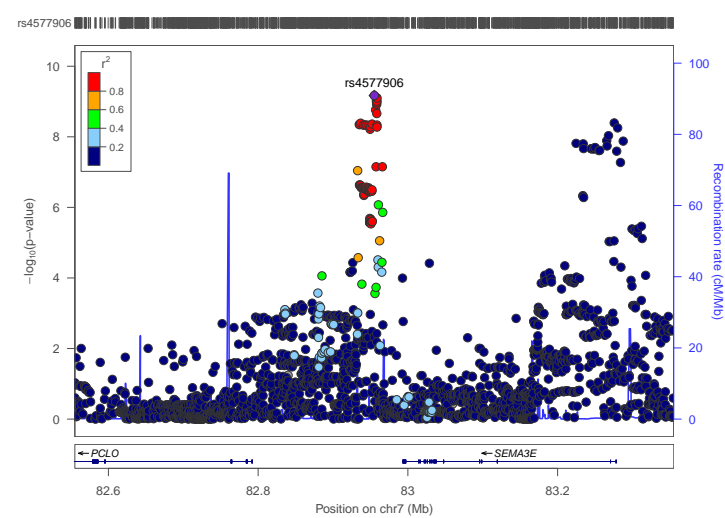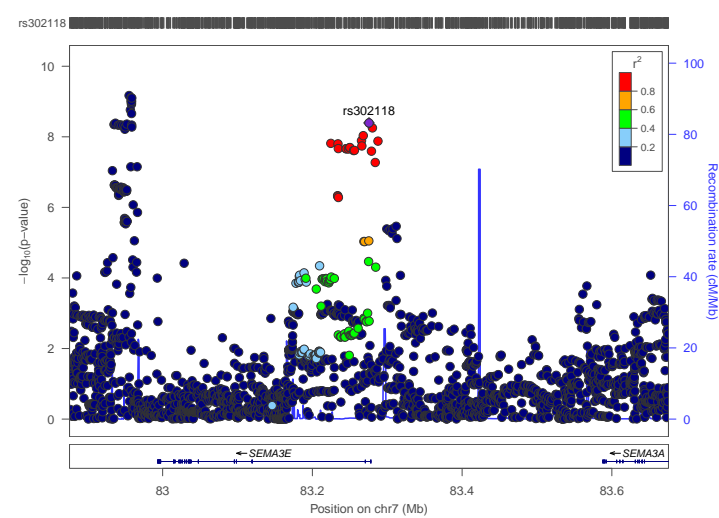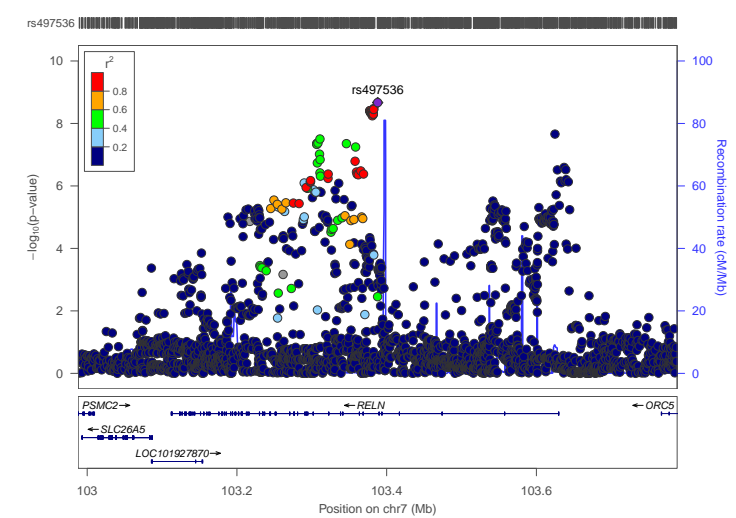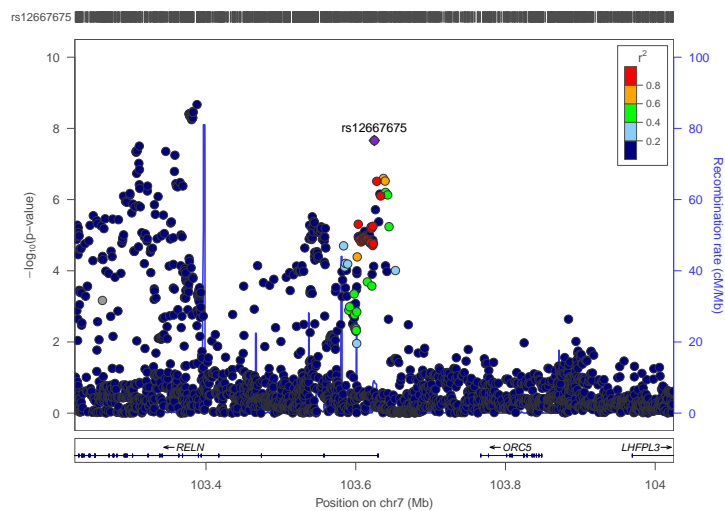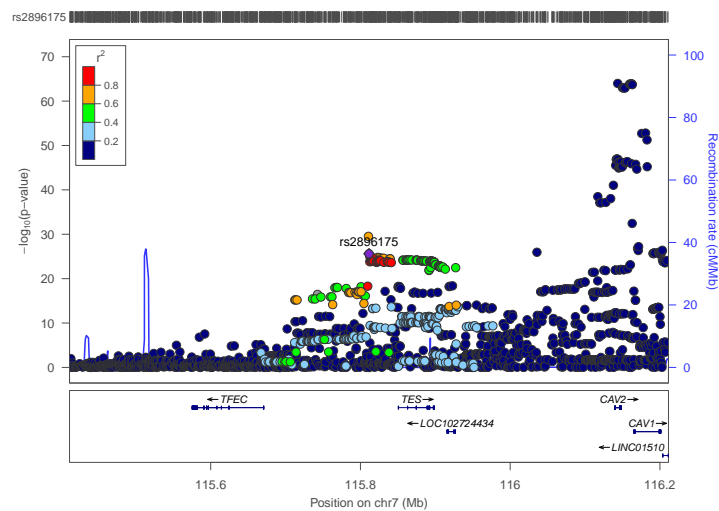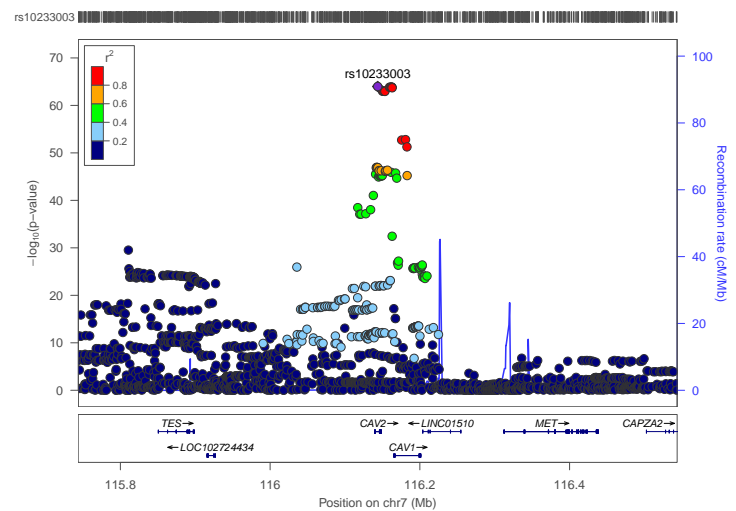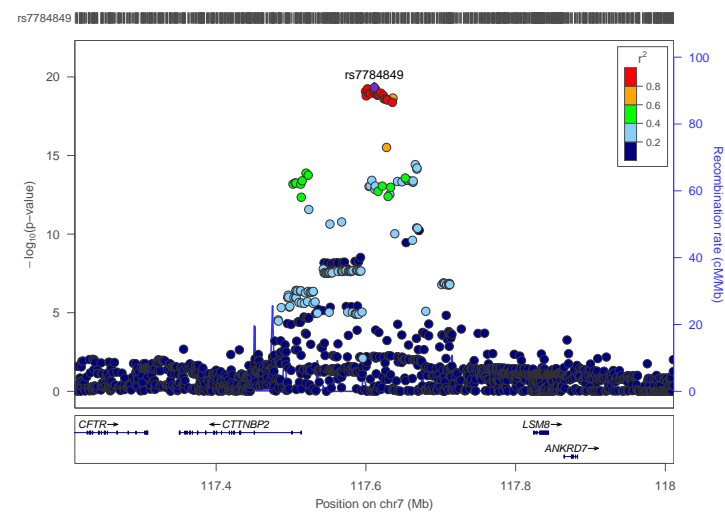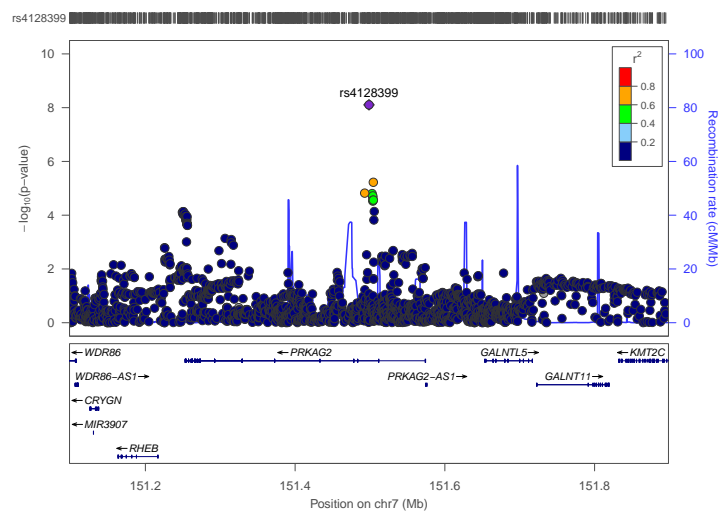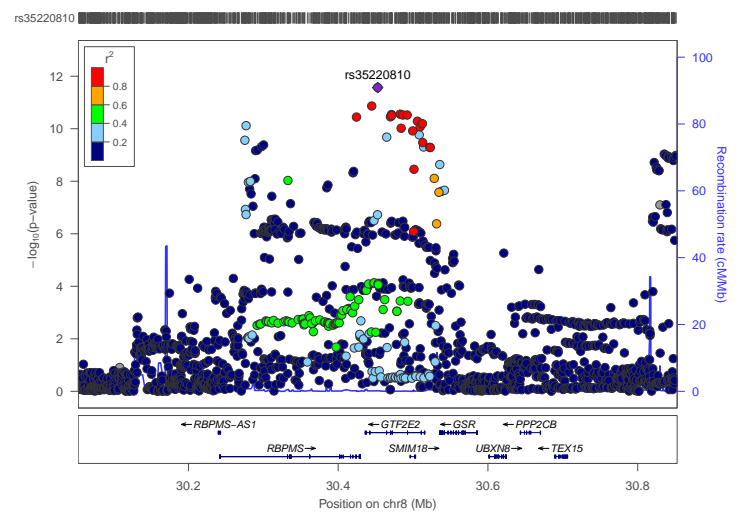

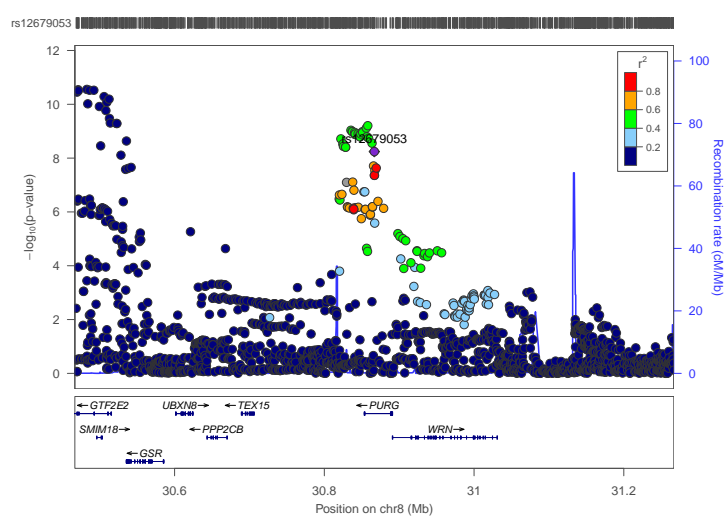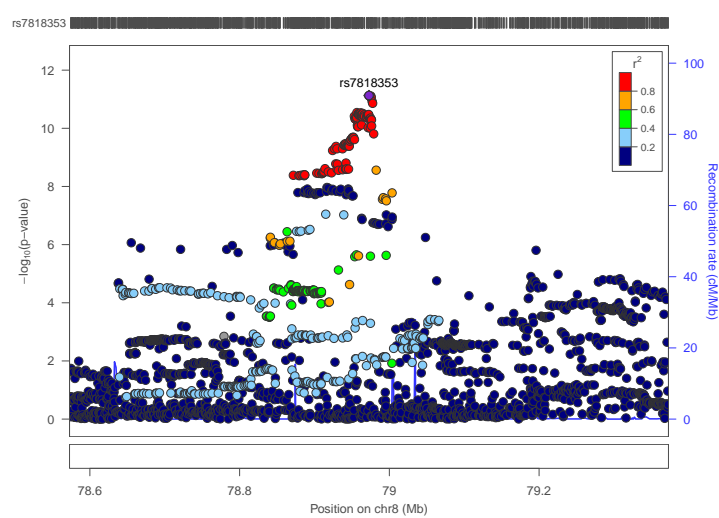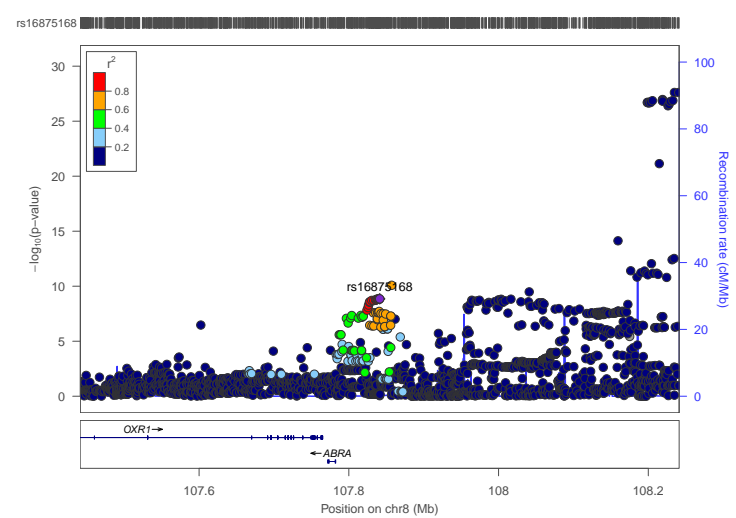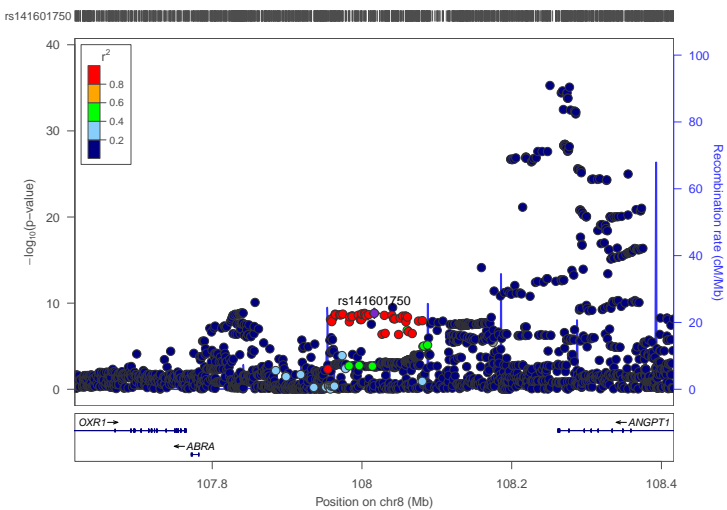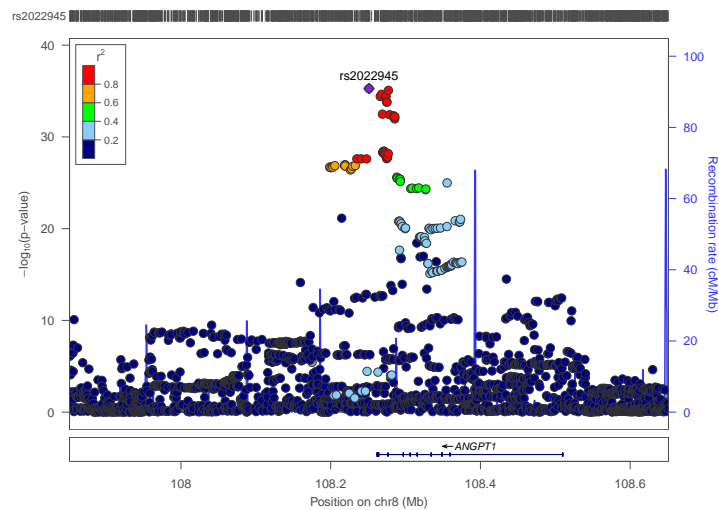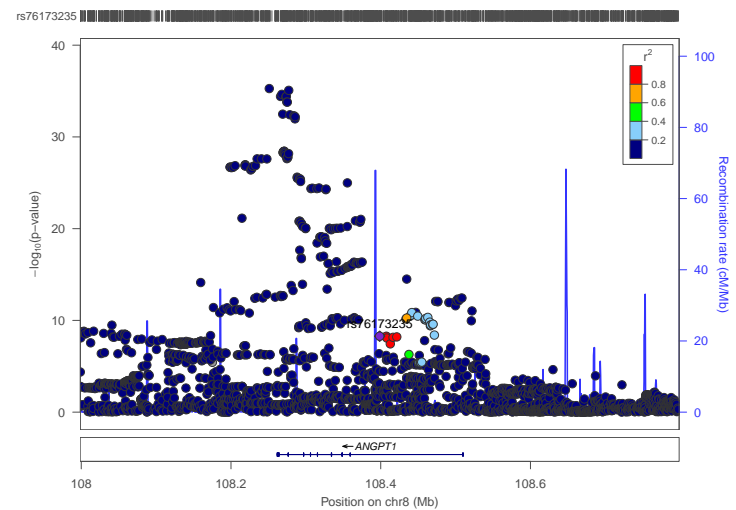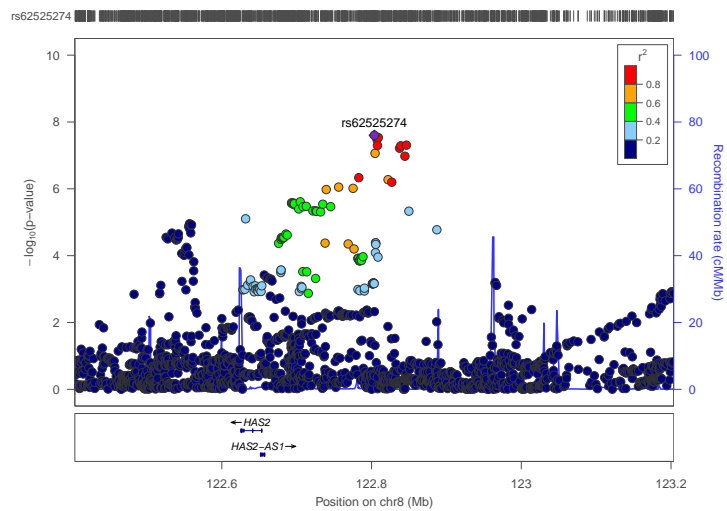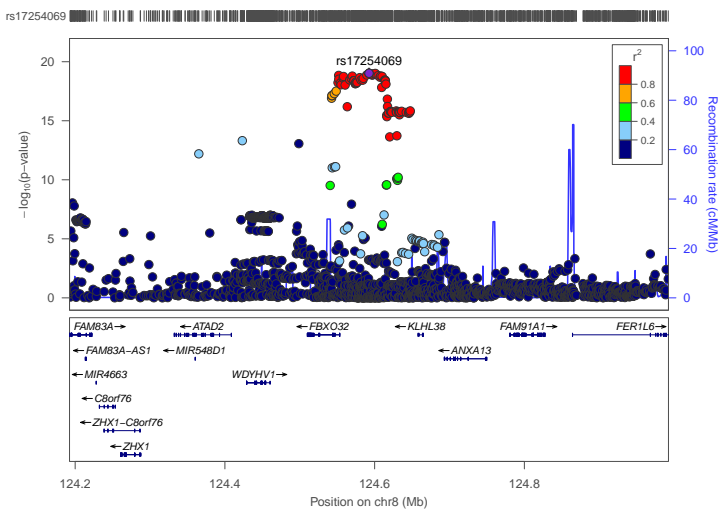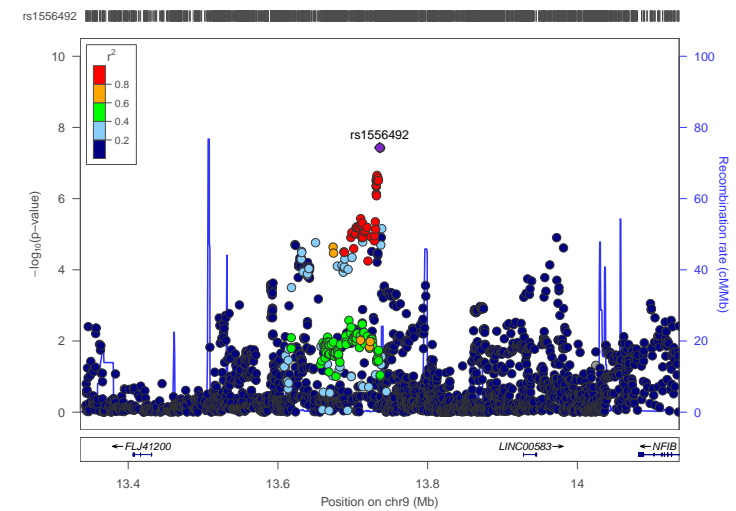

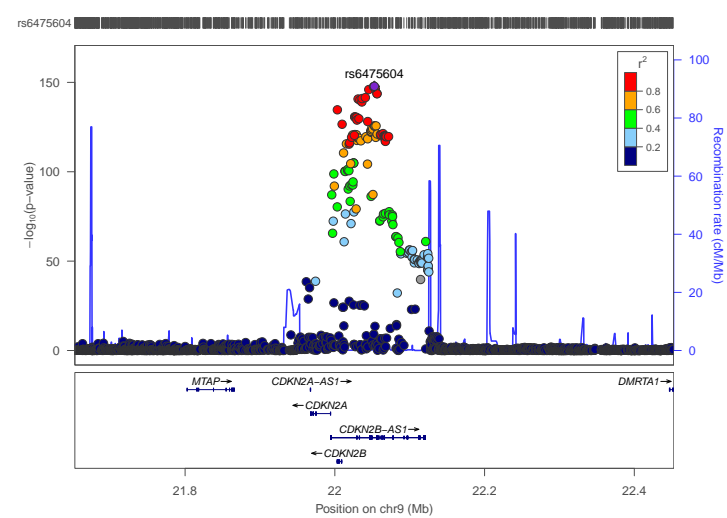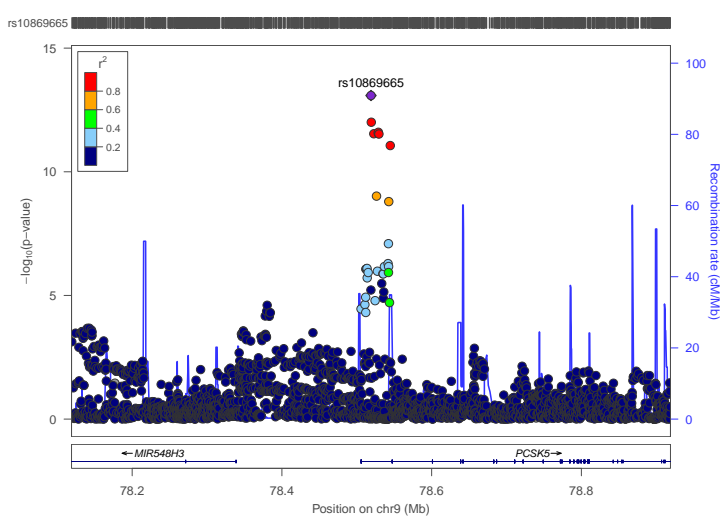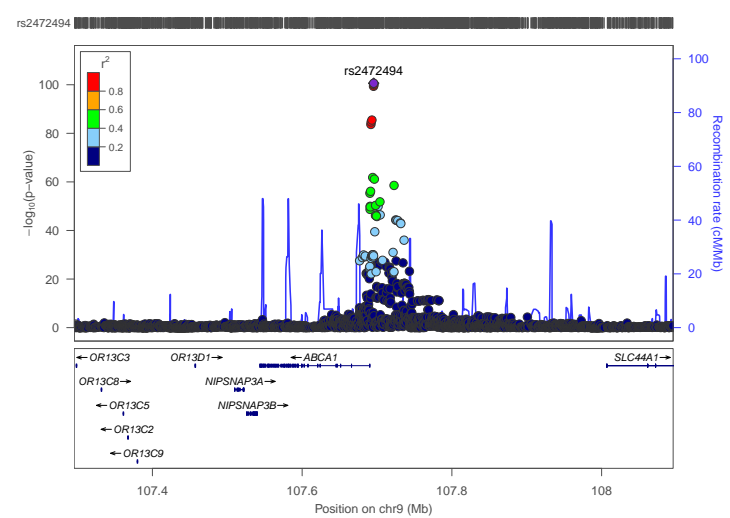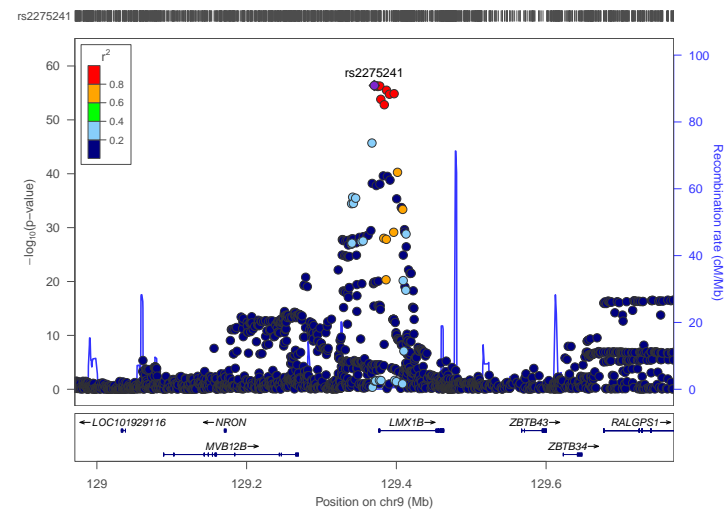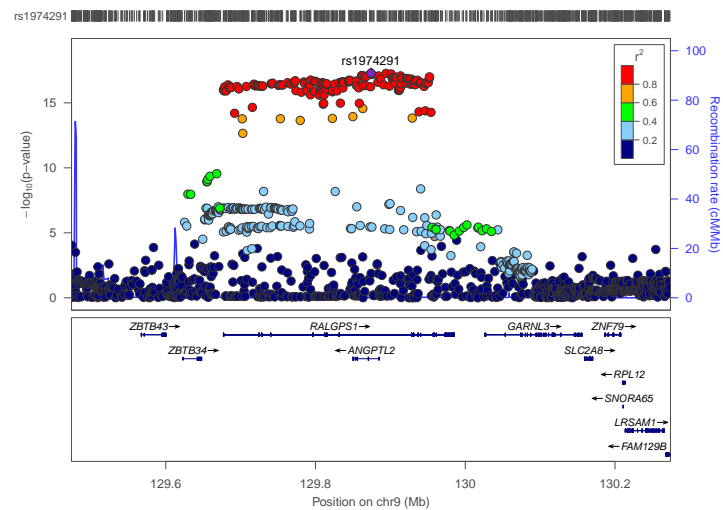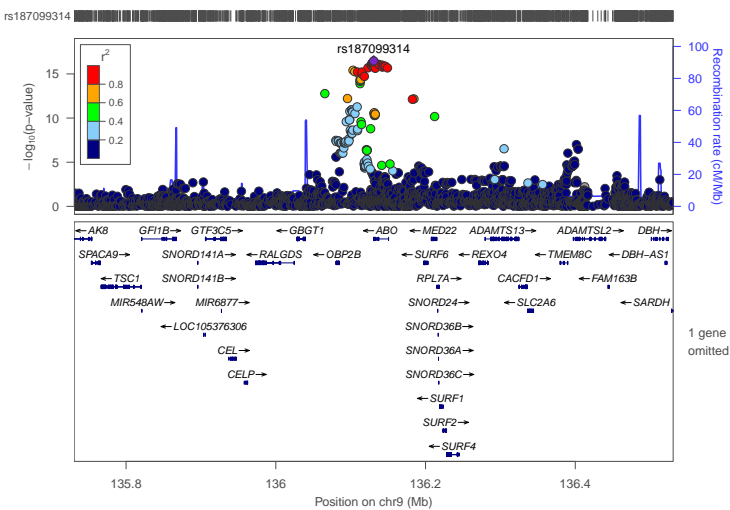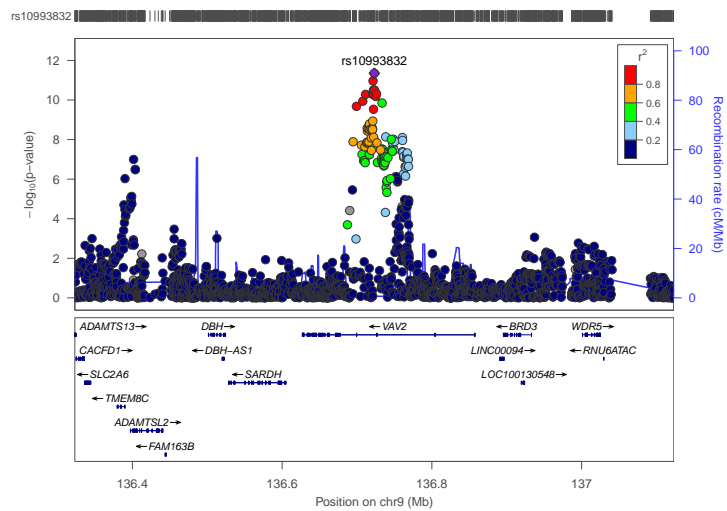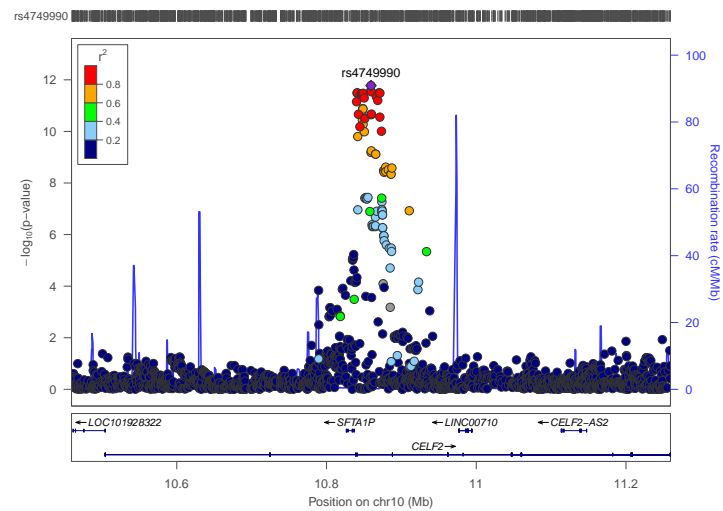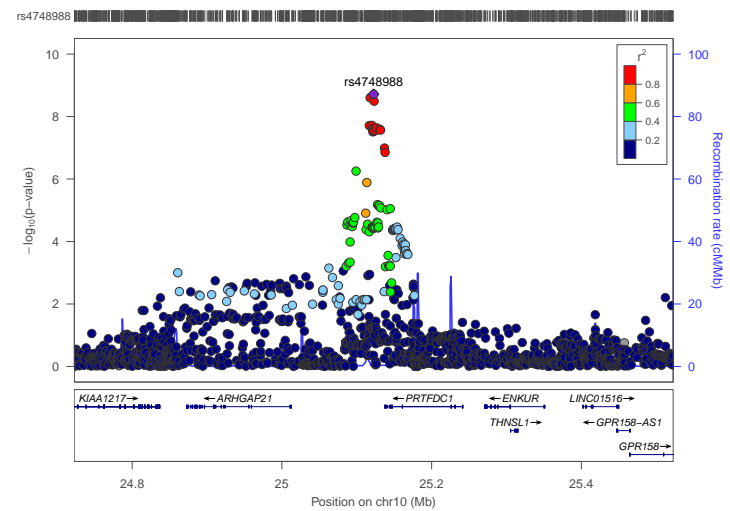

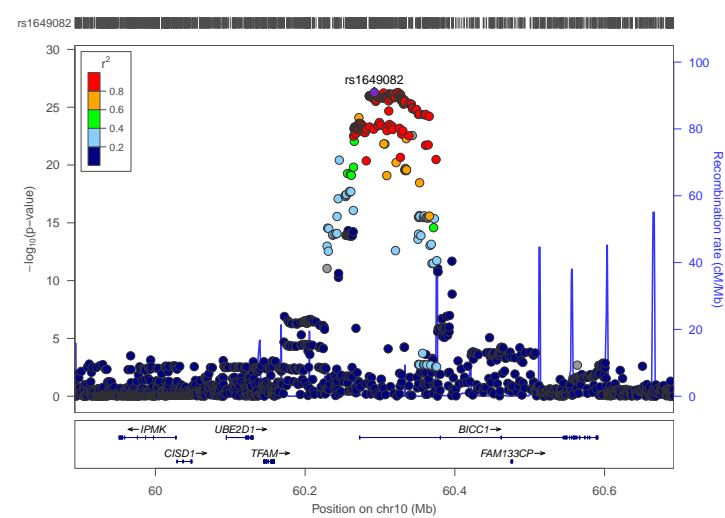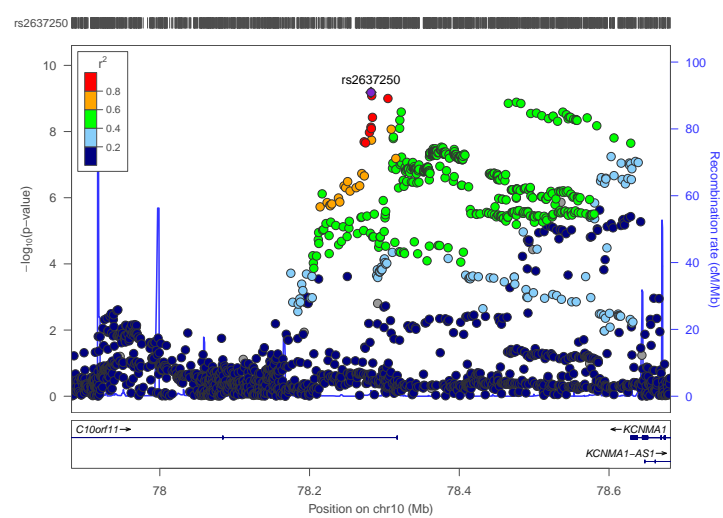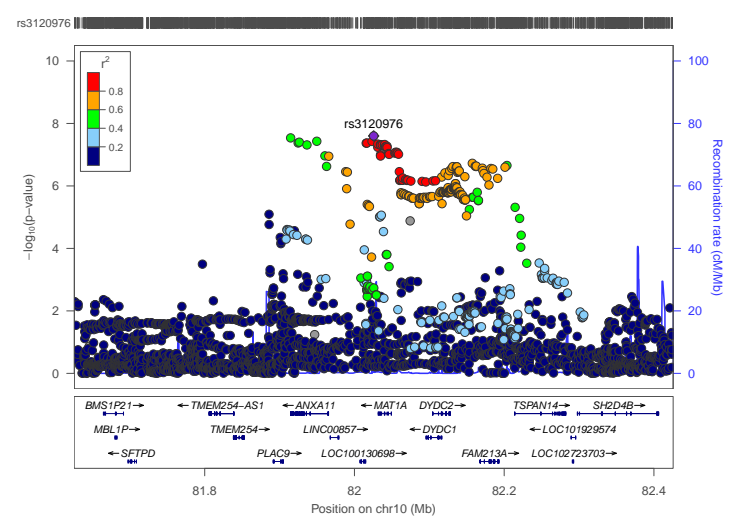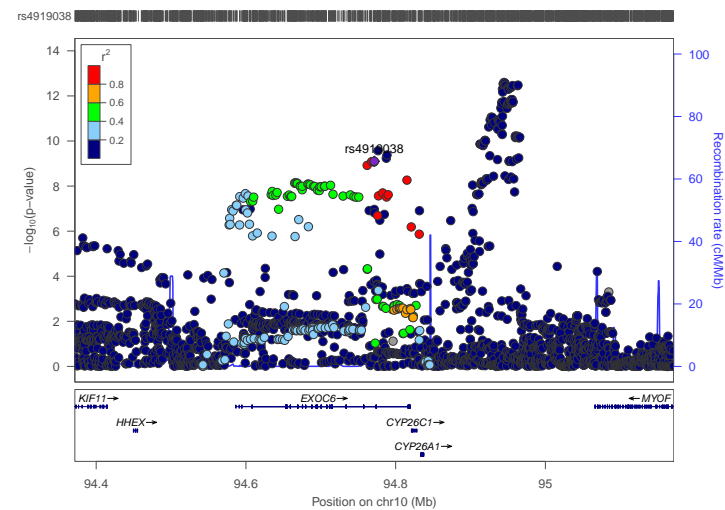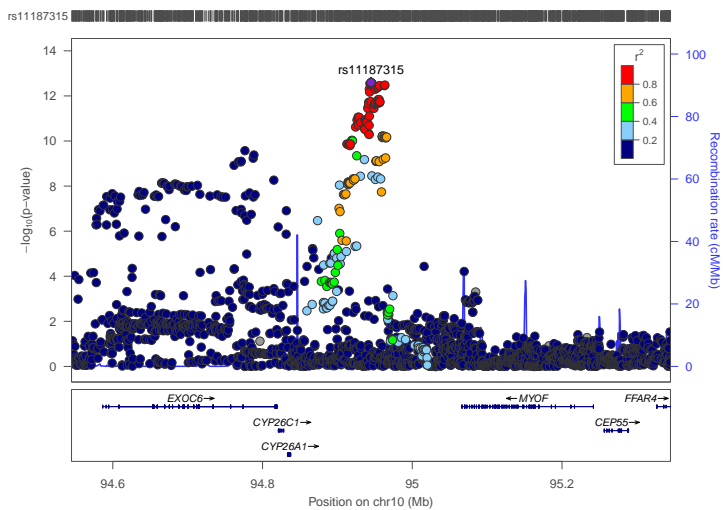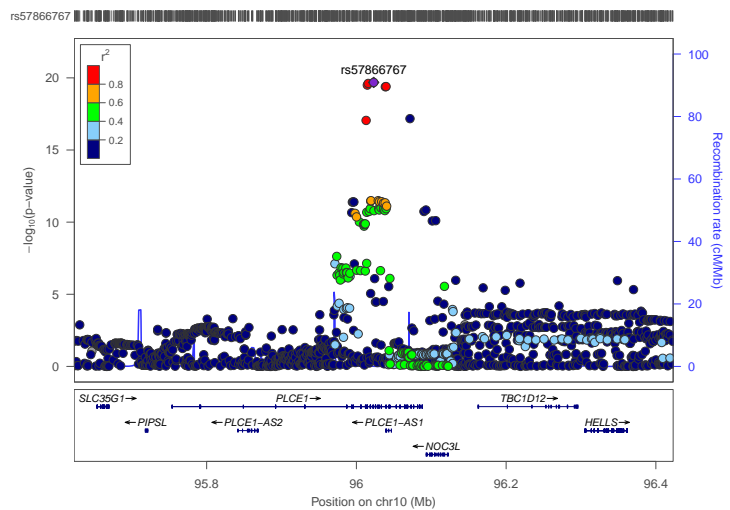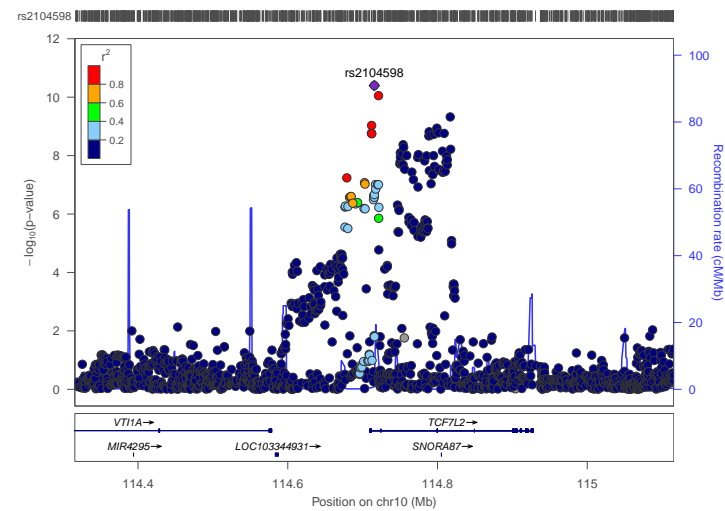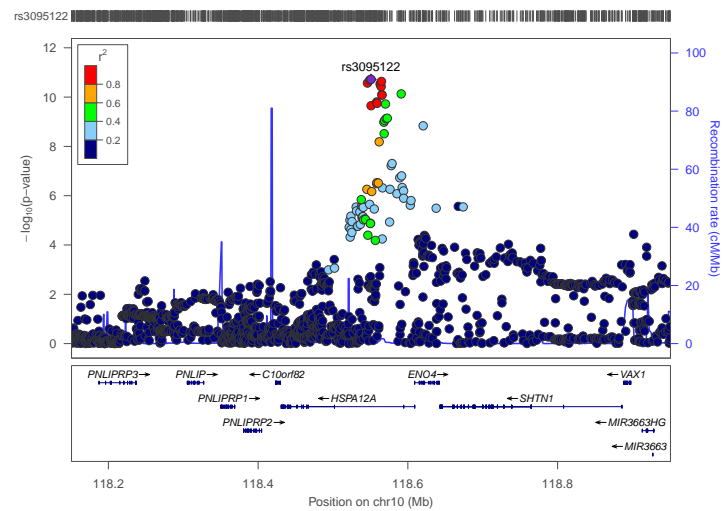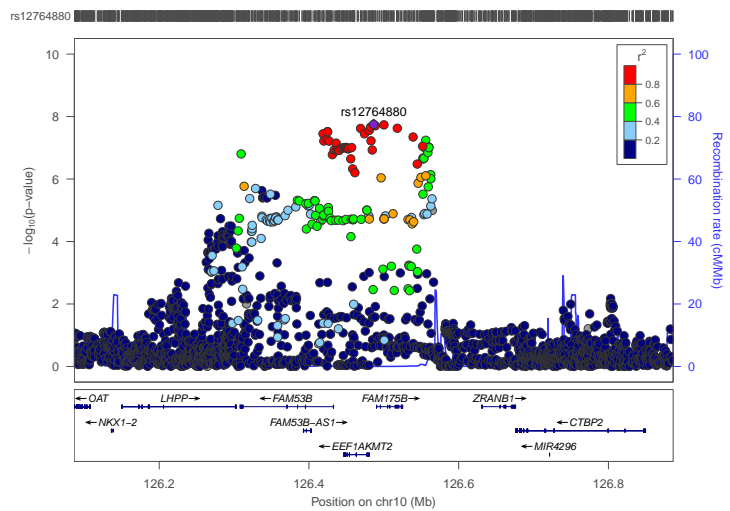

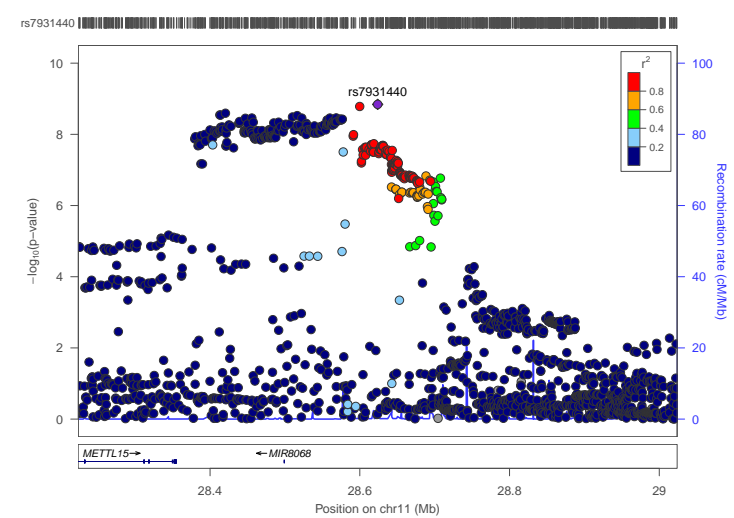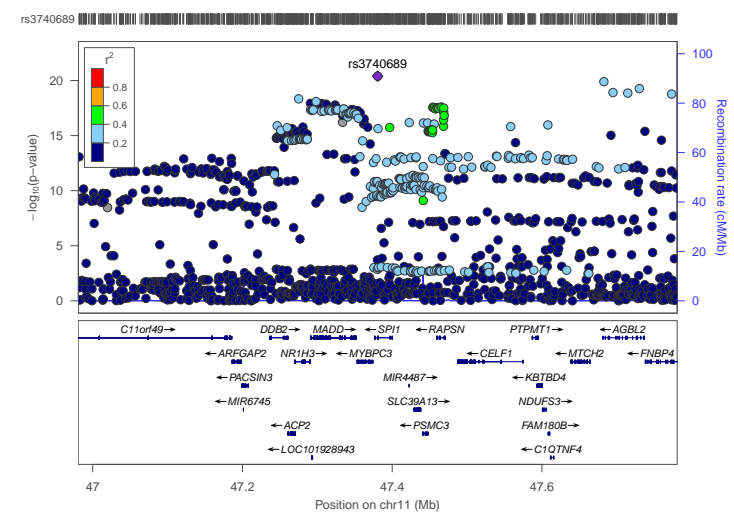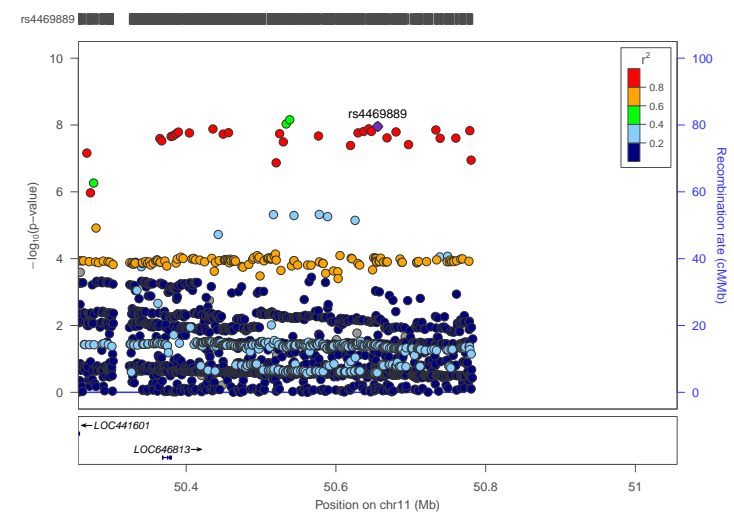

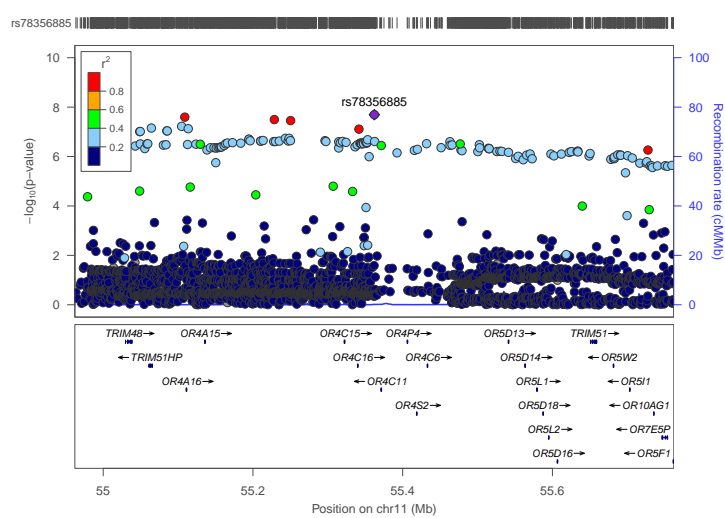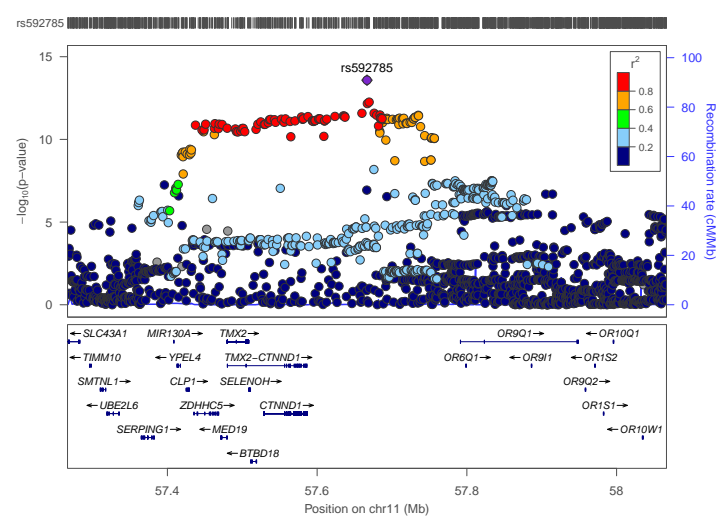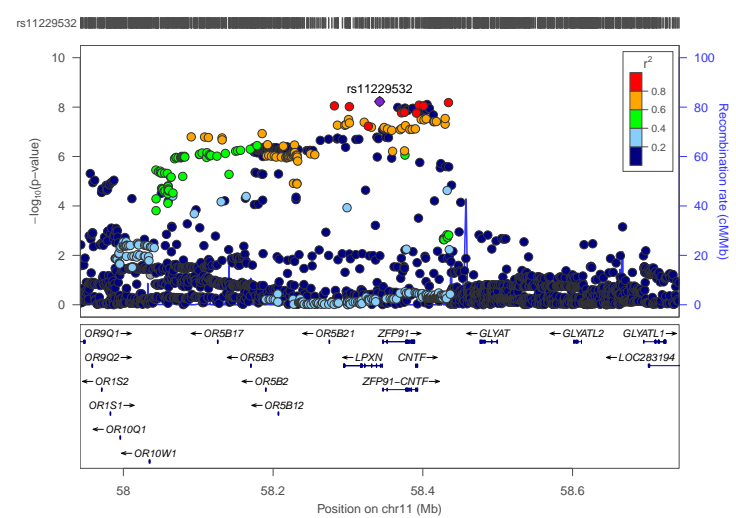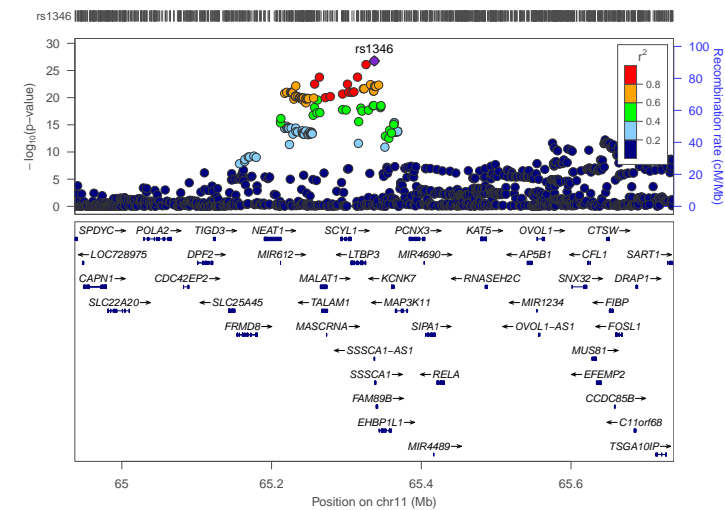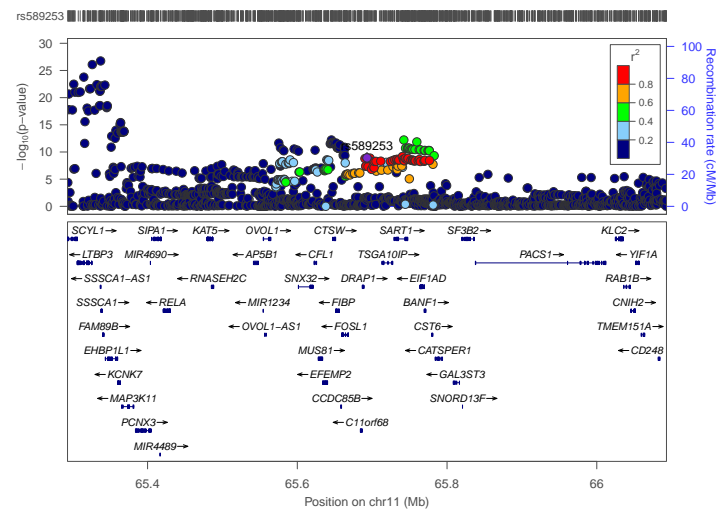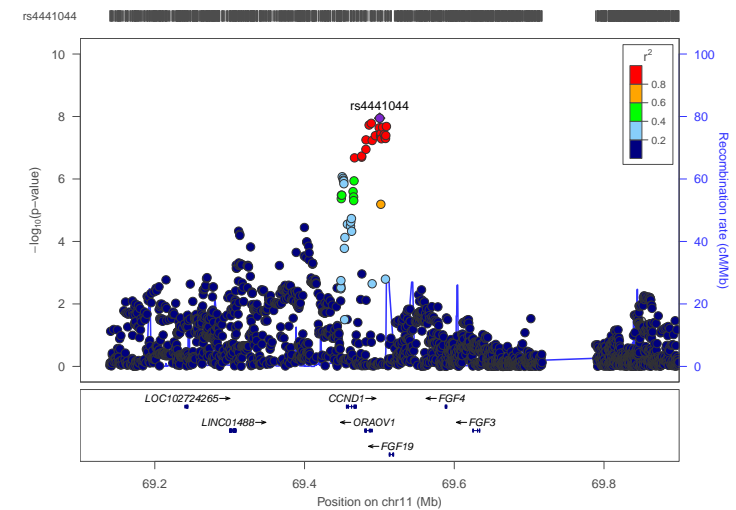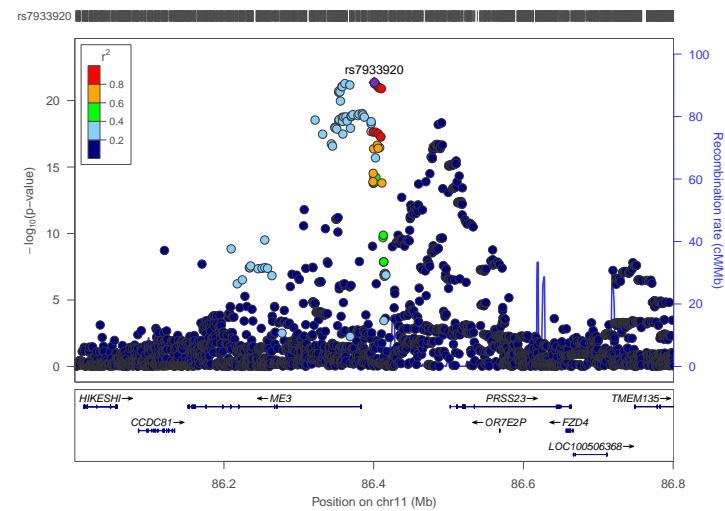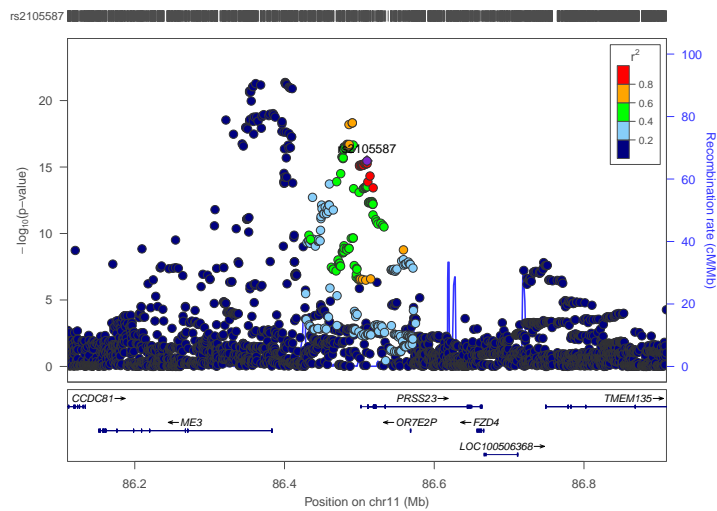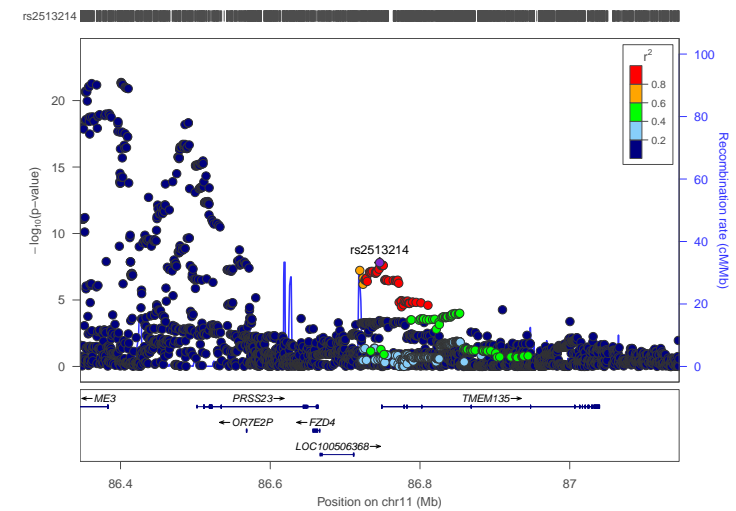

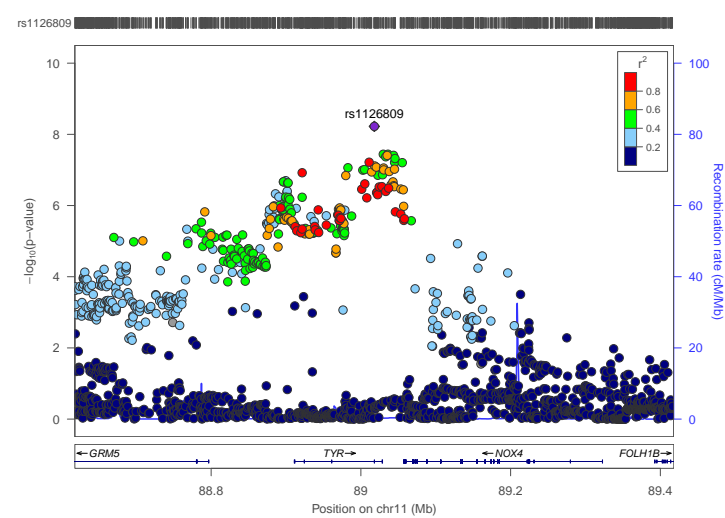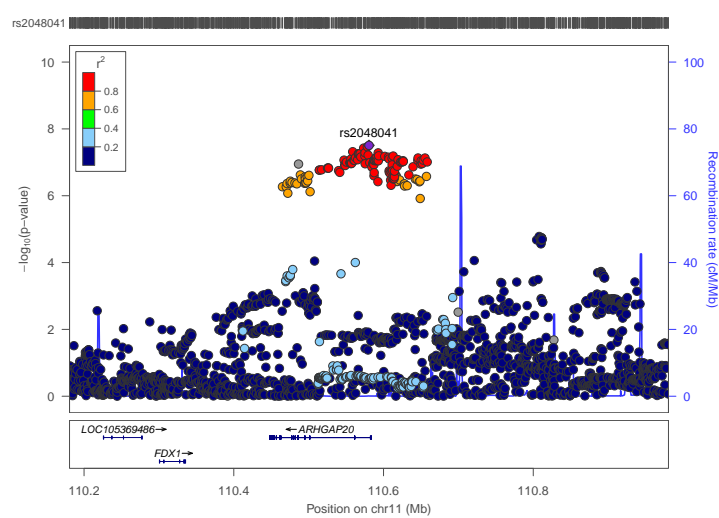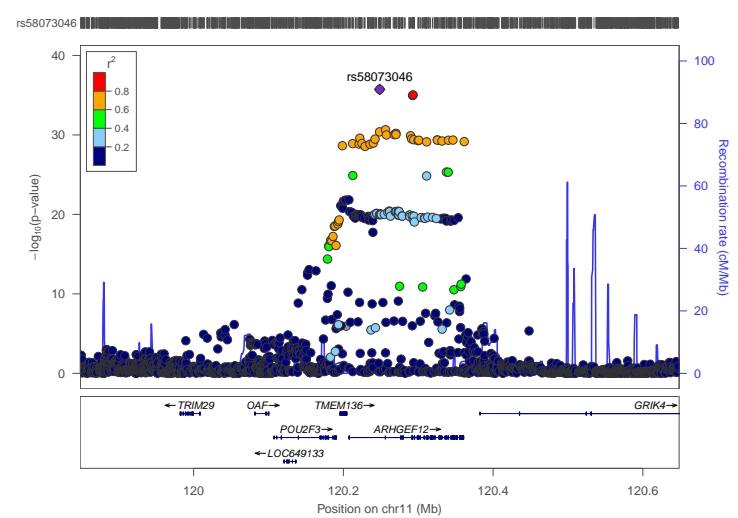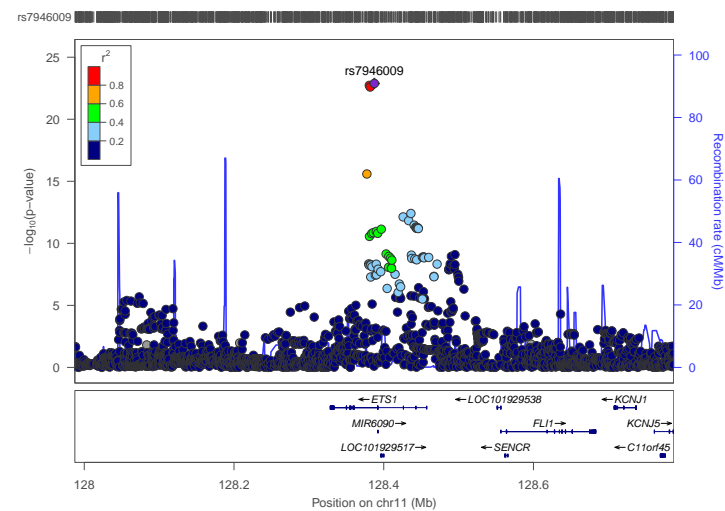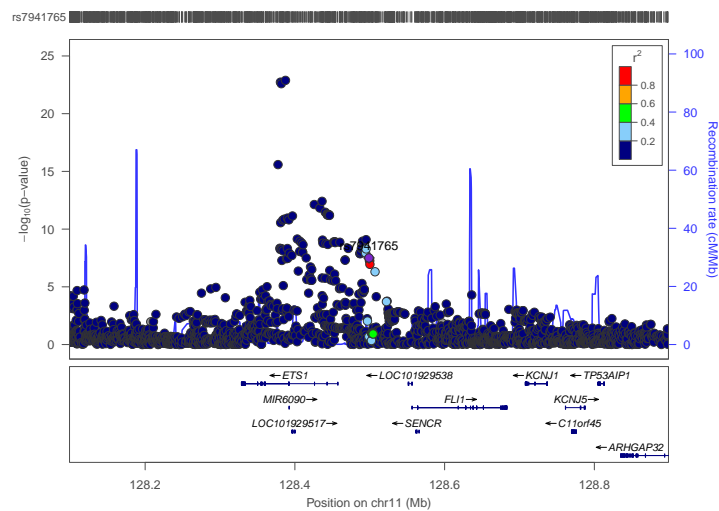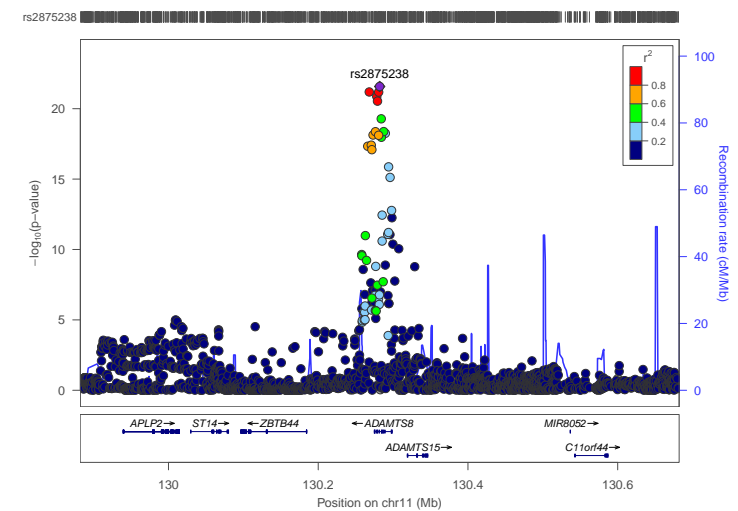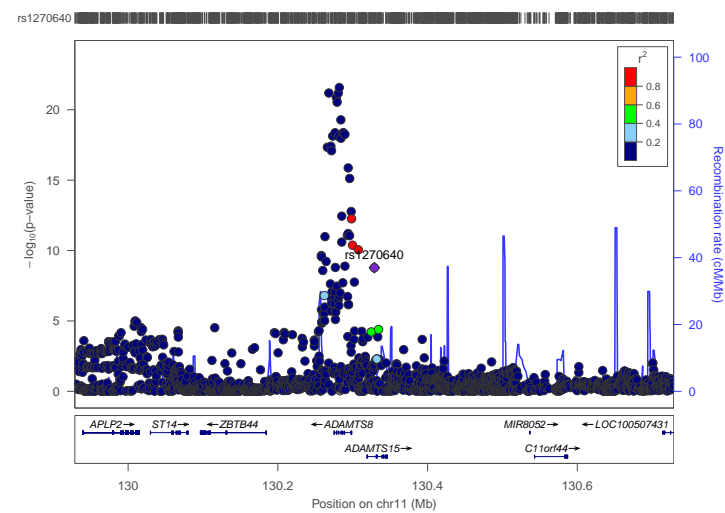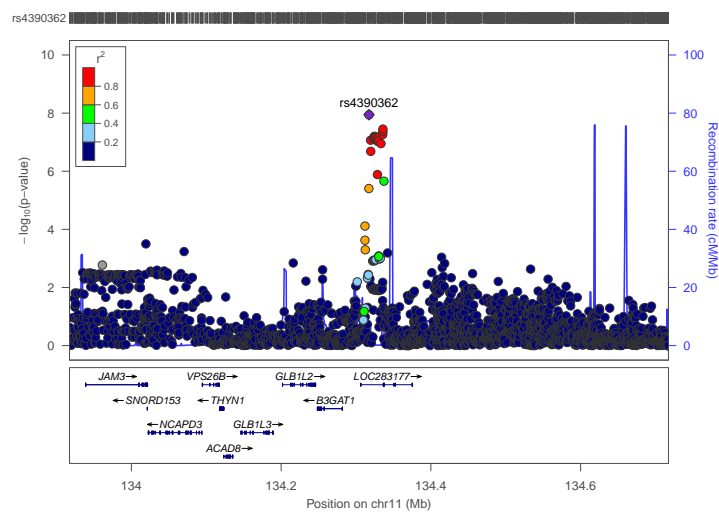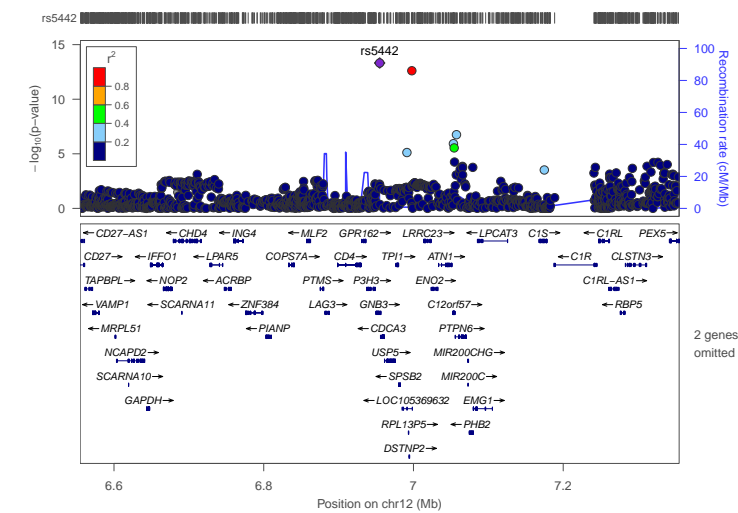

2 genes omitted

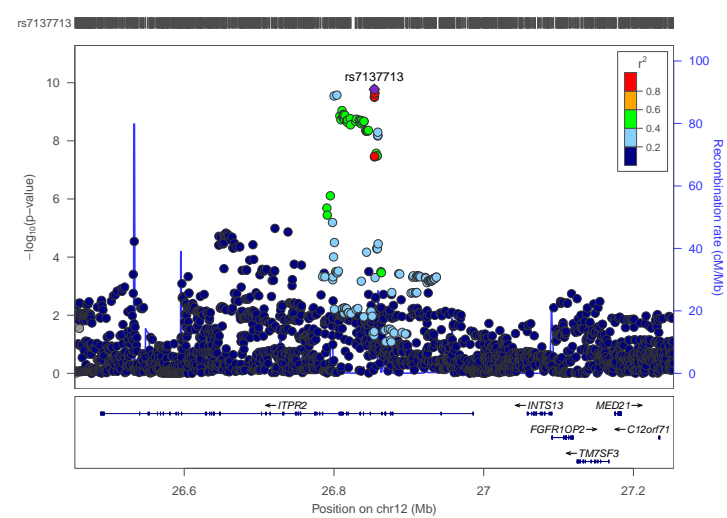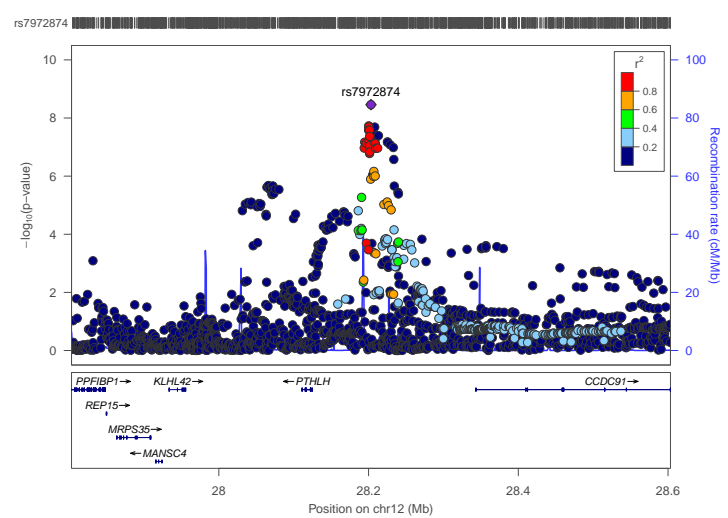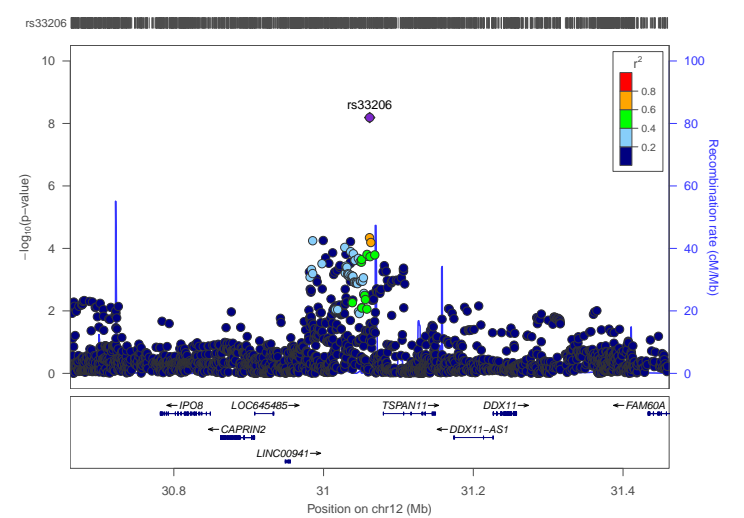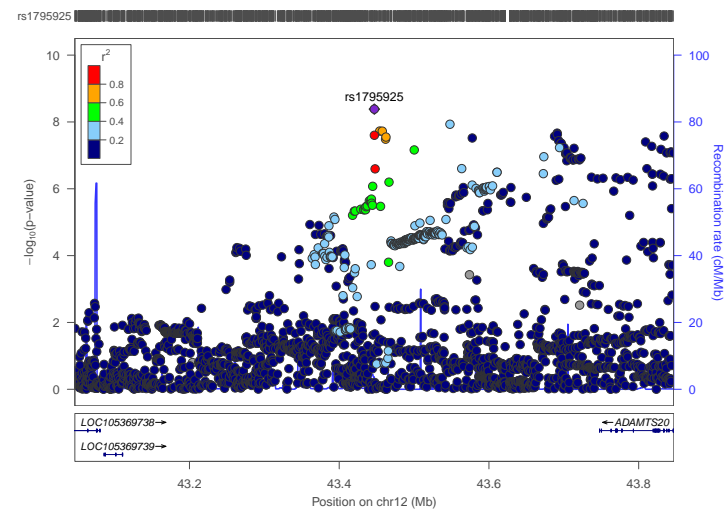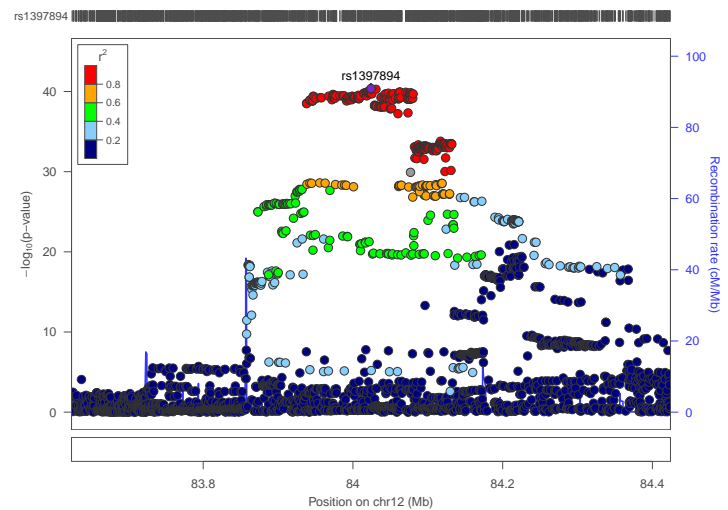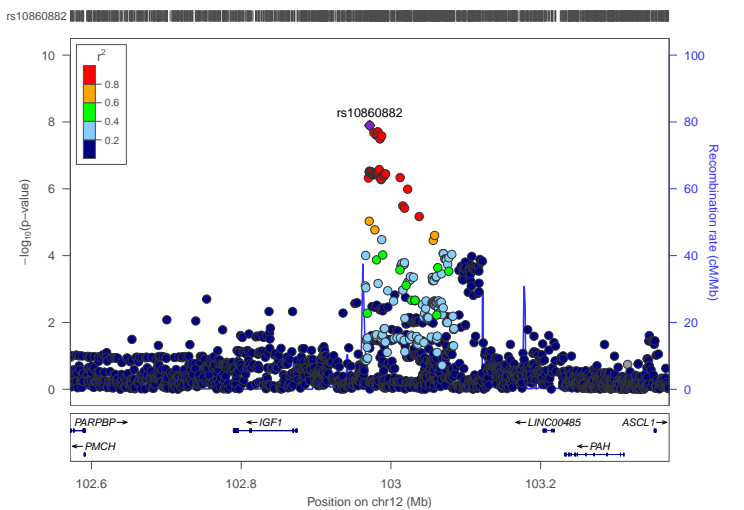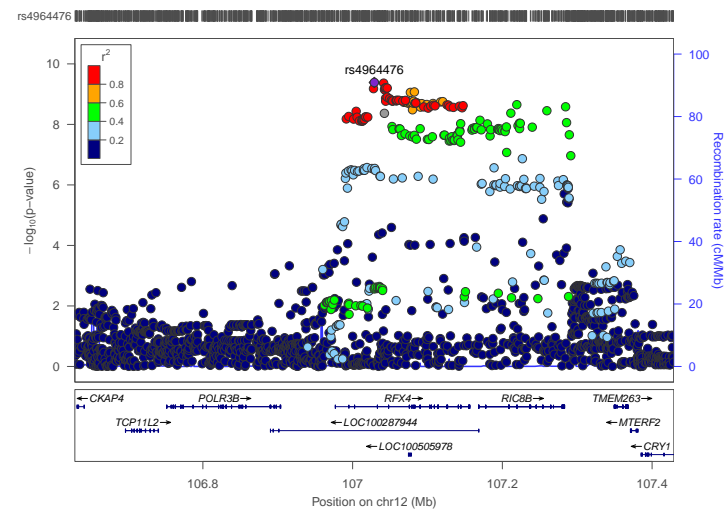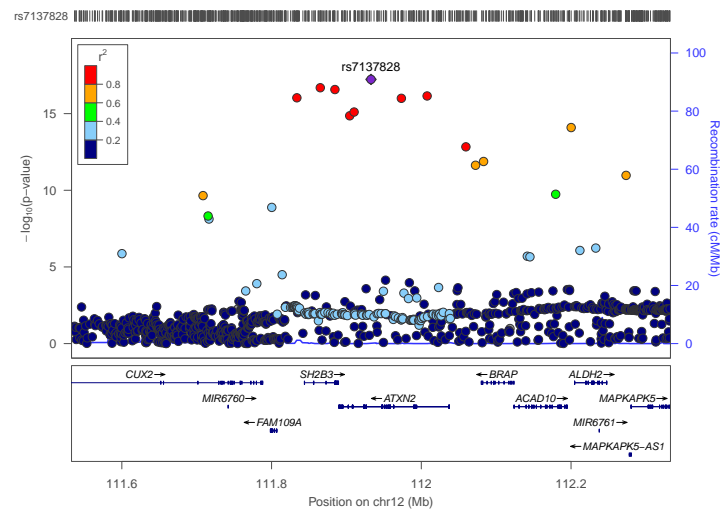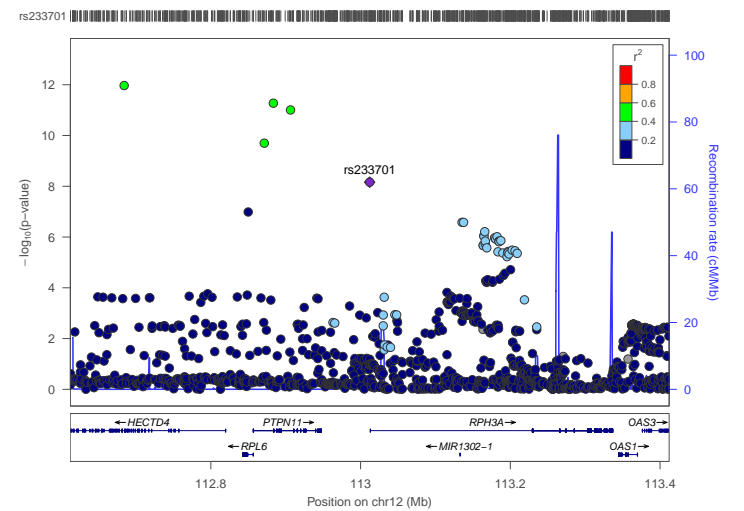

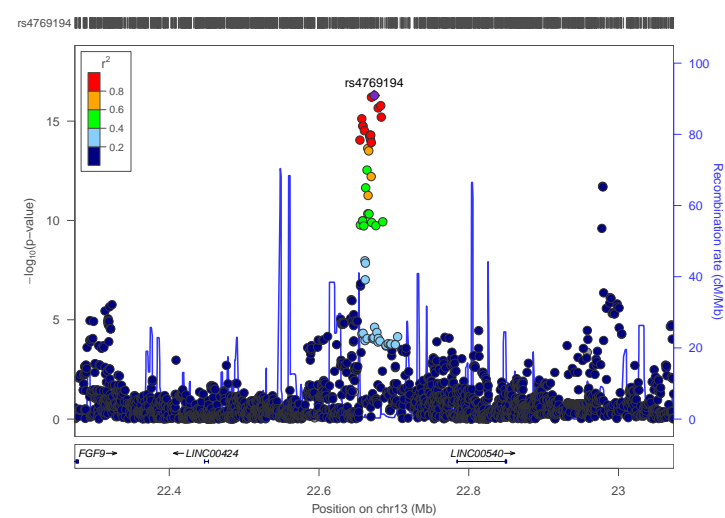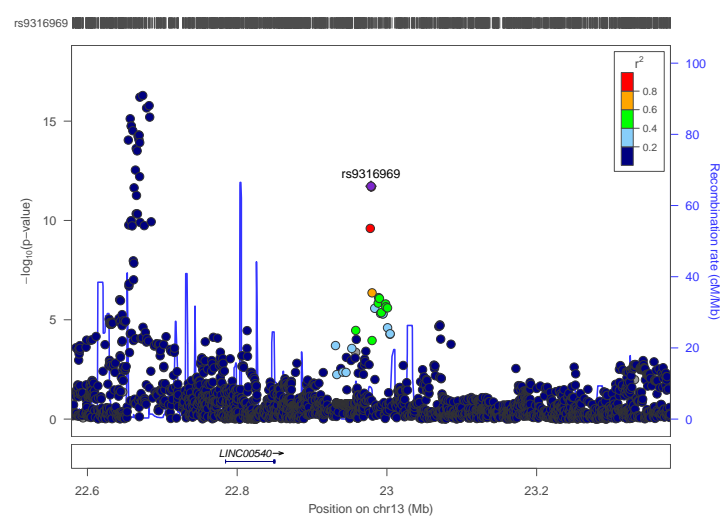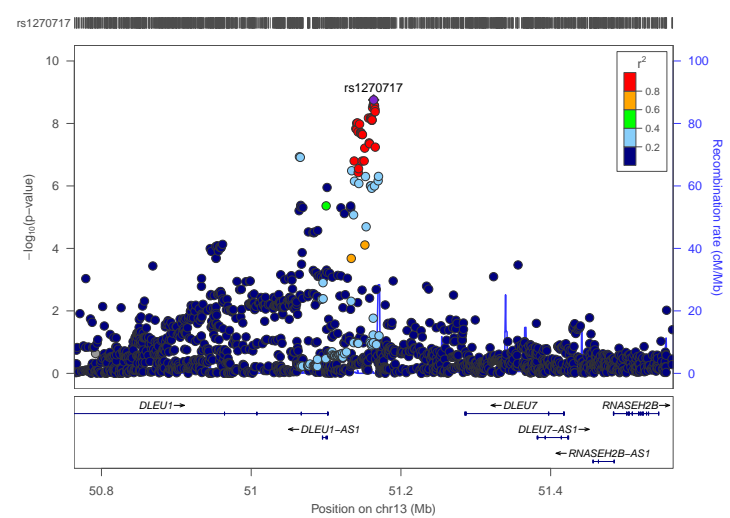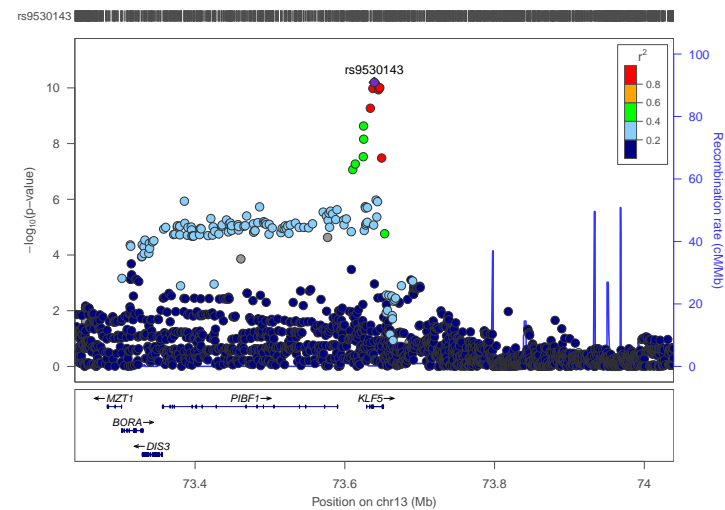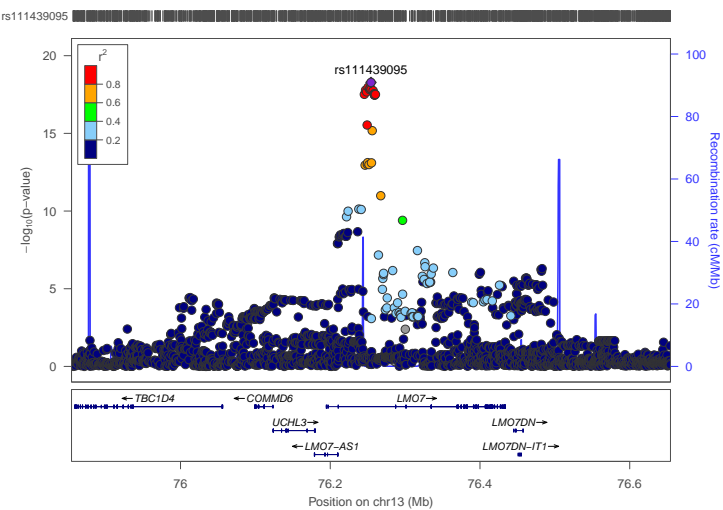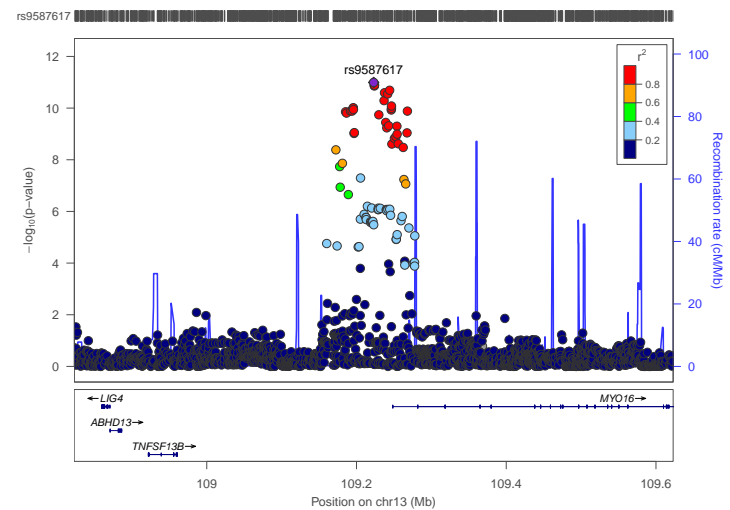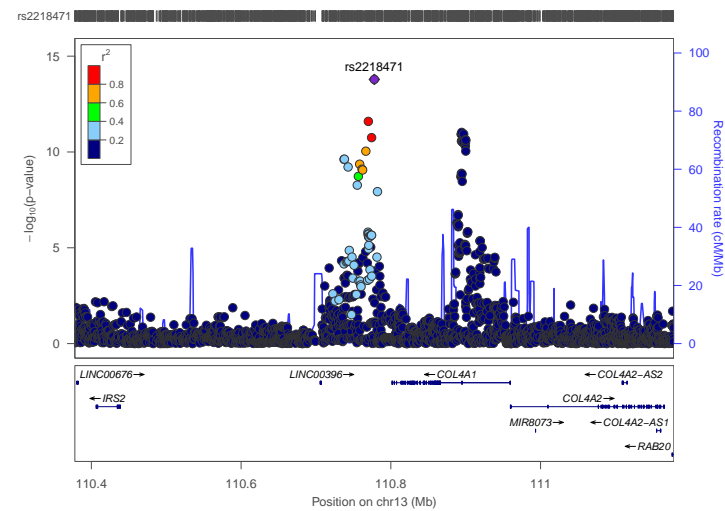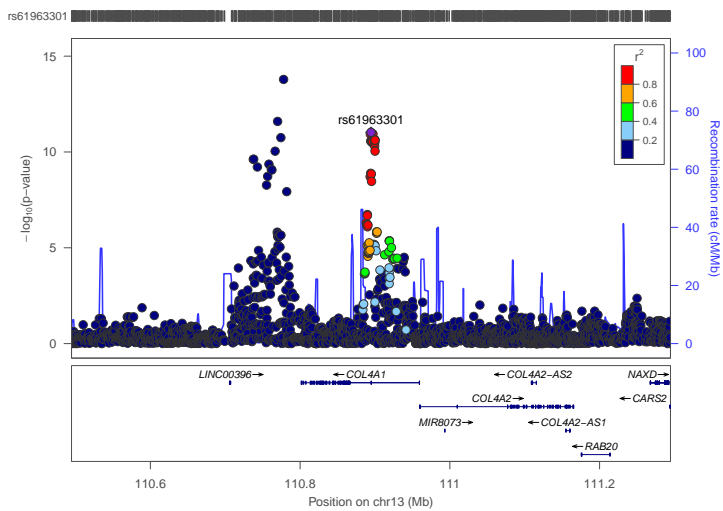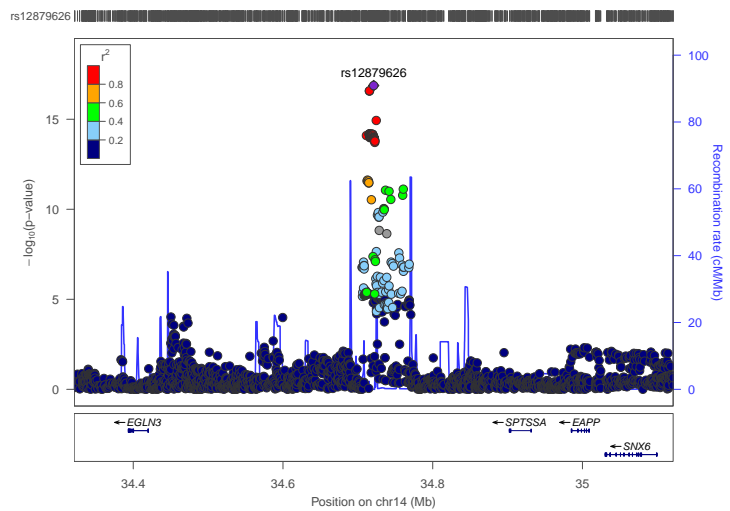

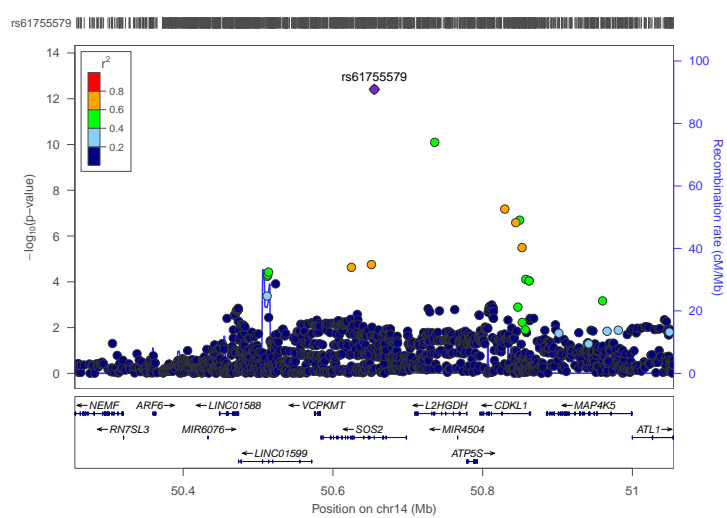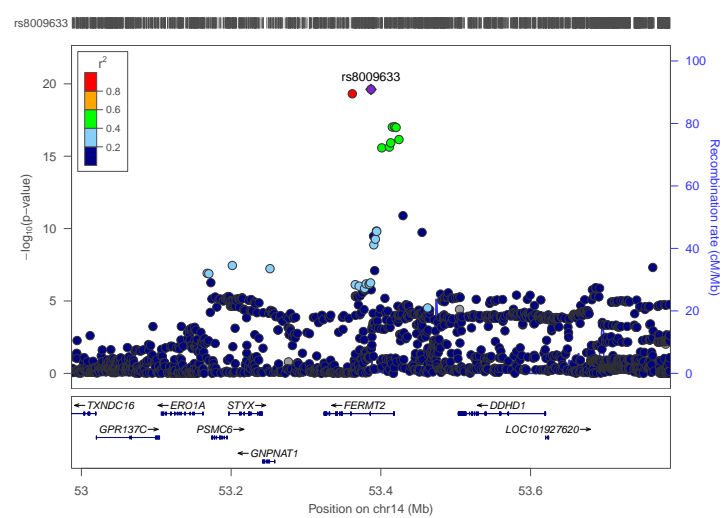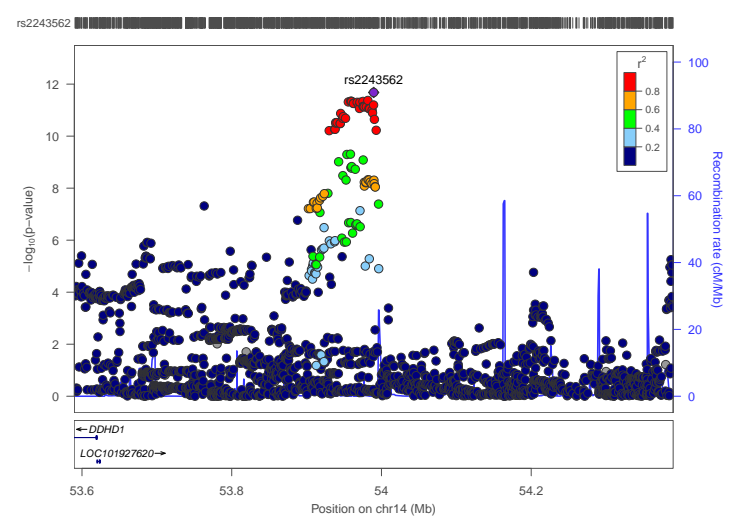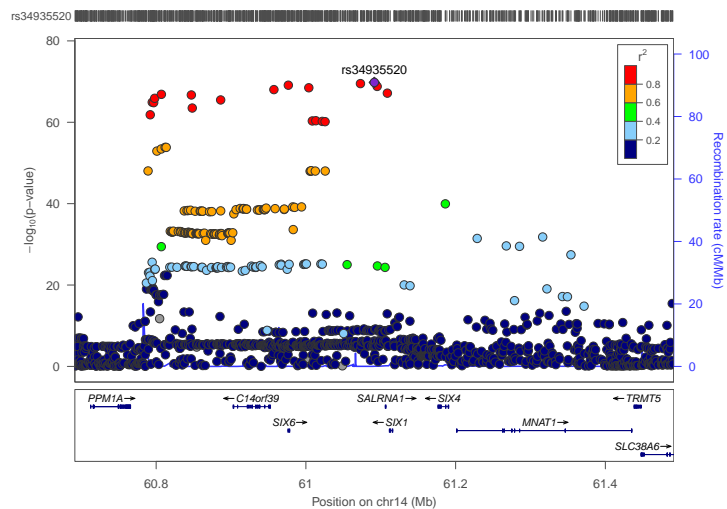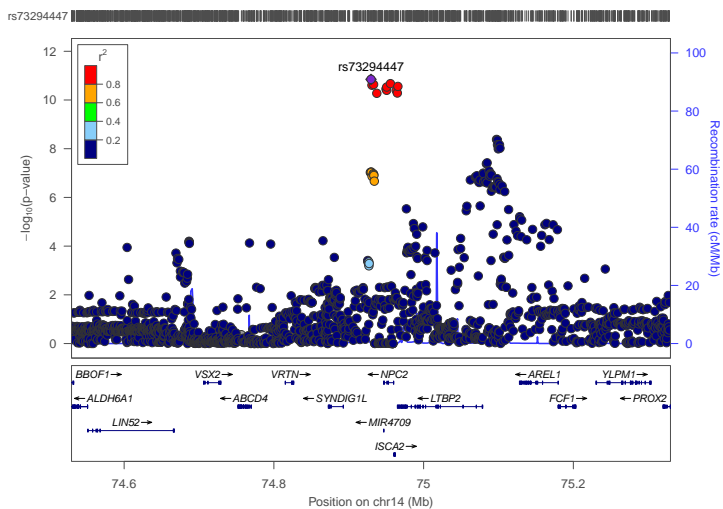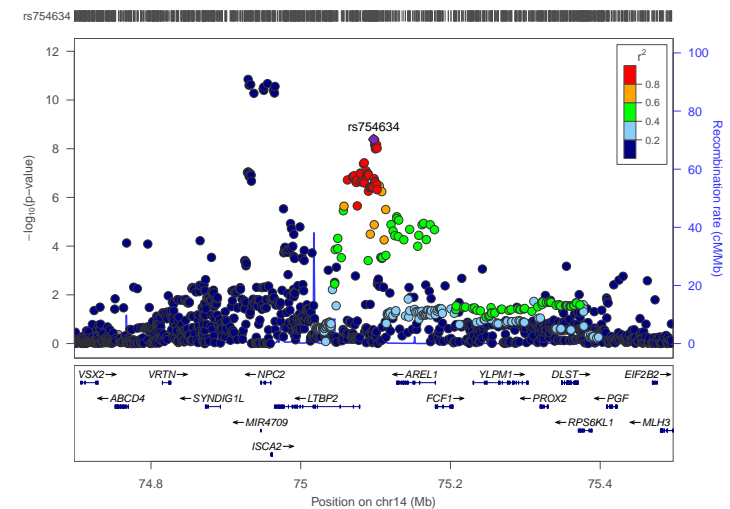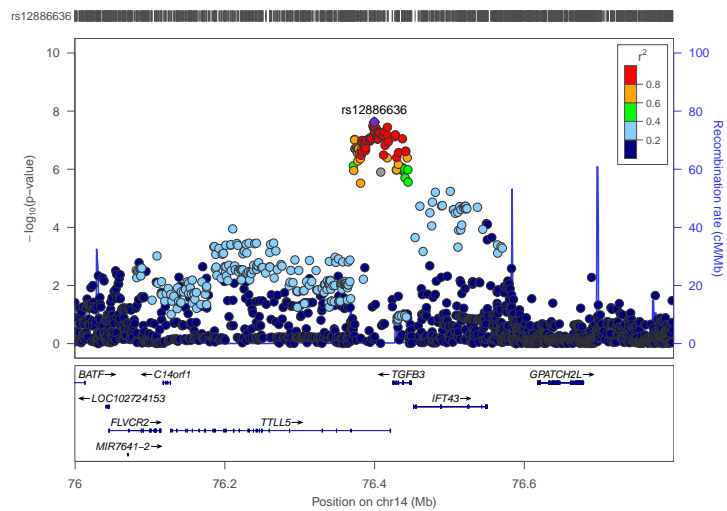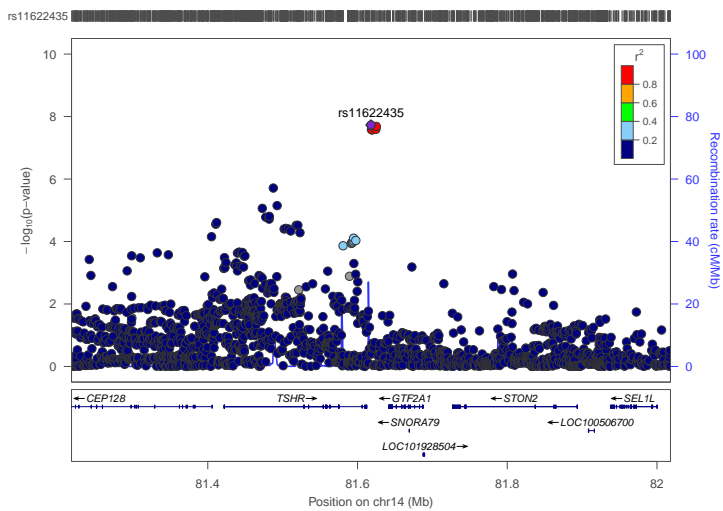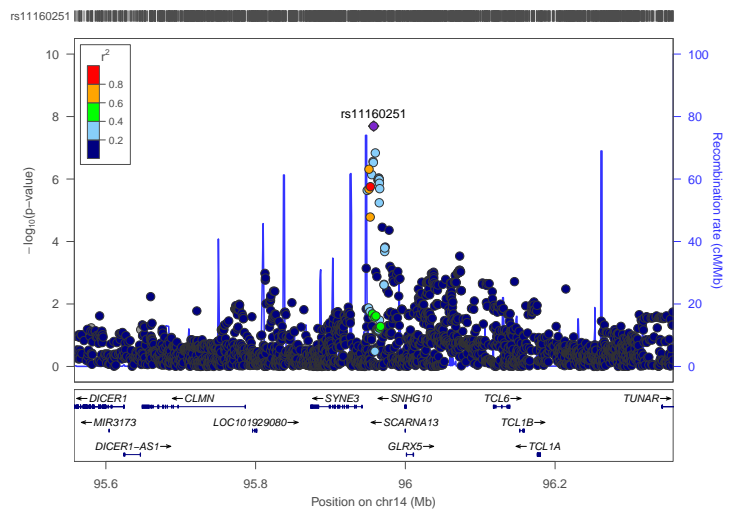

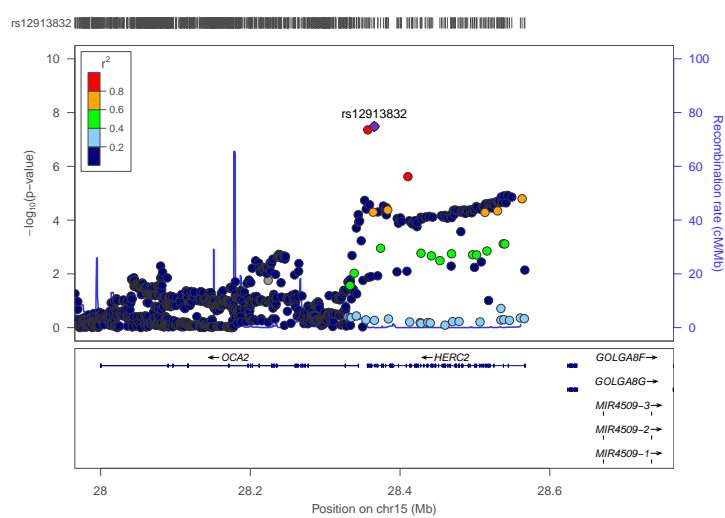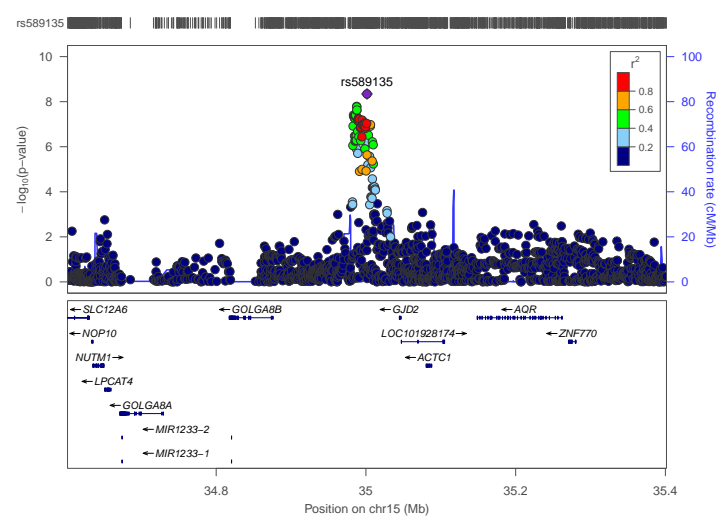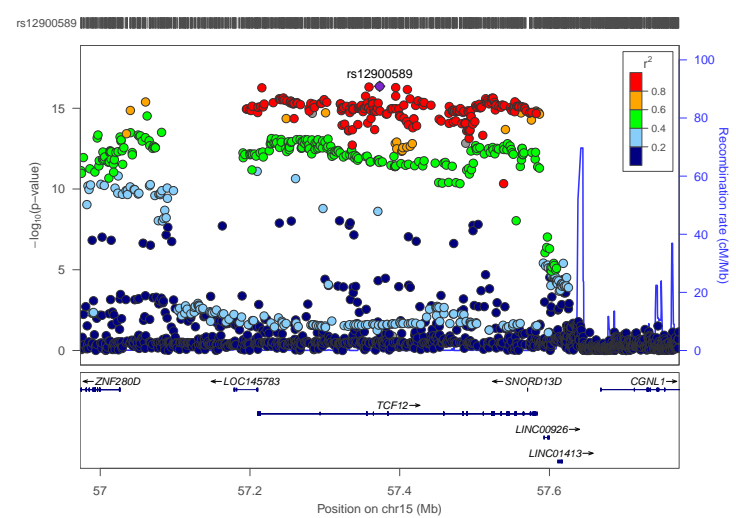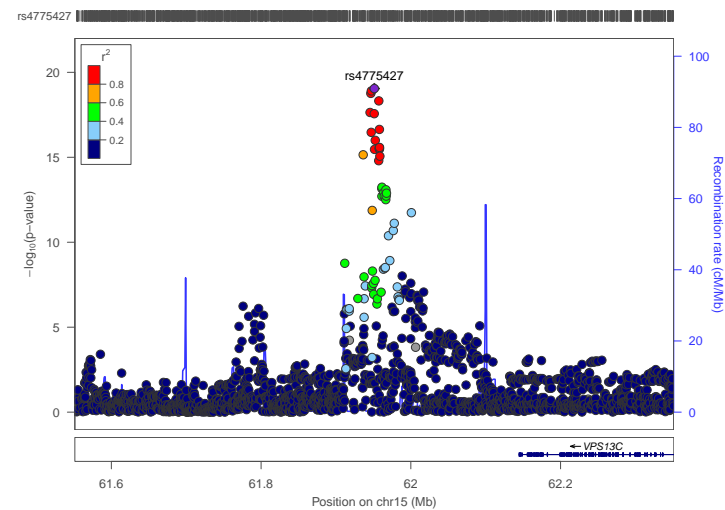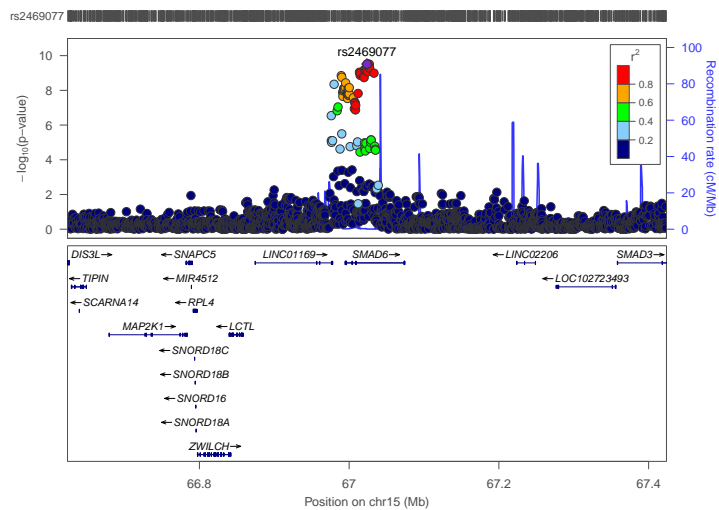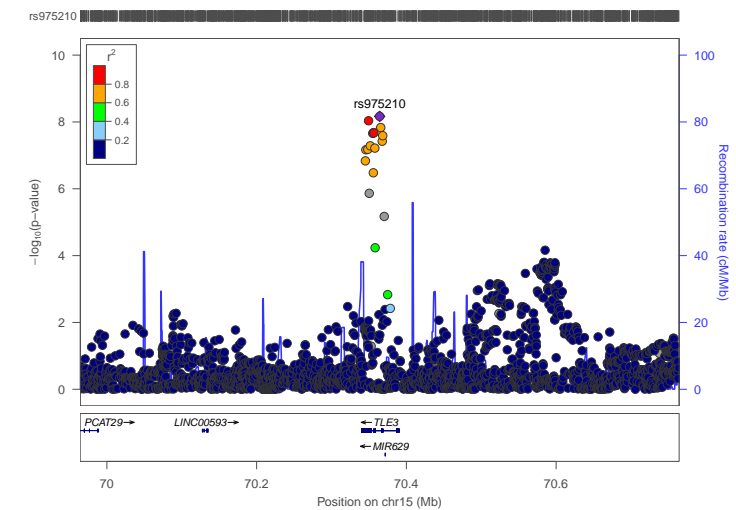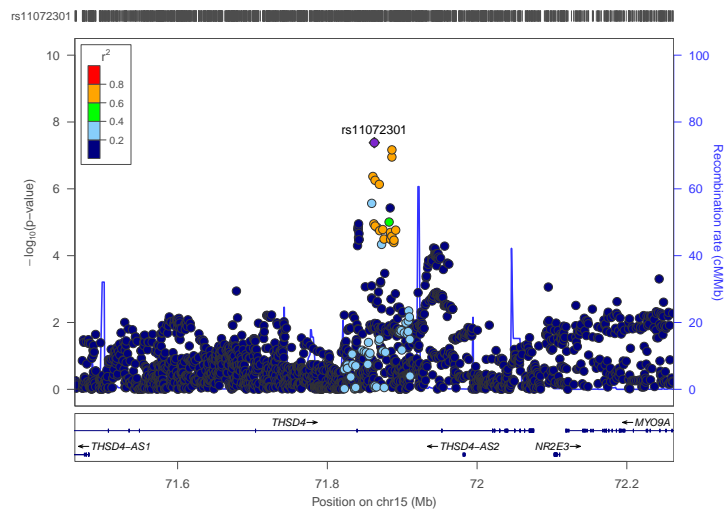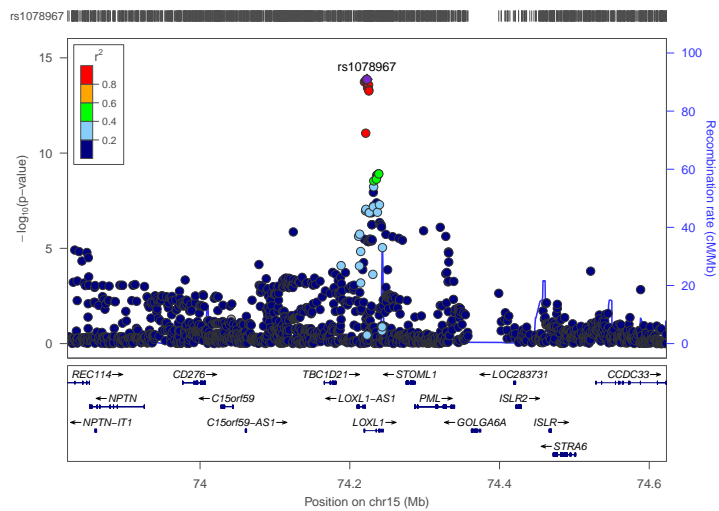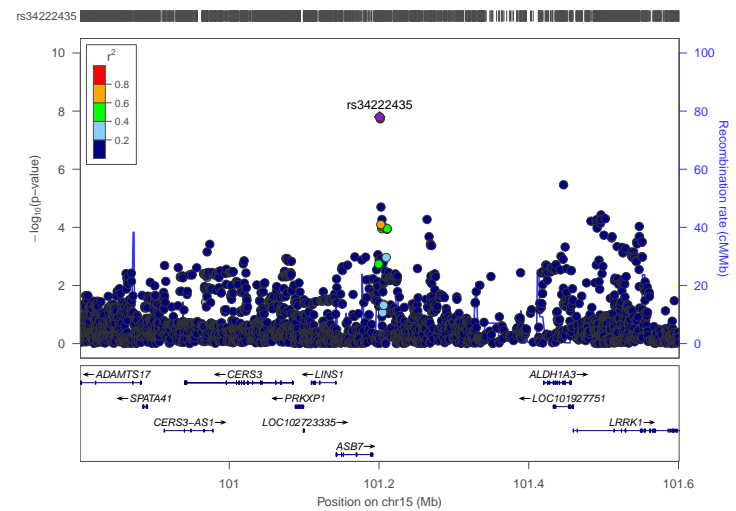

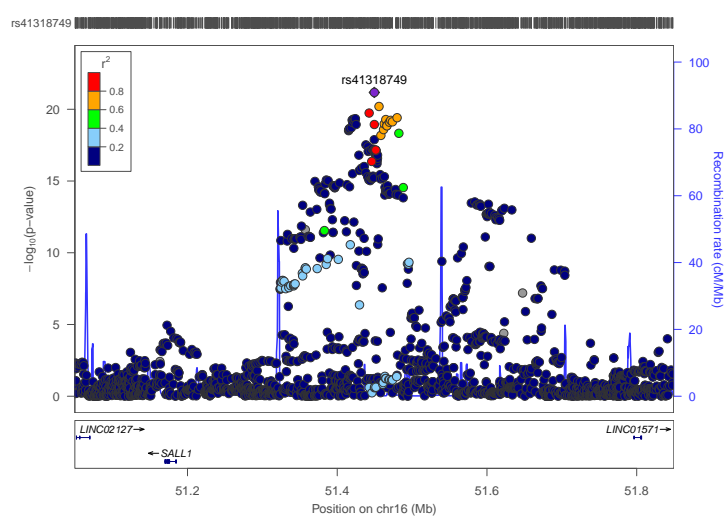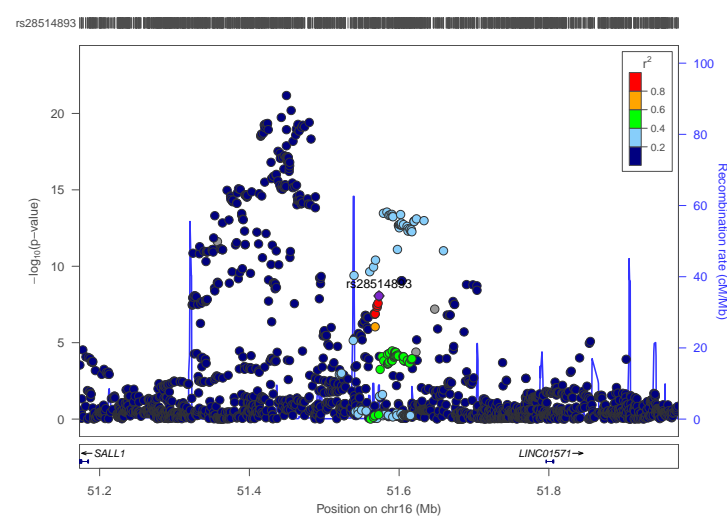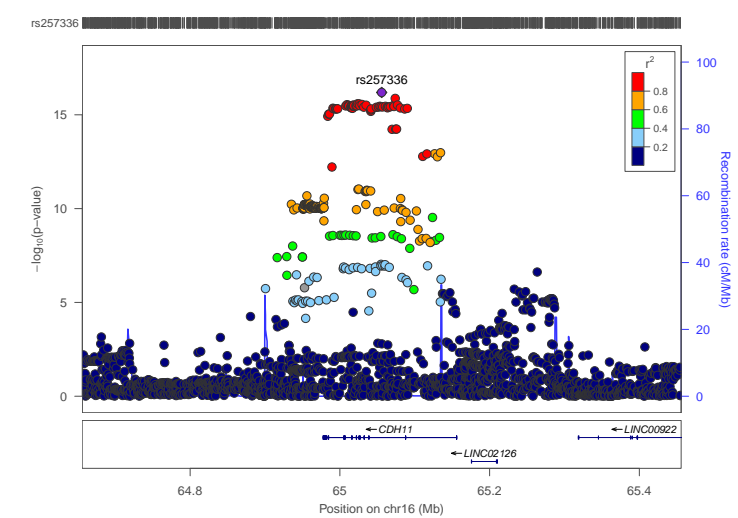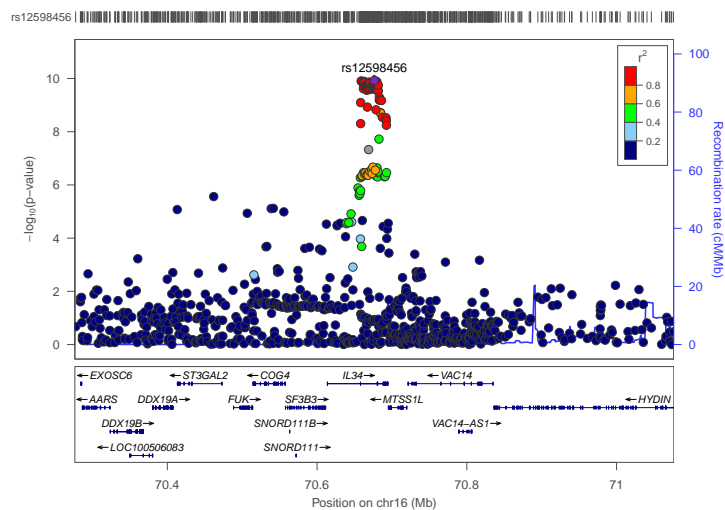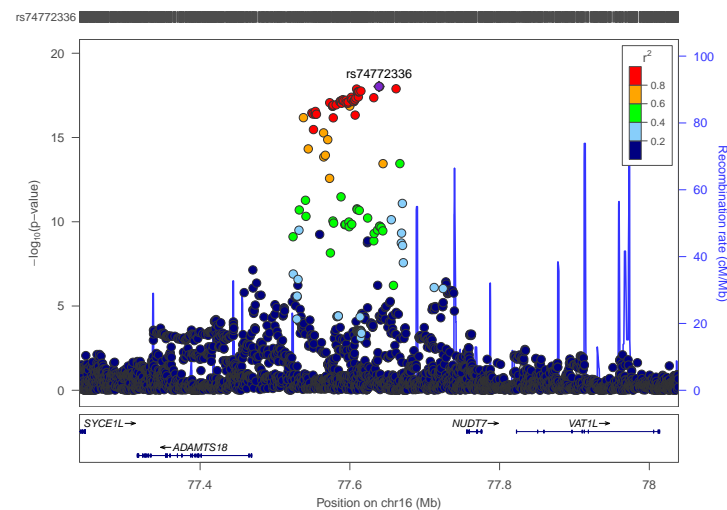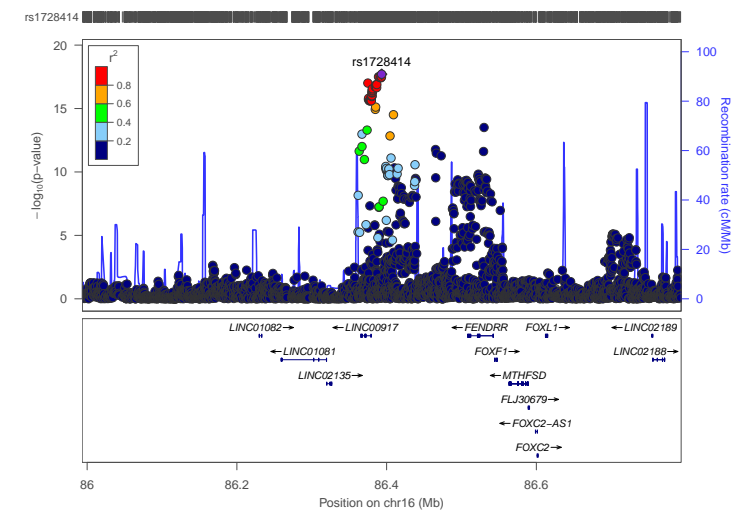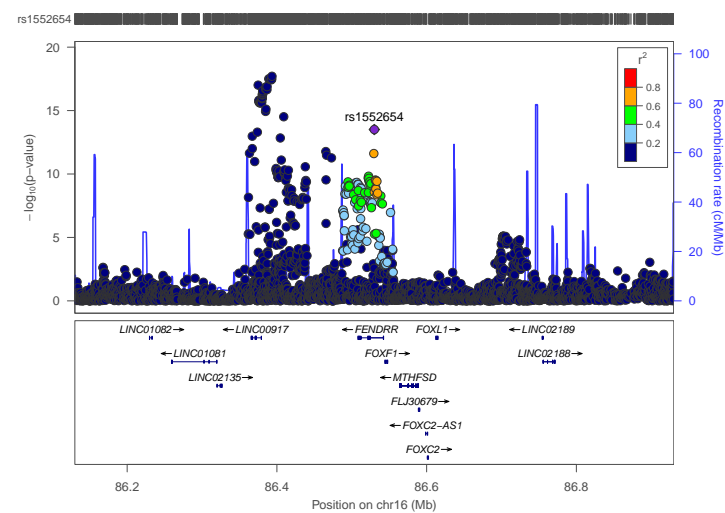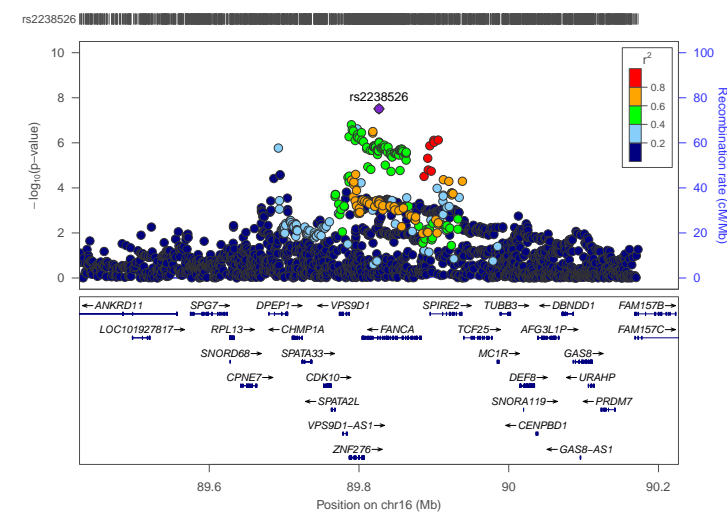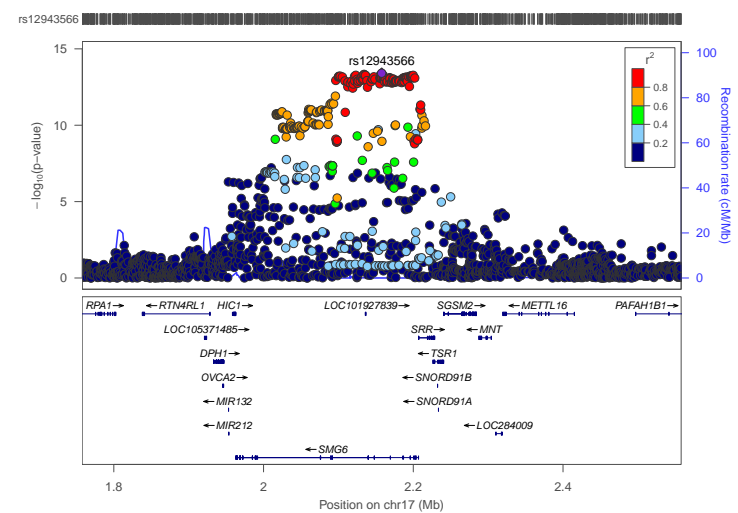

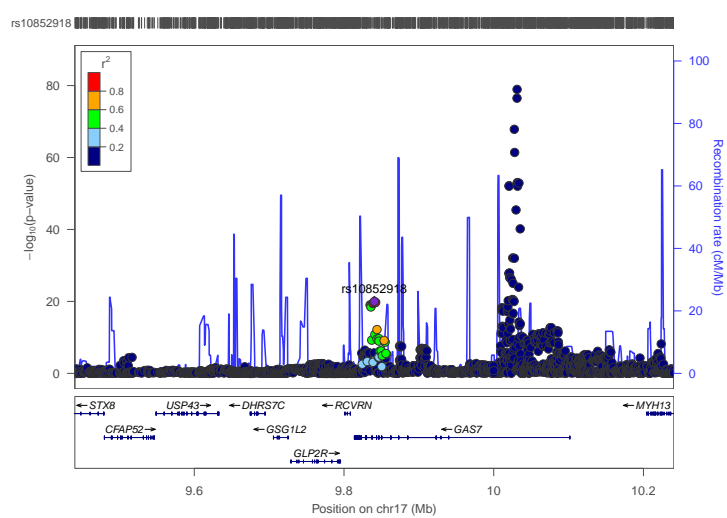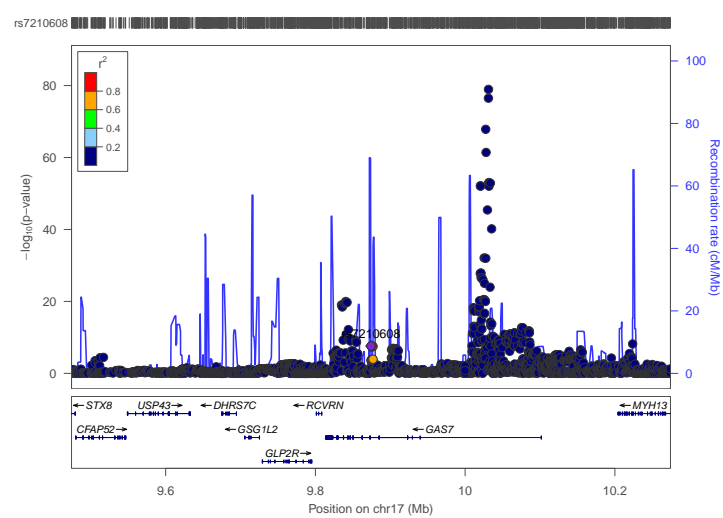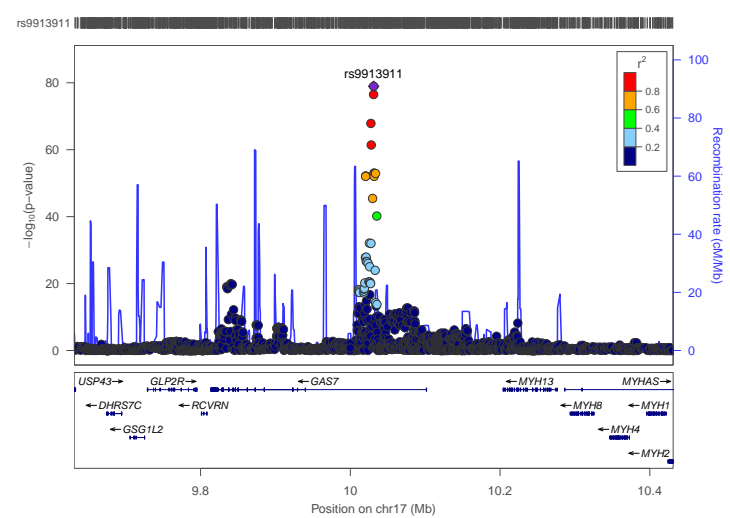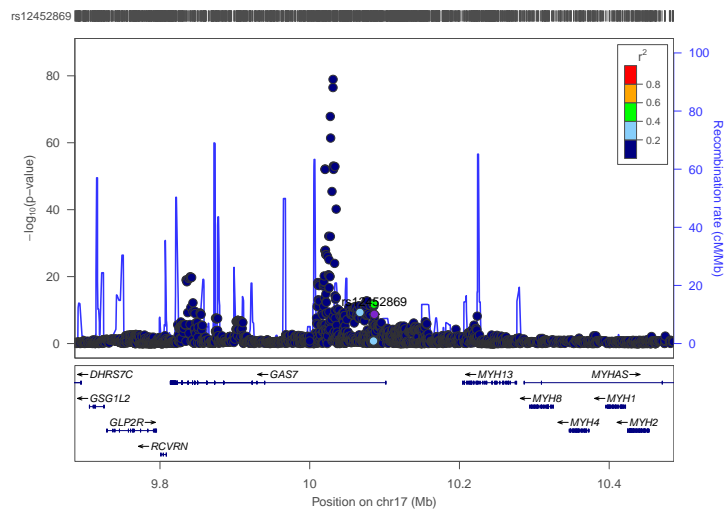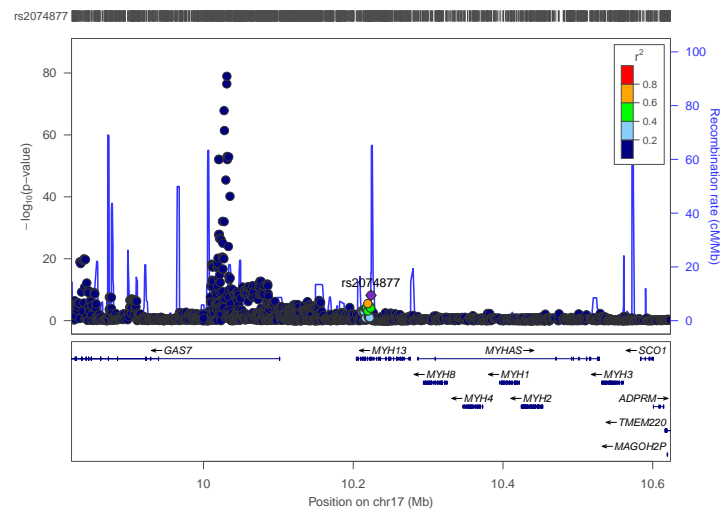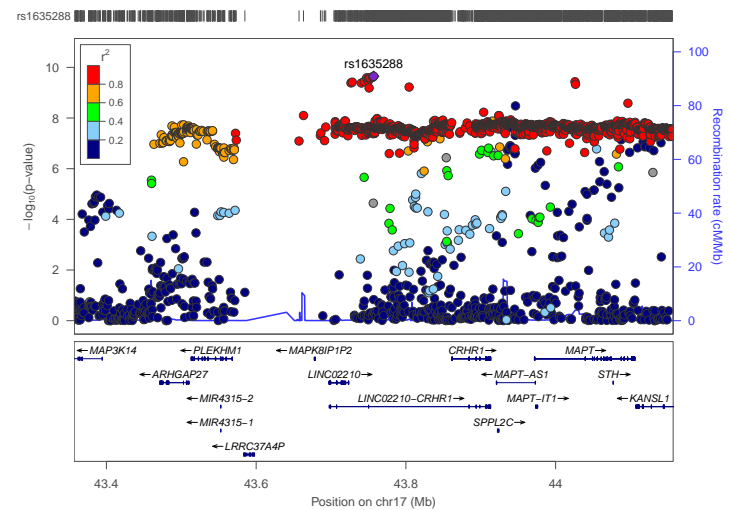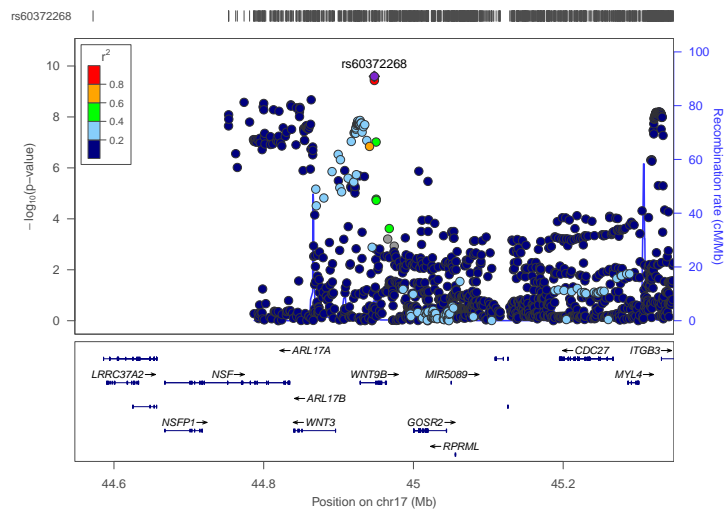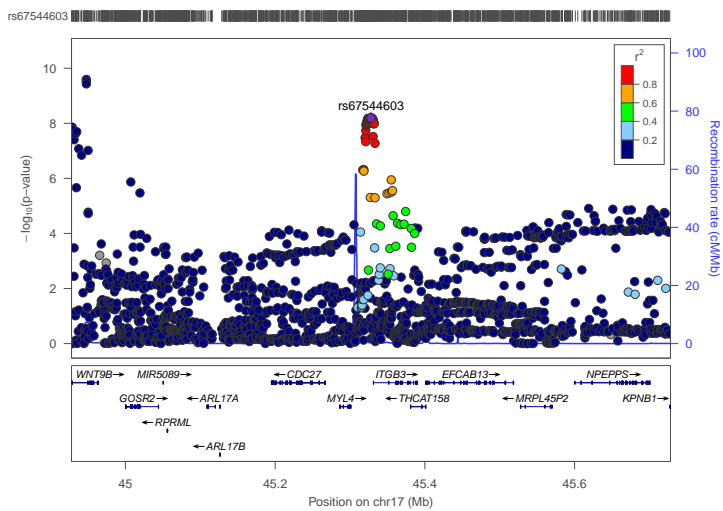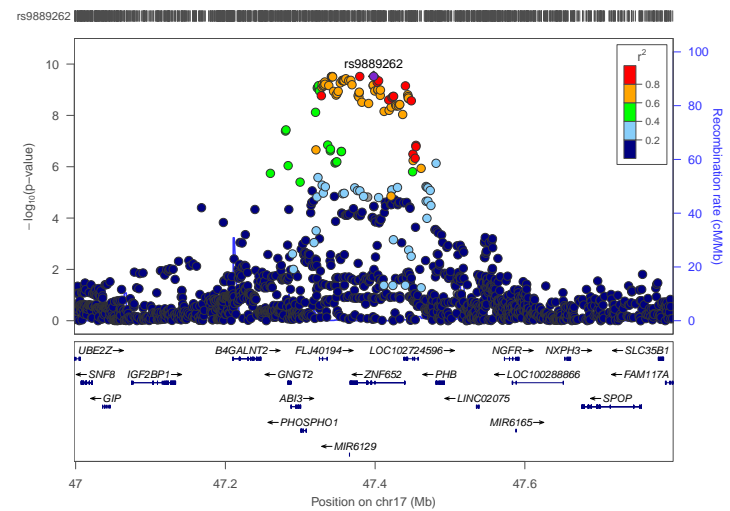

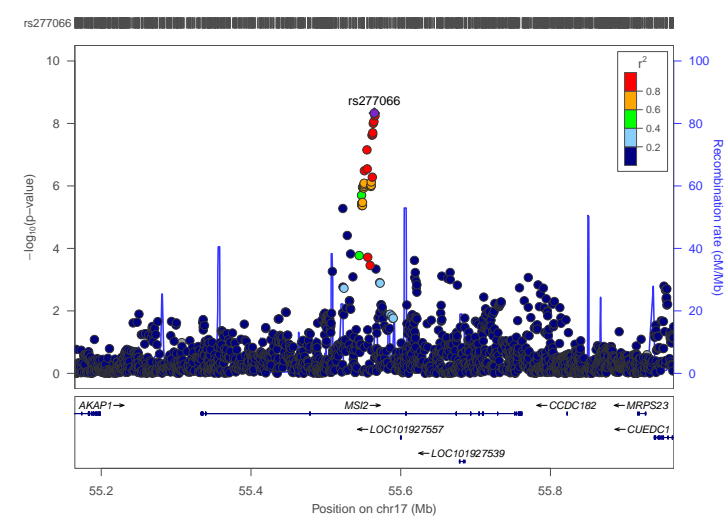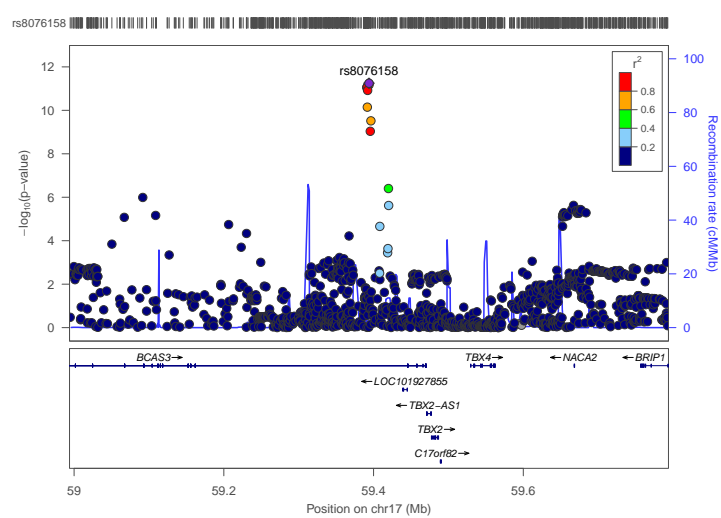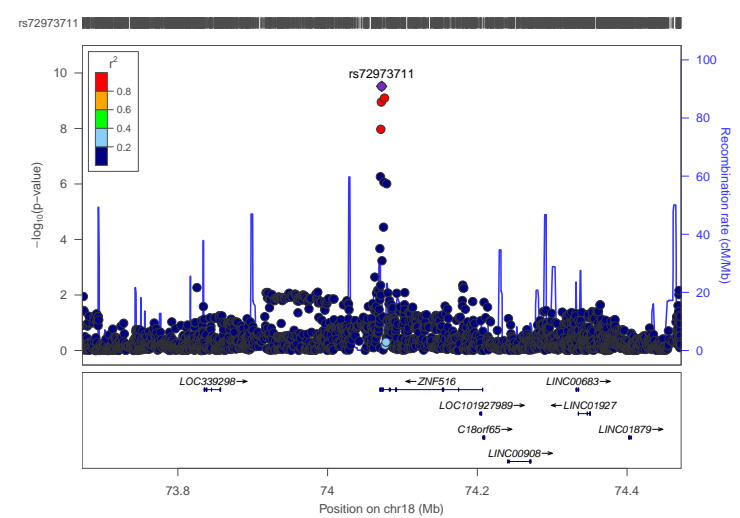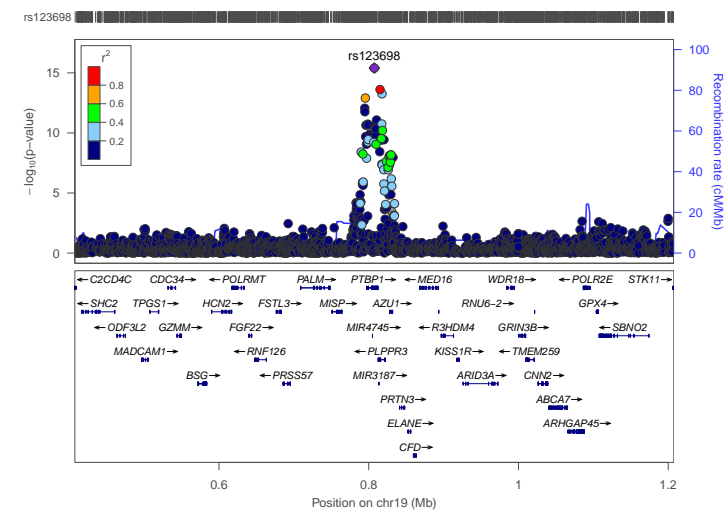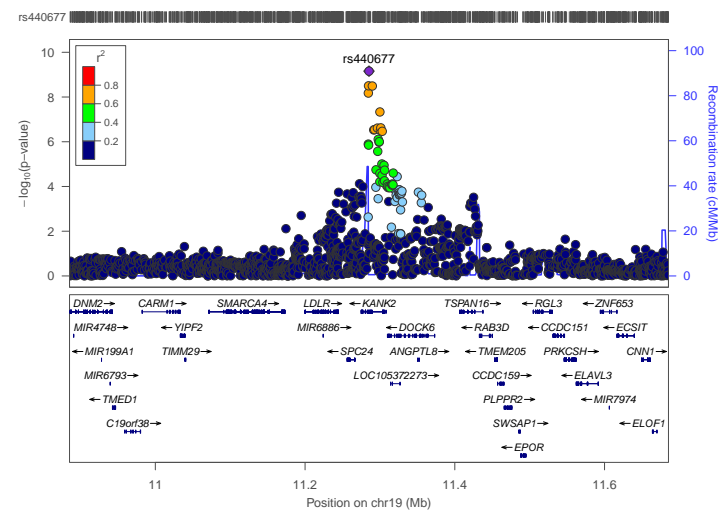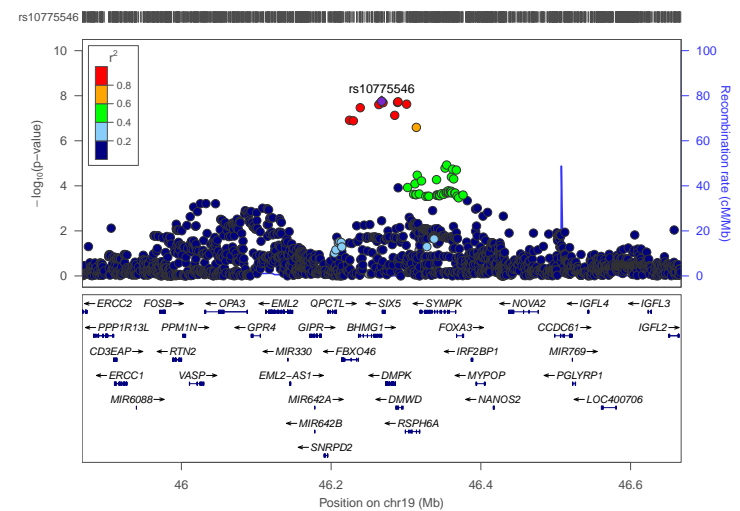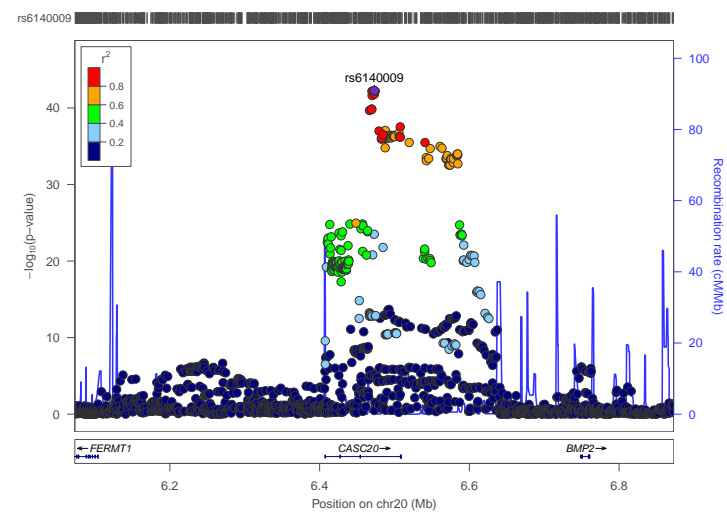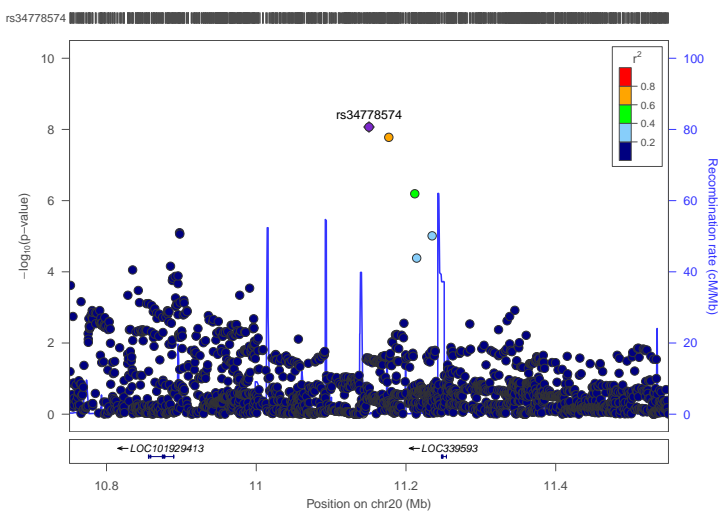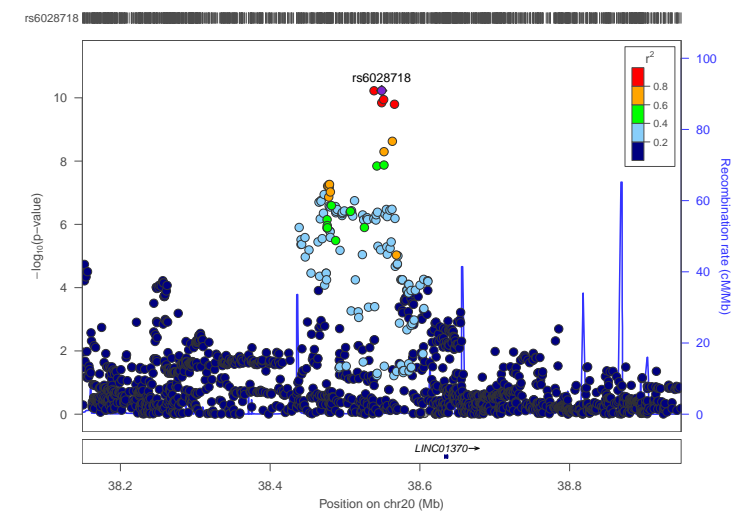

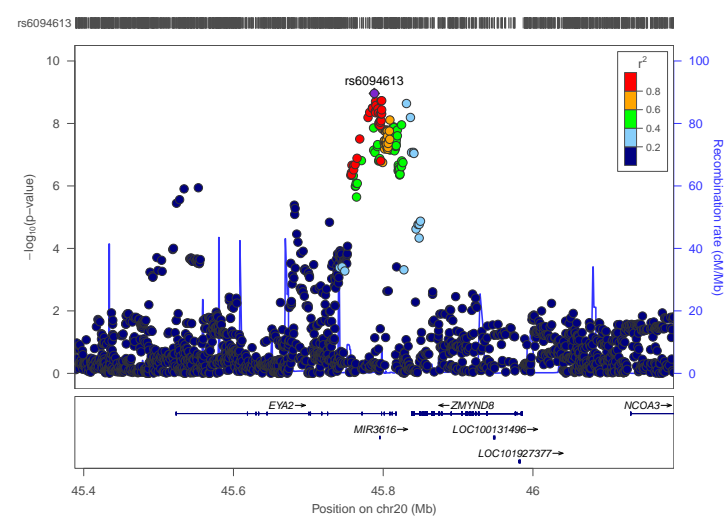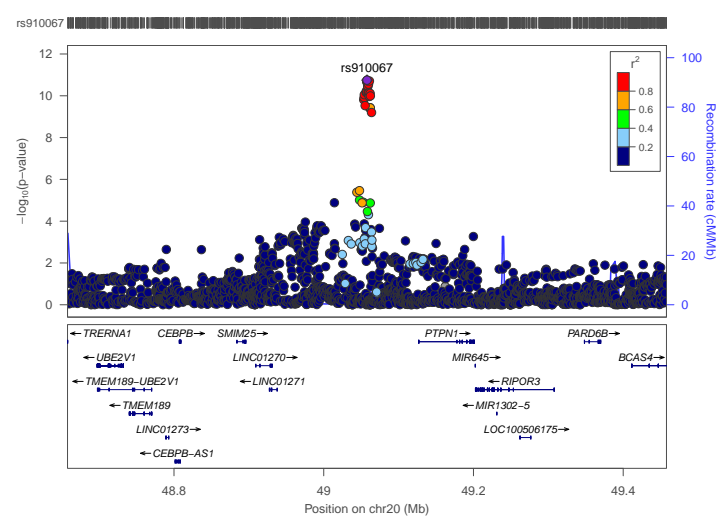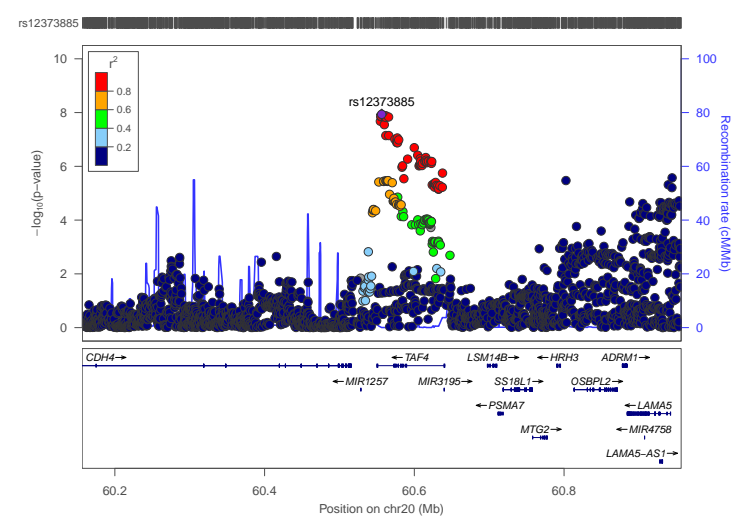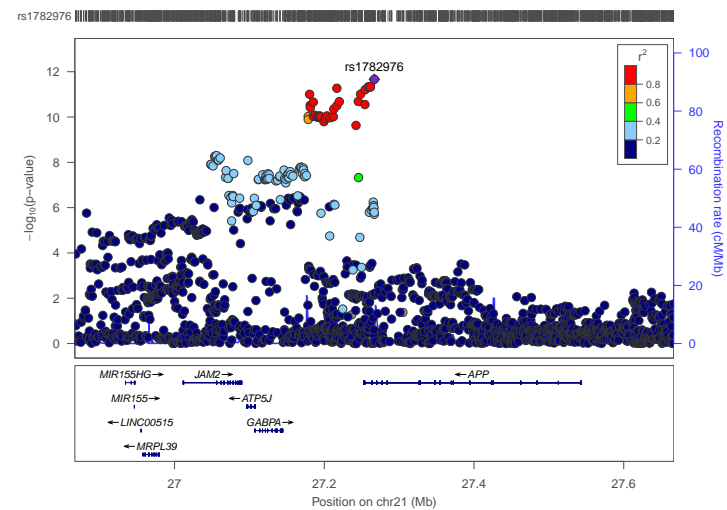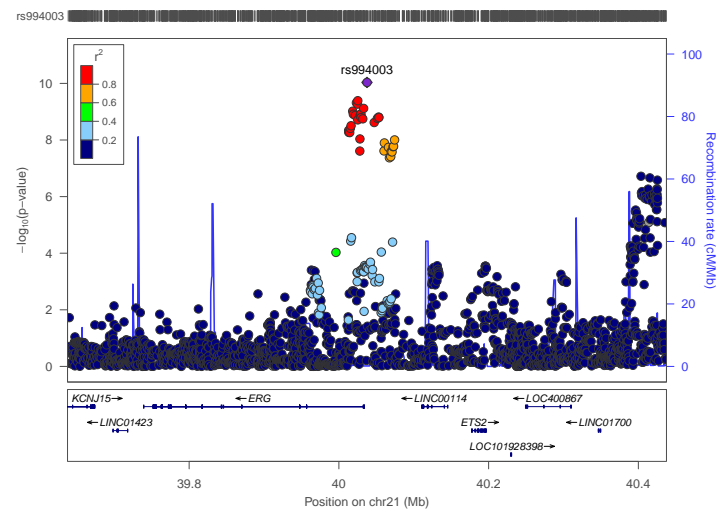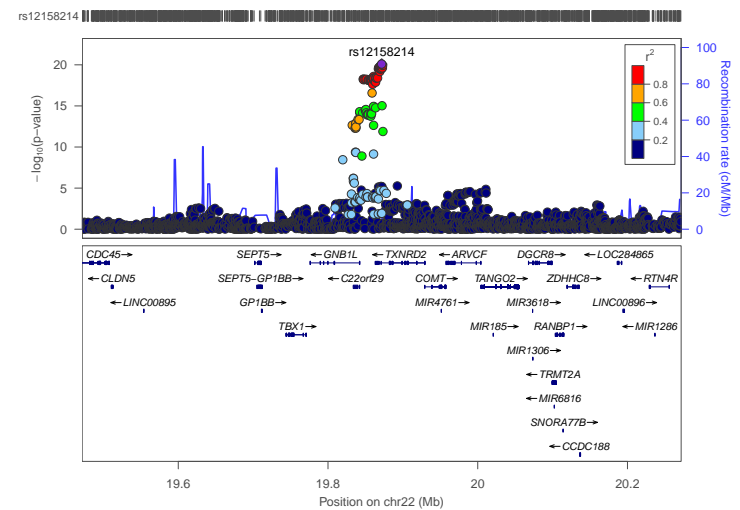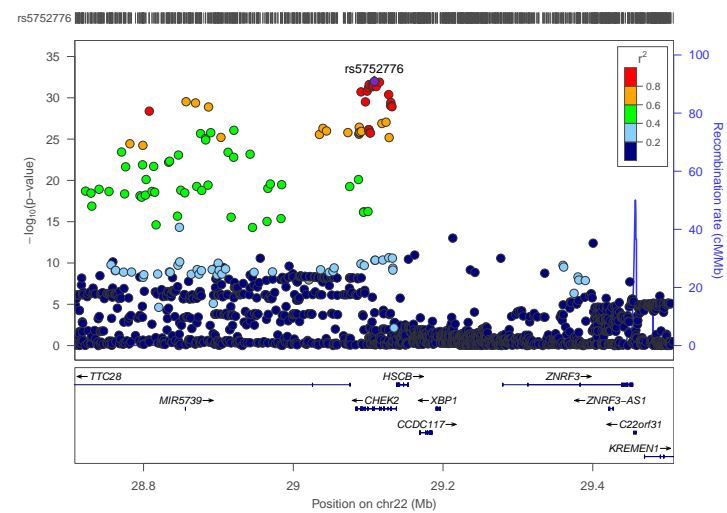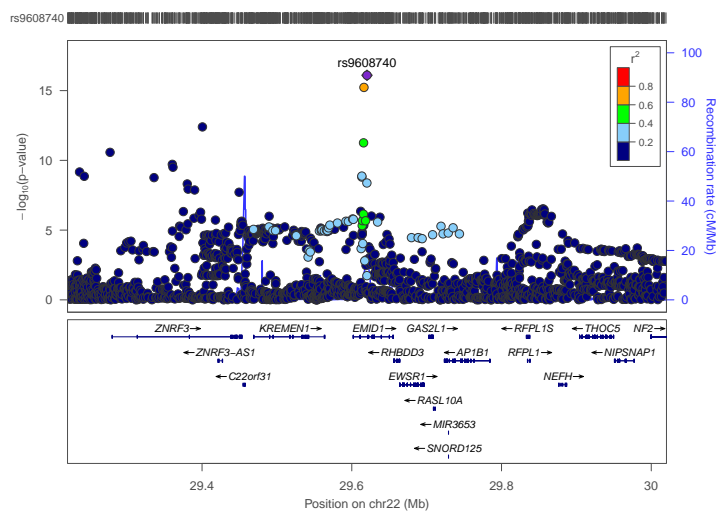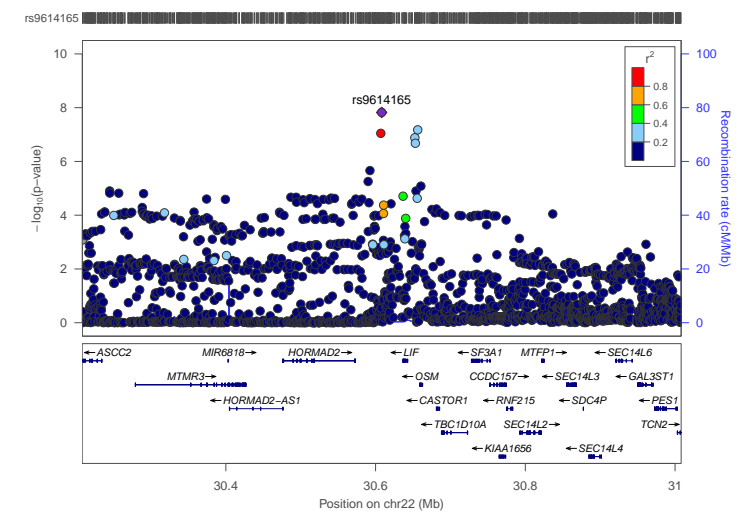

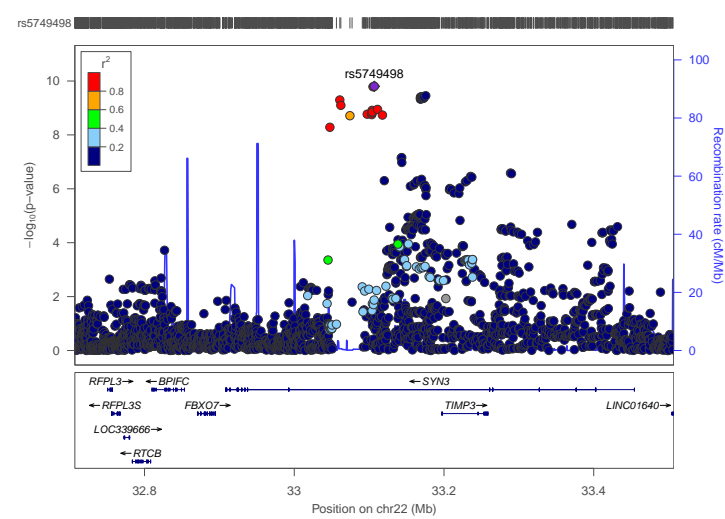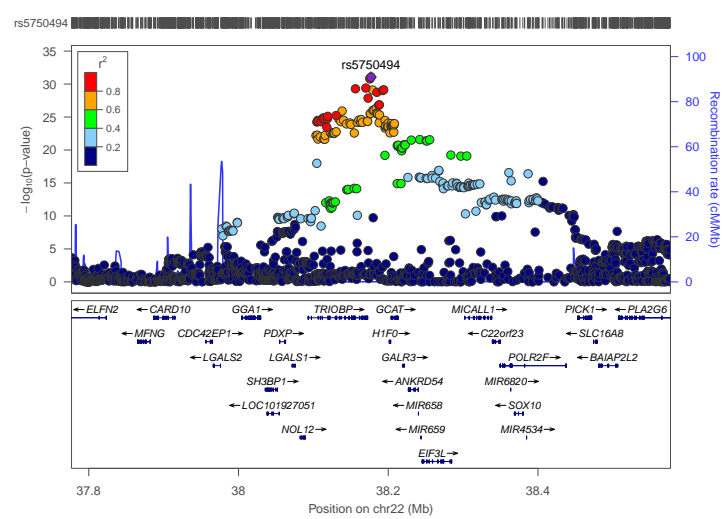

Supplement: Supplementary file 3 — LocusZoom plots for MTAG in European population. [file 41588_2023_1428_MOESM3_ESM.pdf]

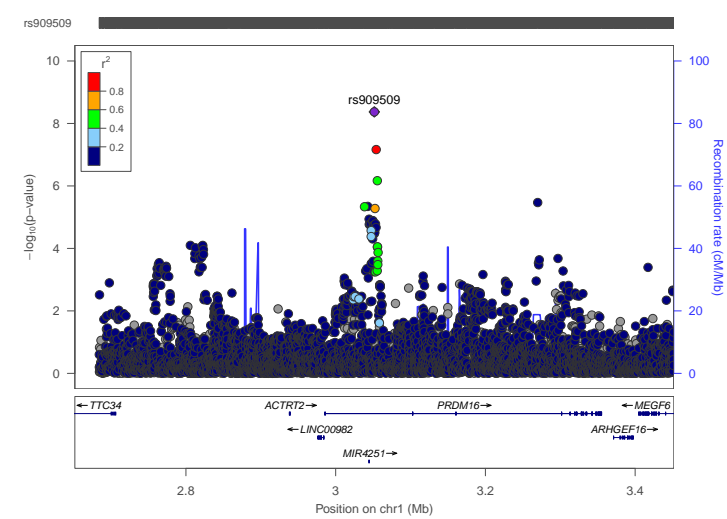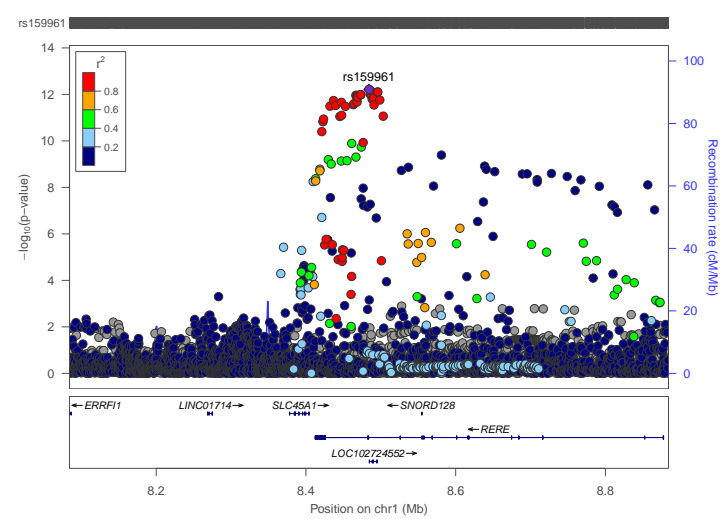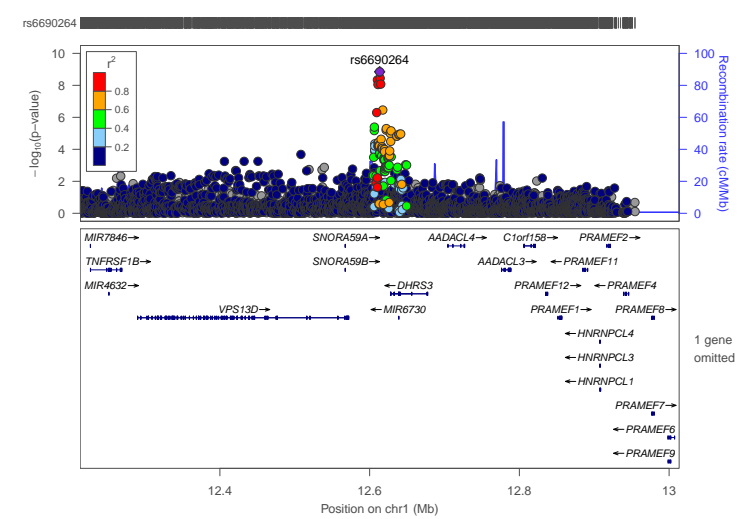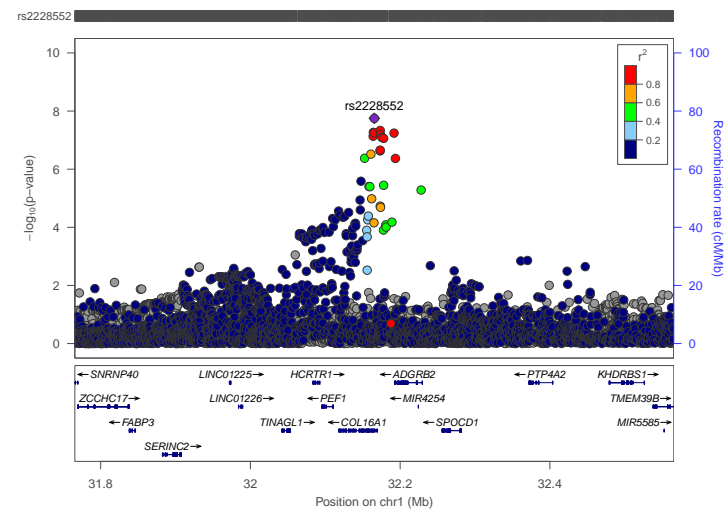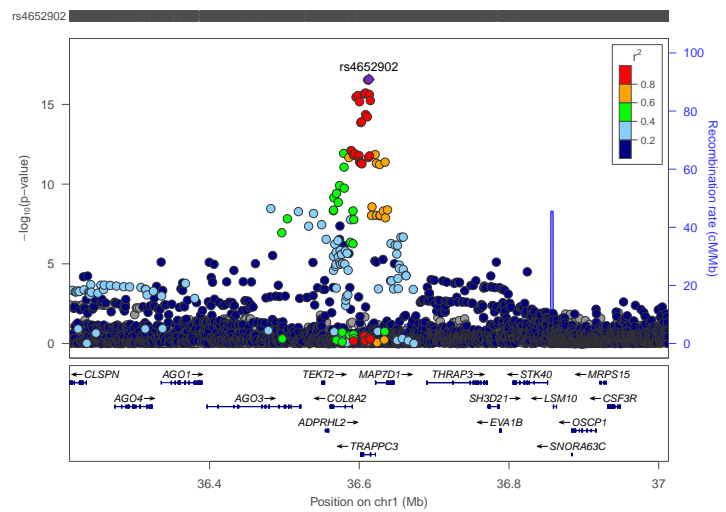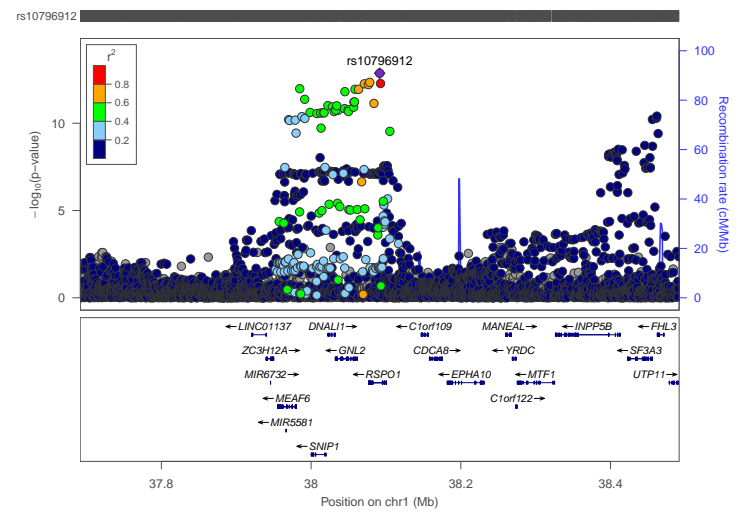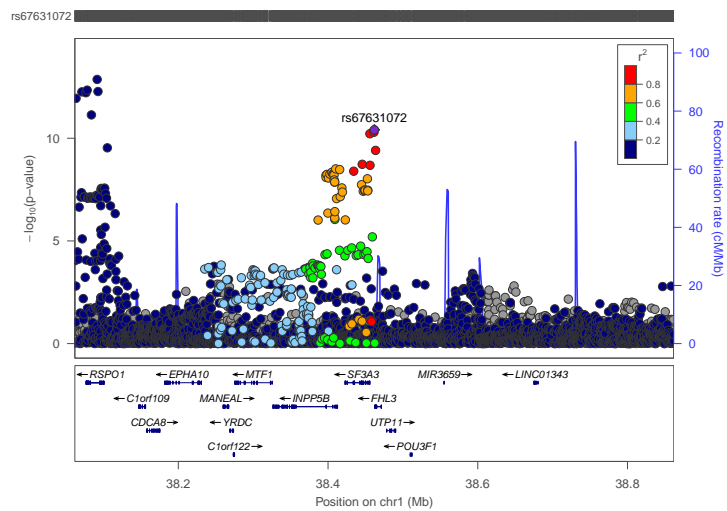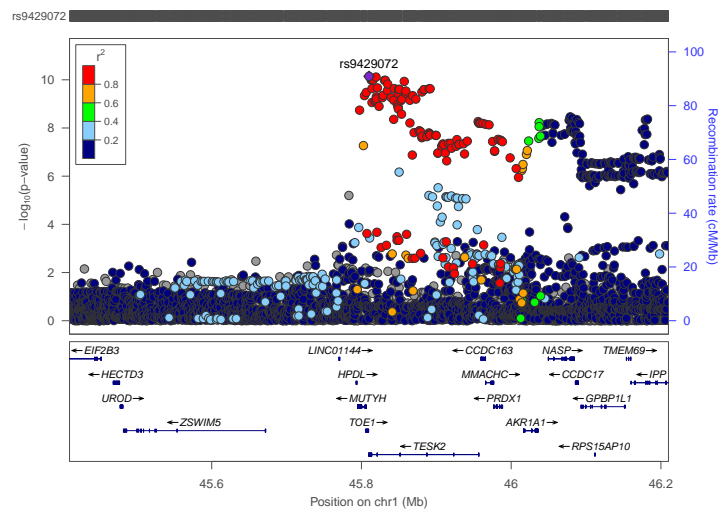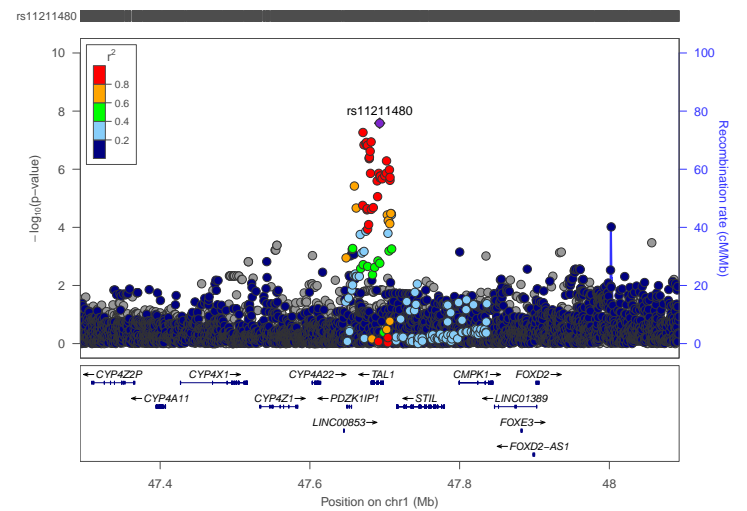

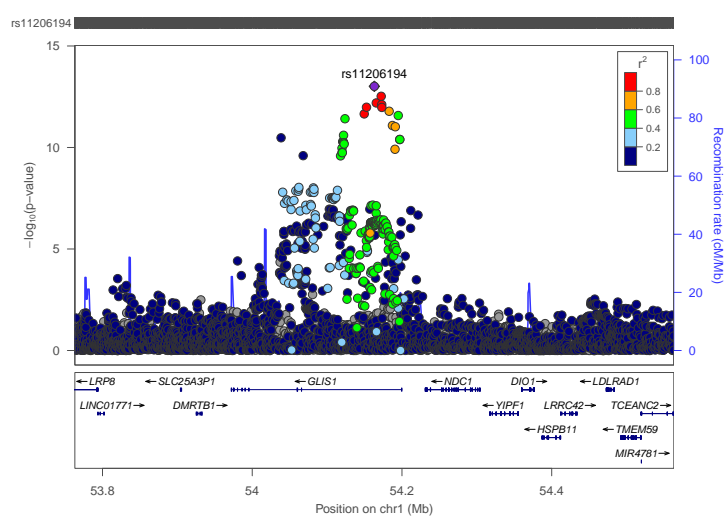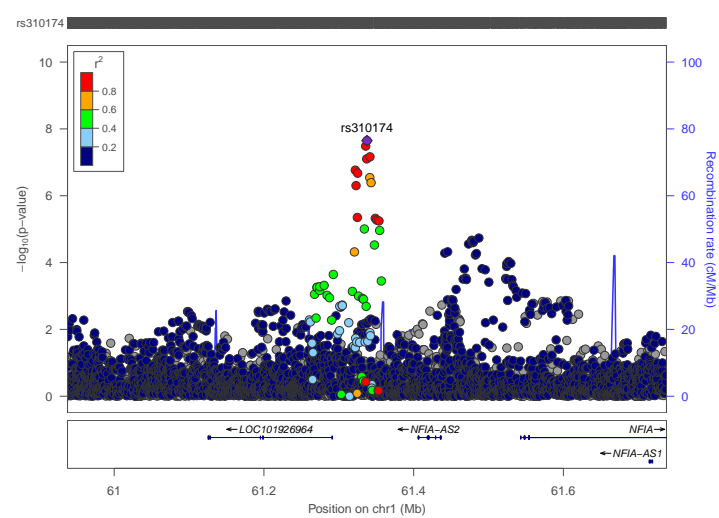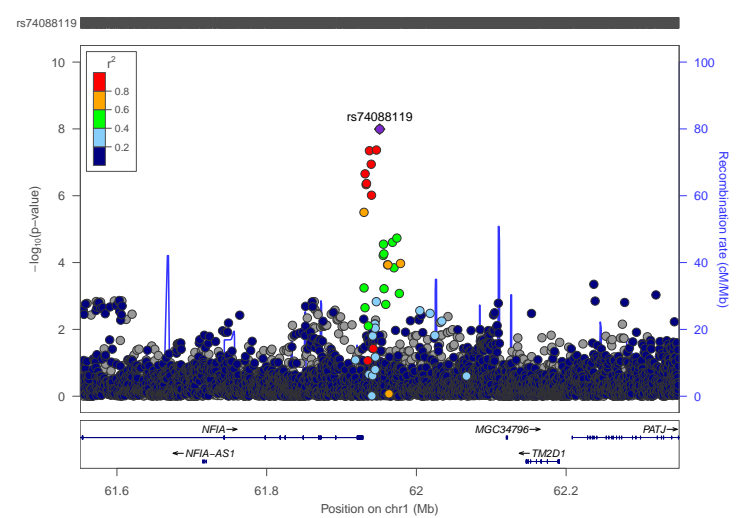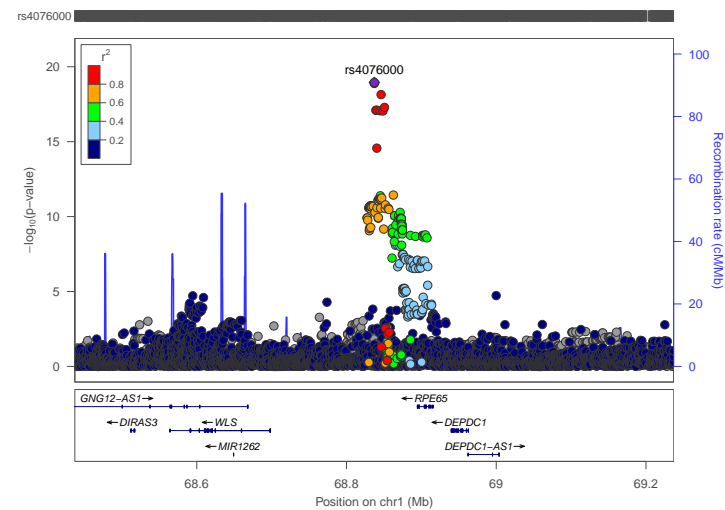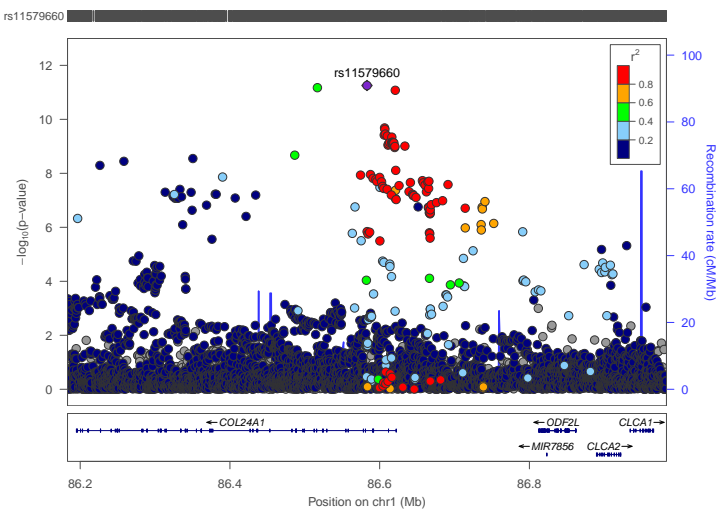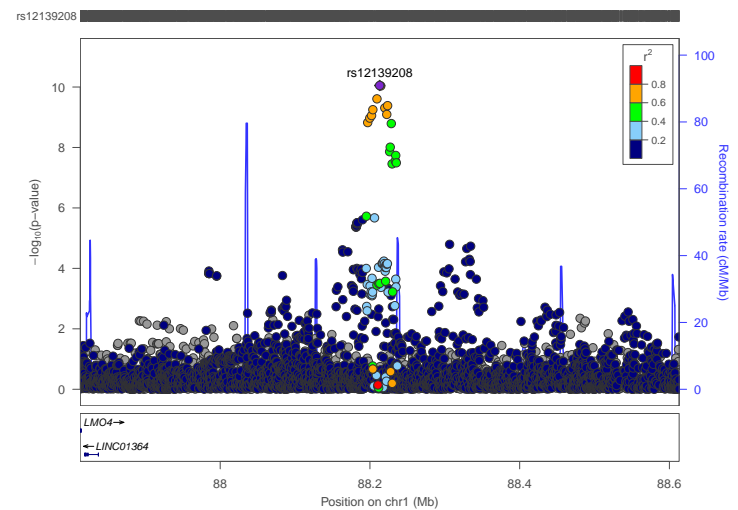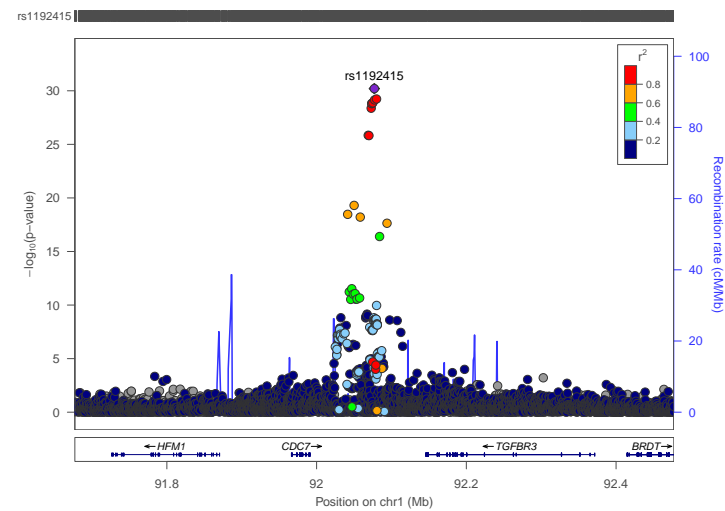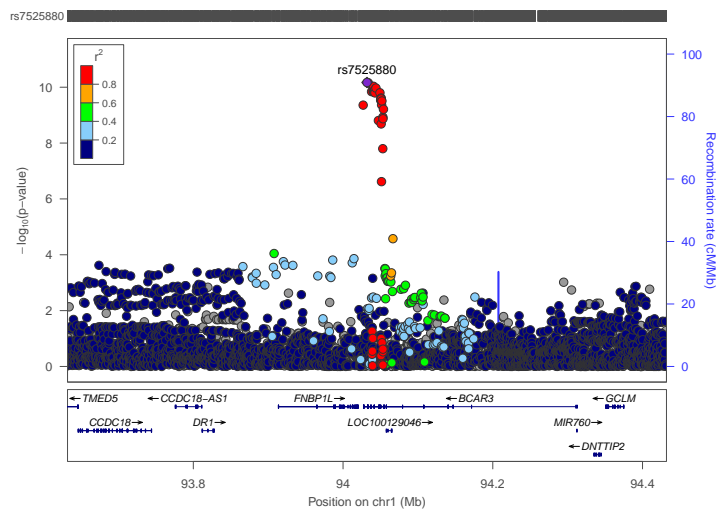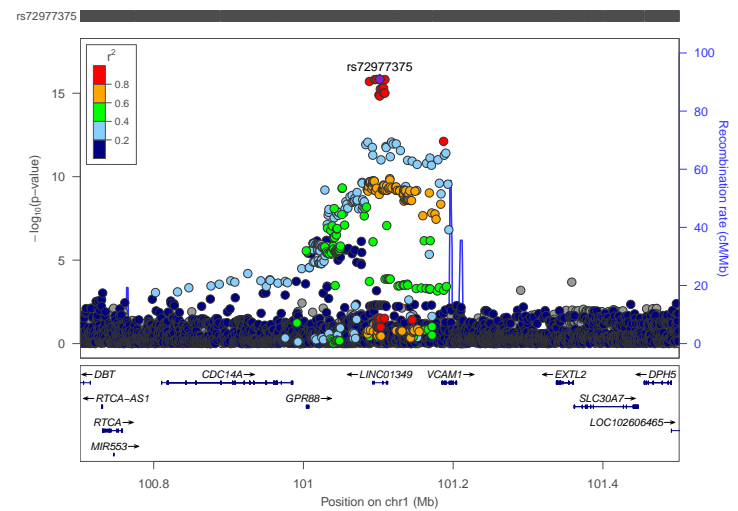

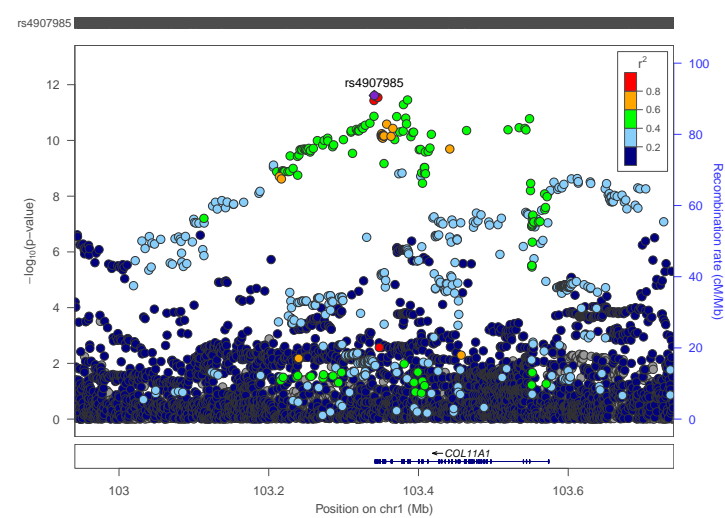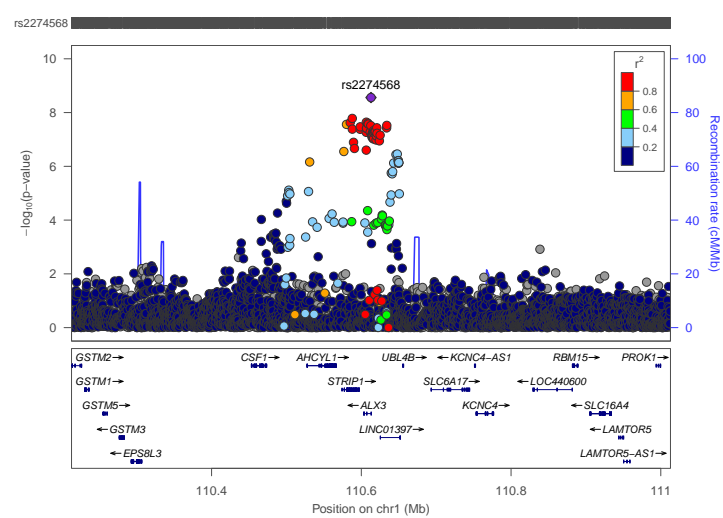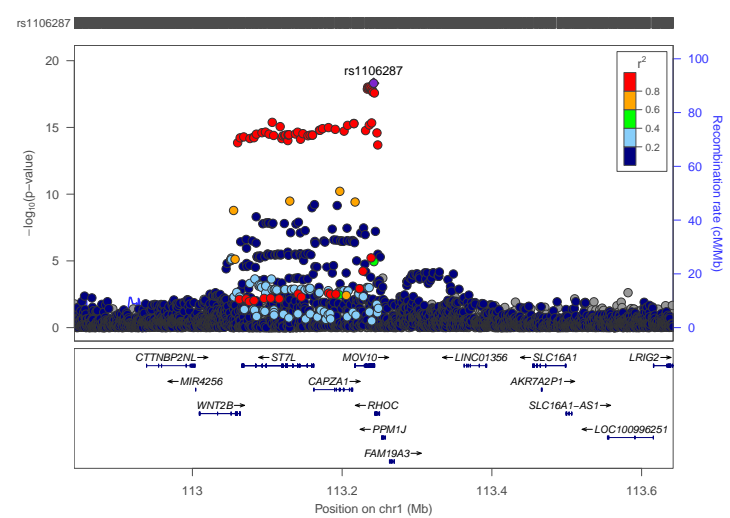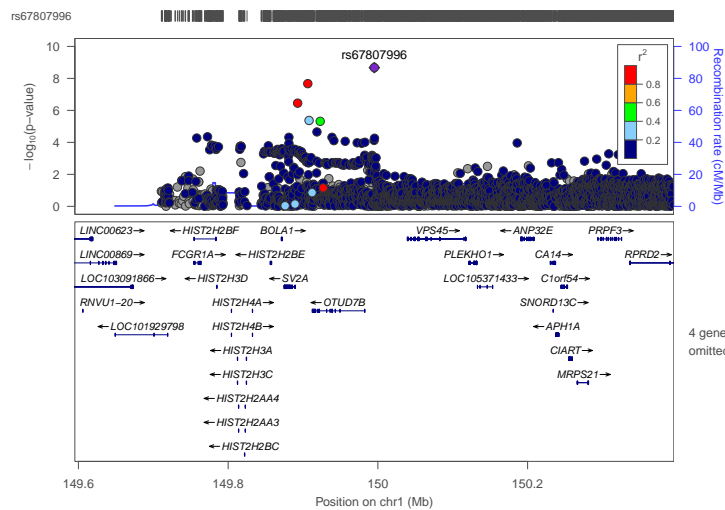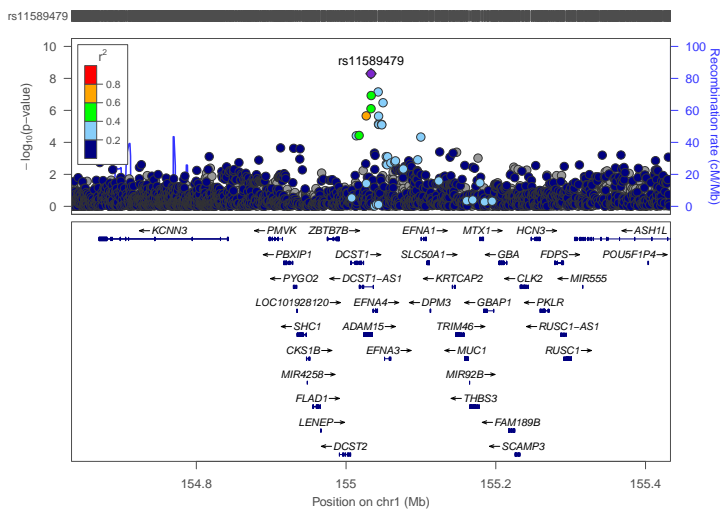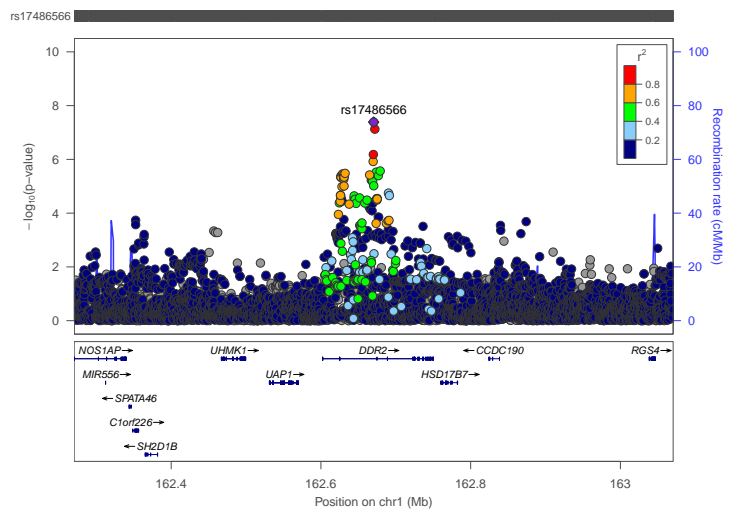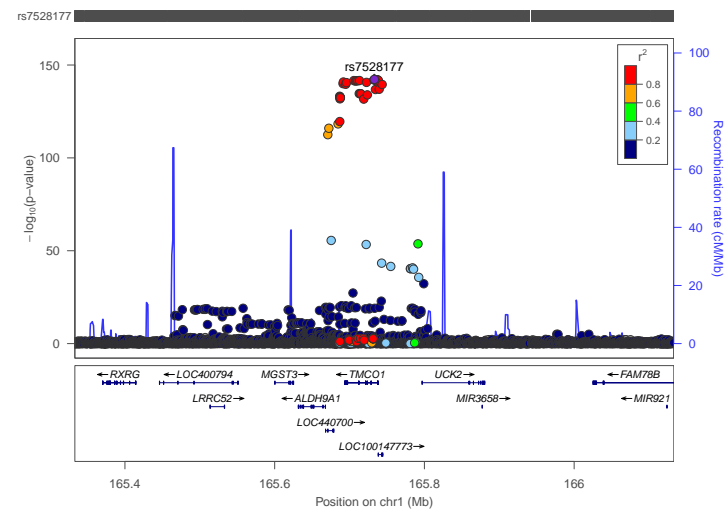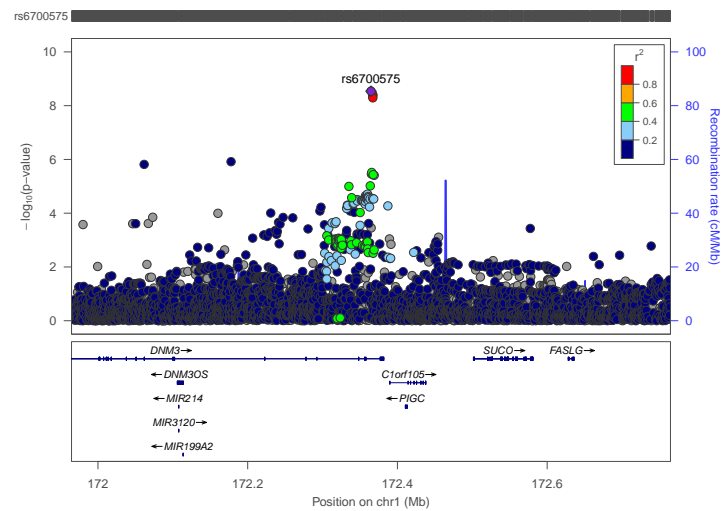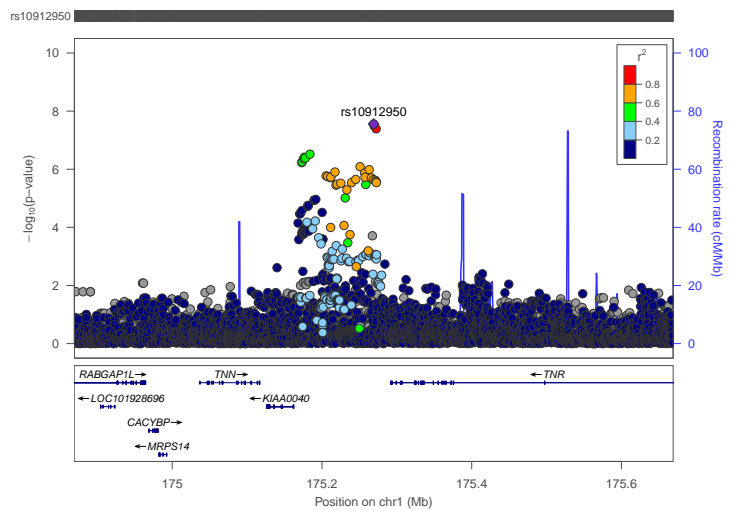

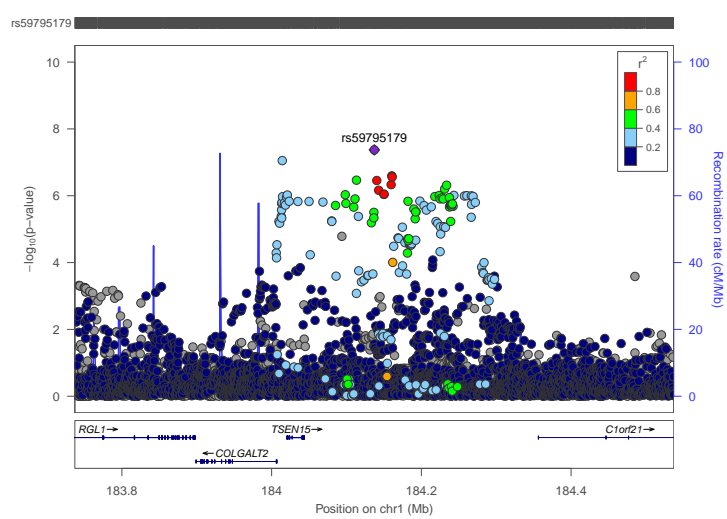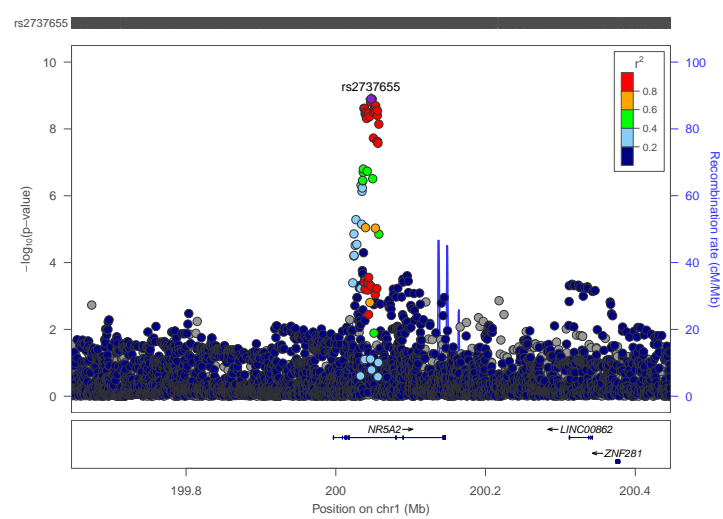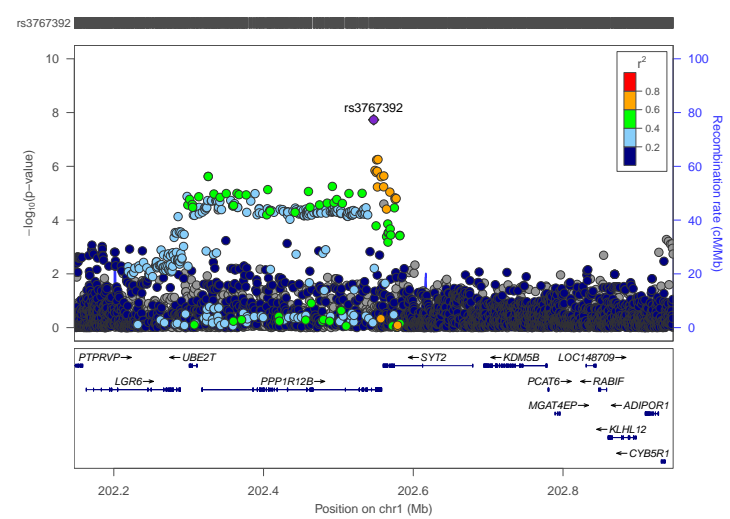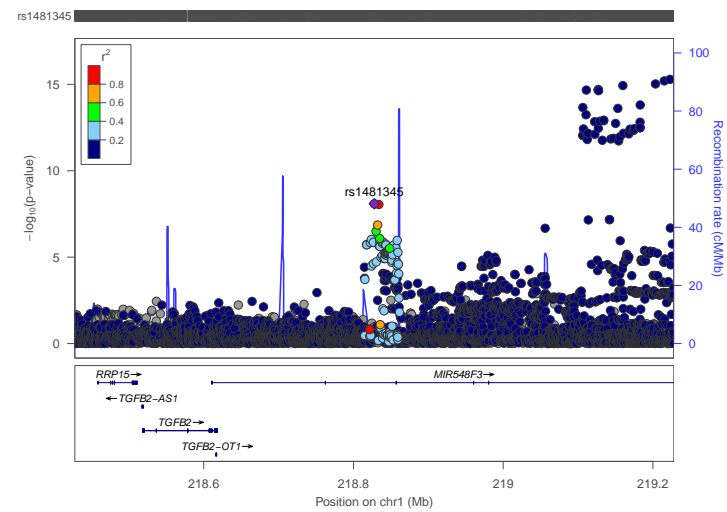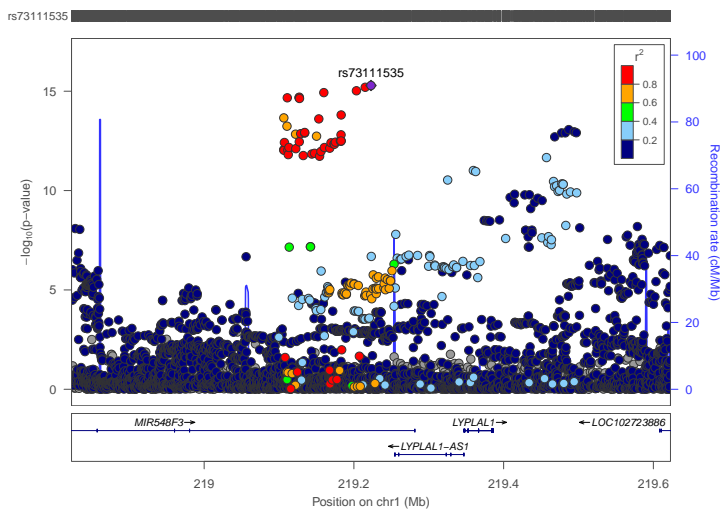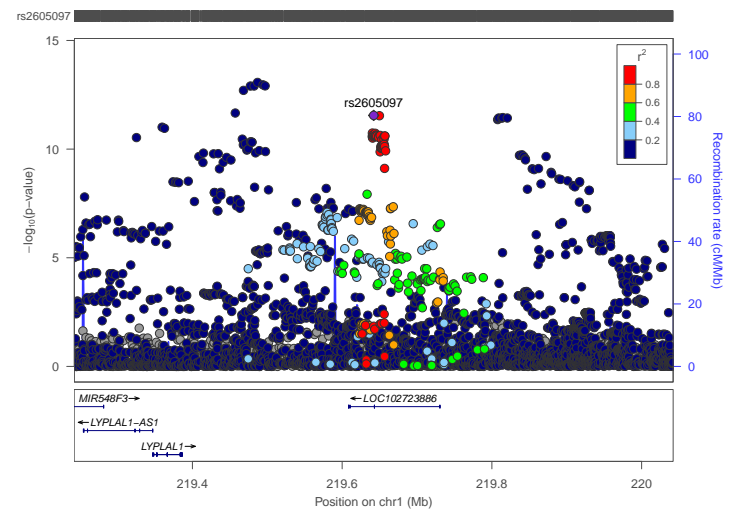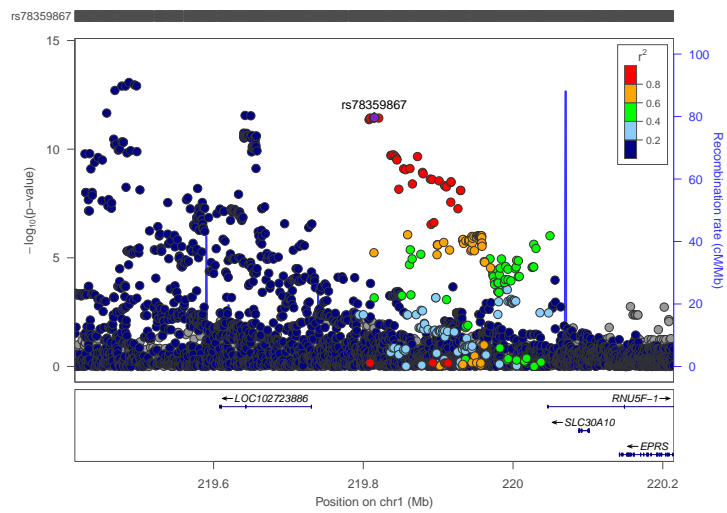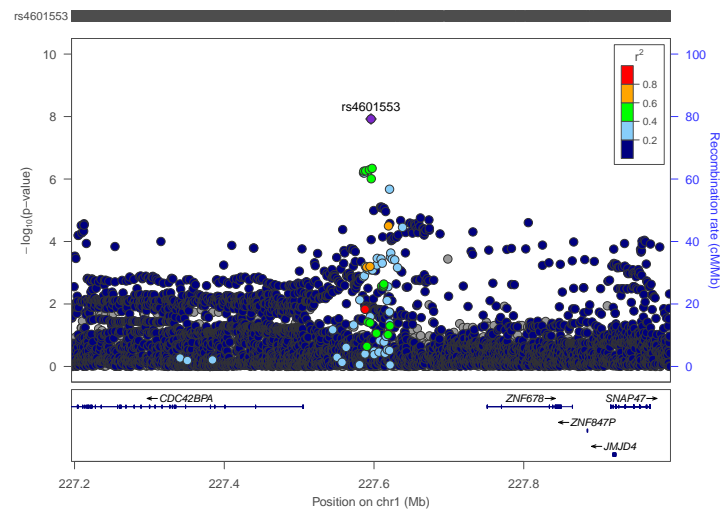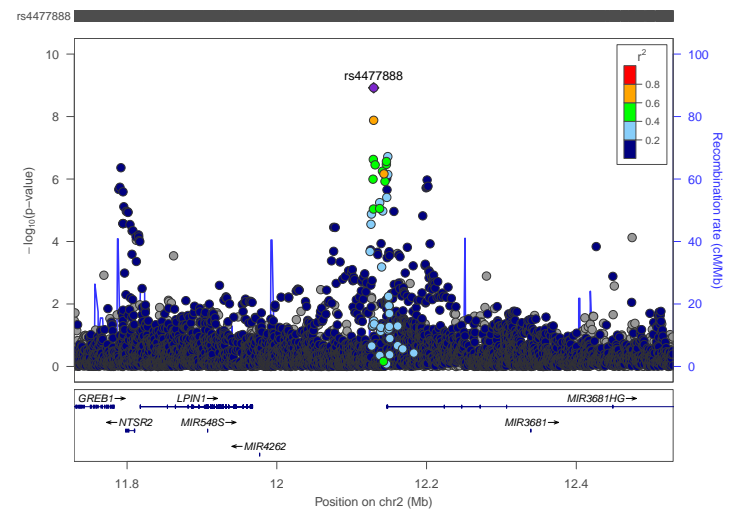

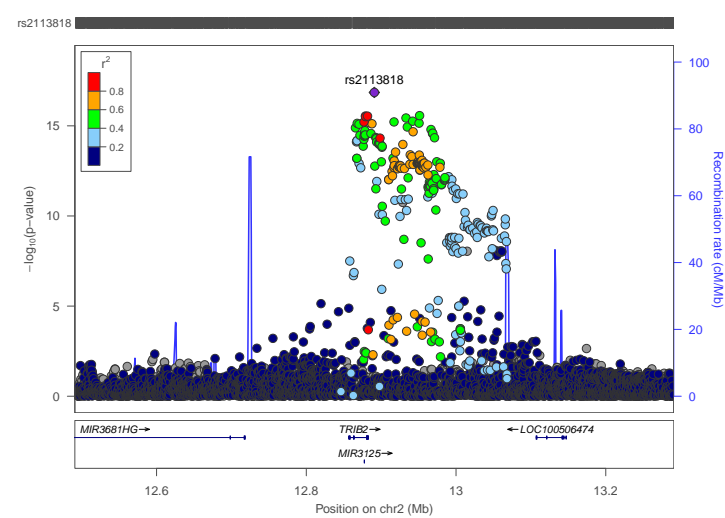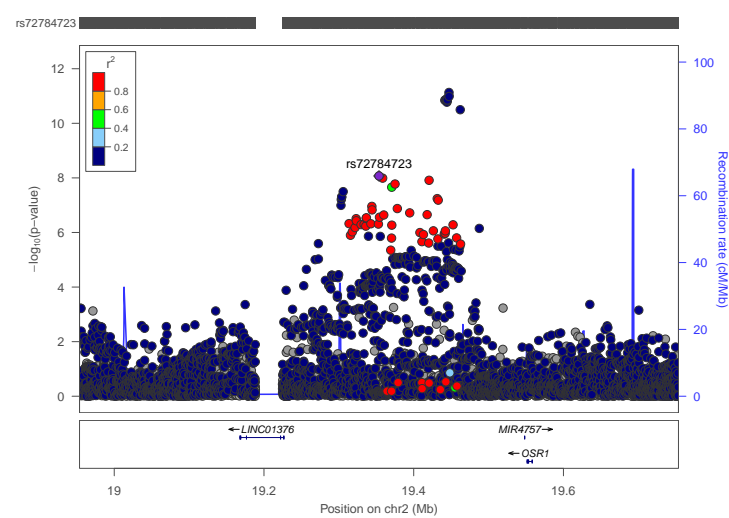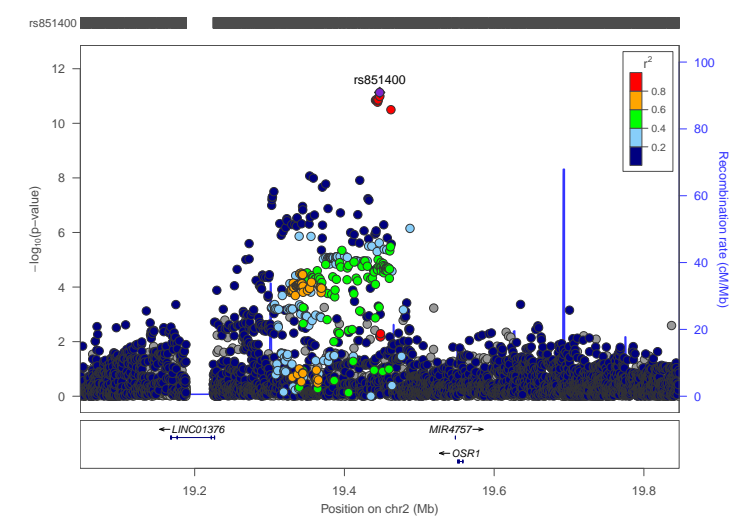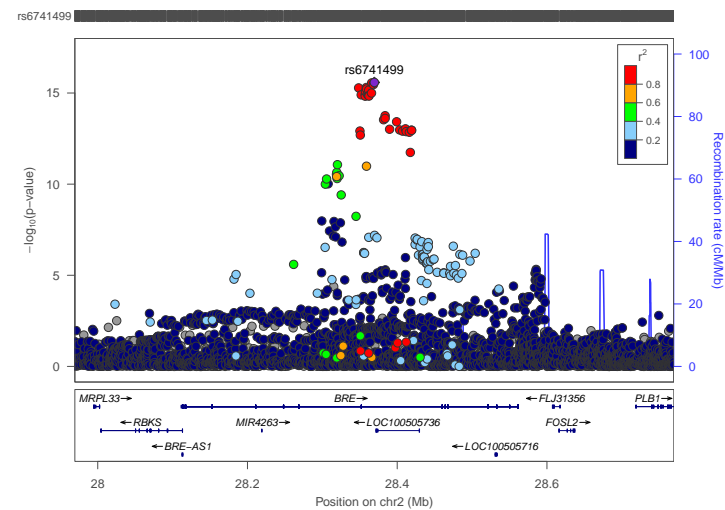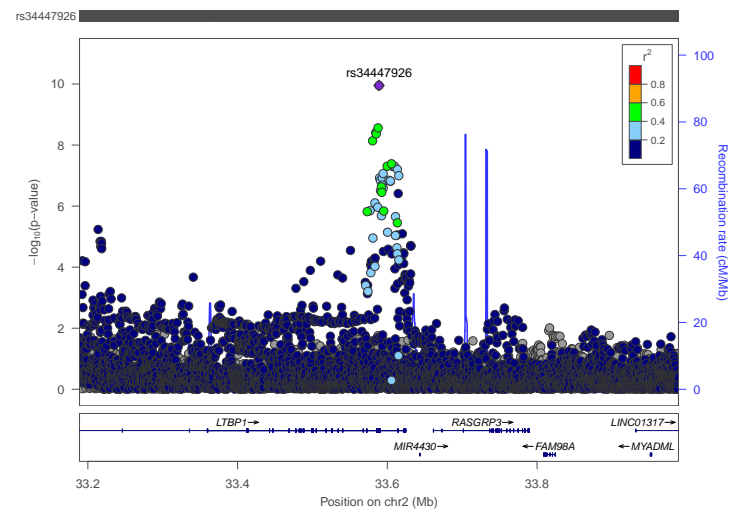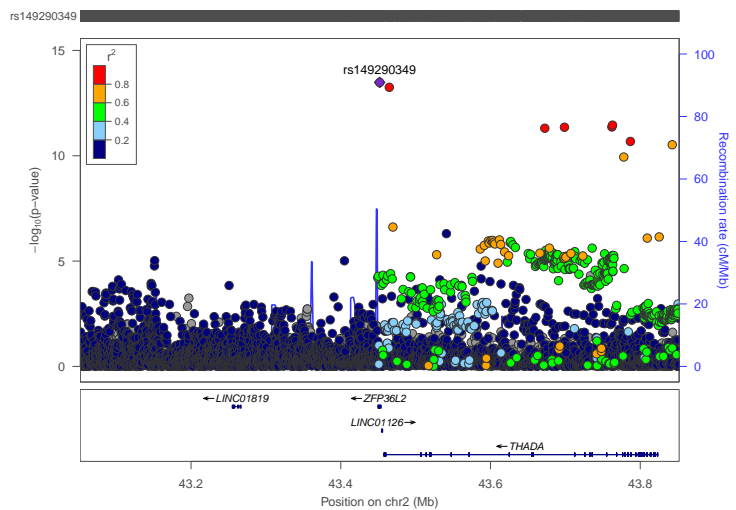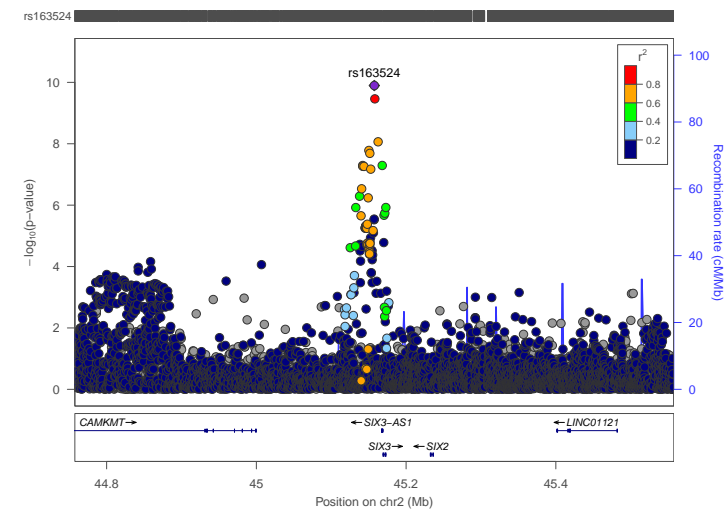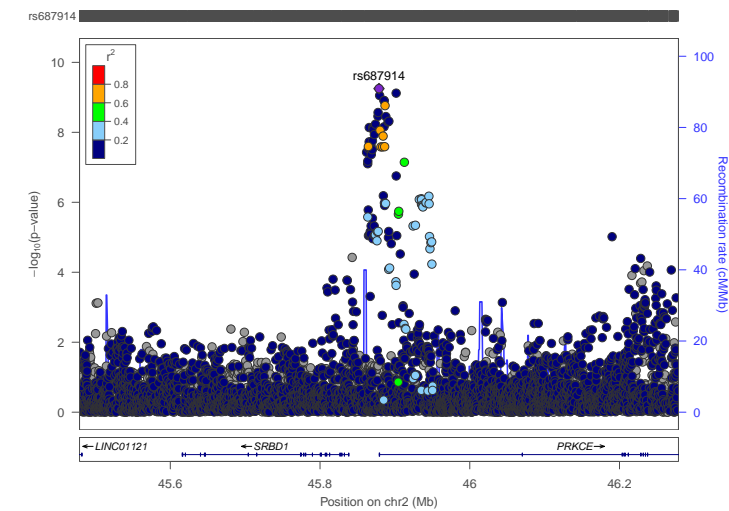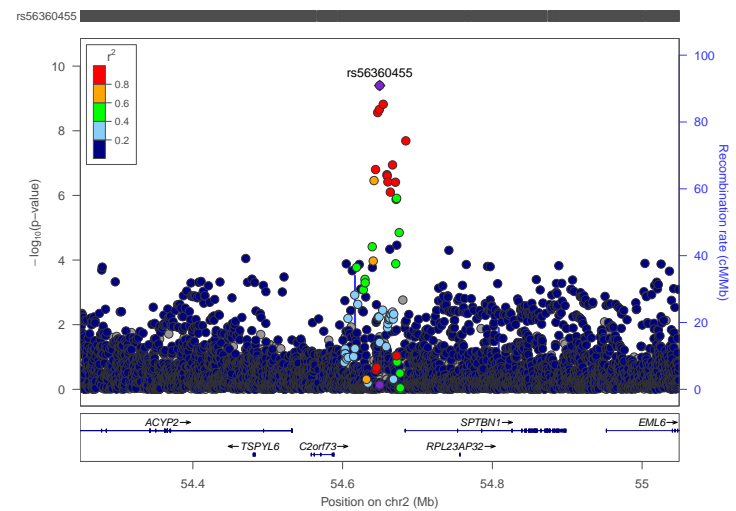

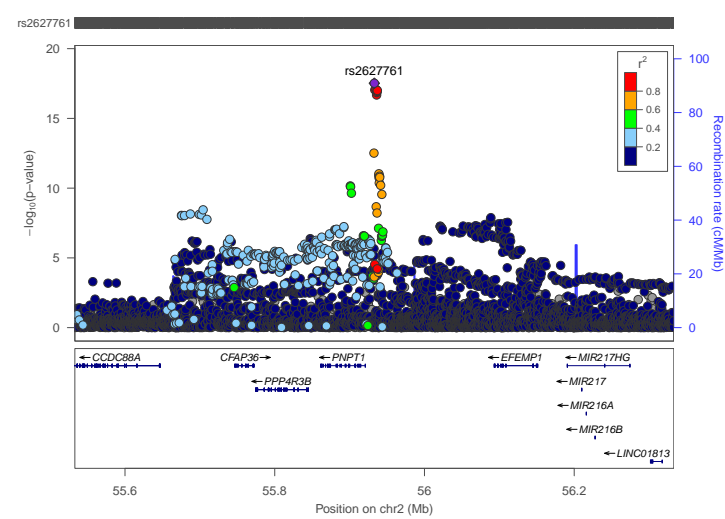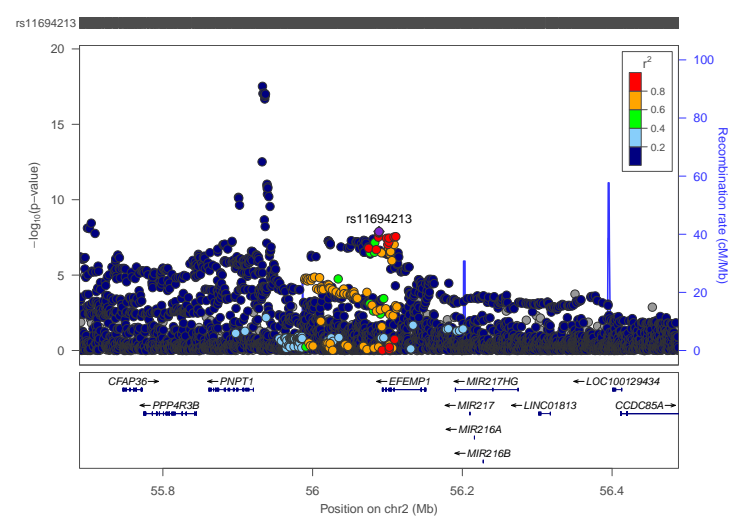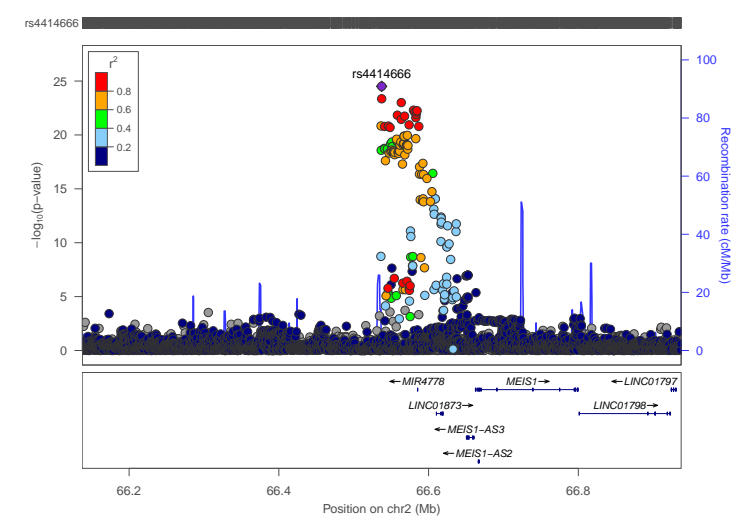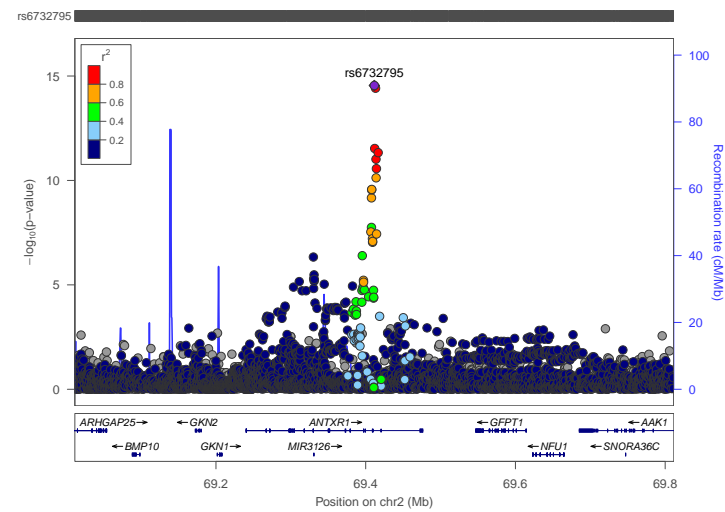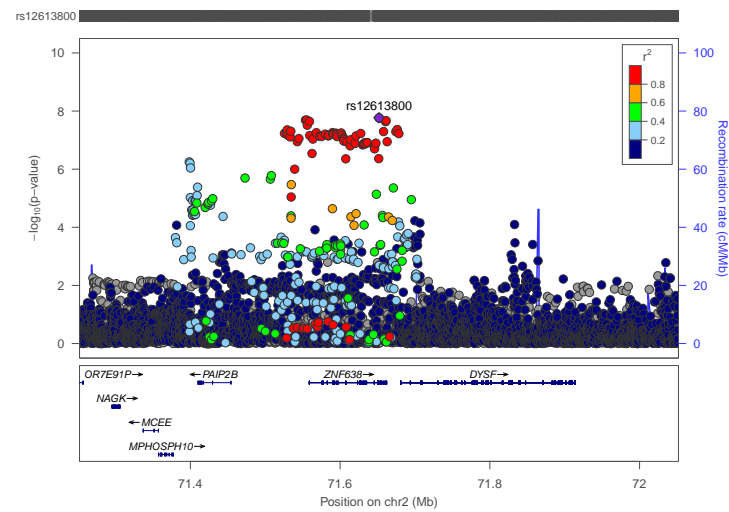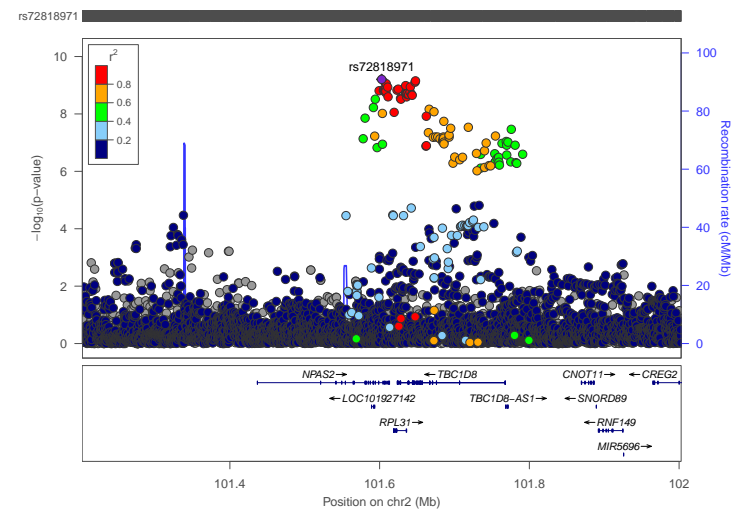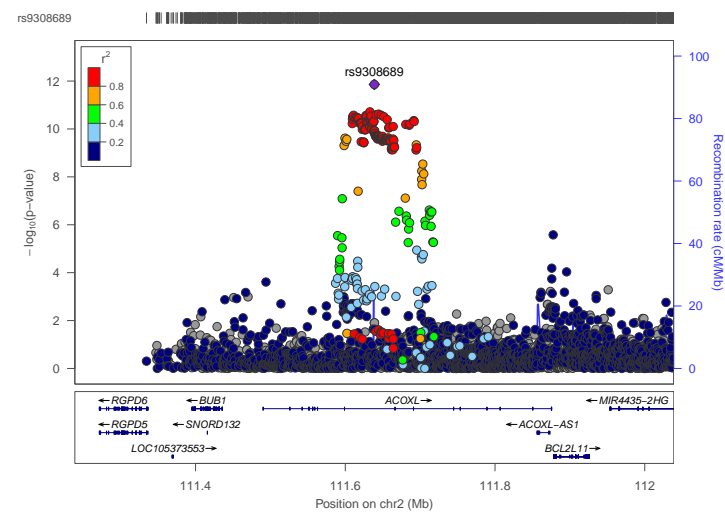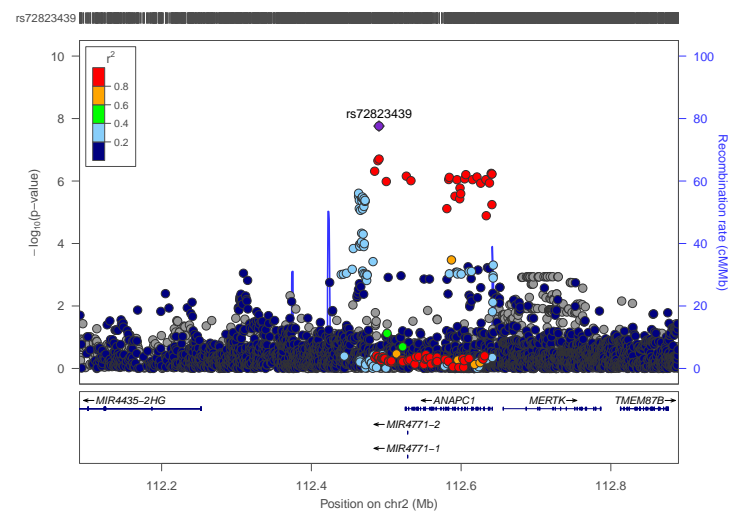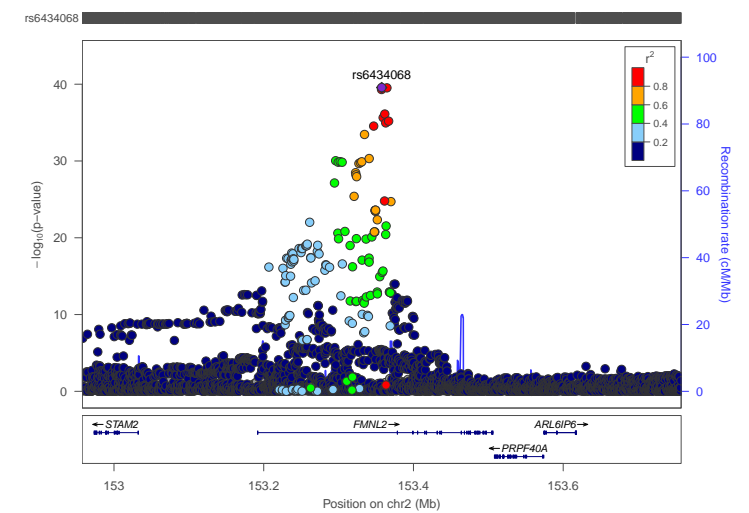

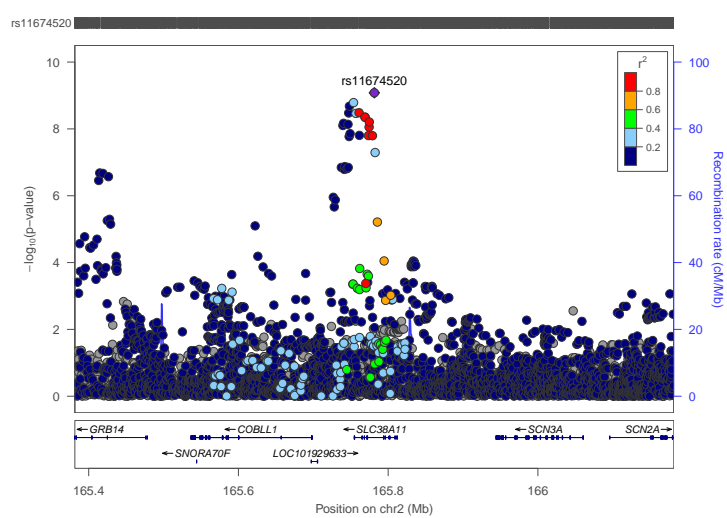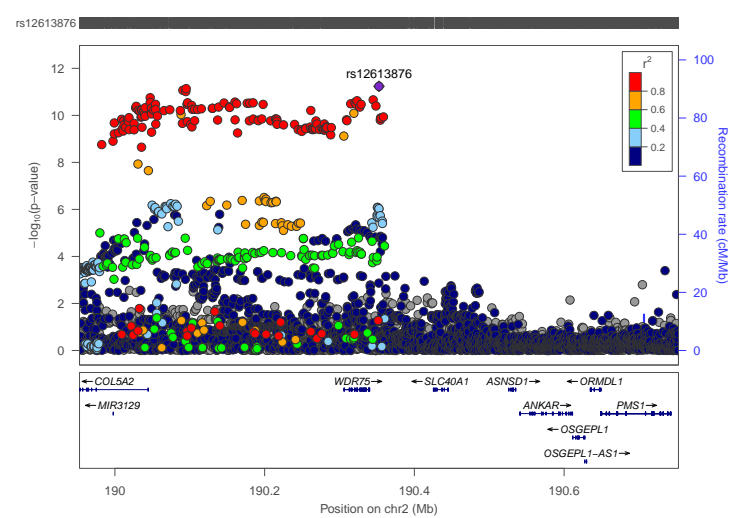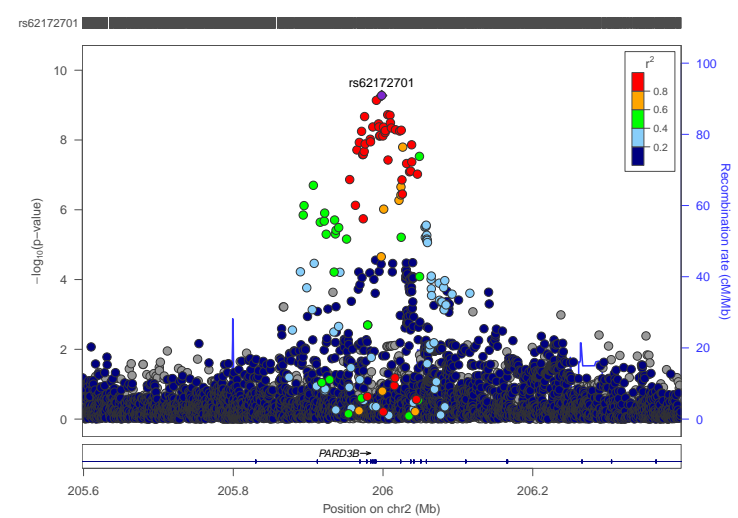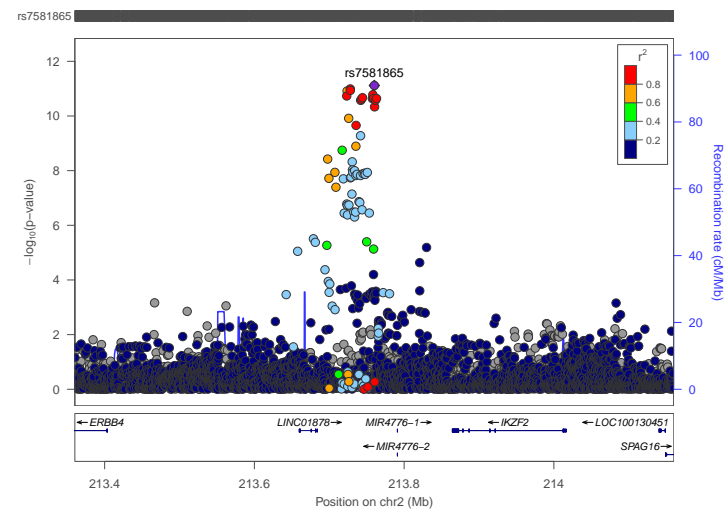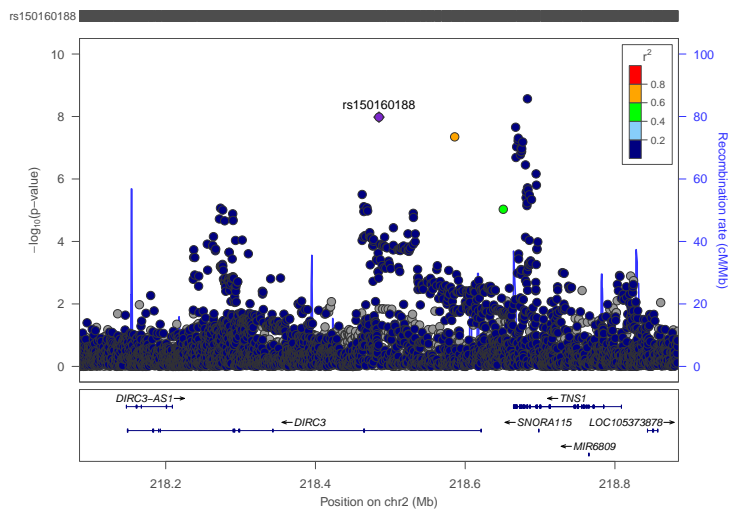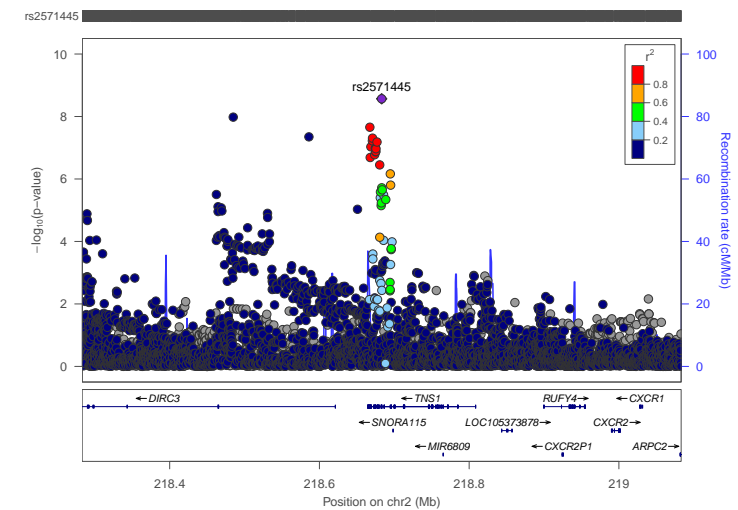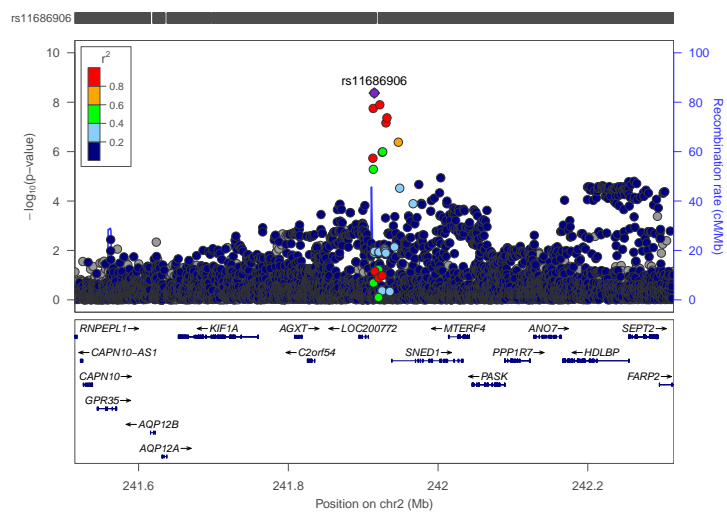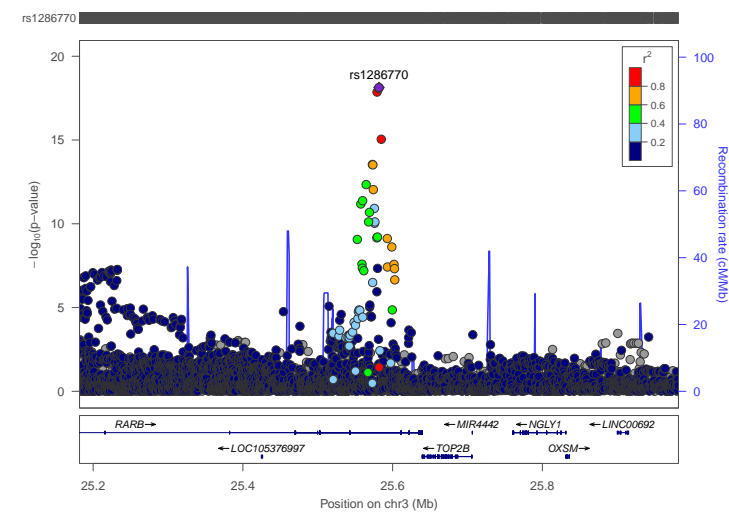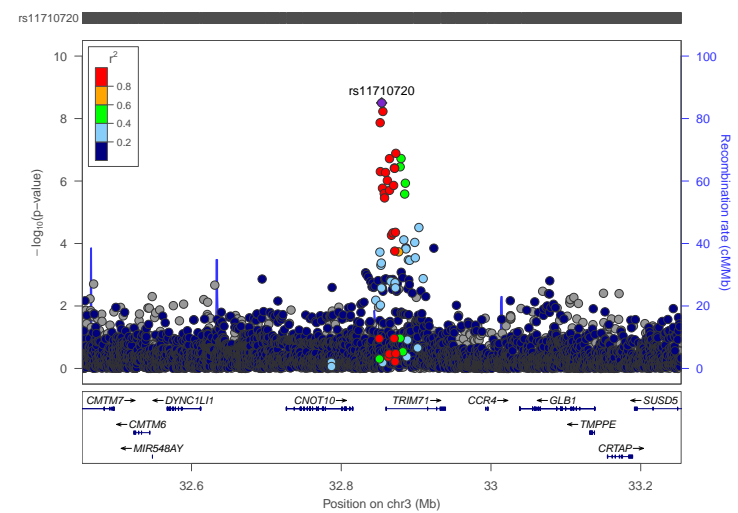

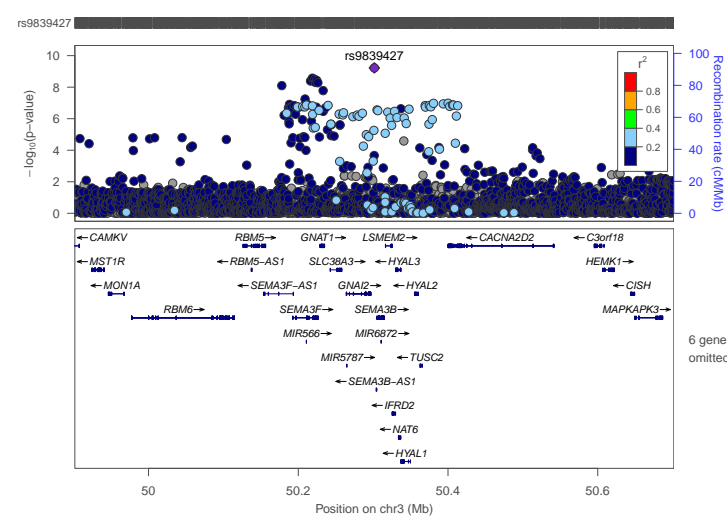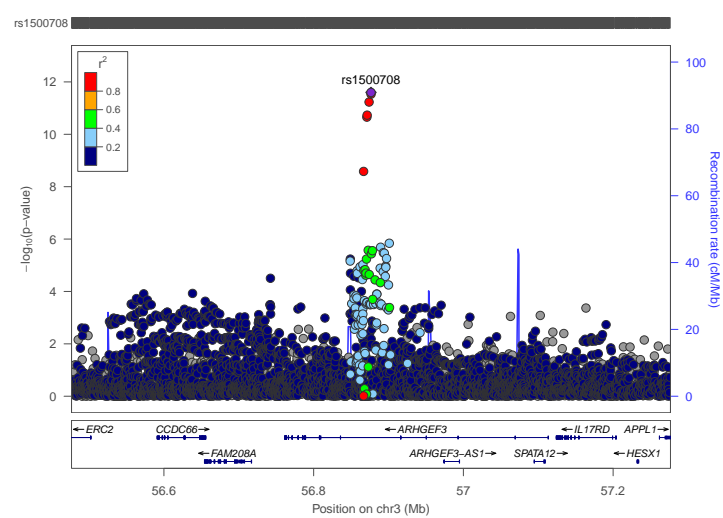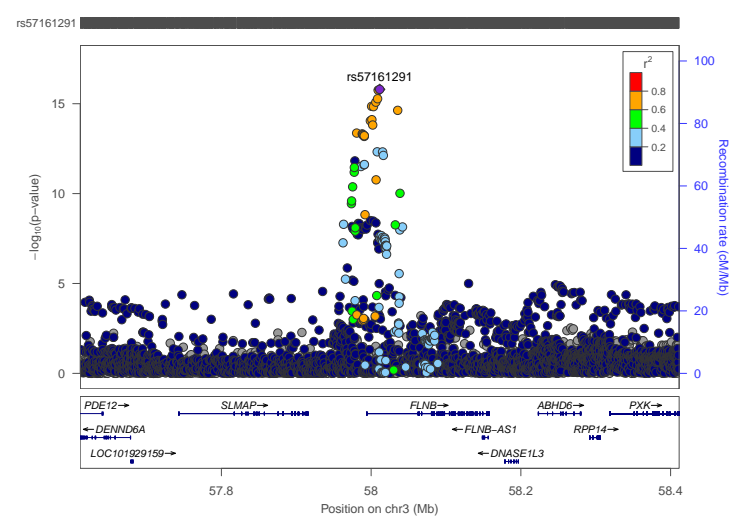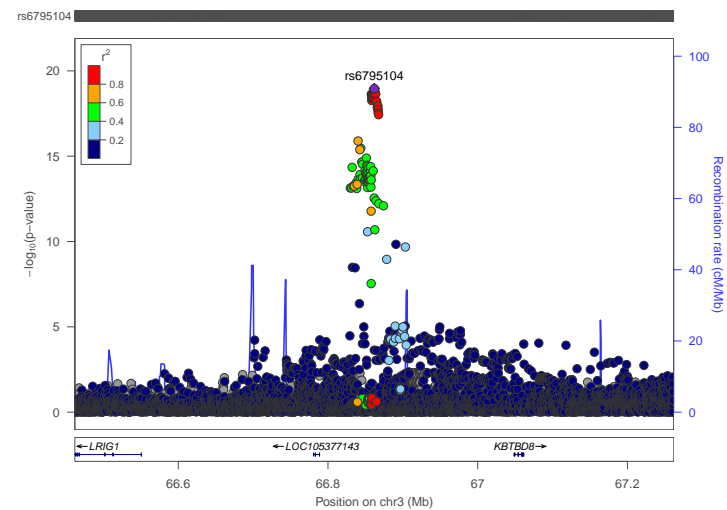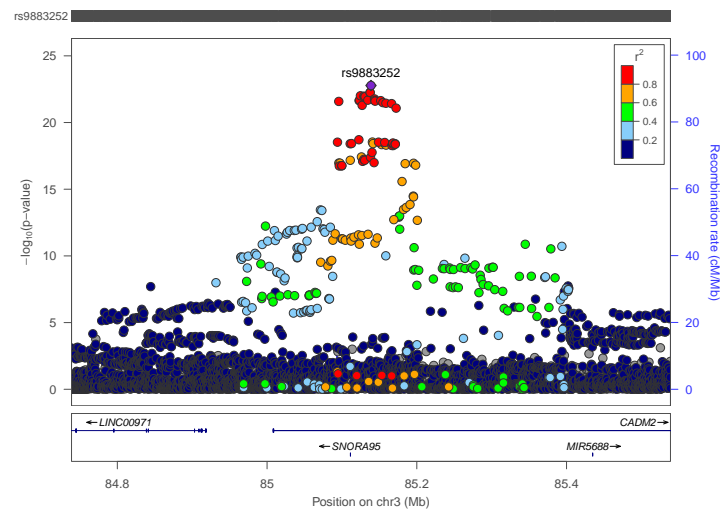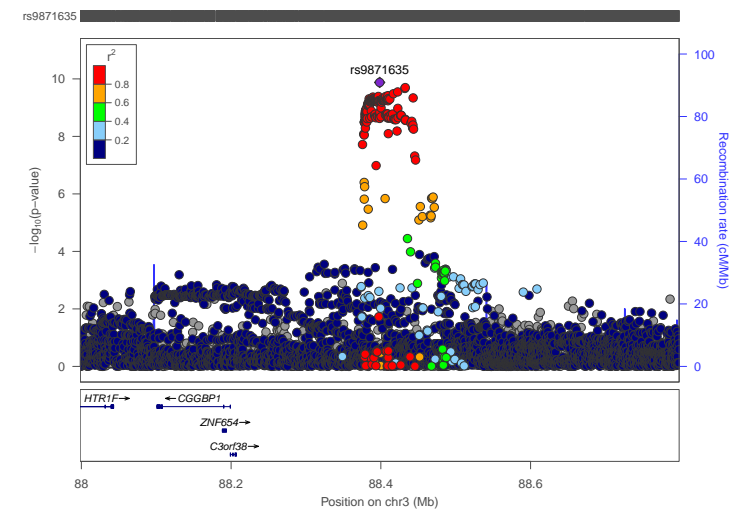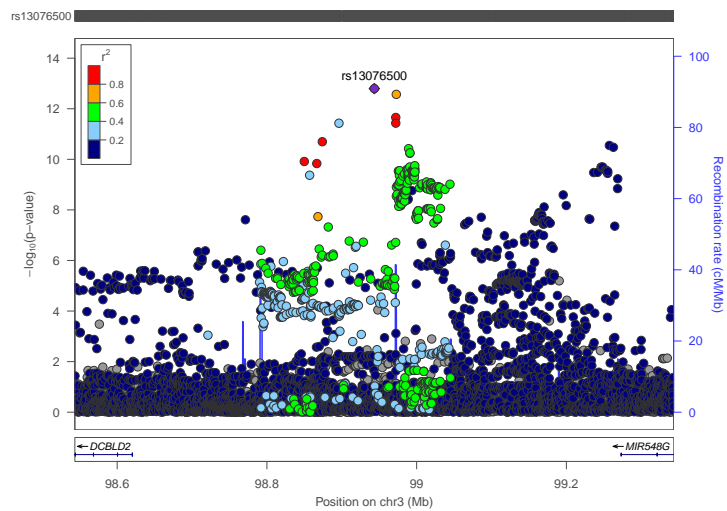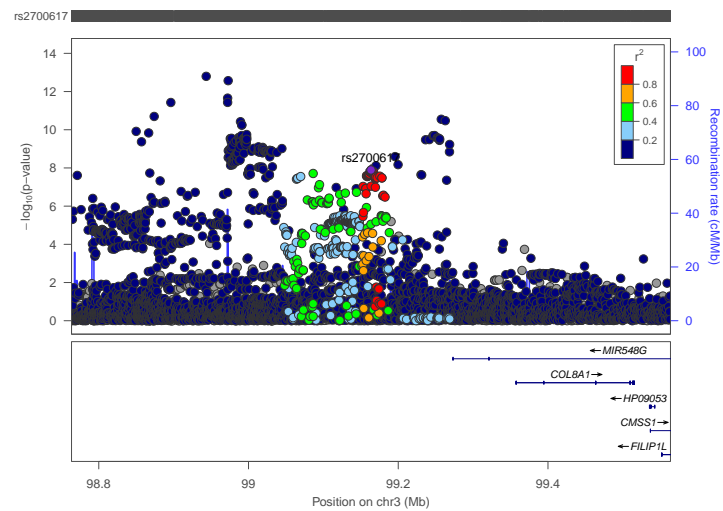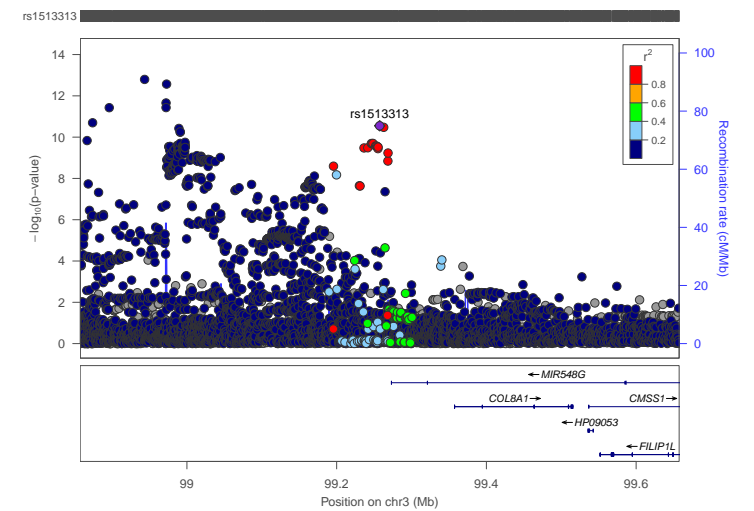

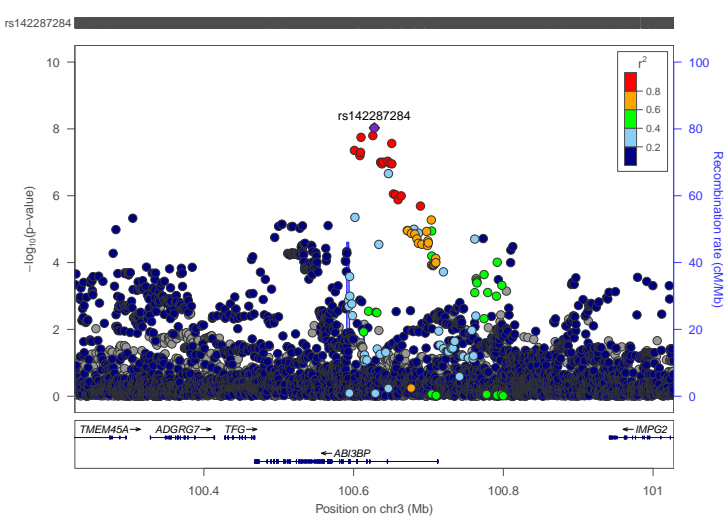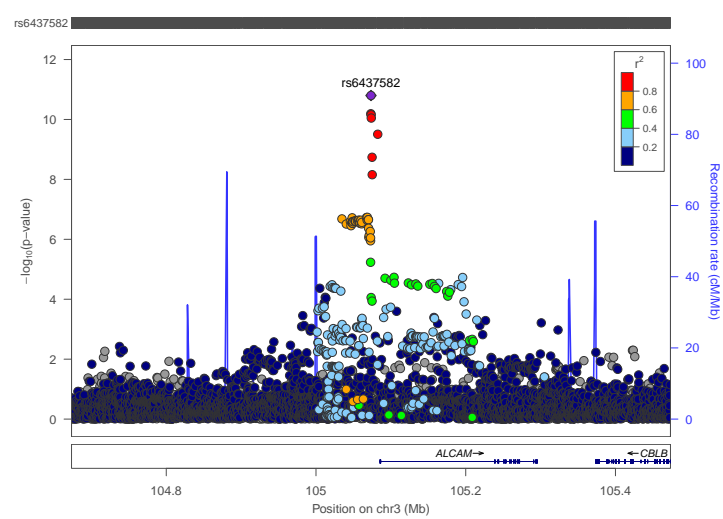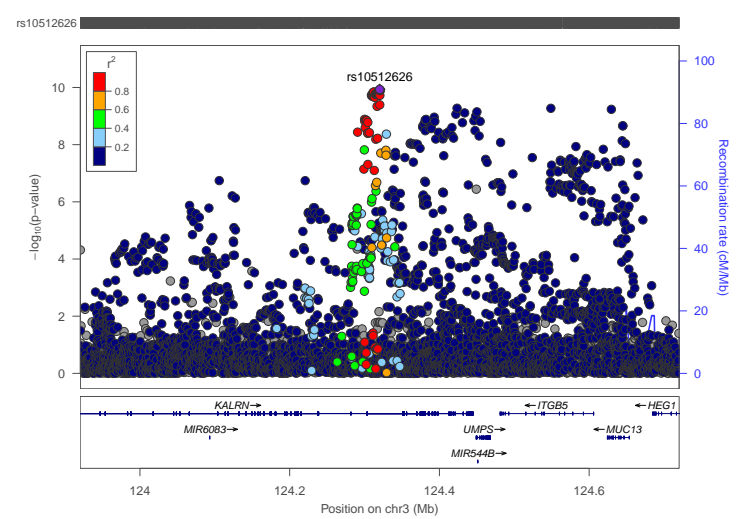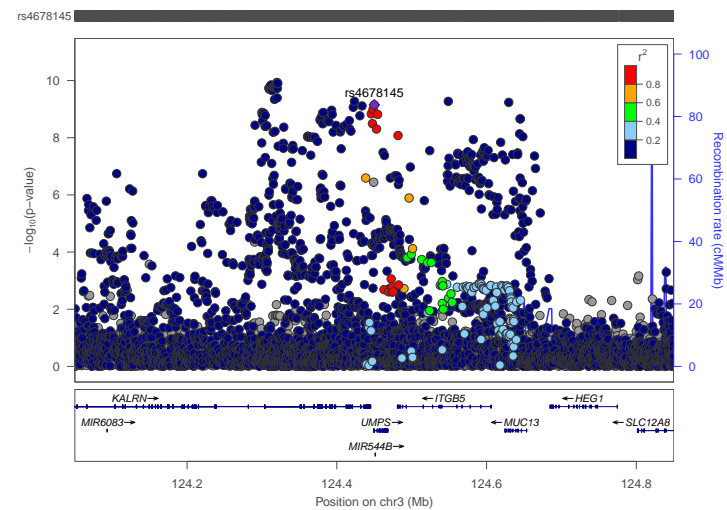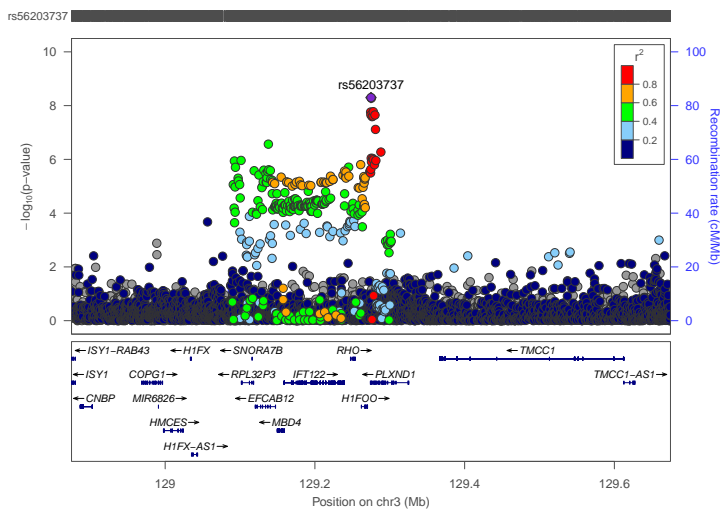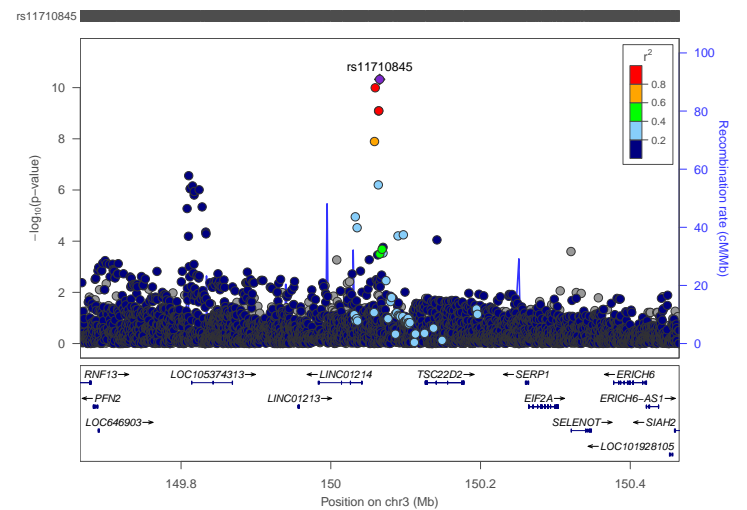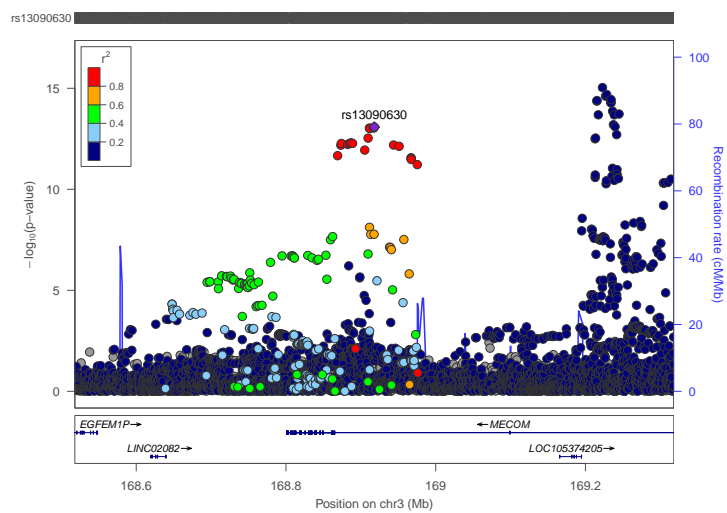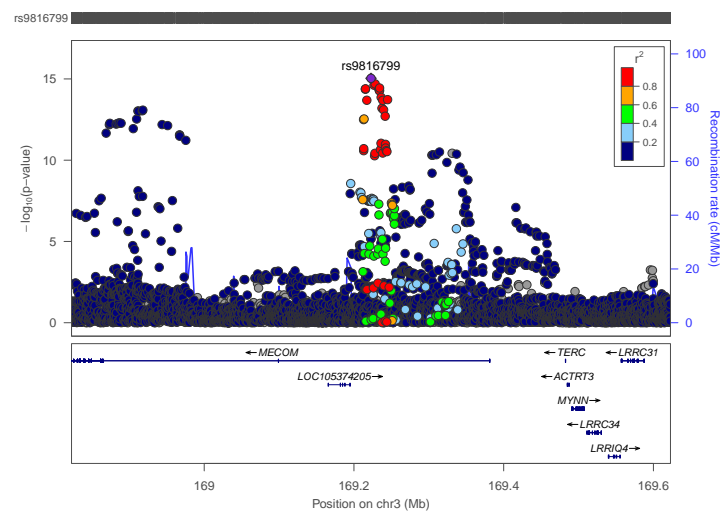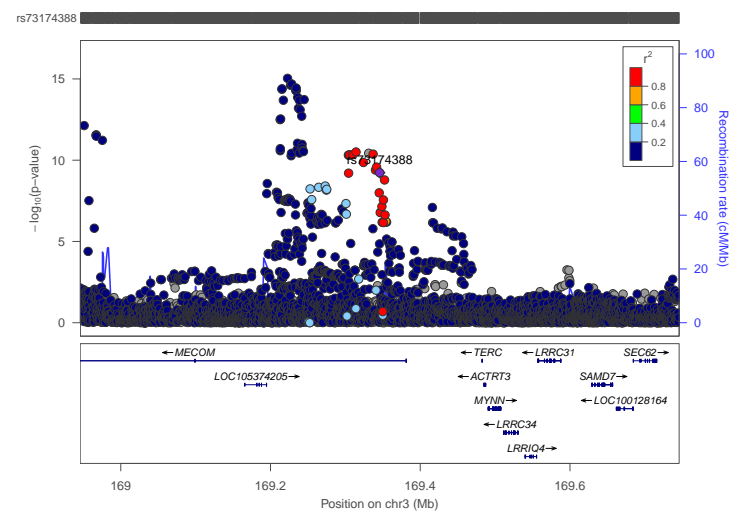

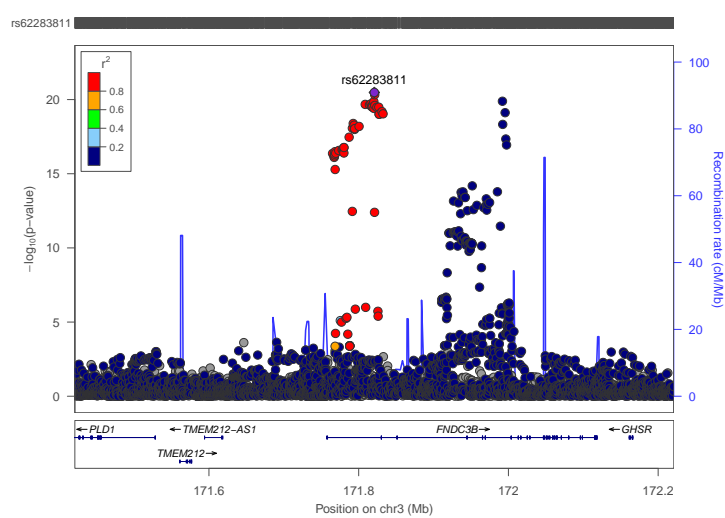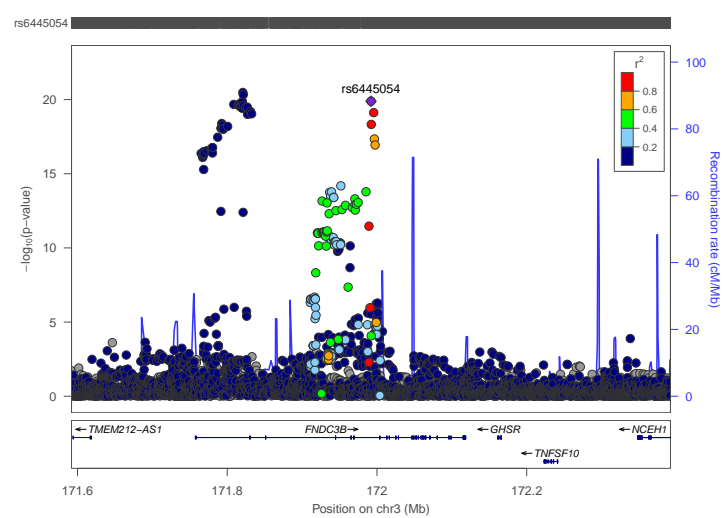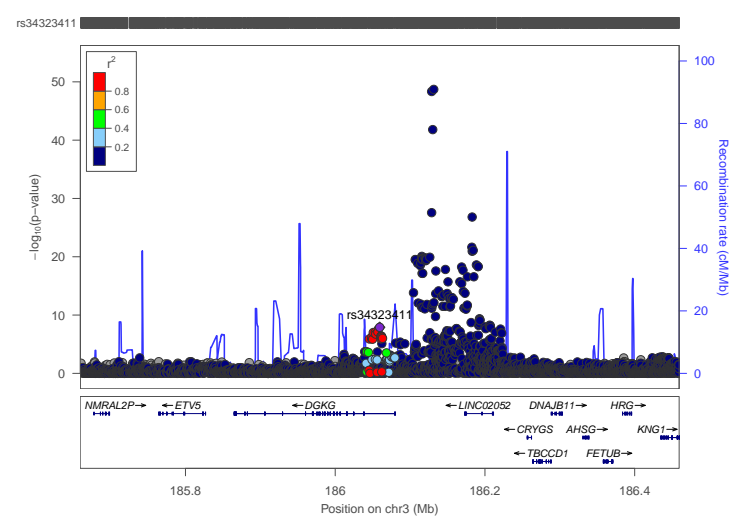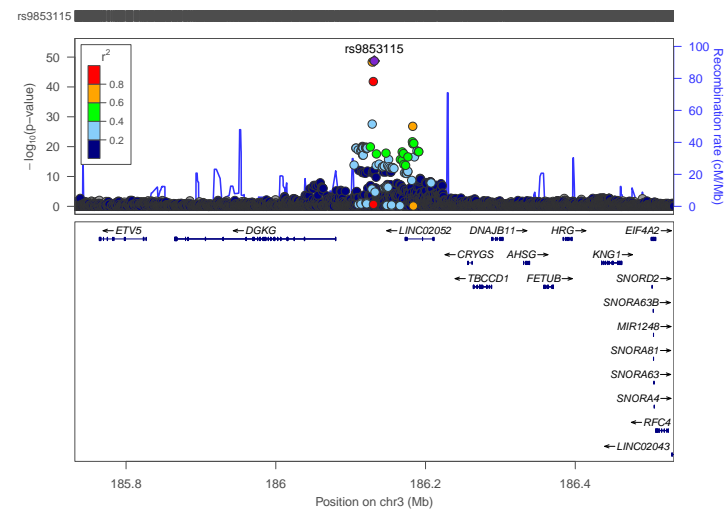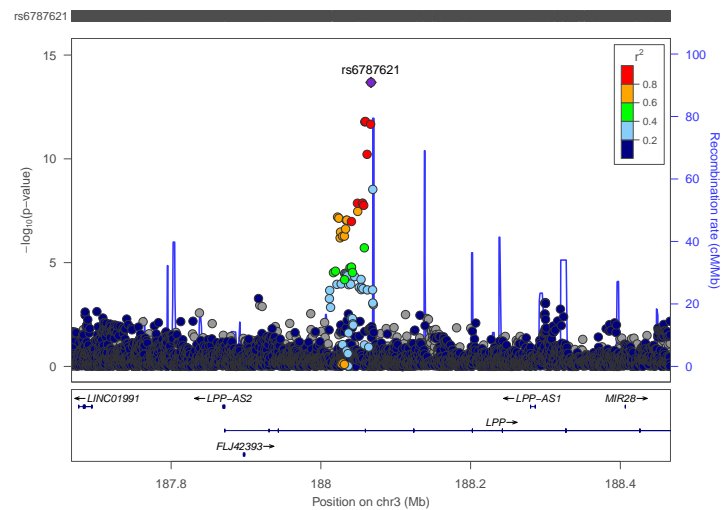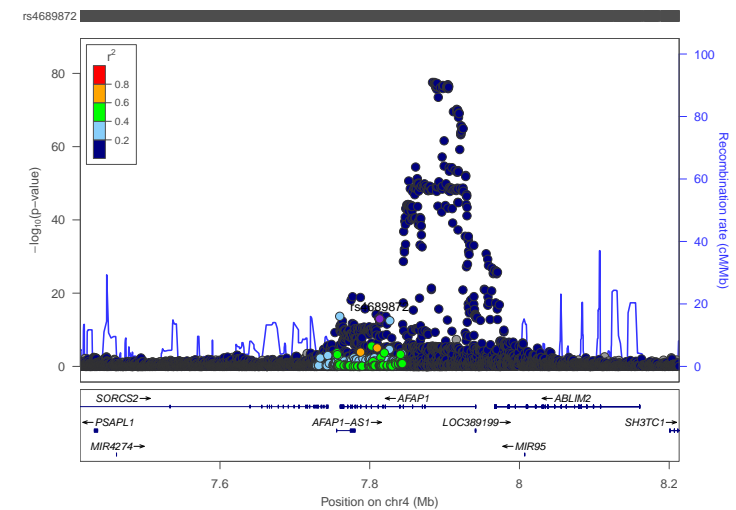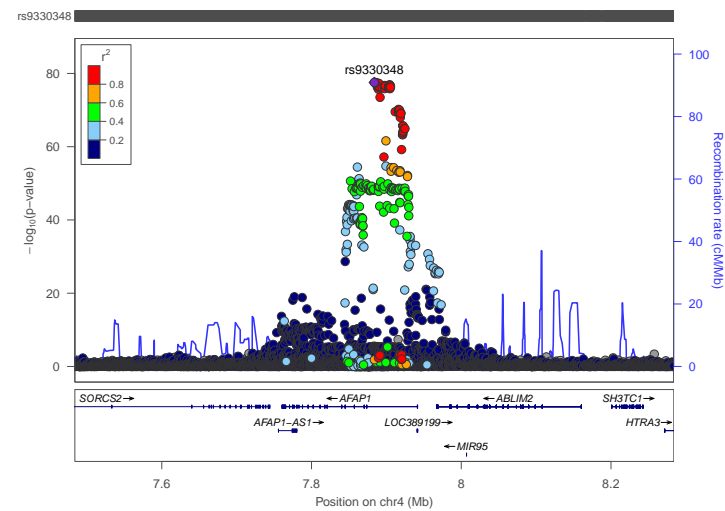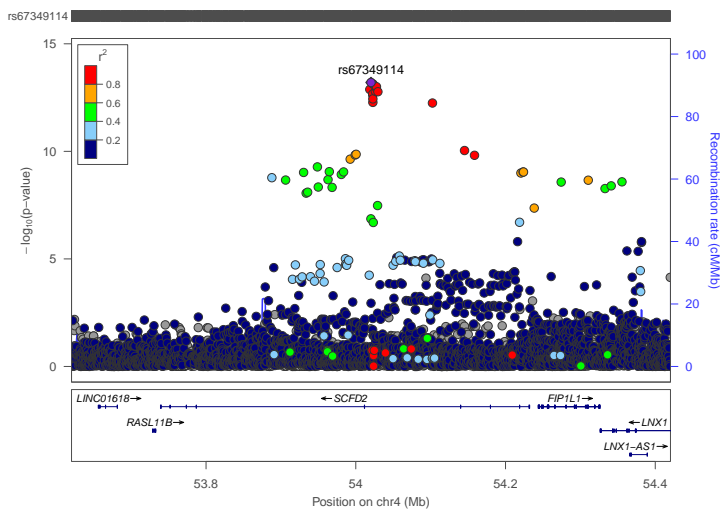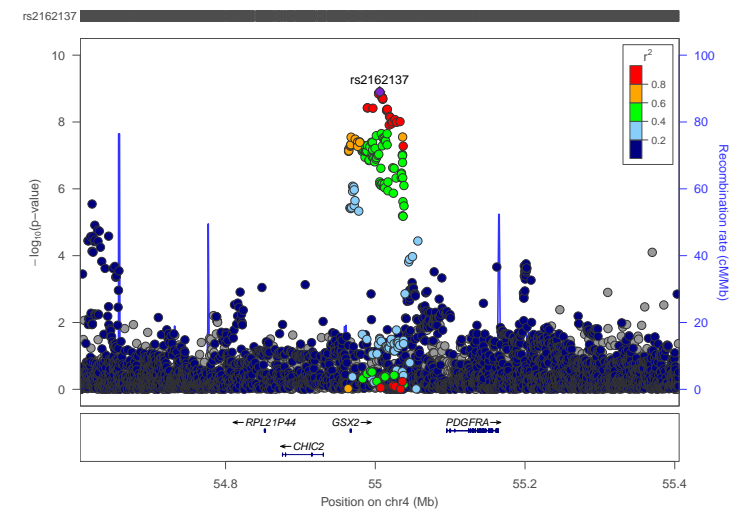

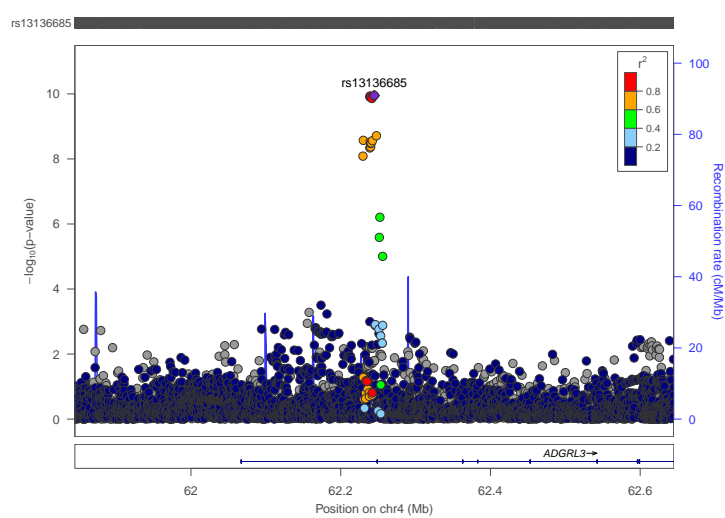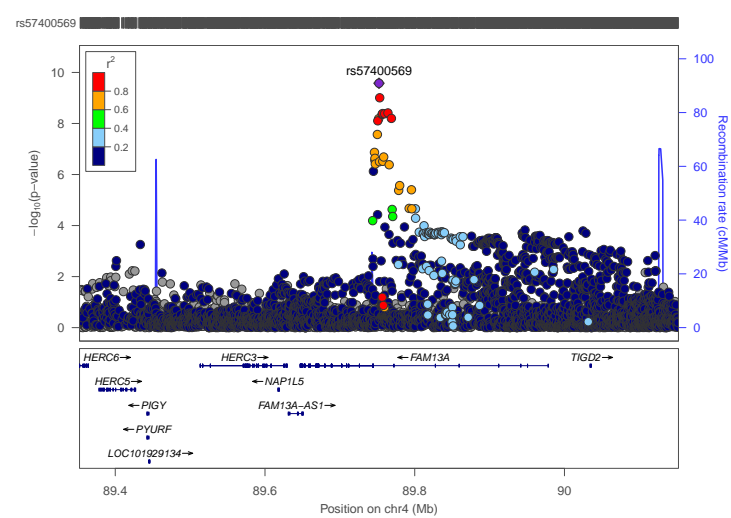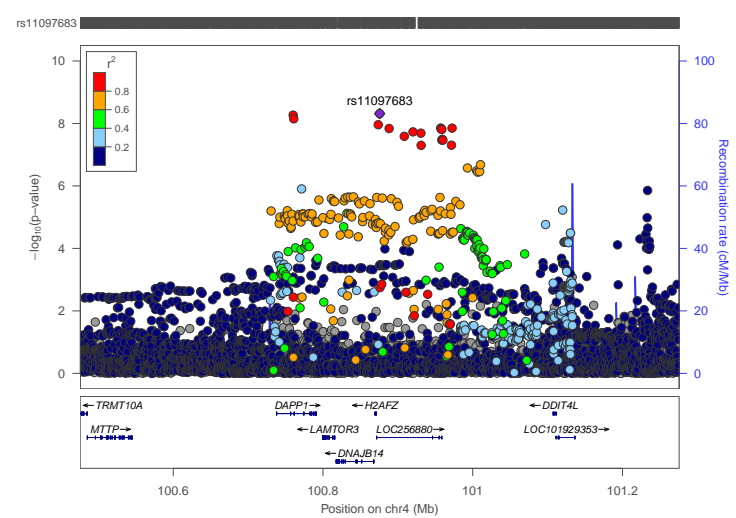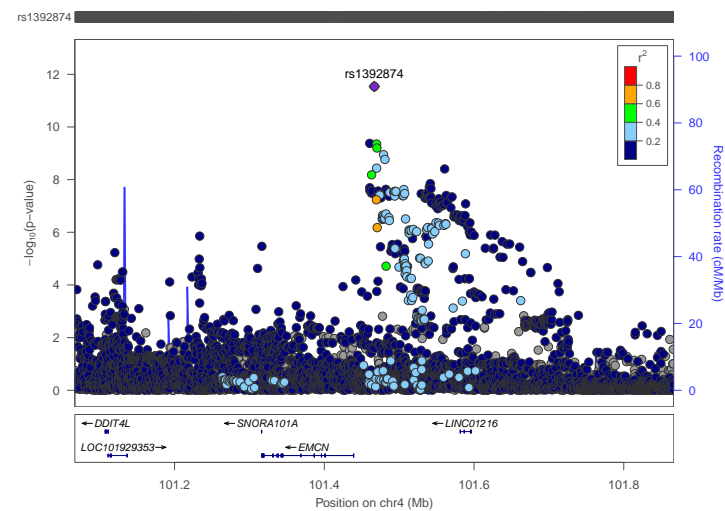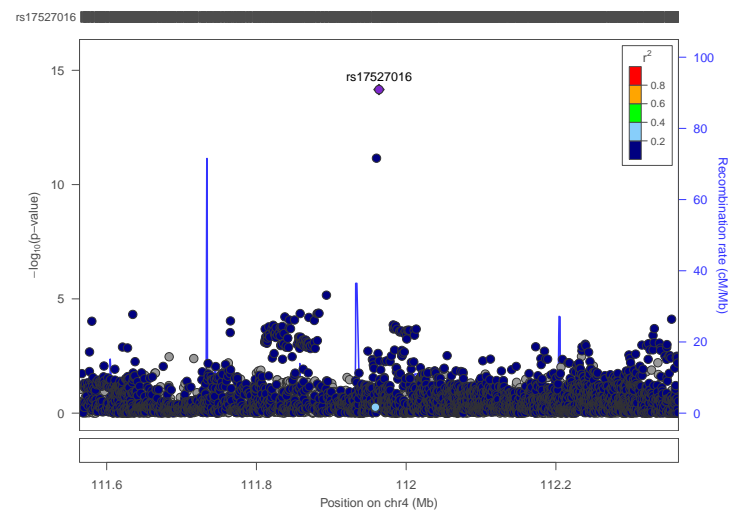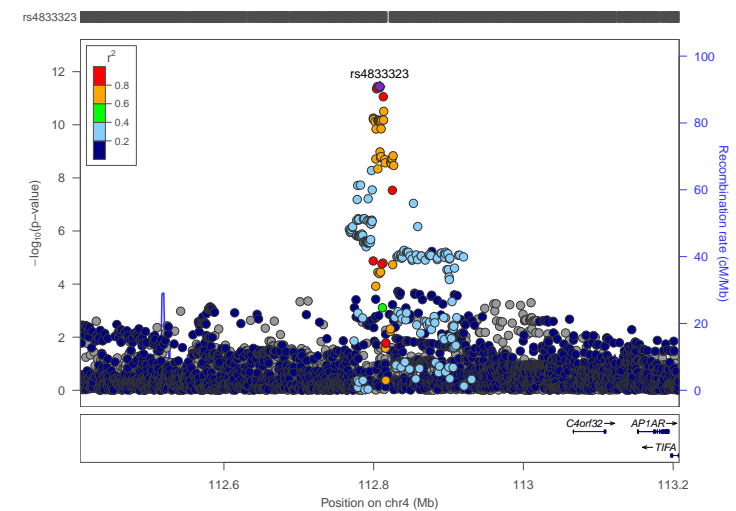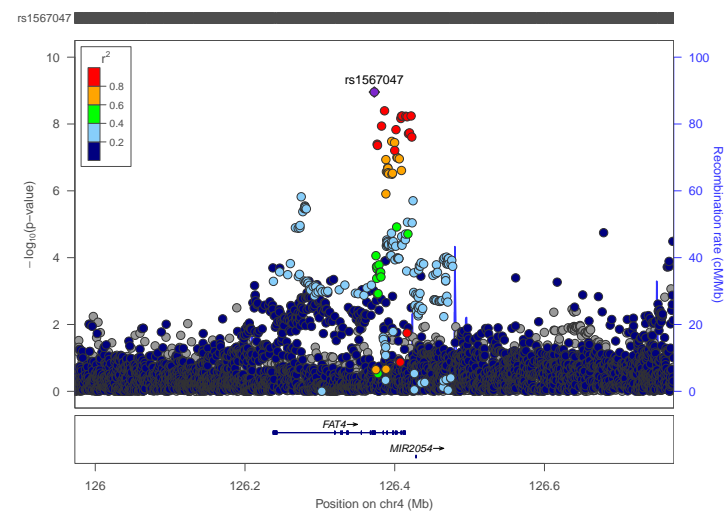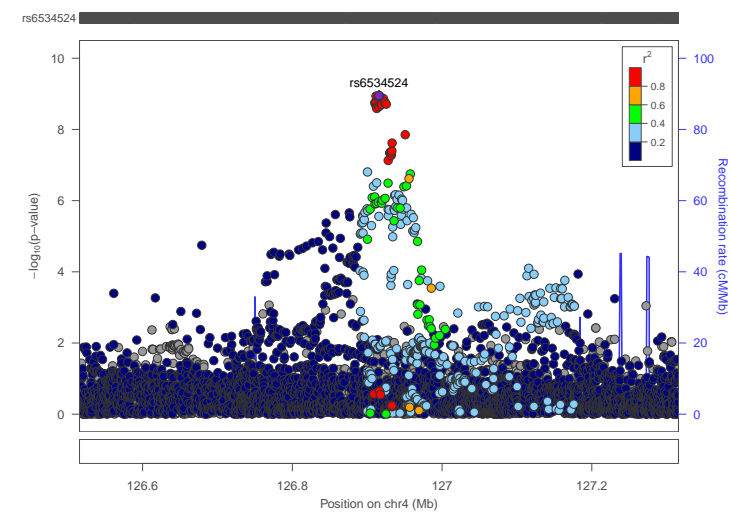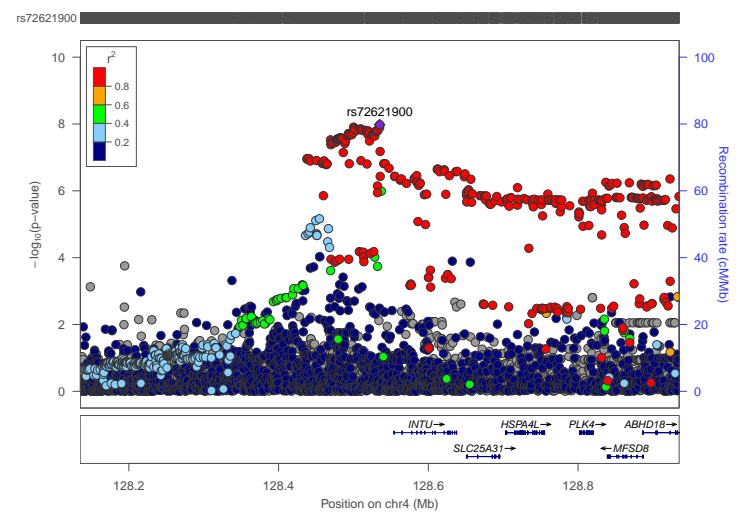

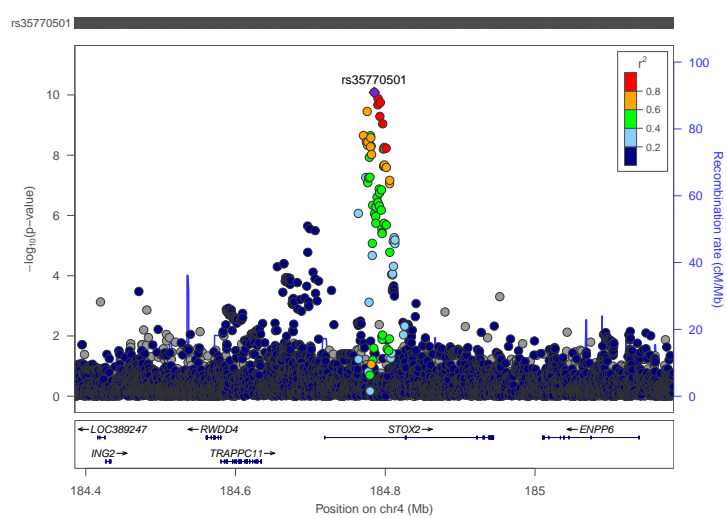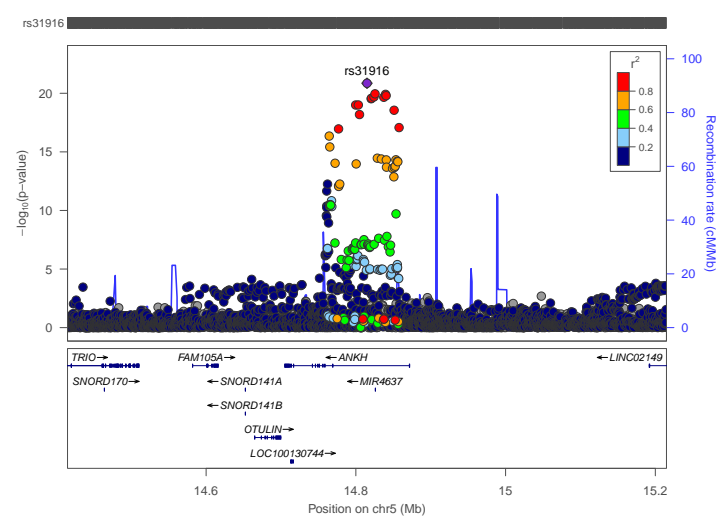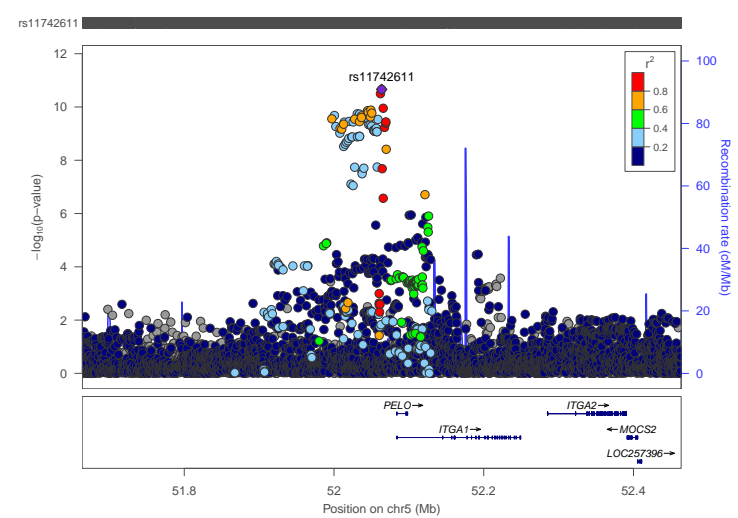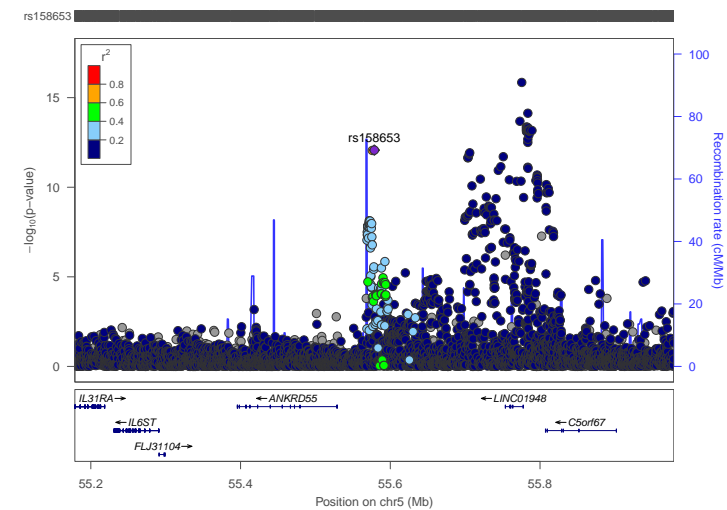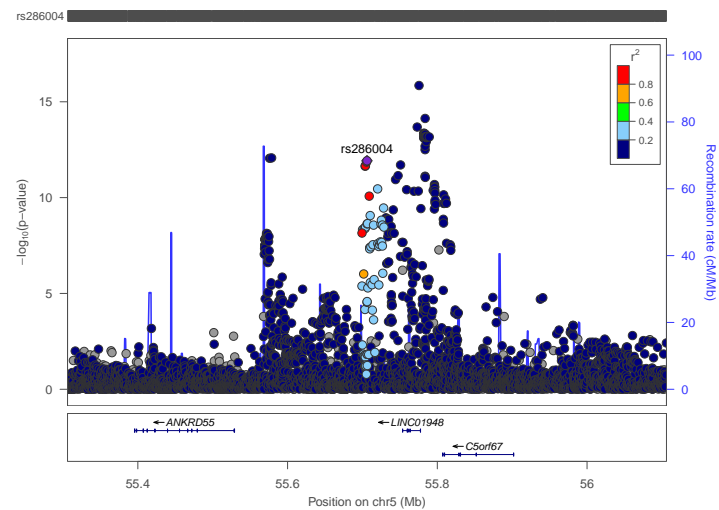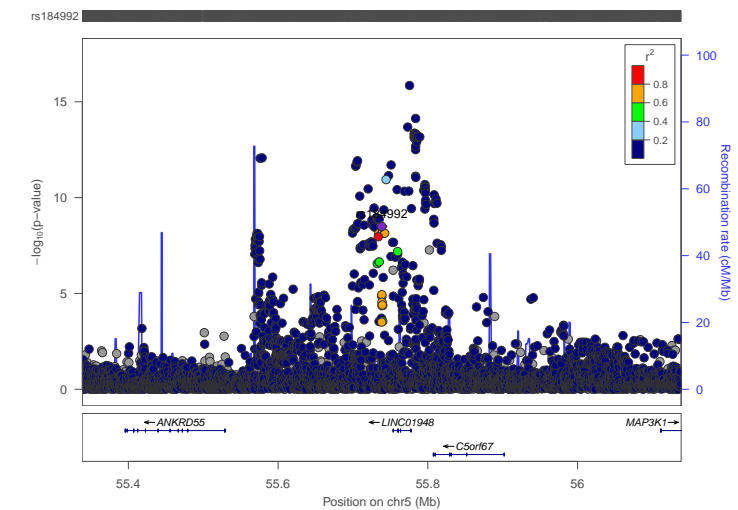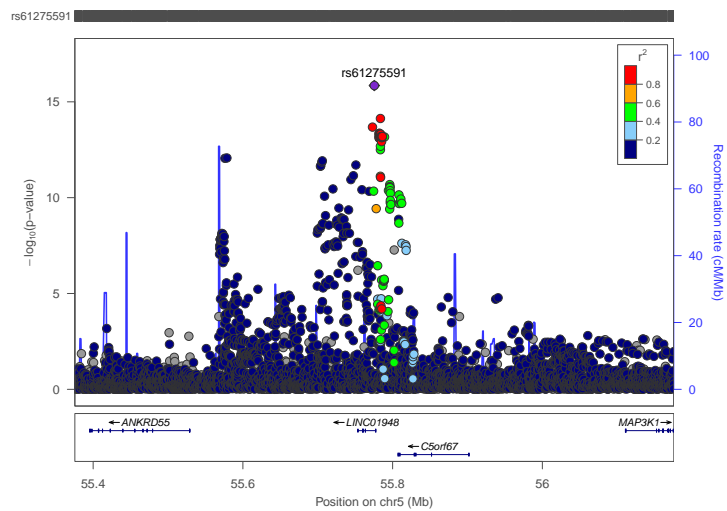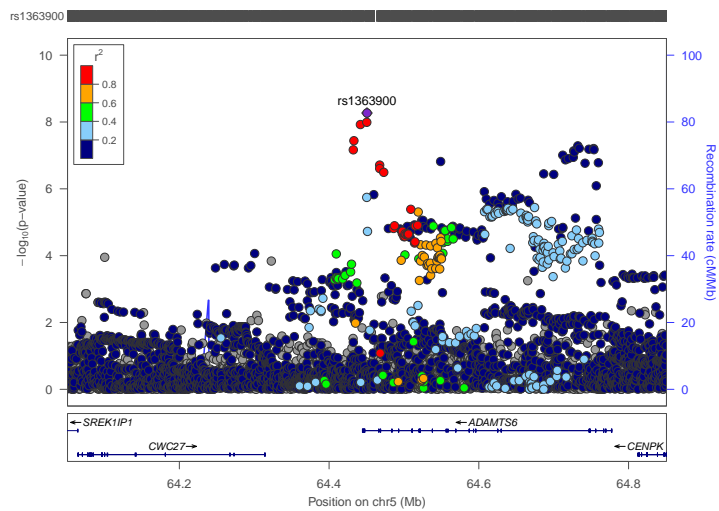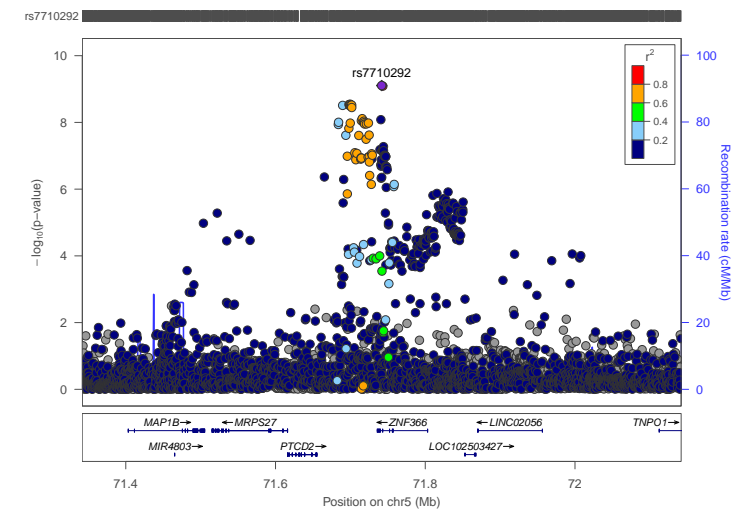

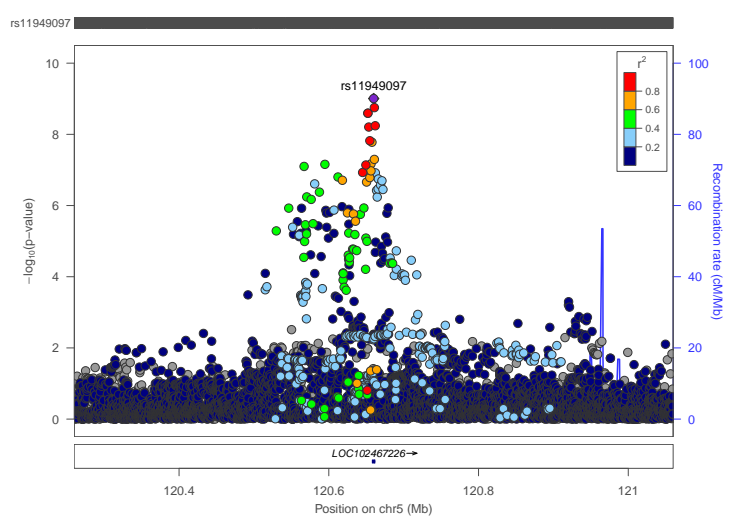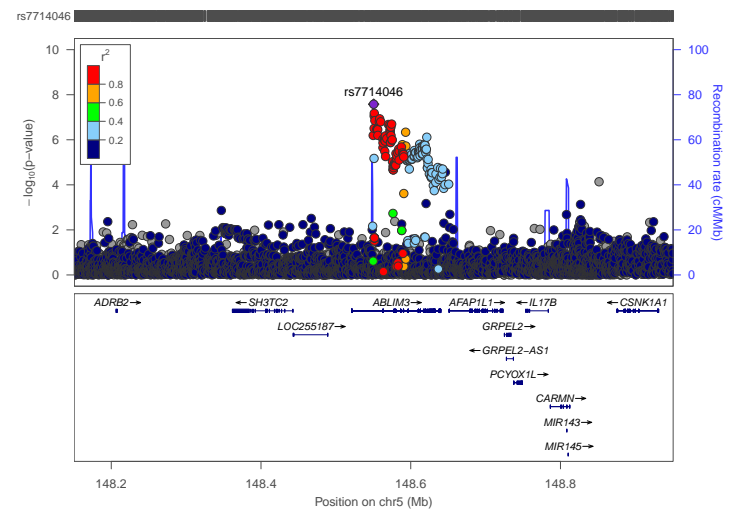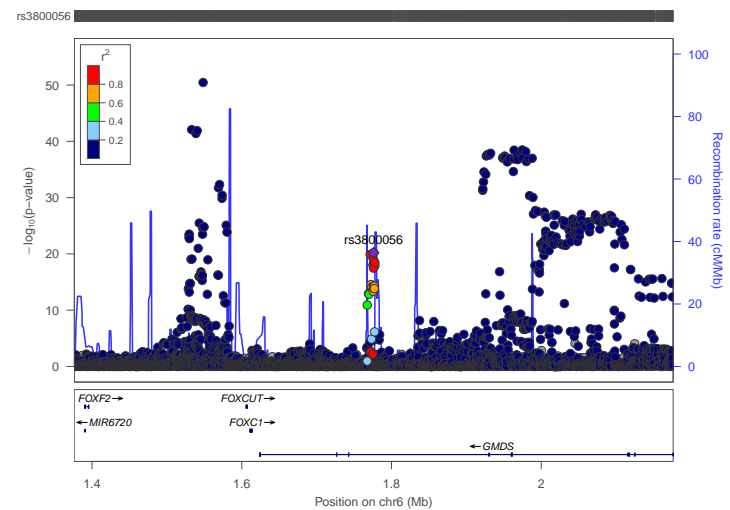

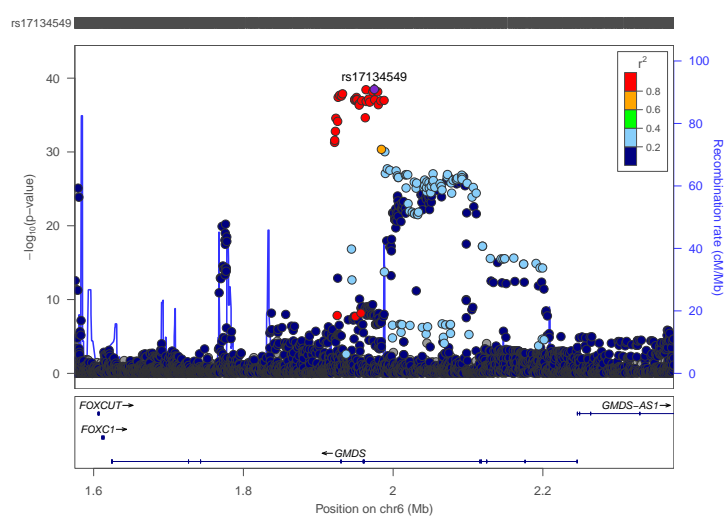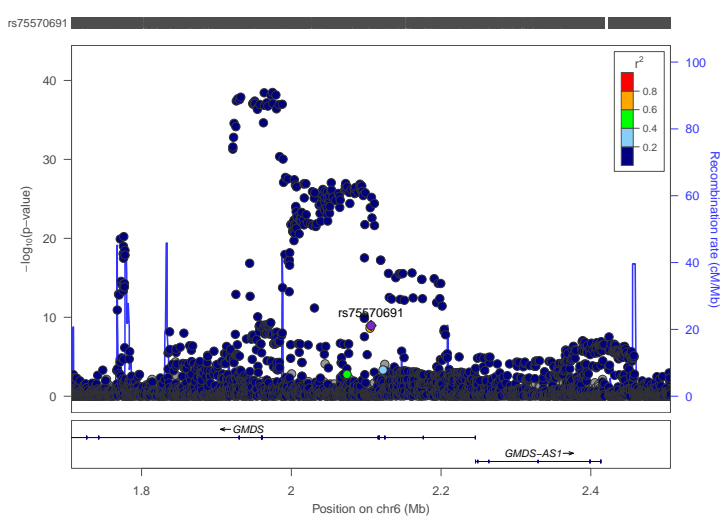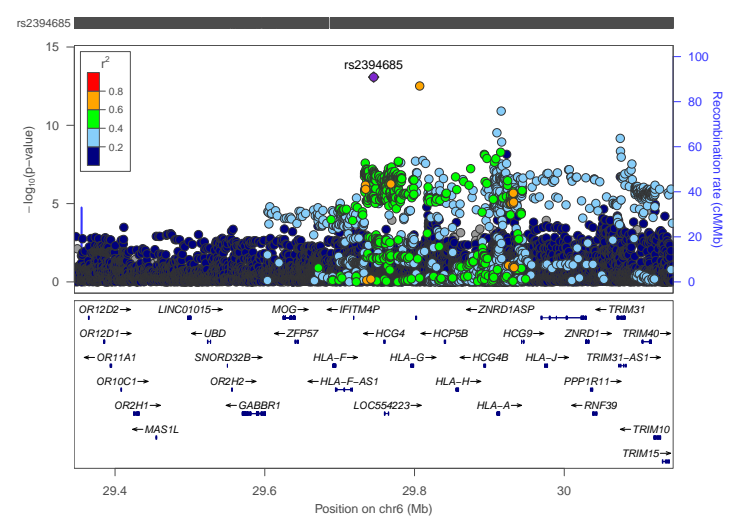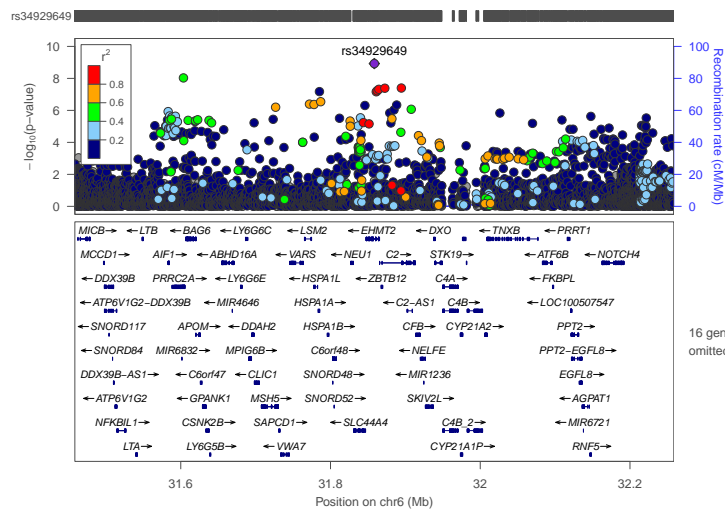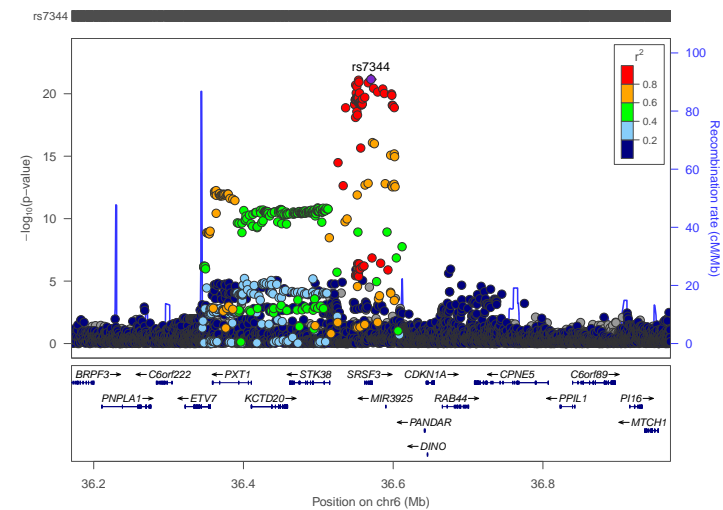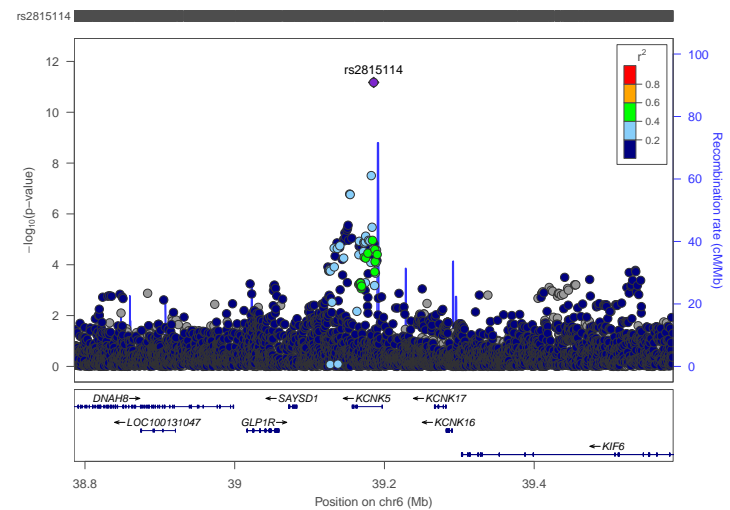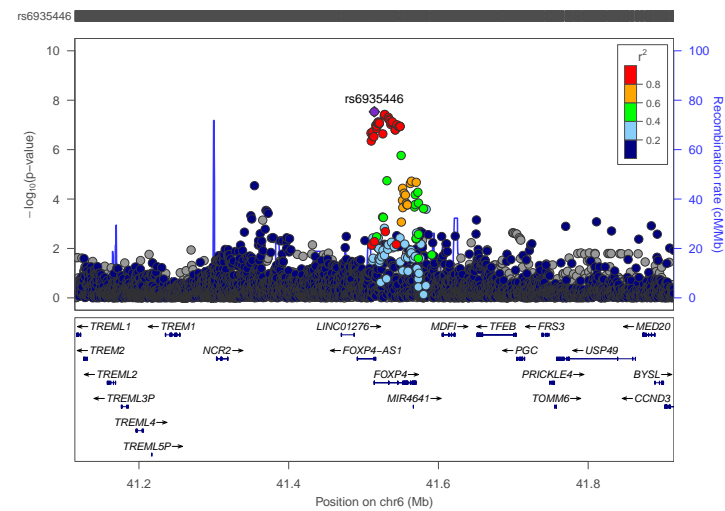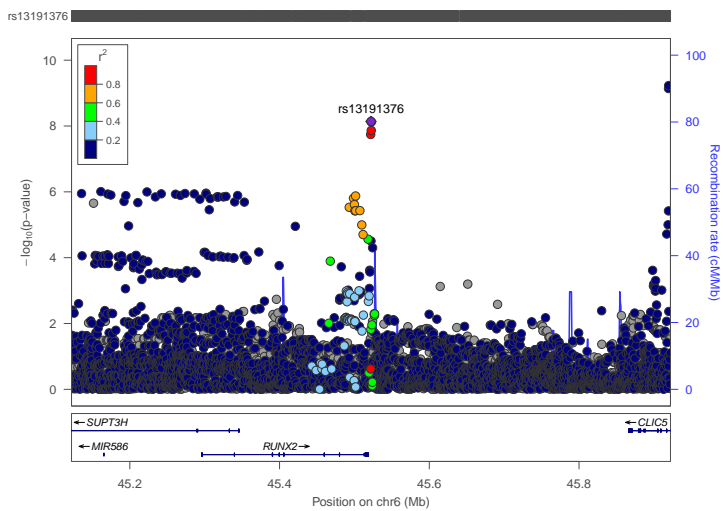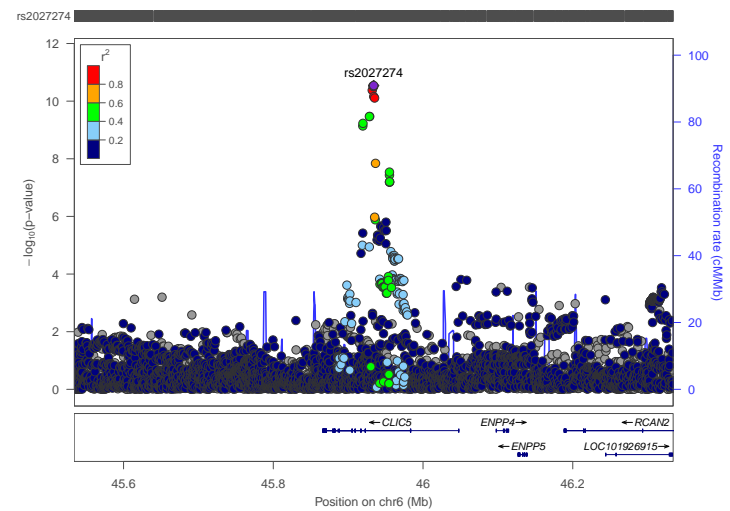

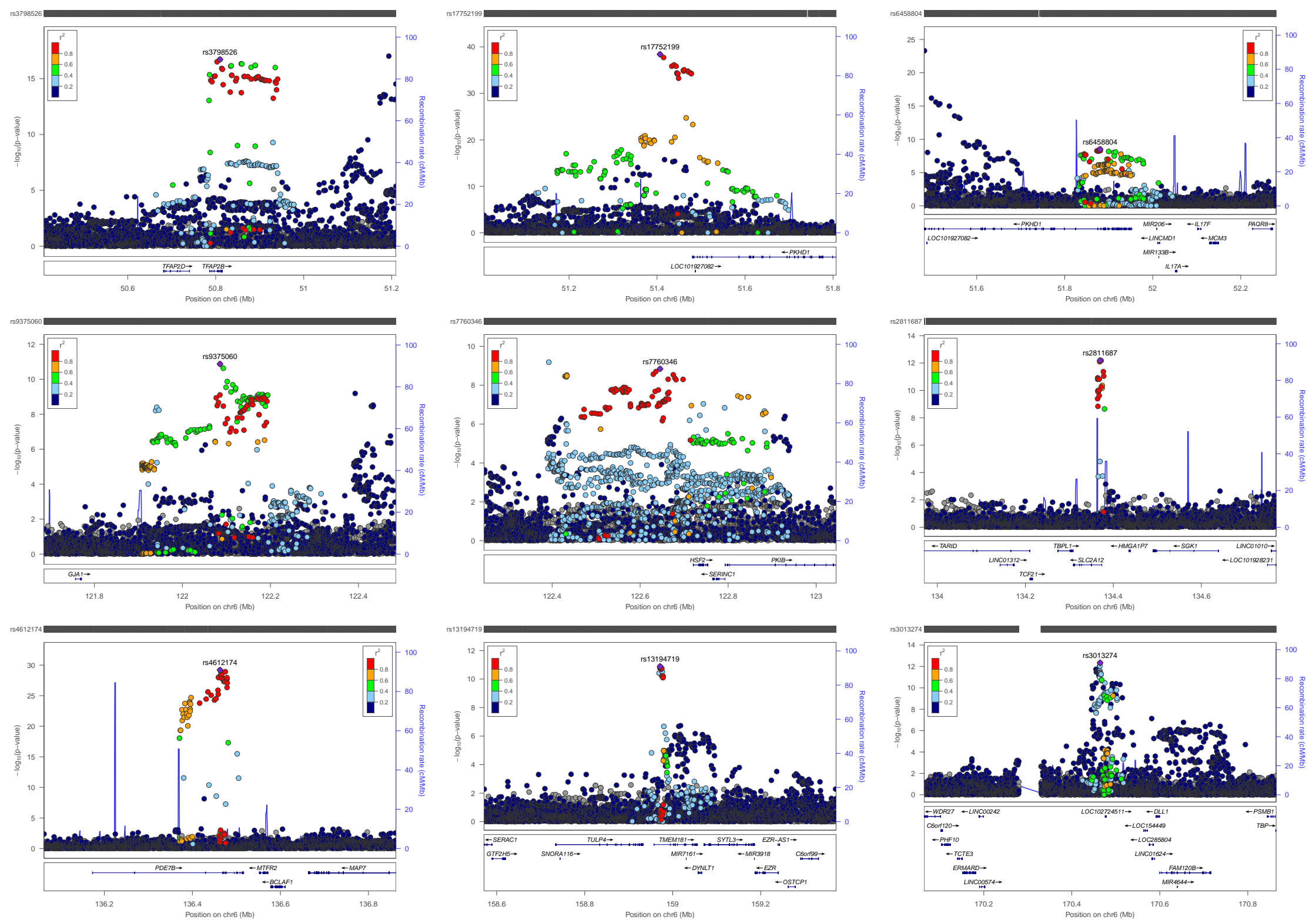

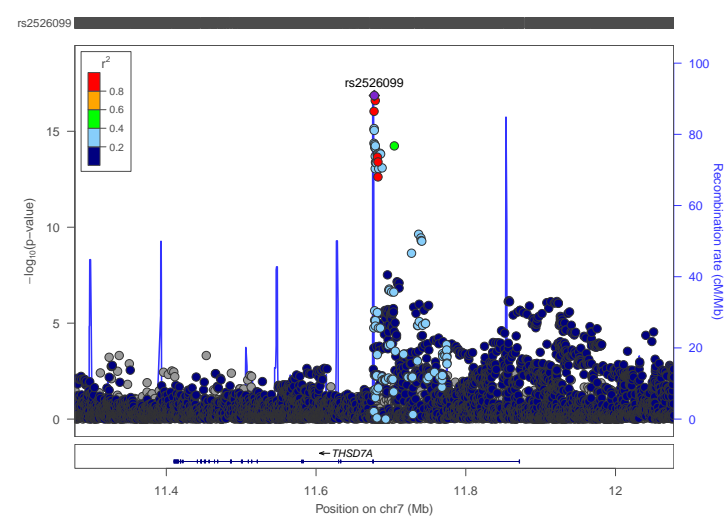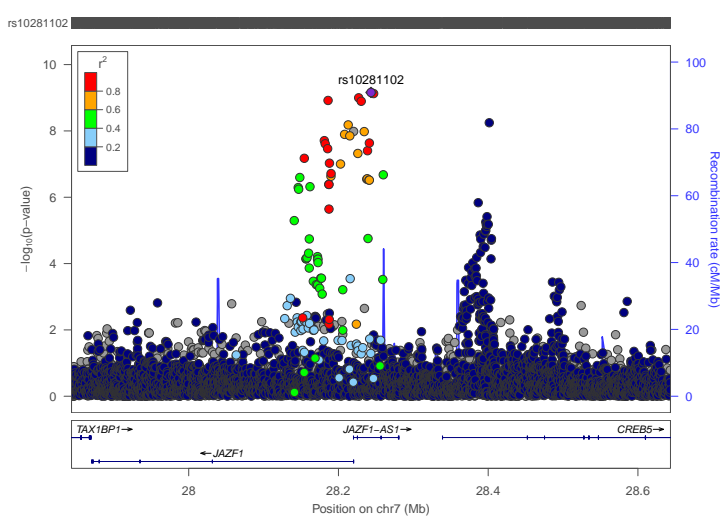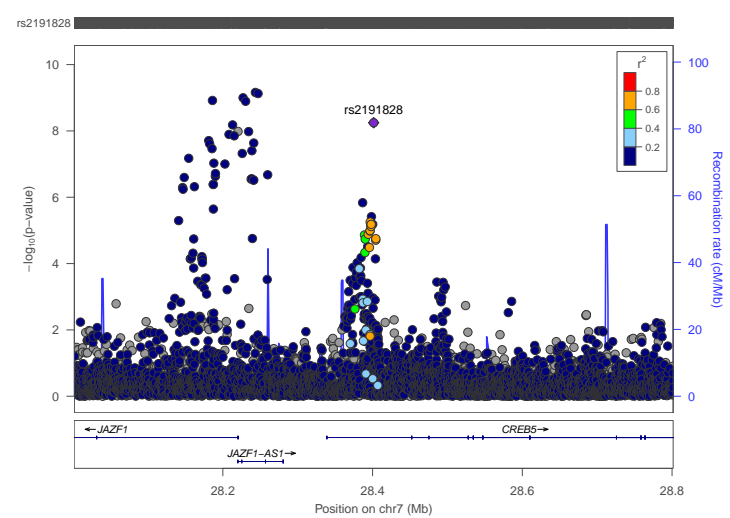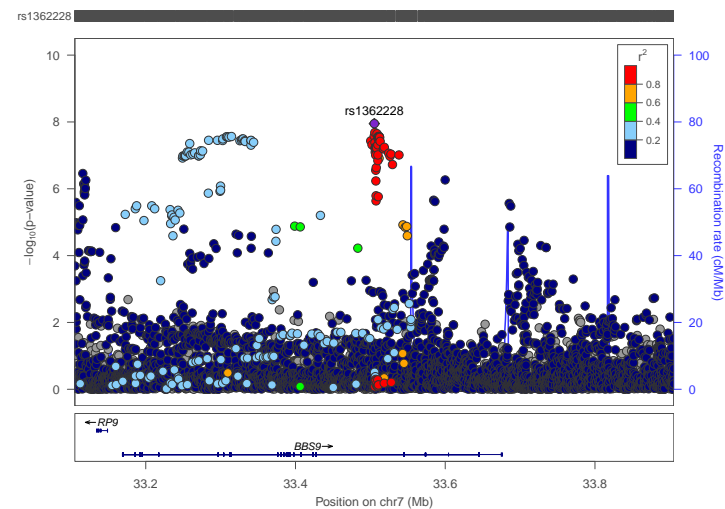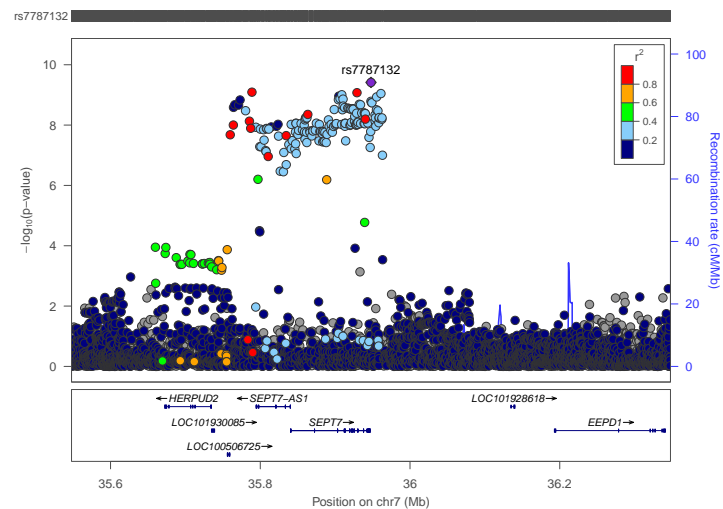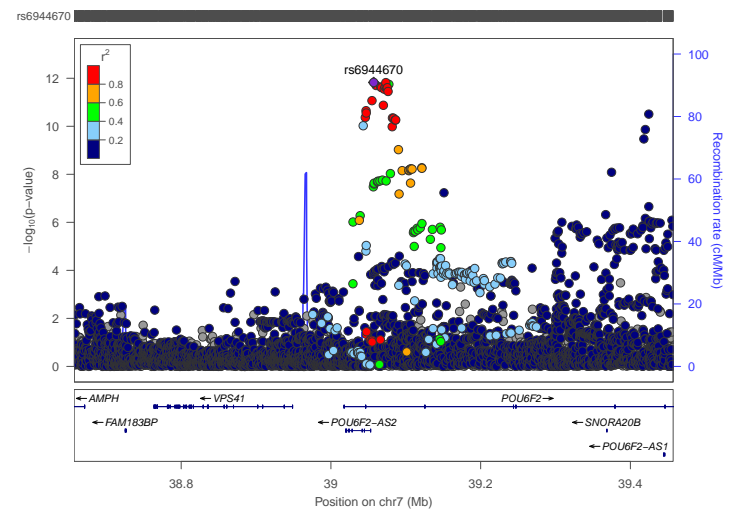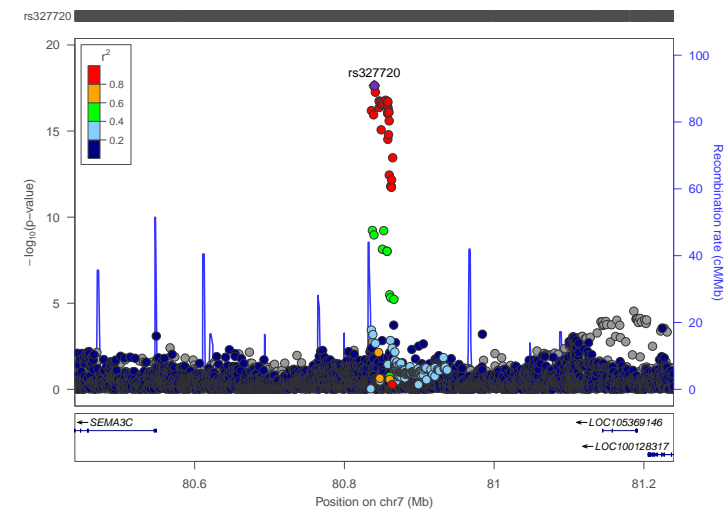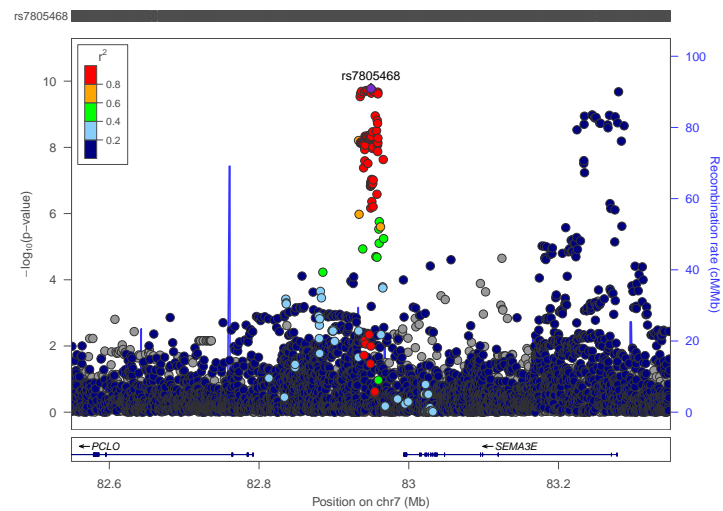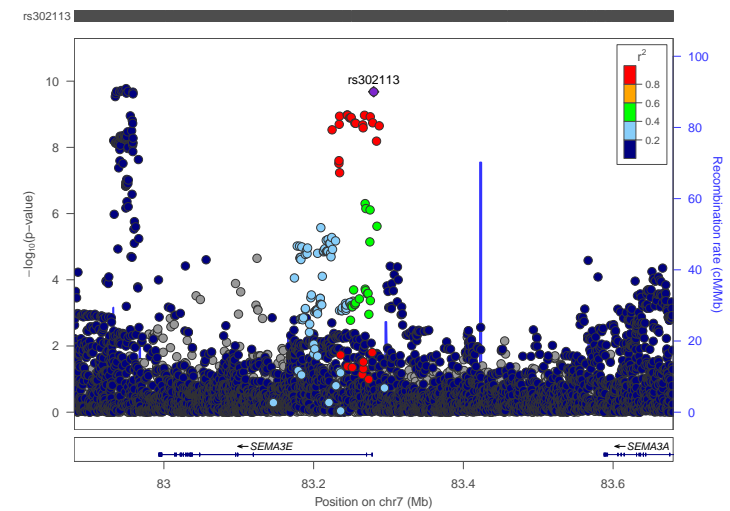

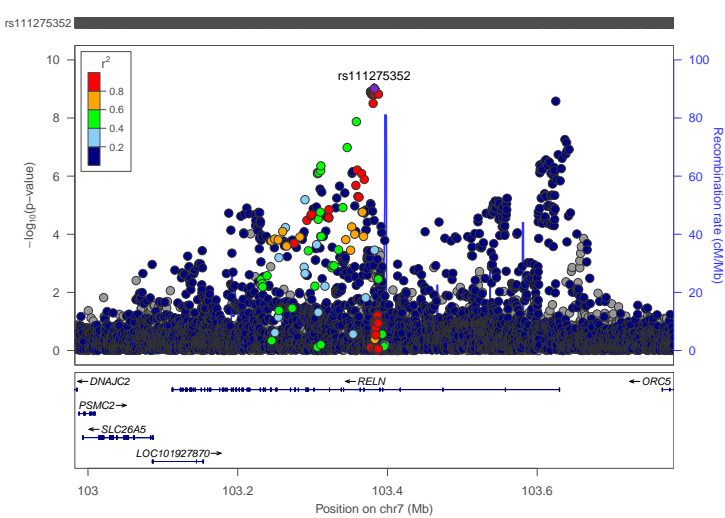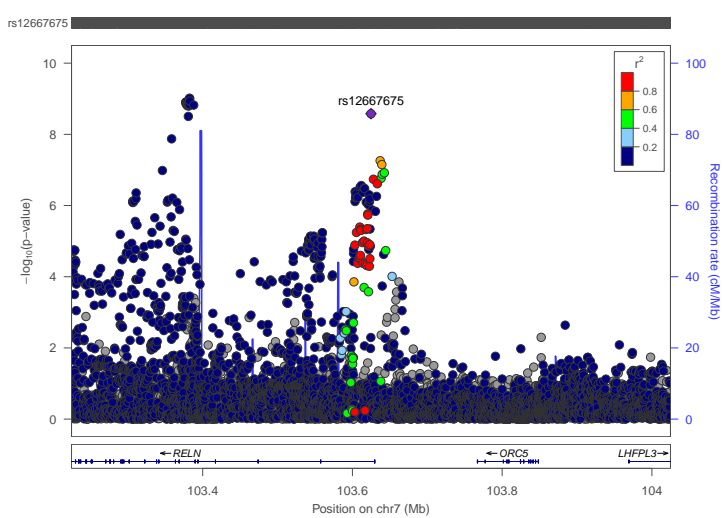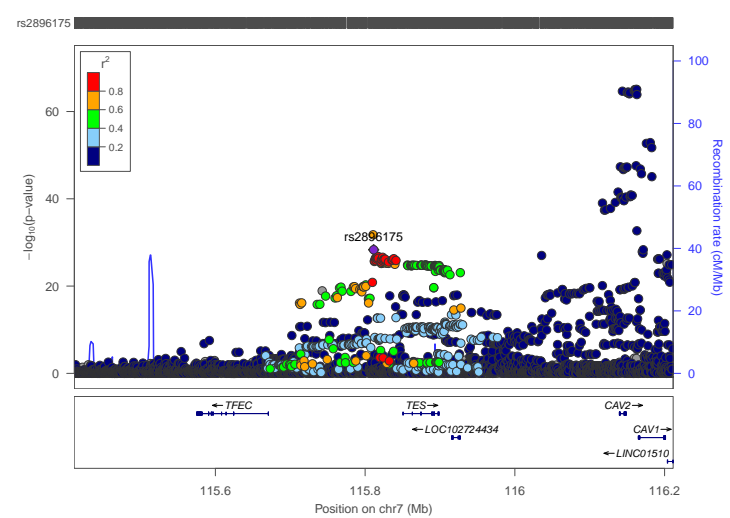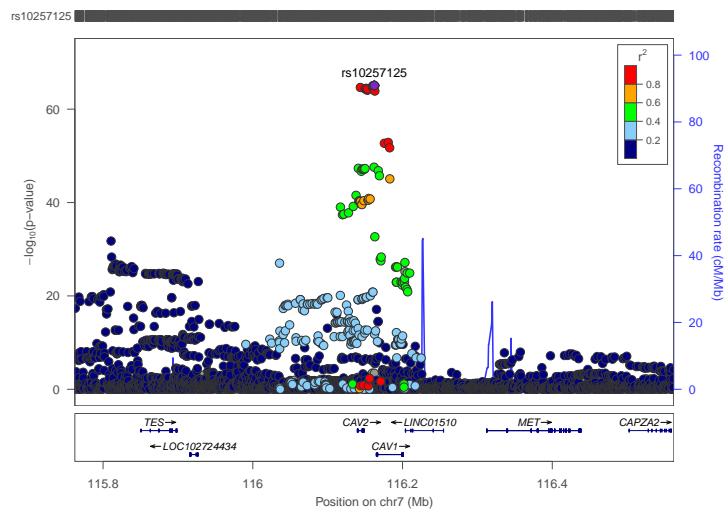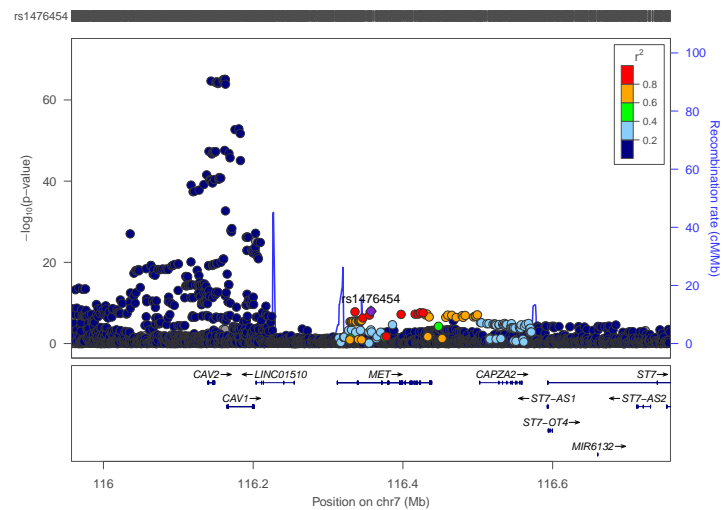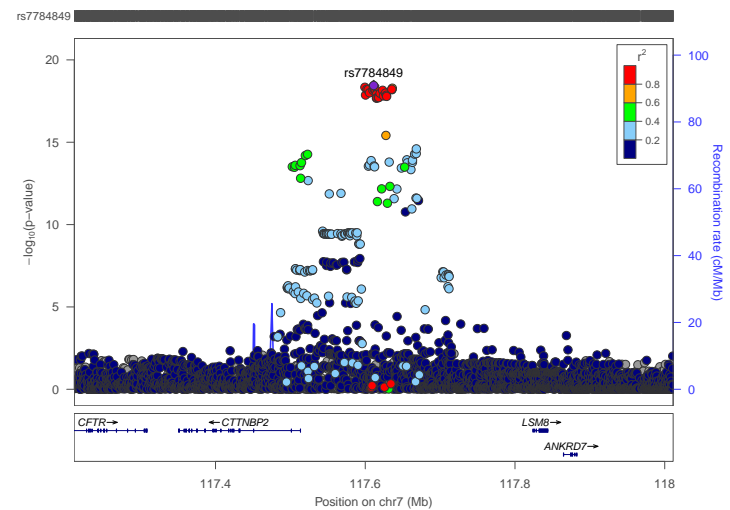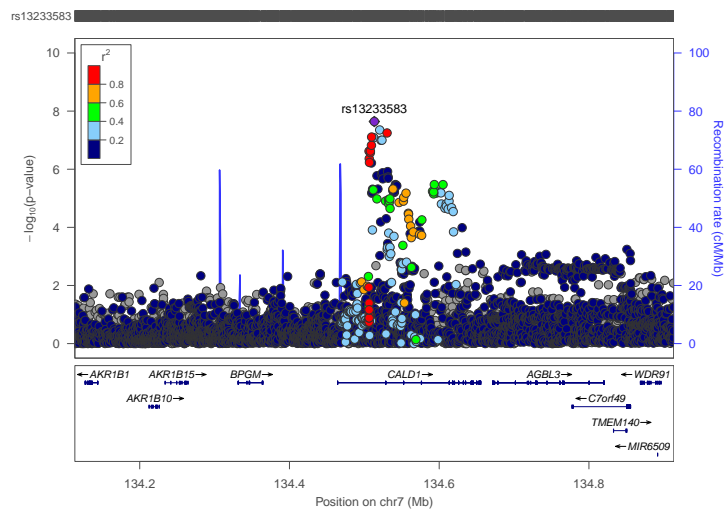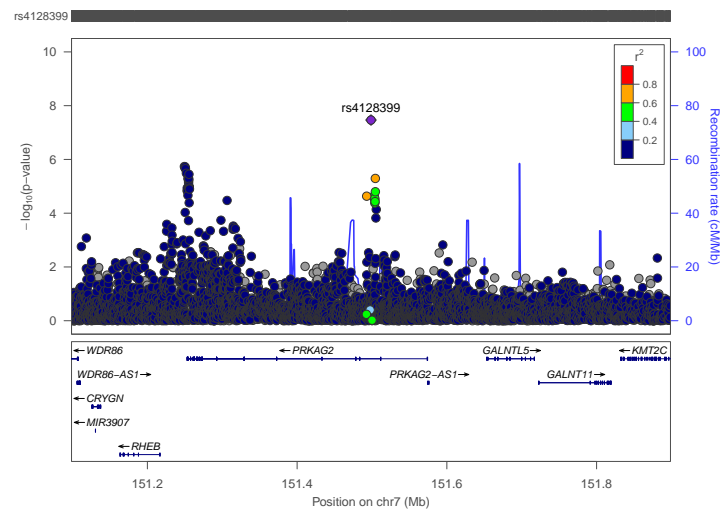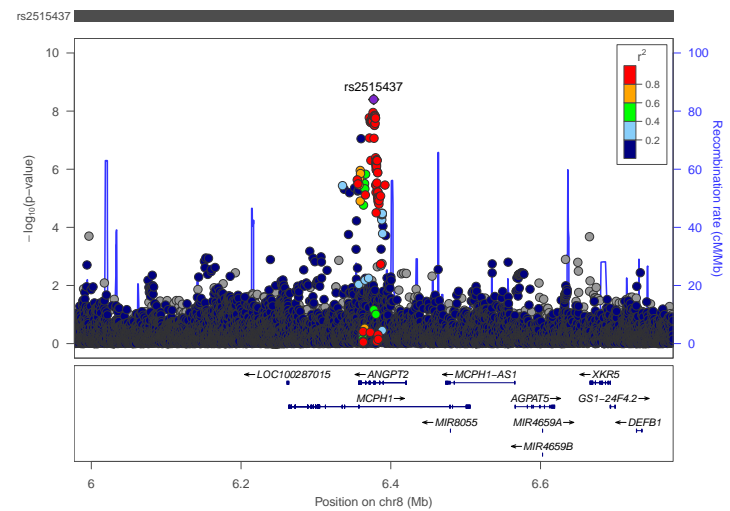

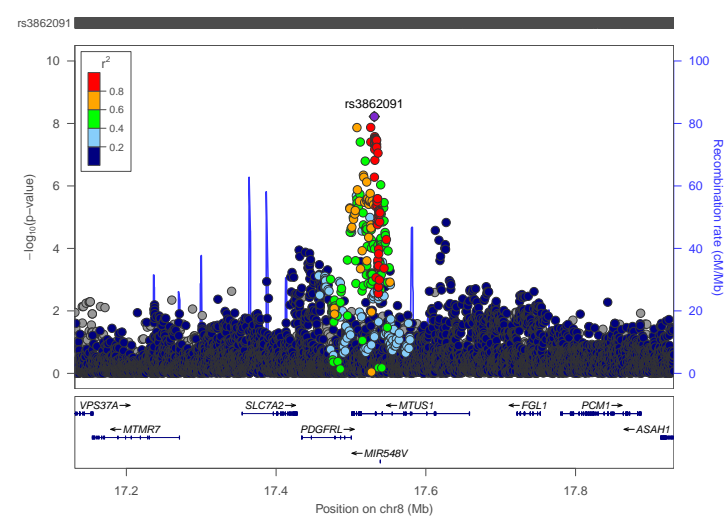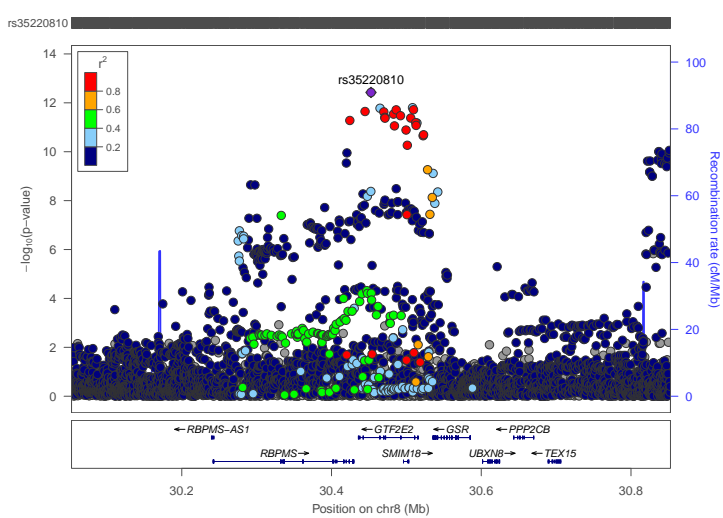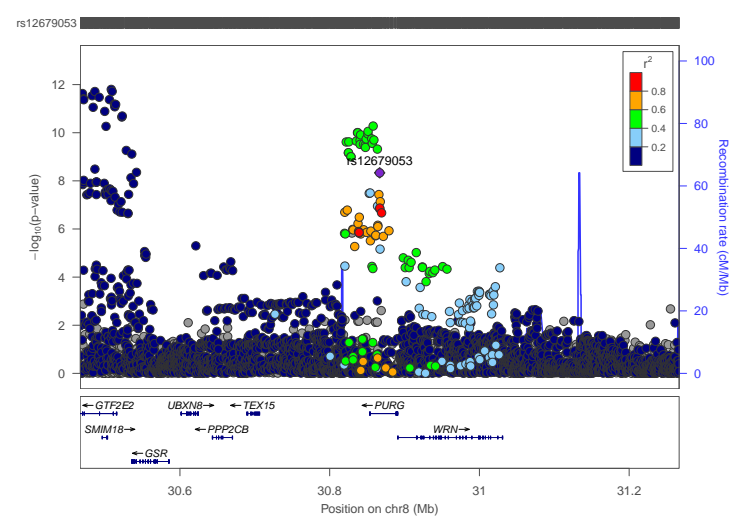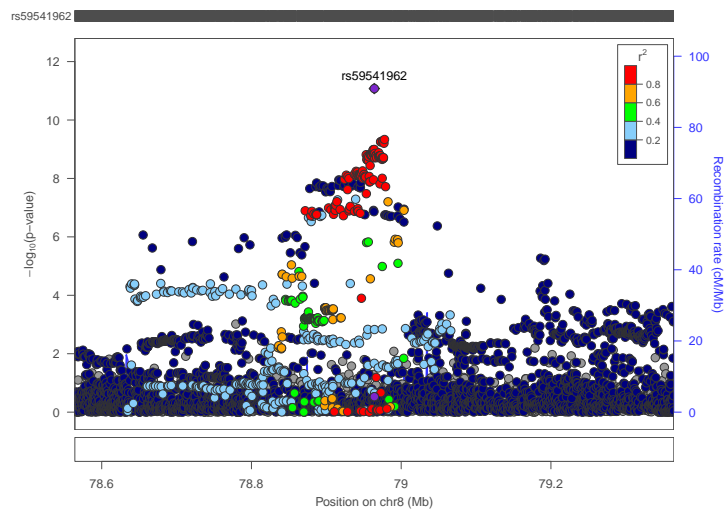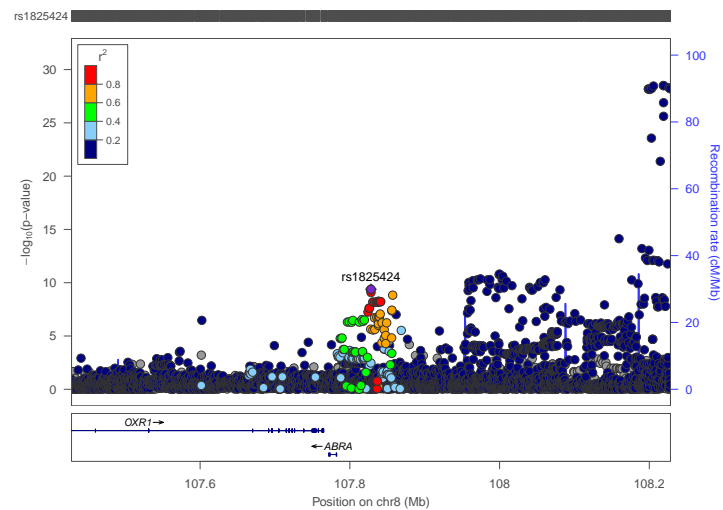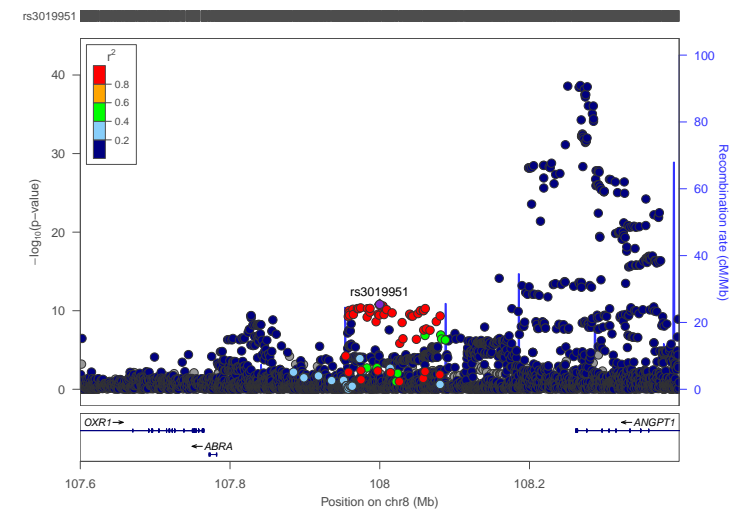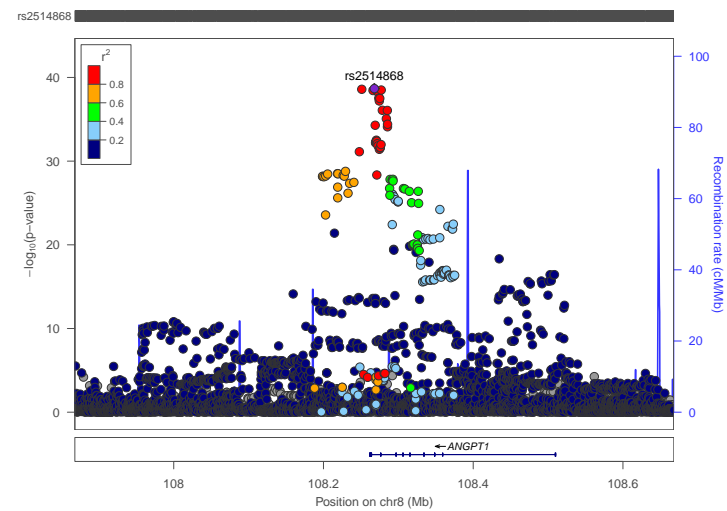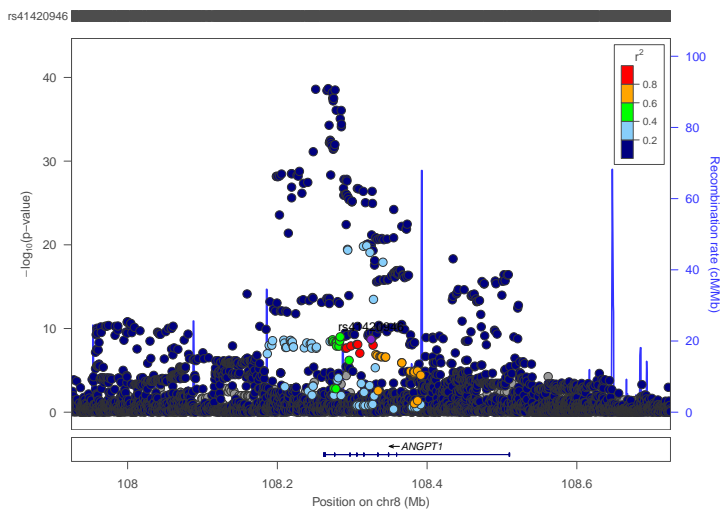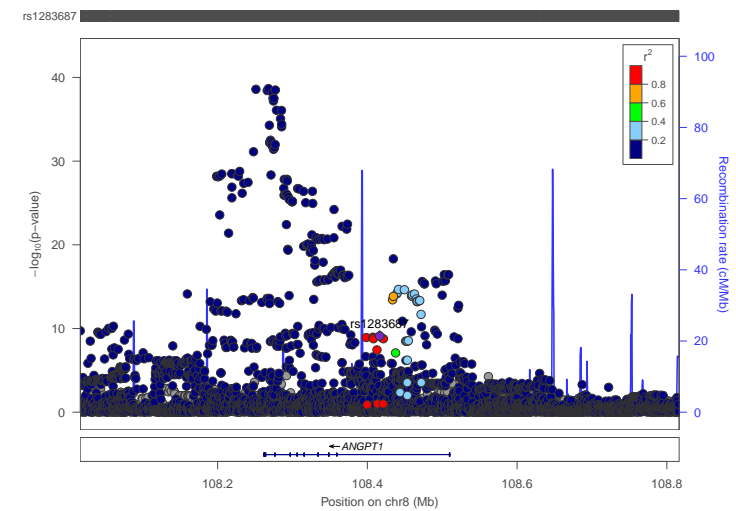

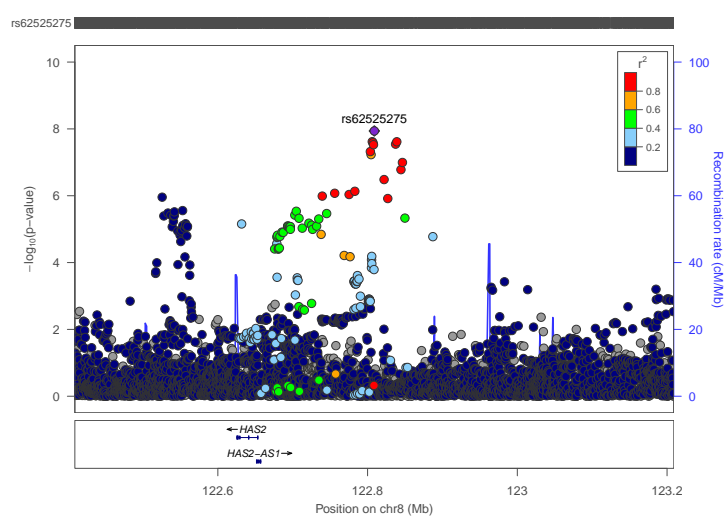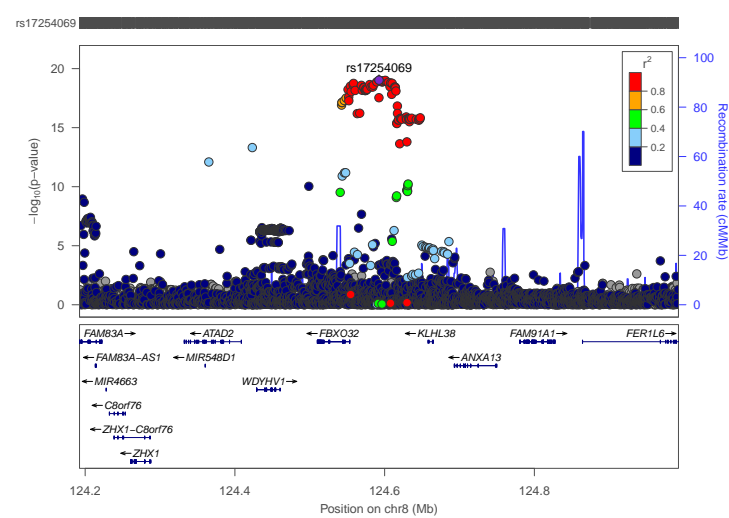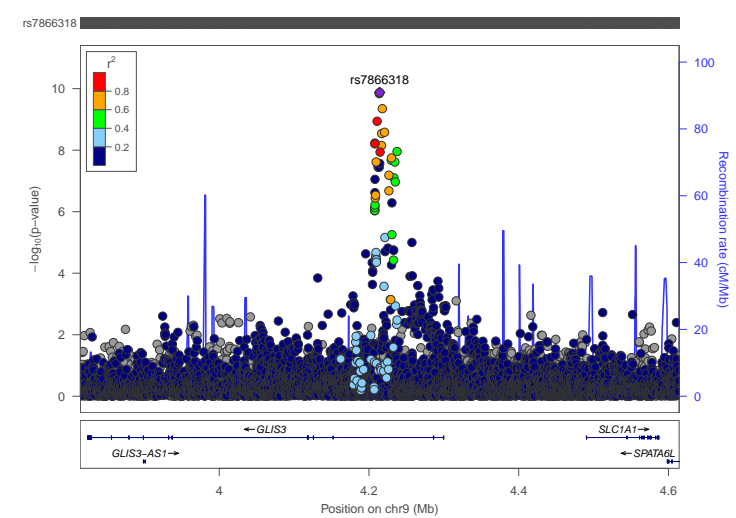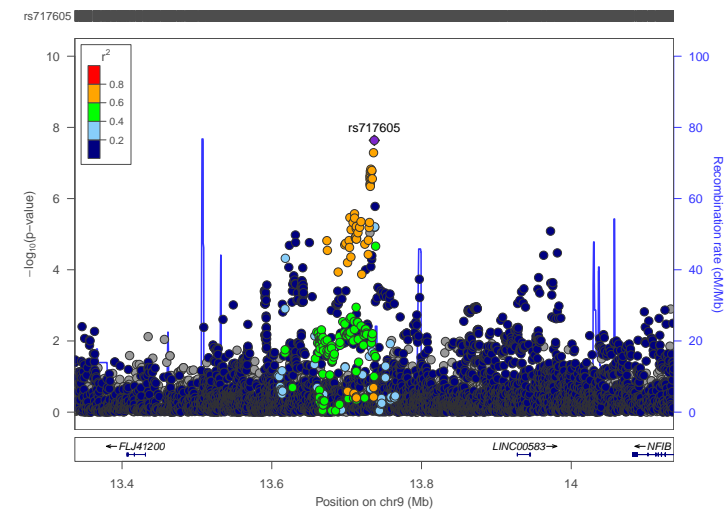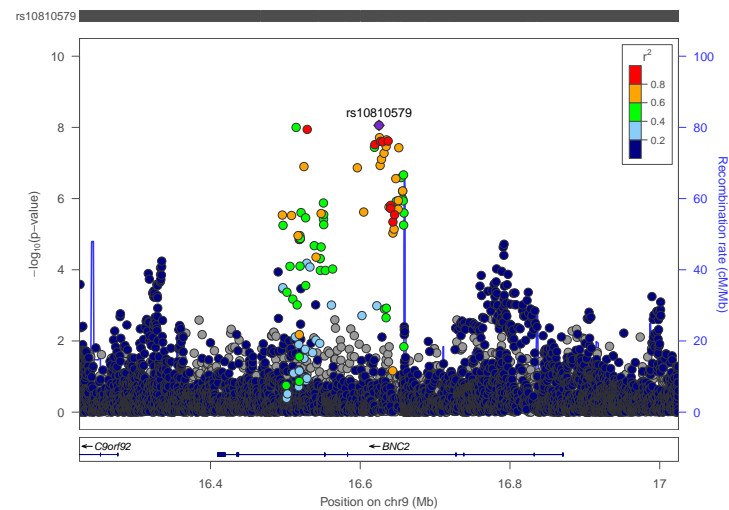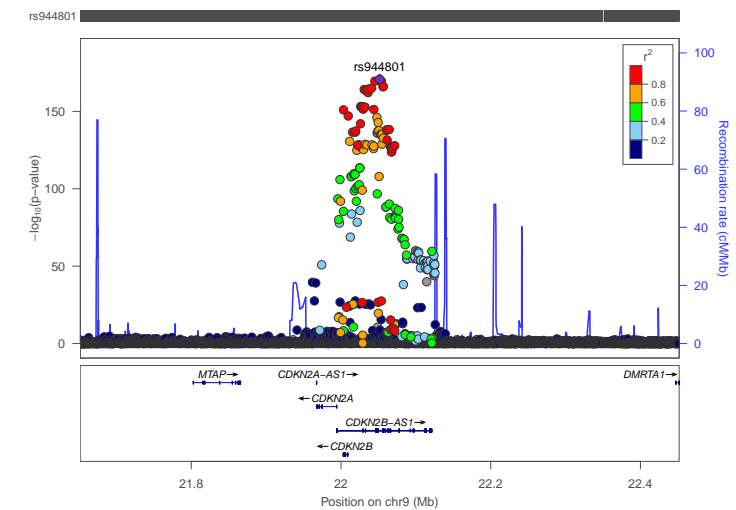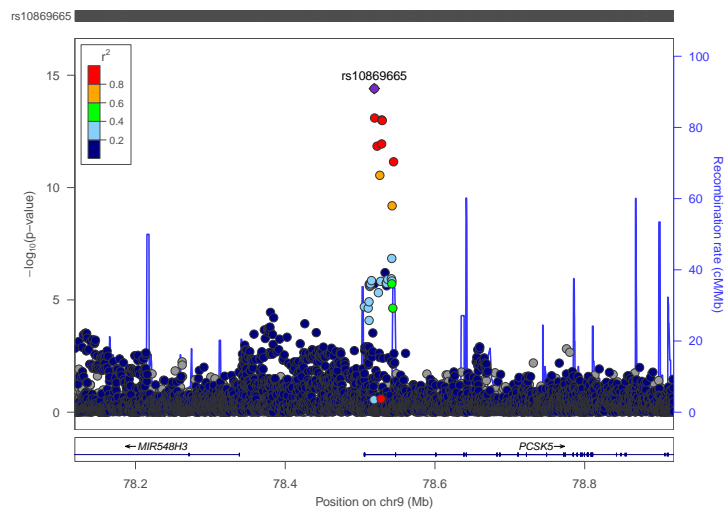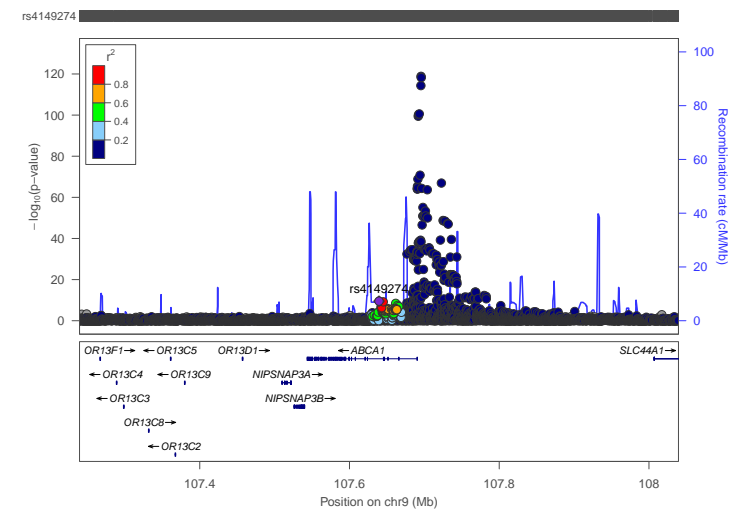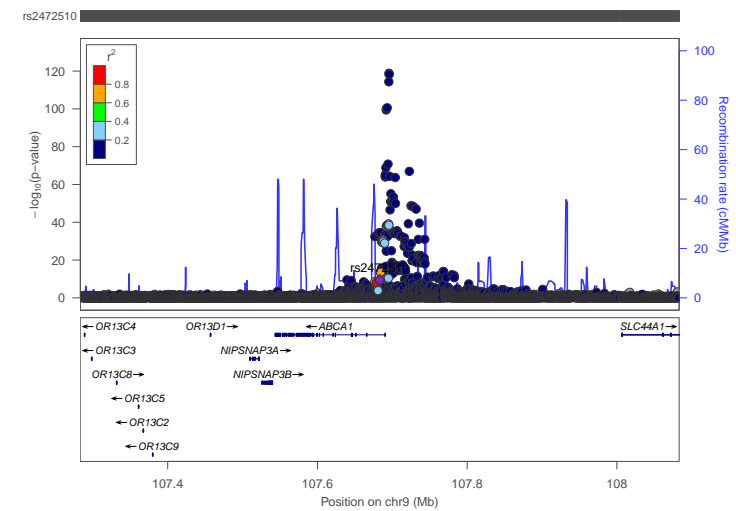

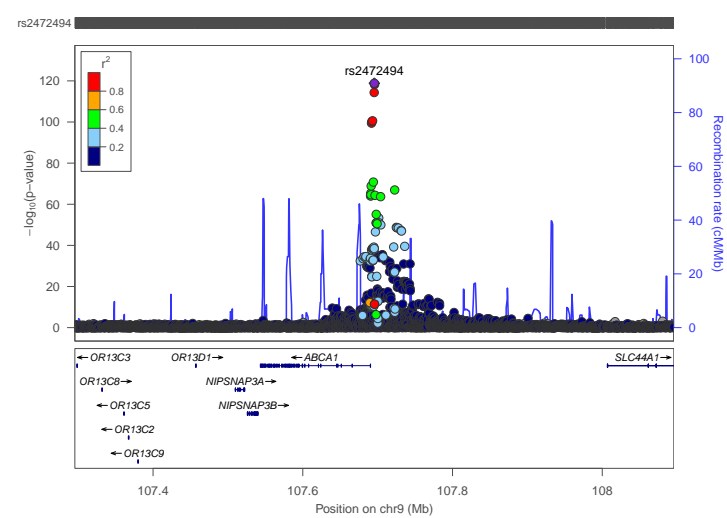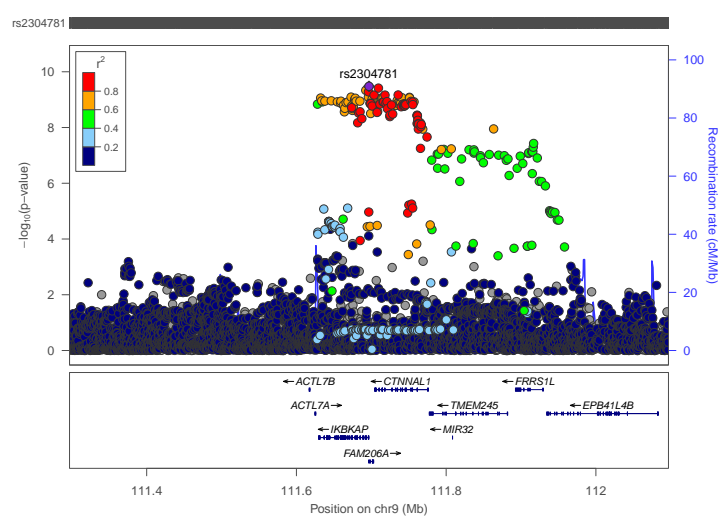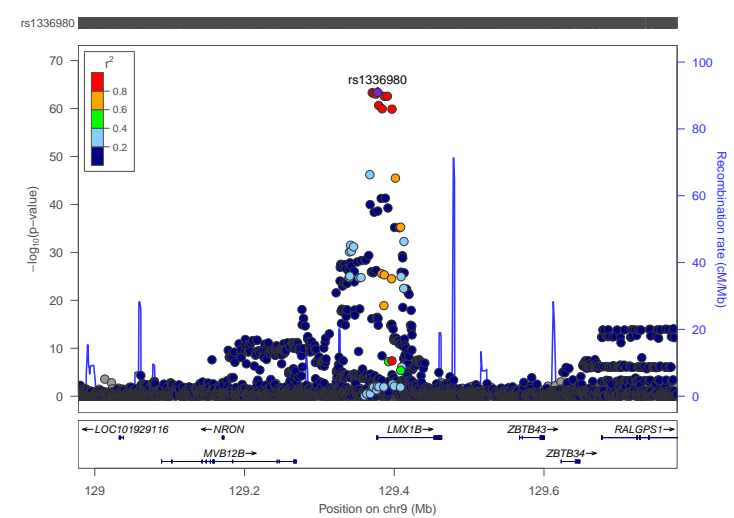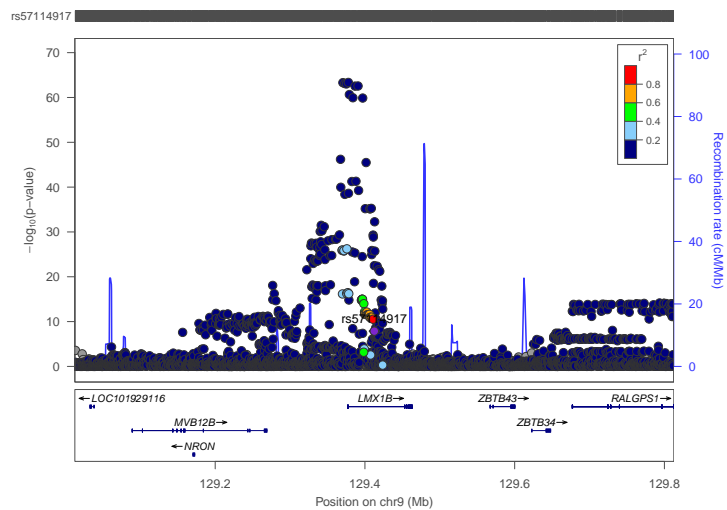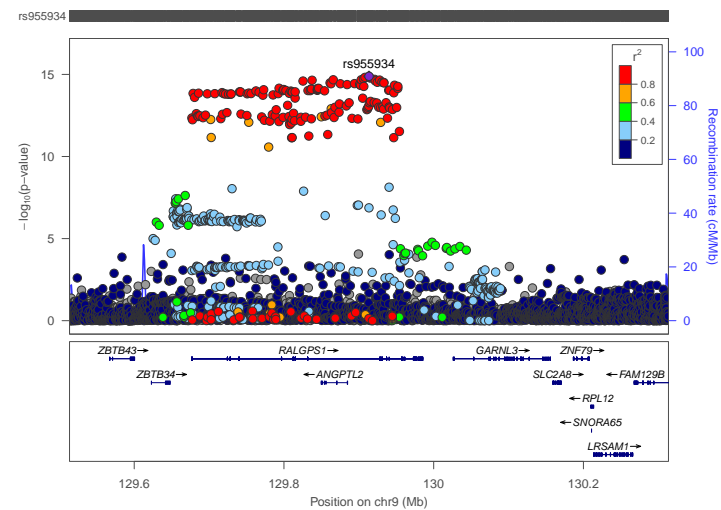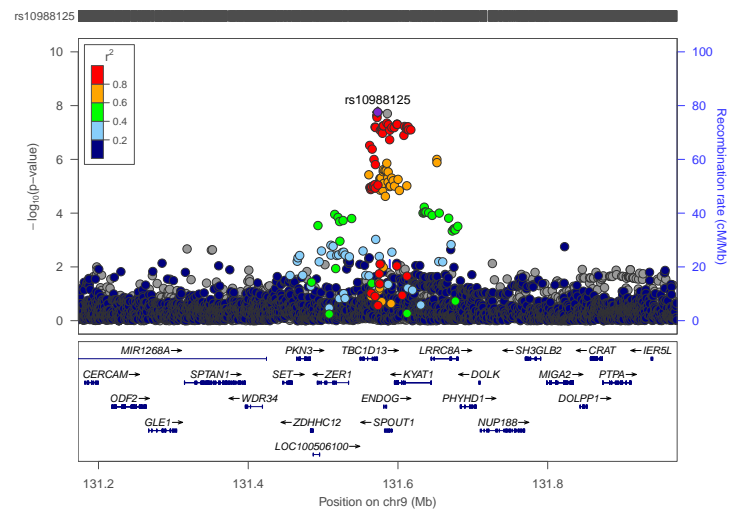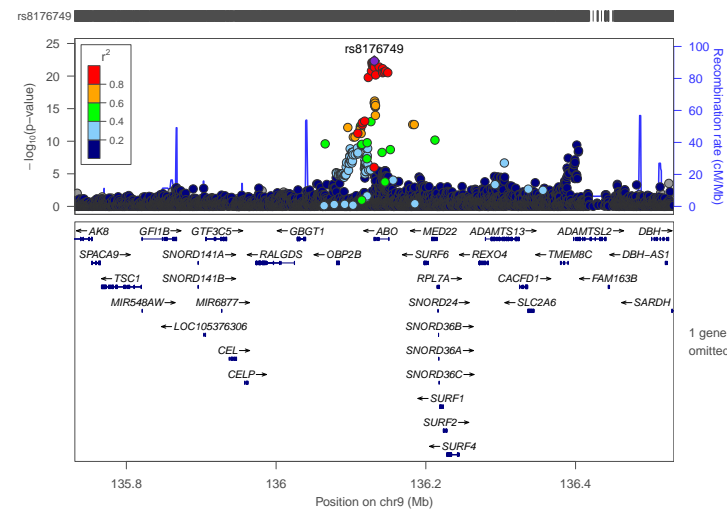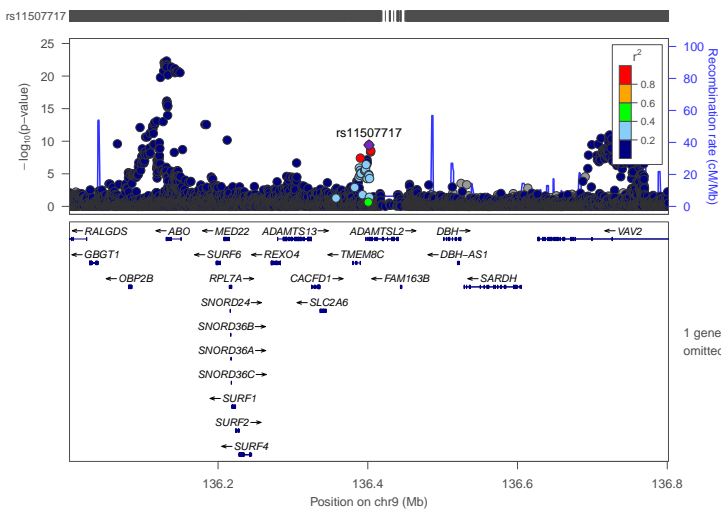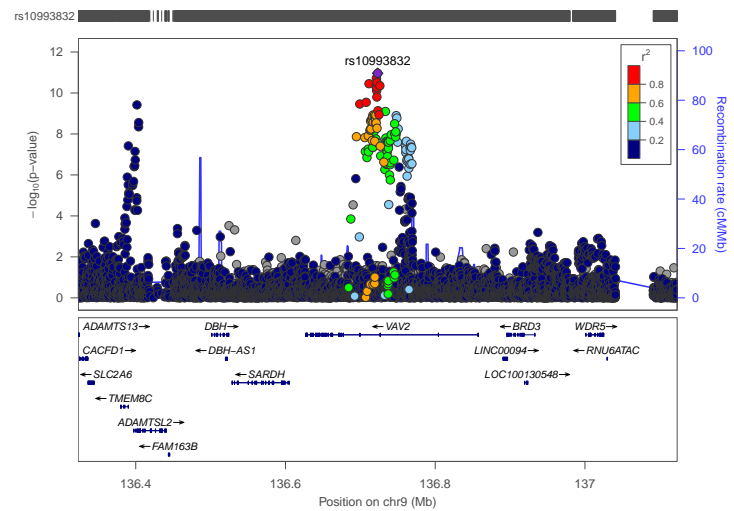

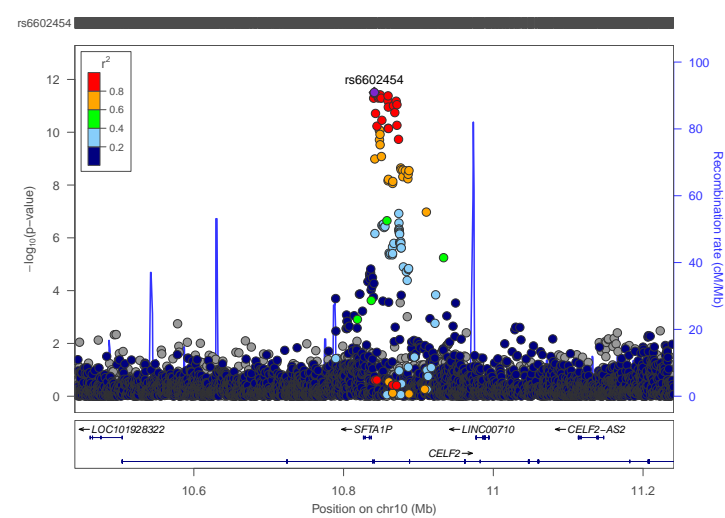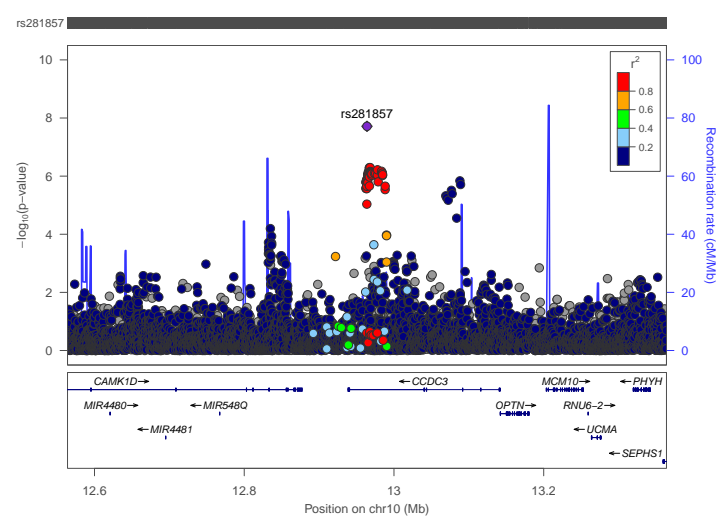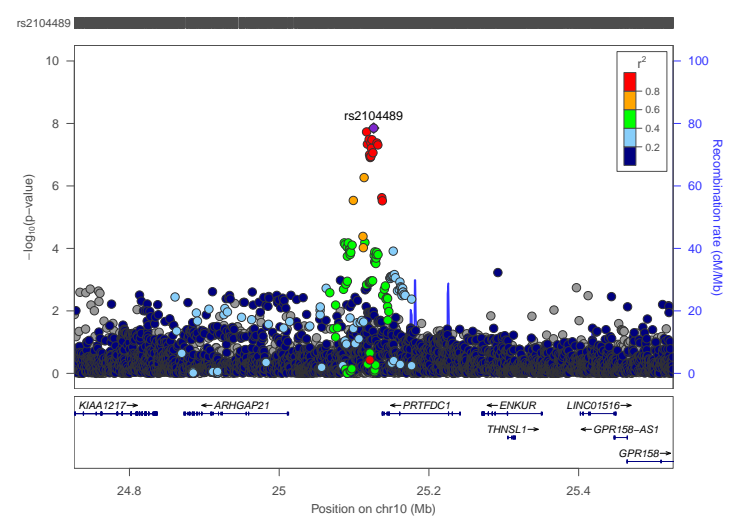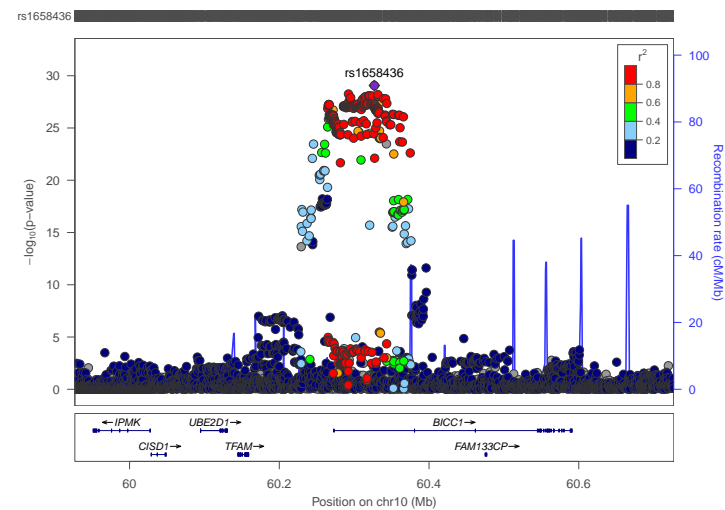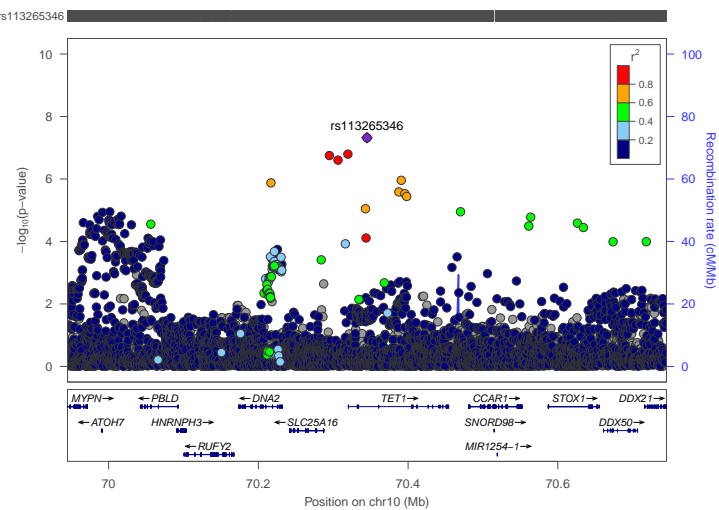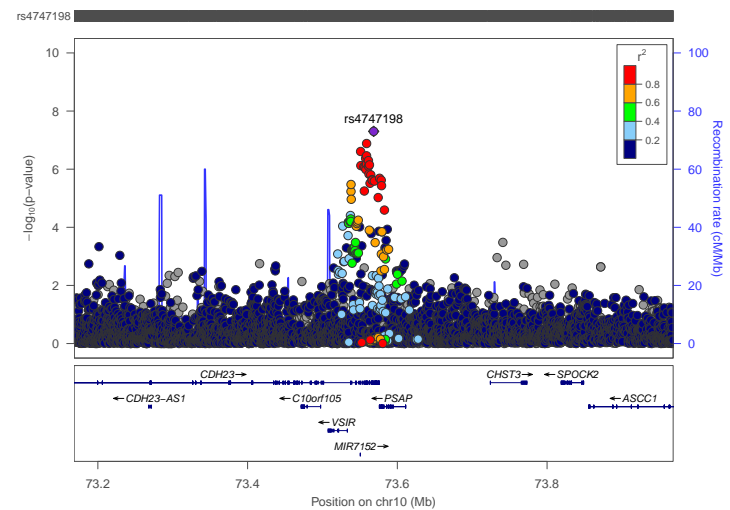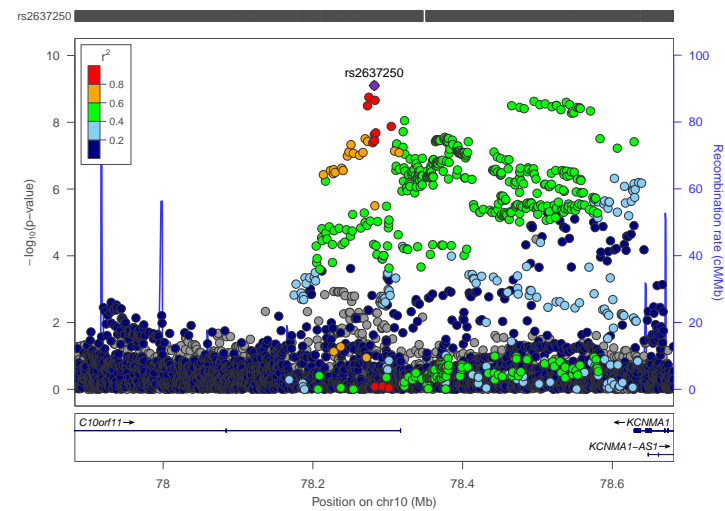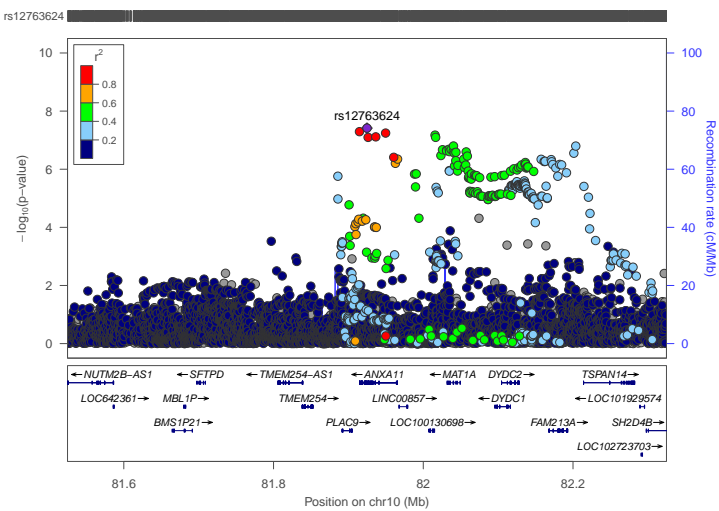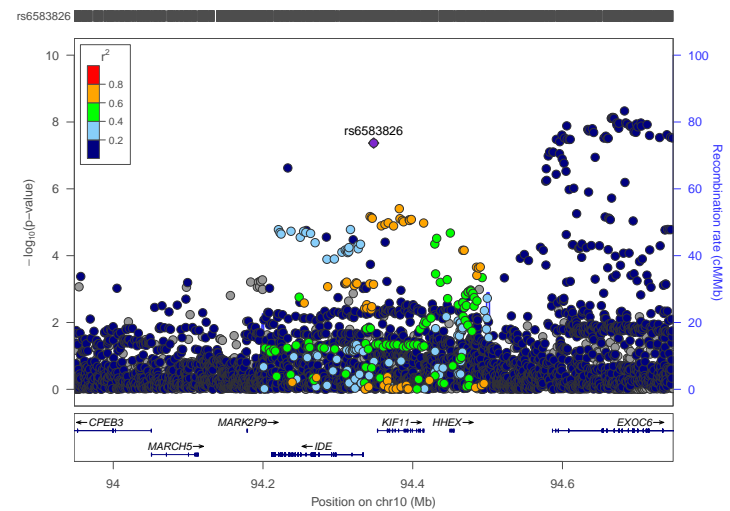

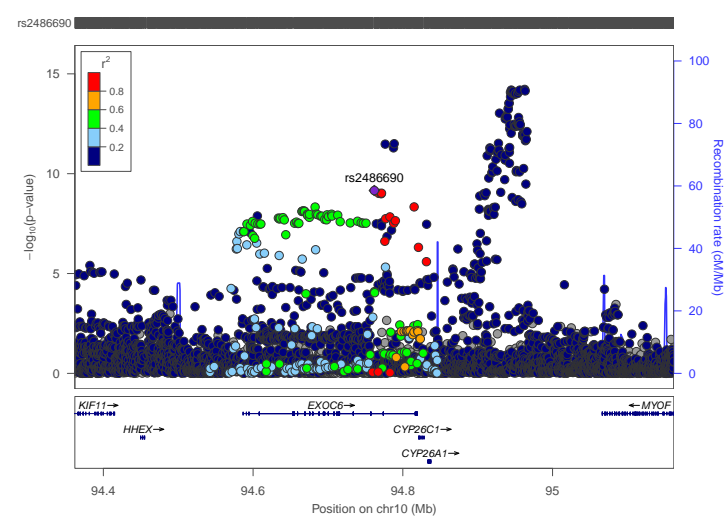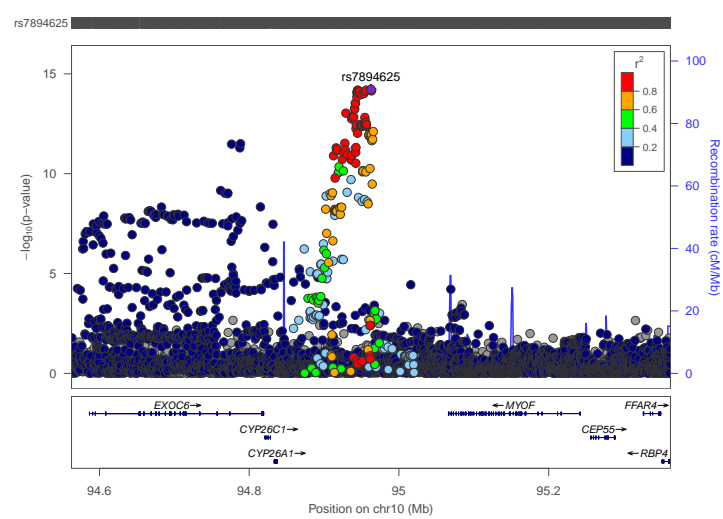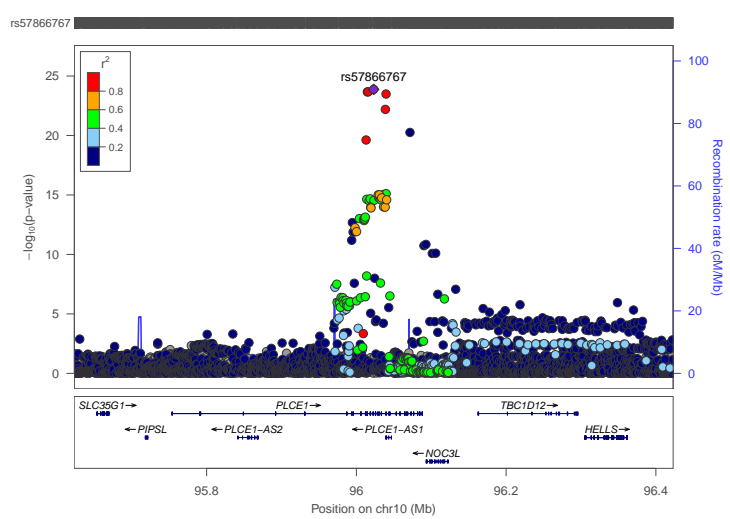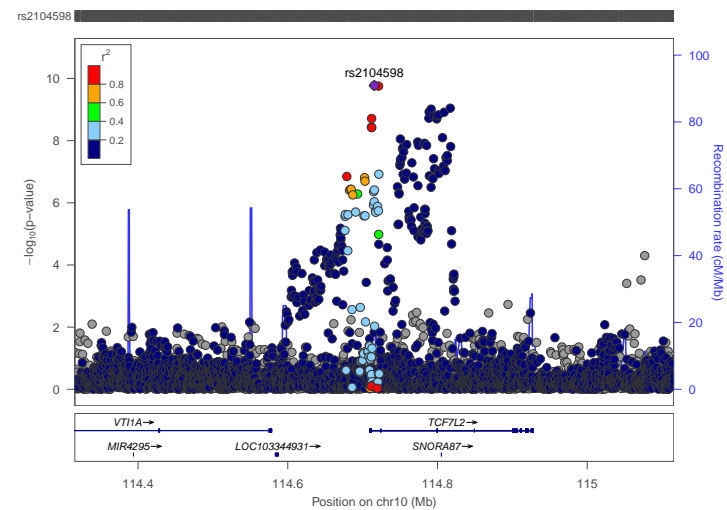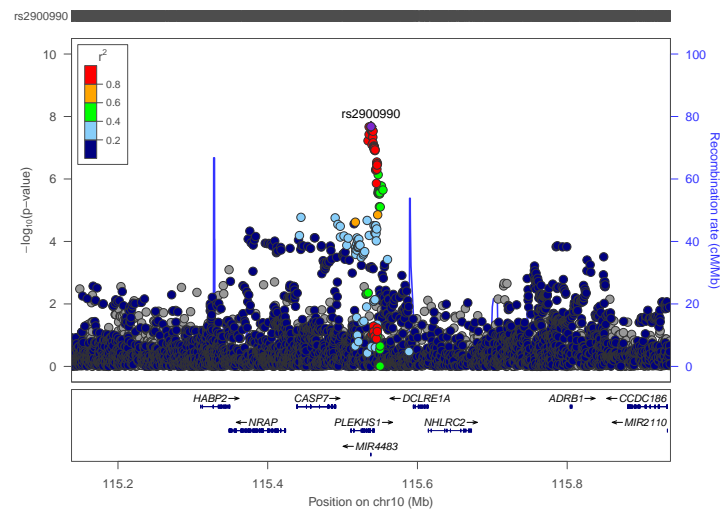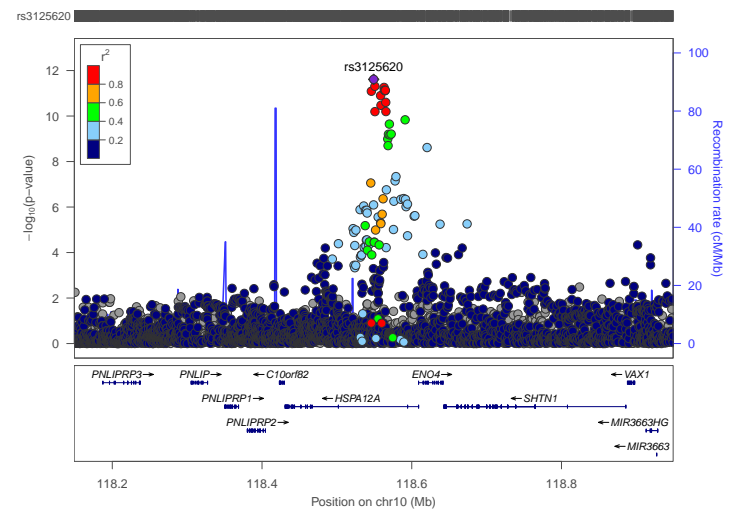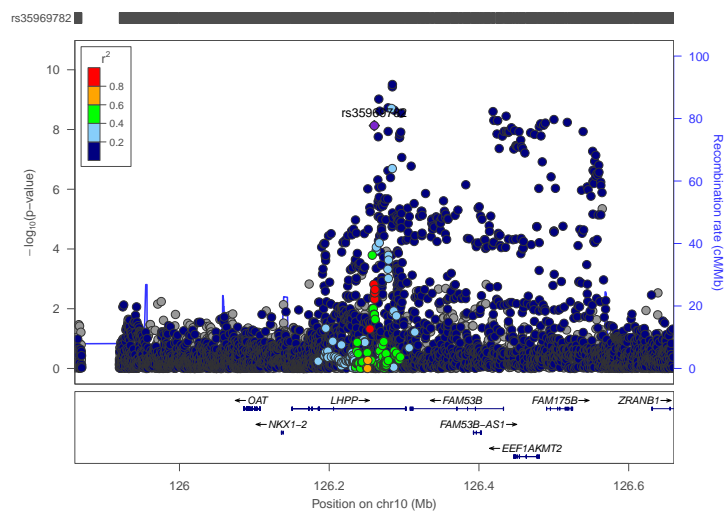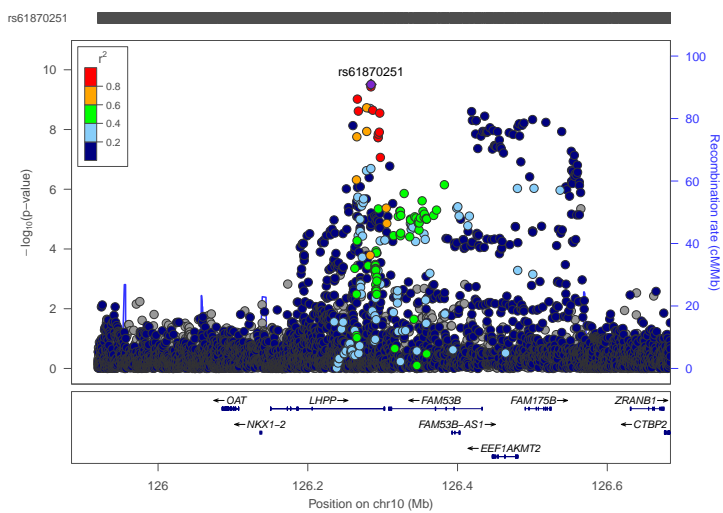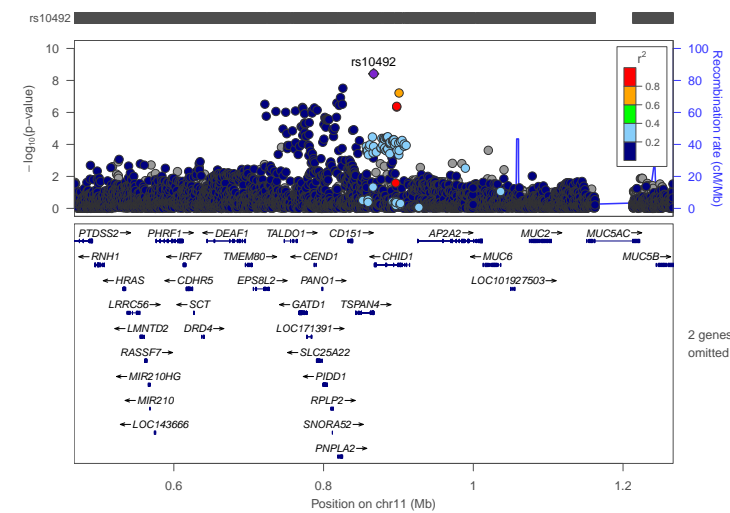

2 genes omitted

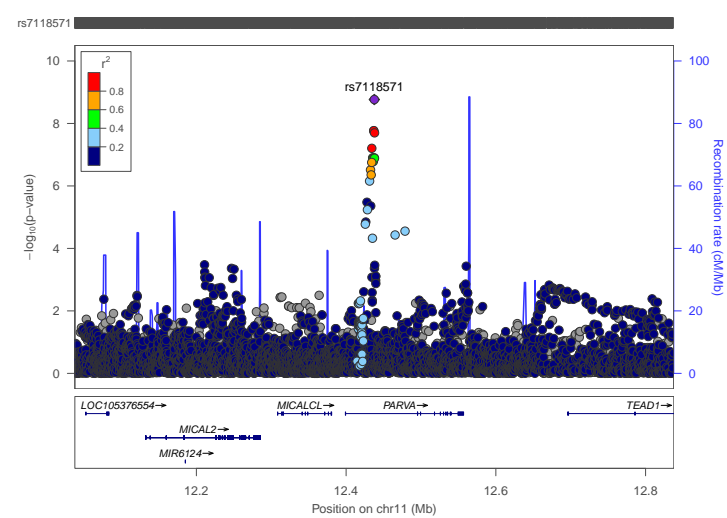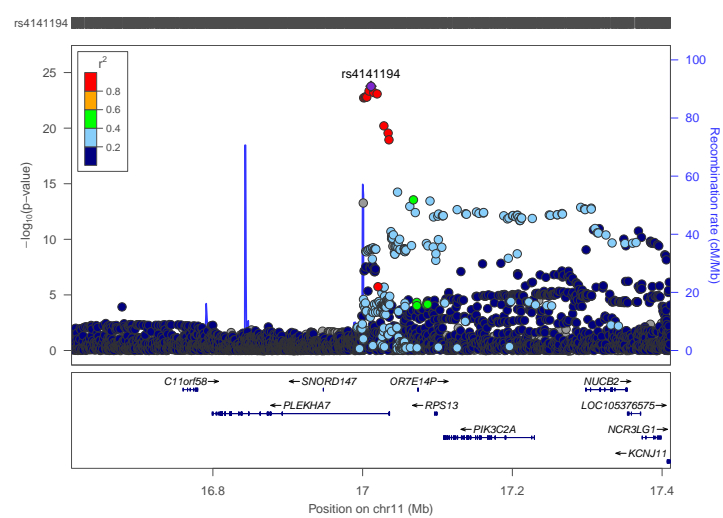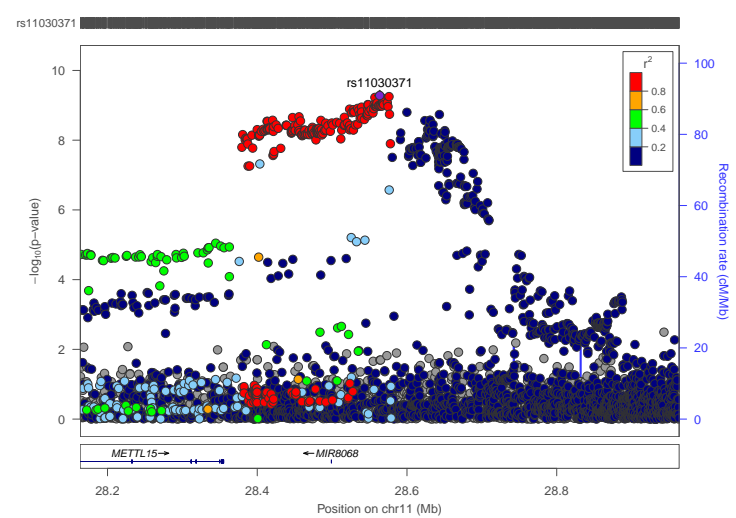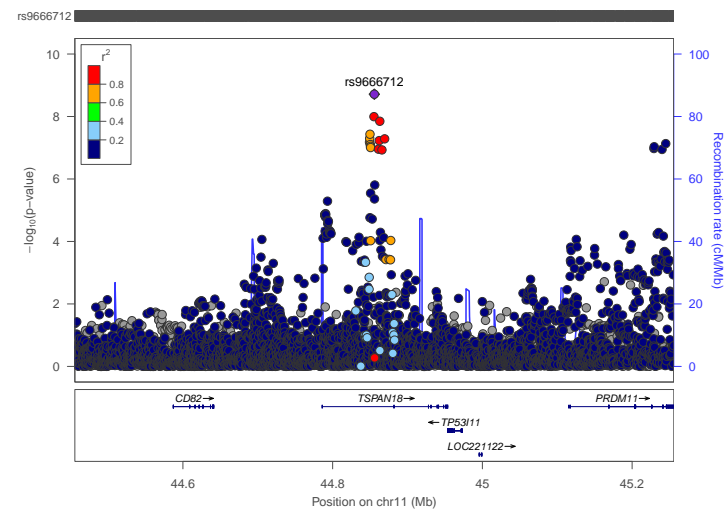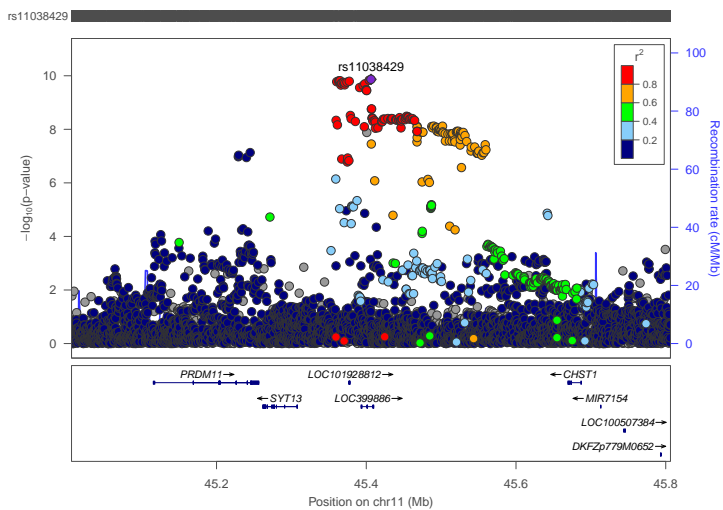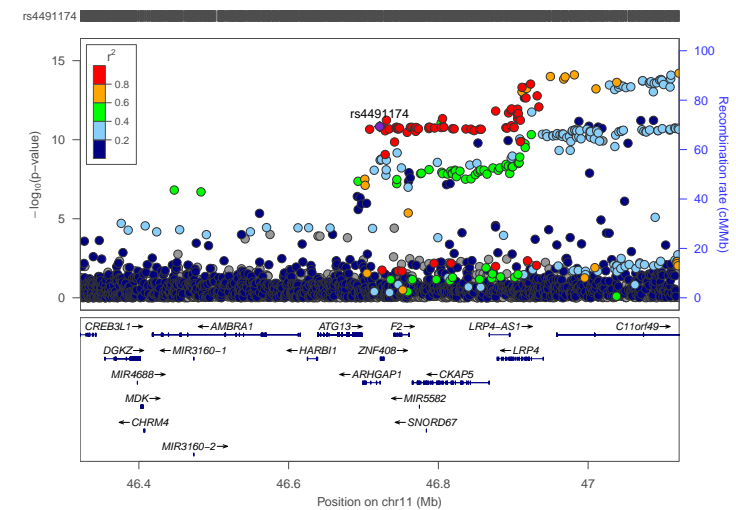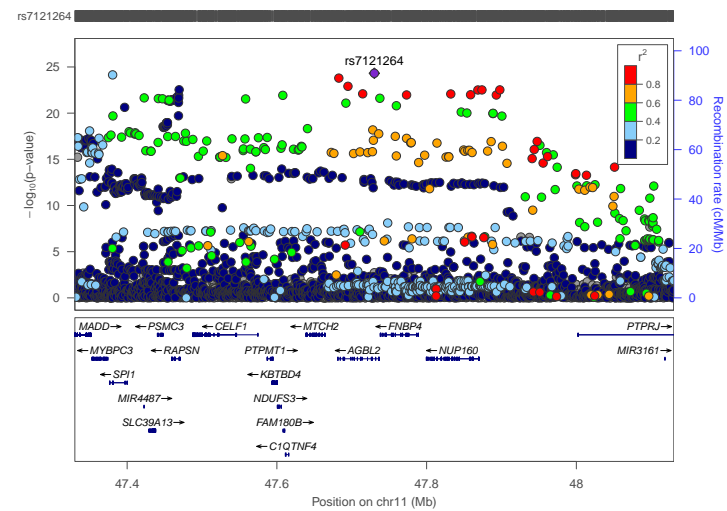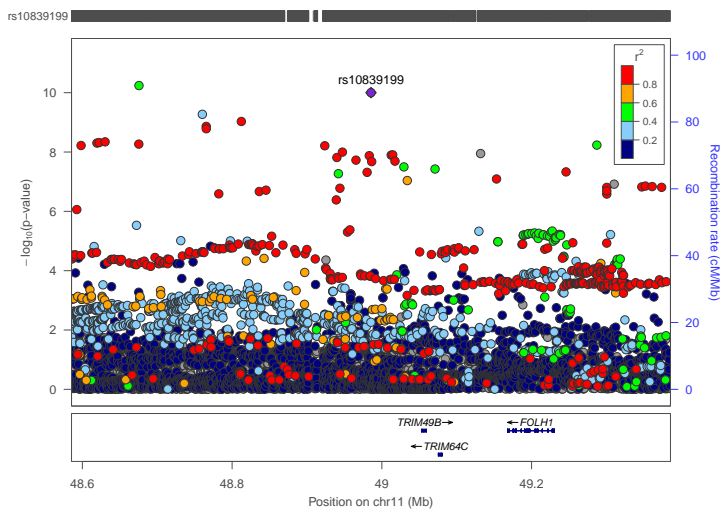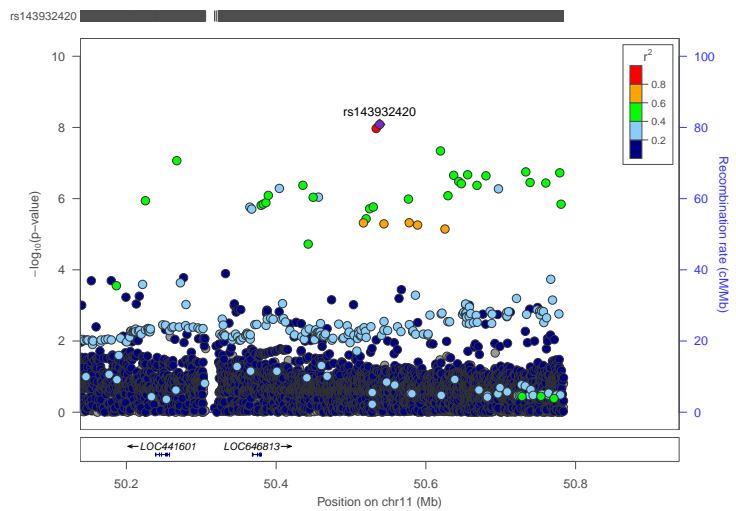

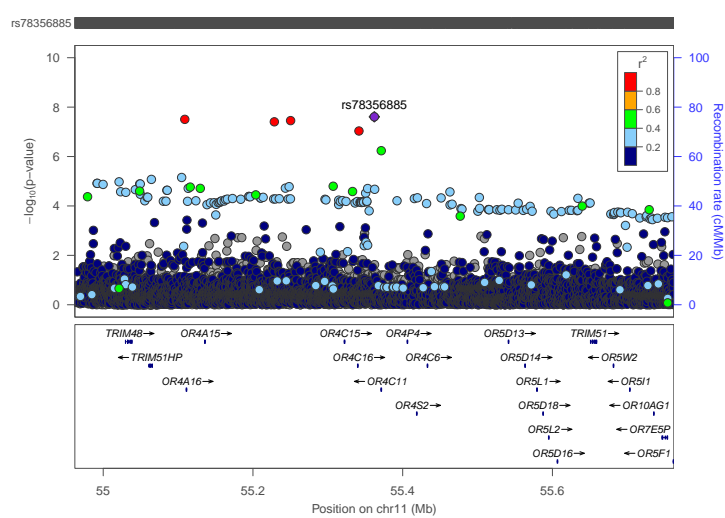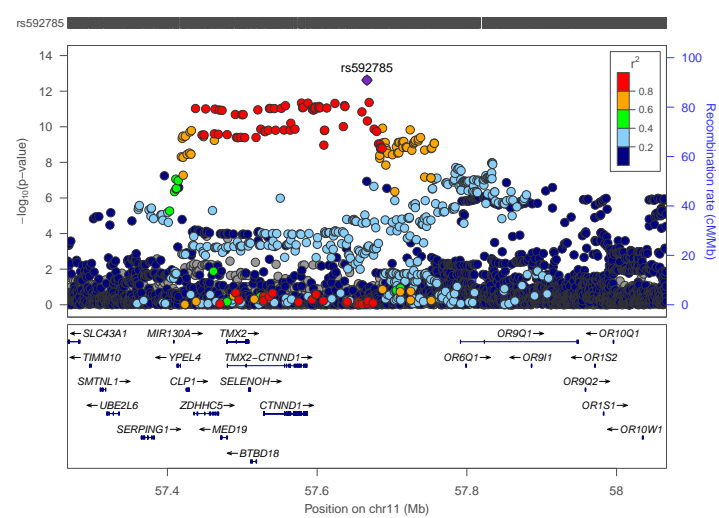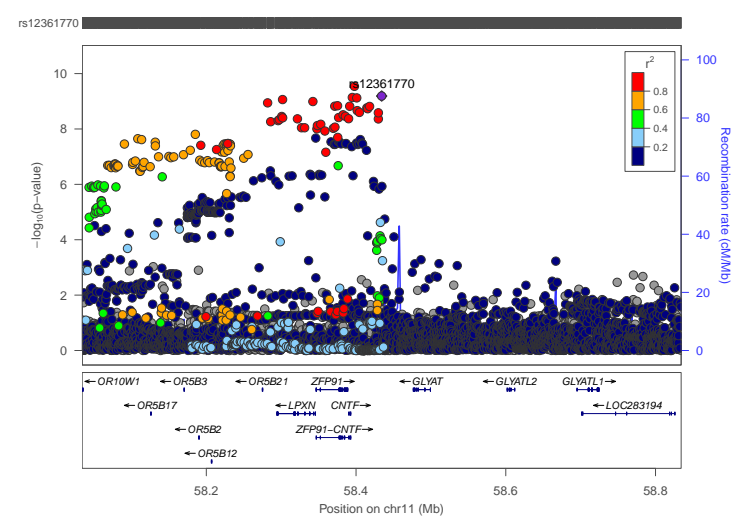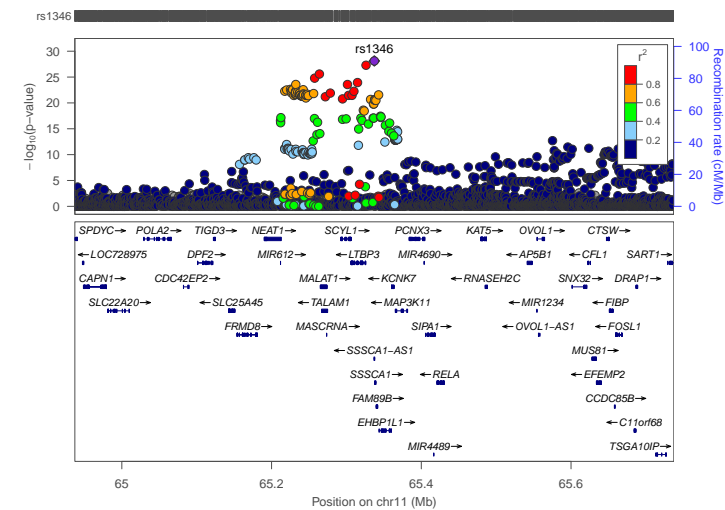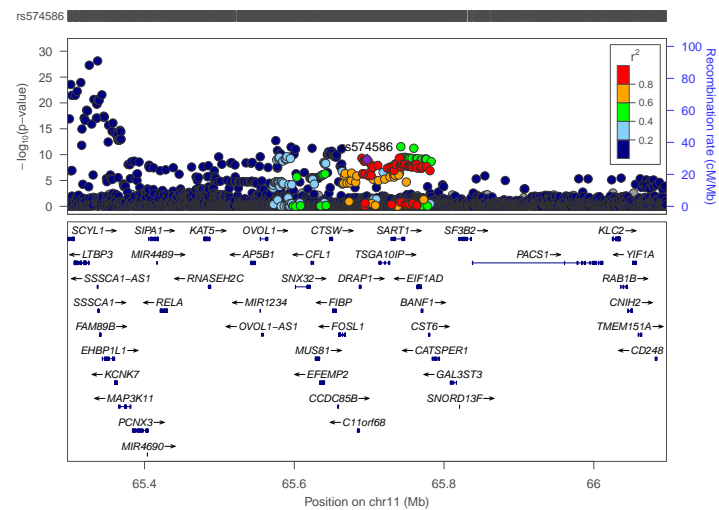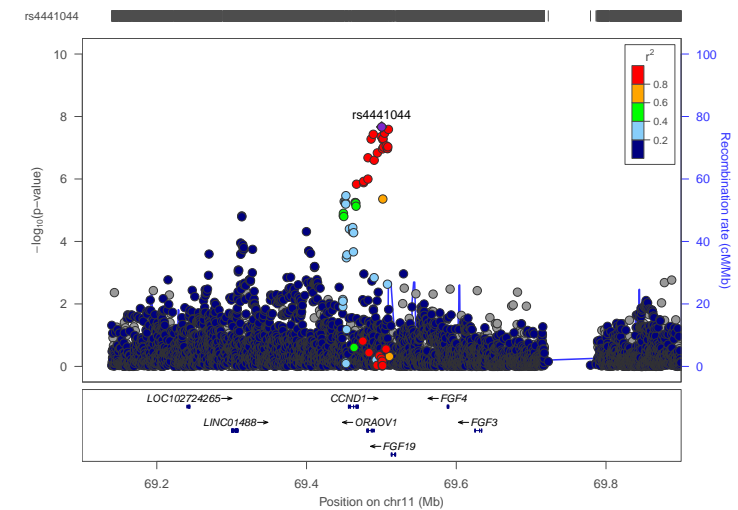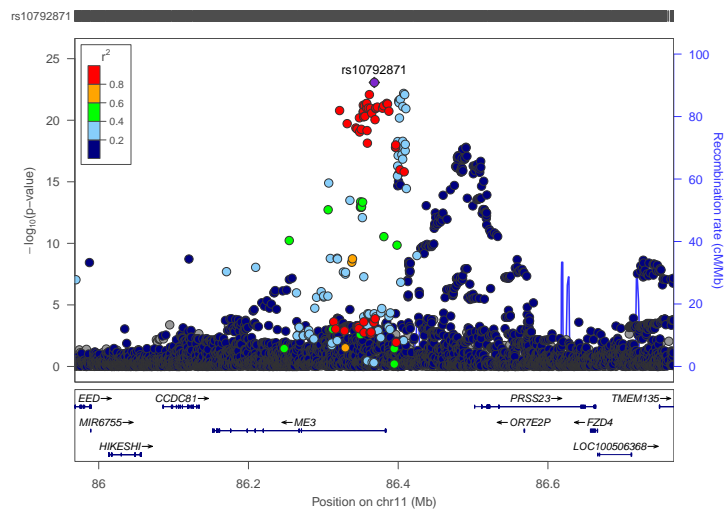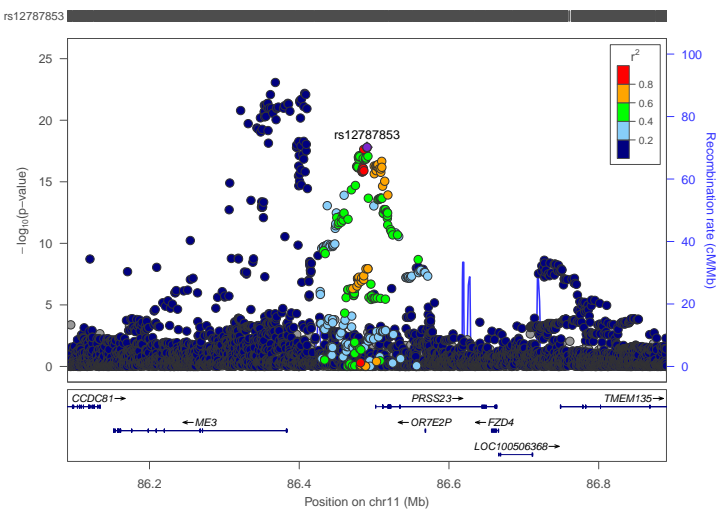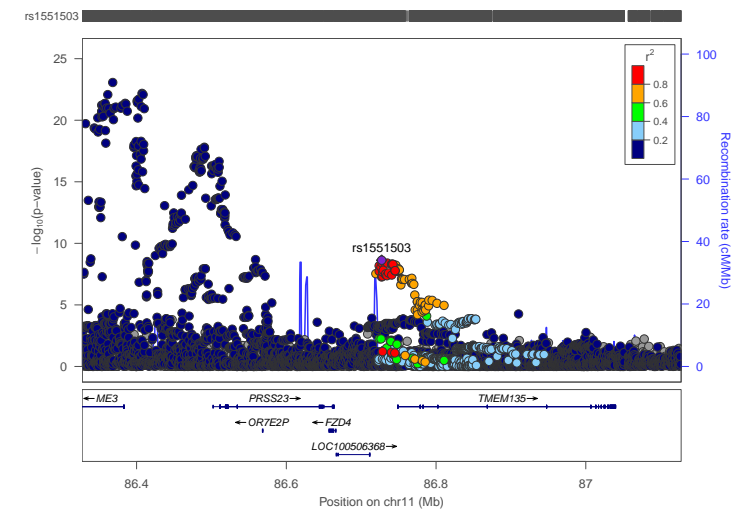

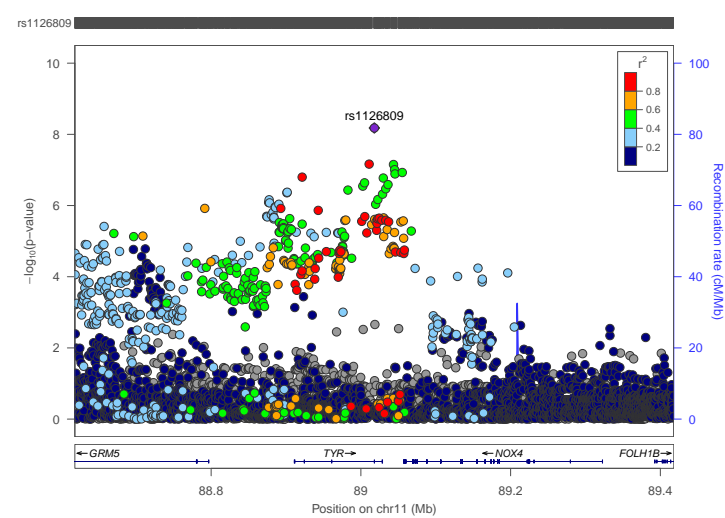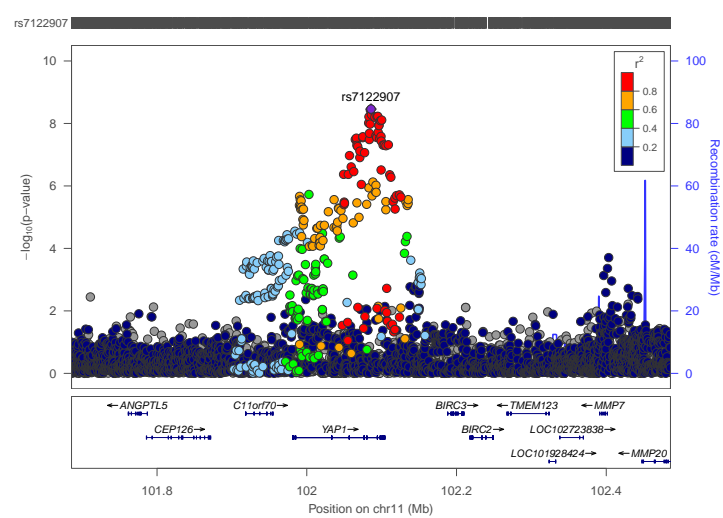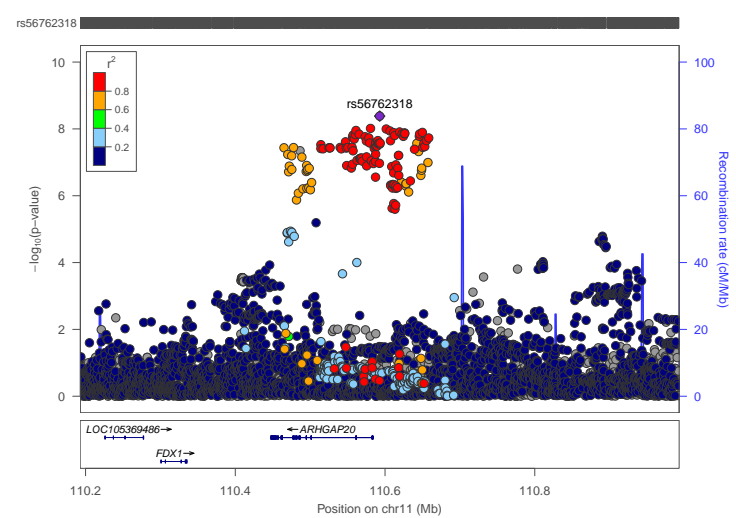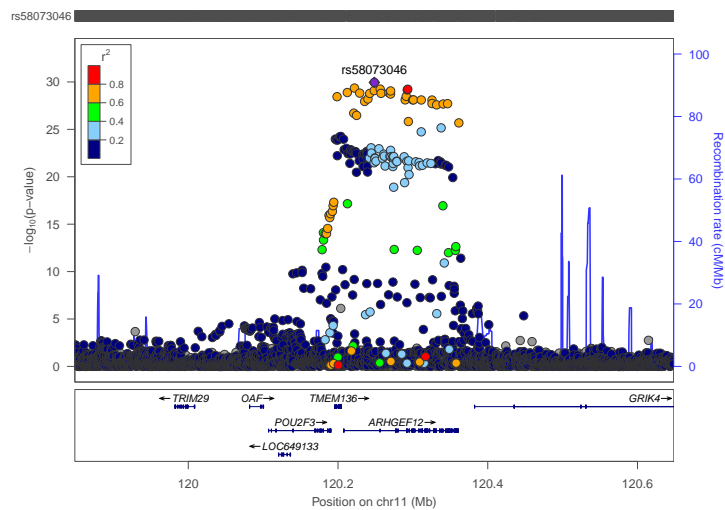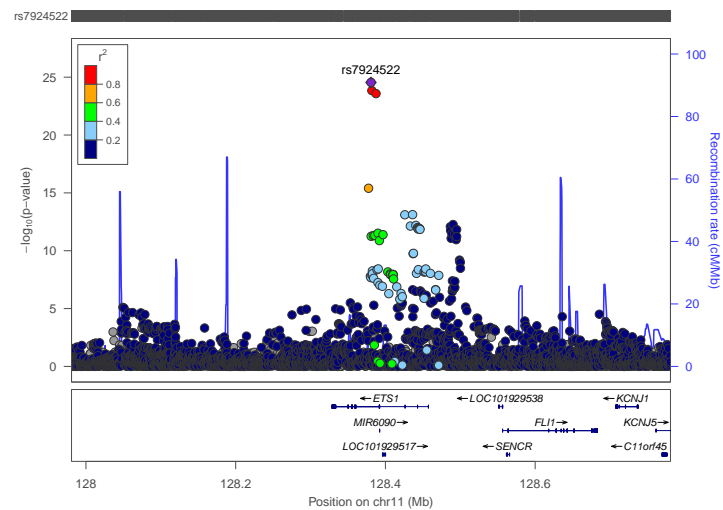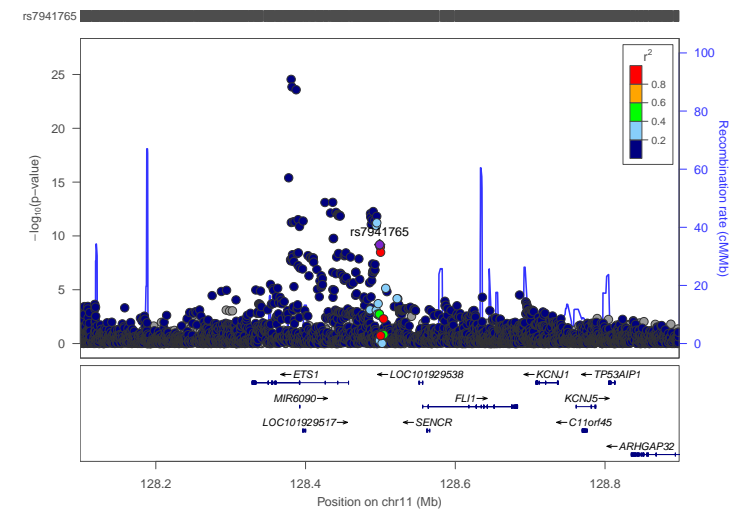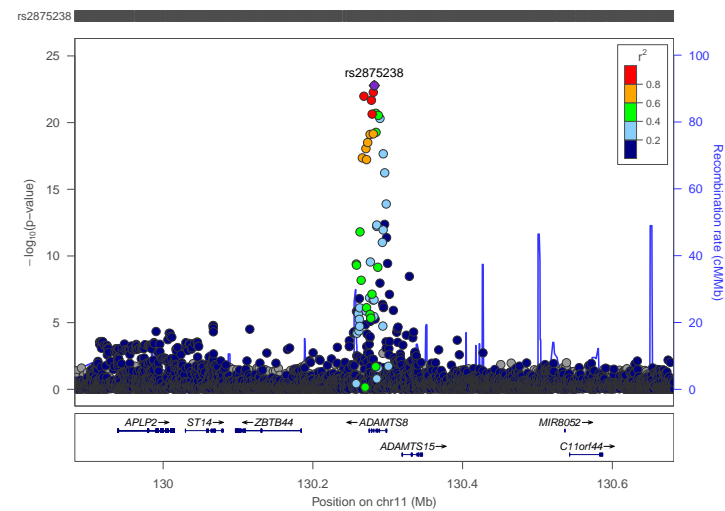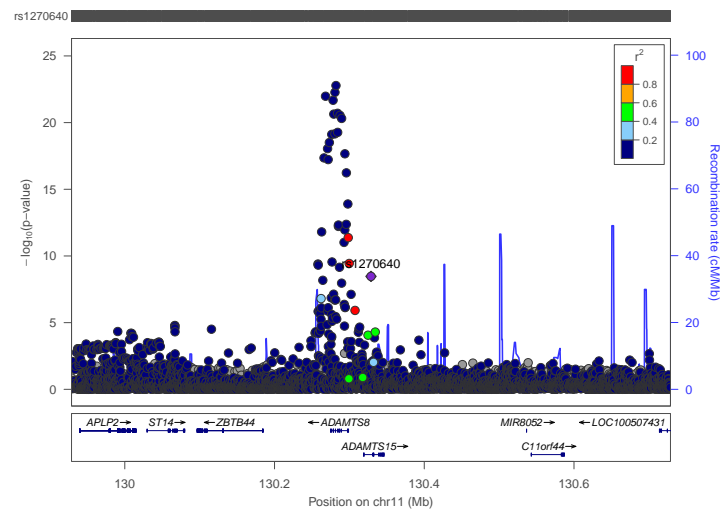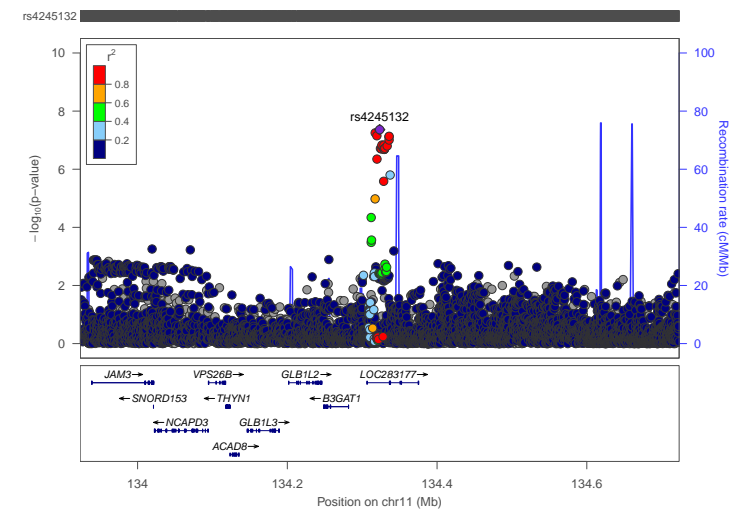

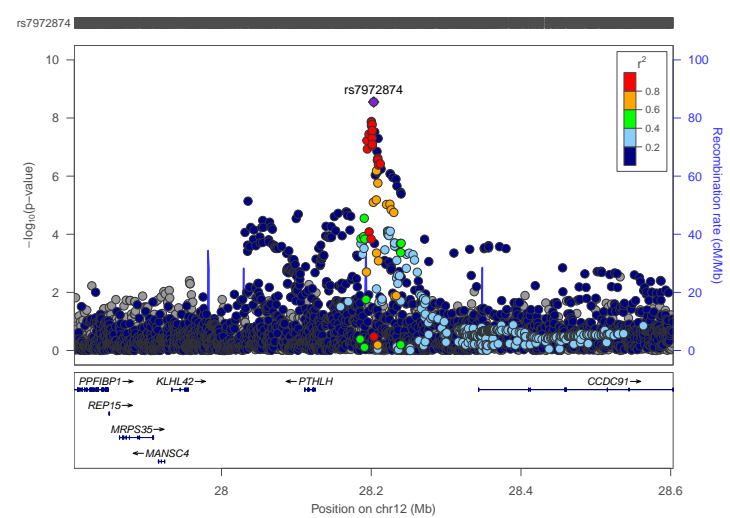

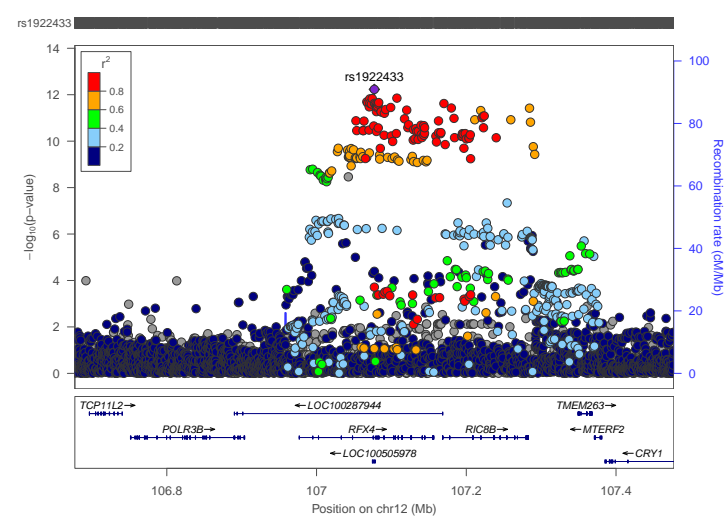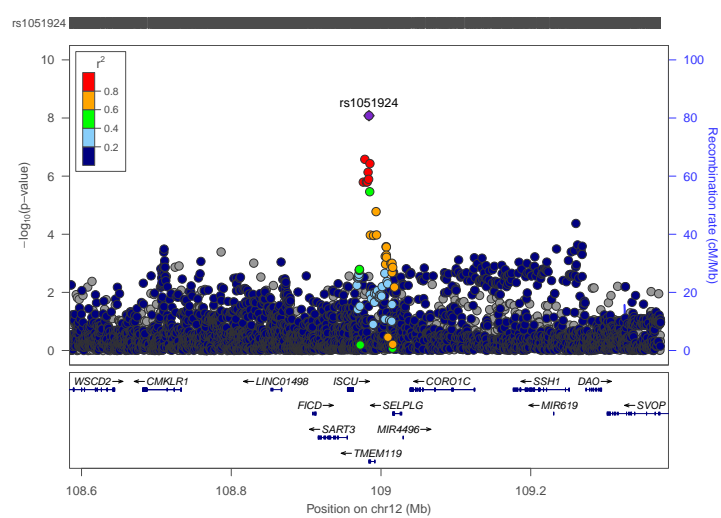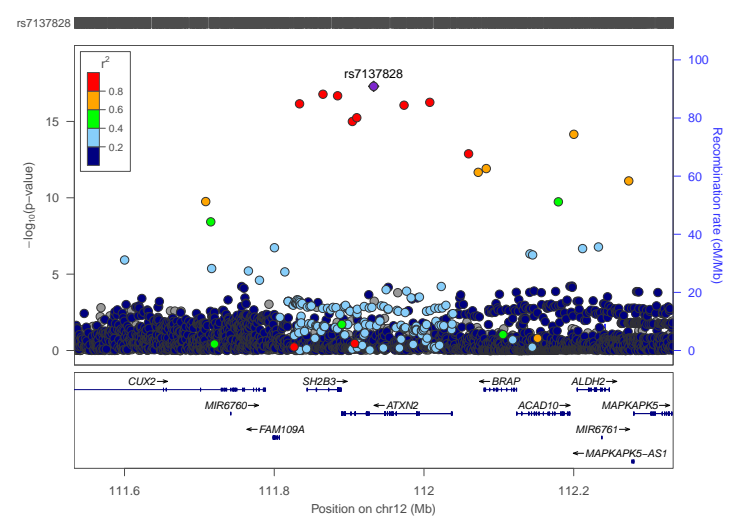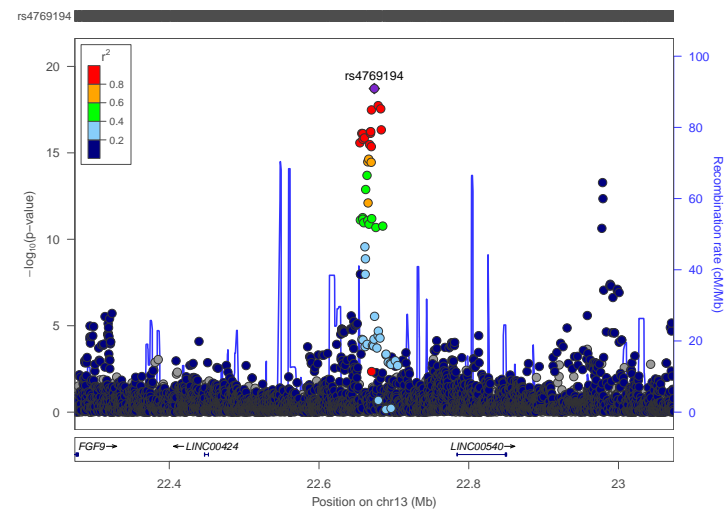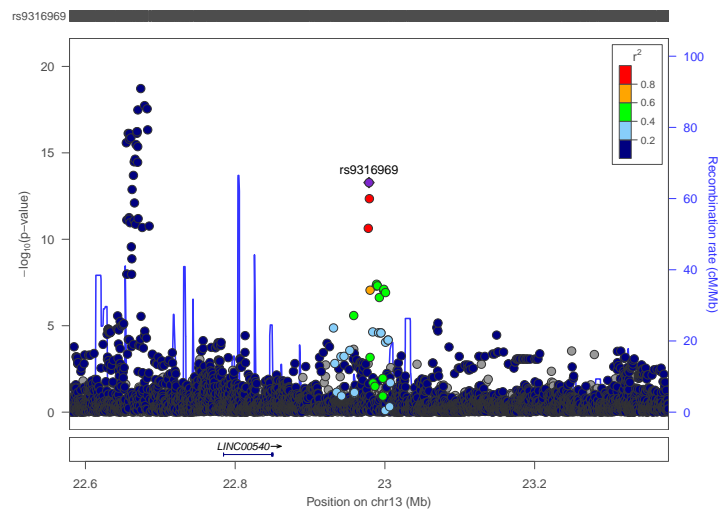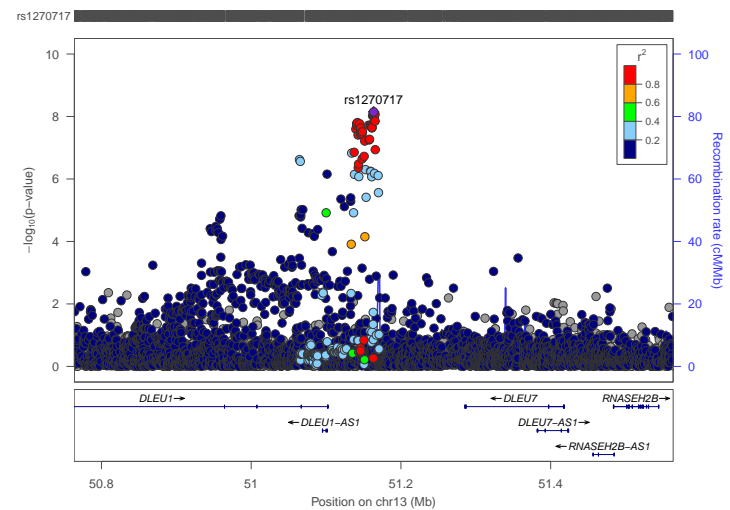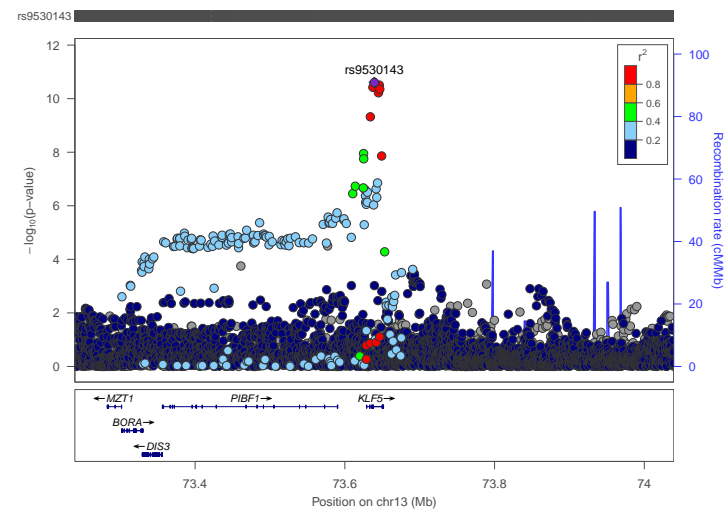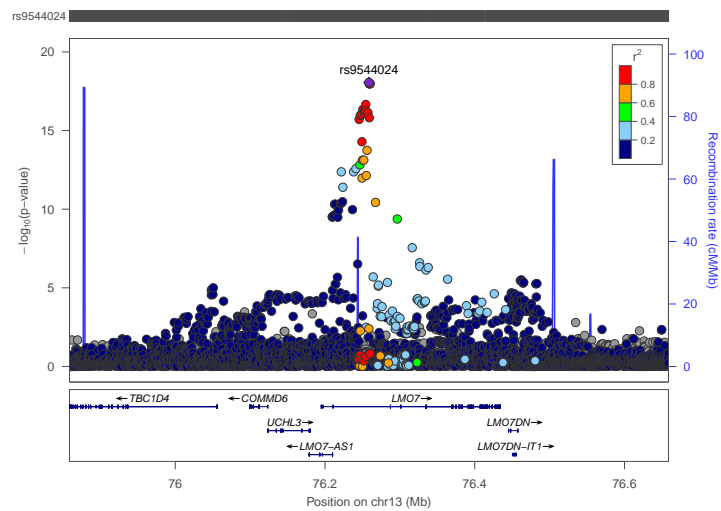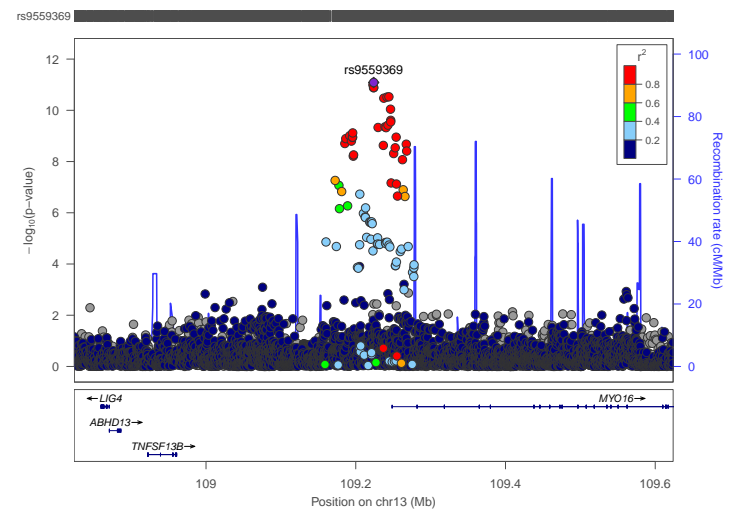

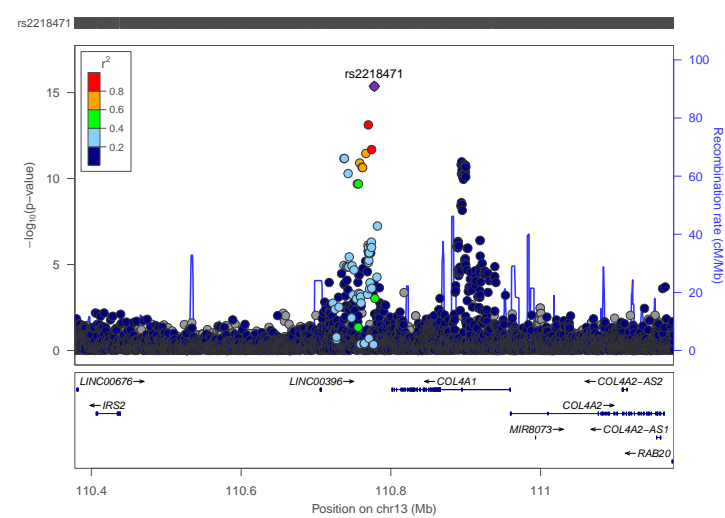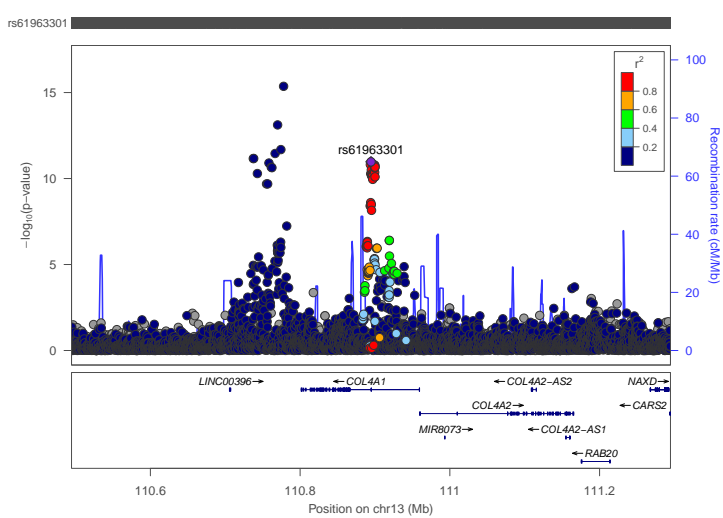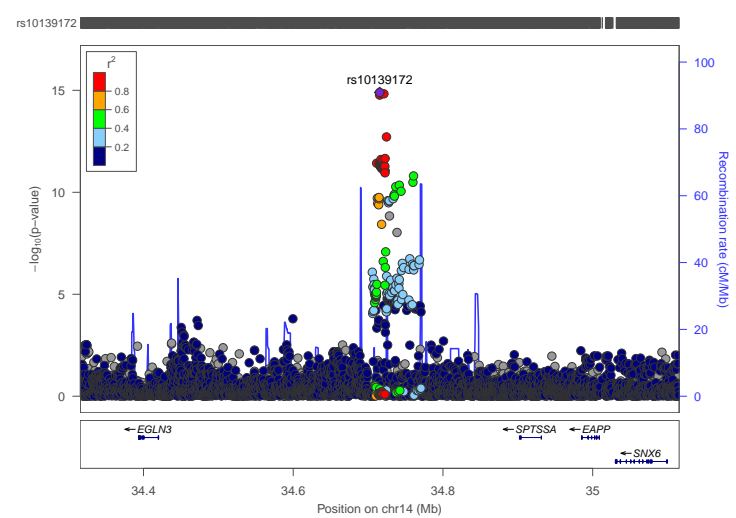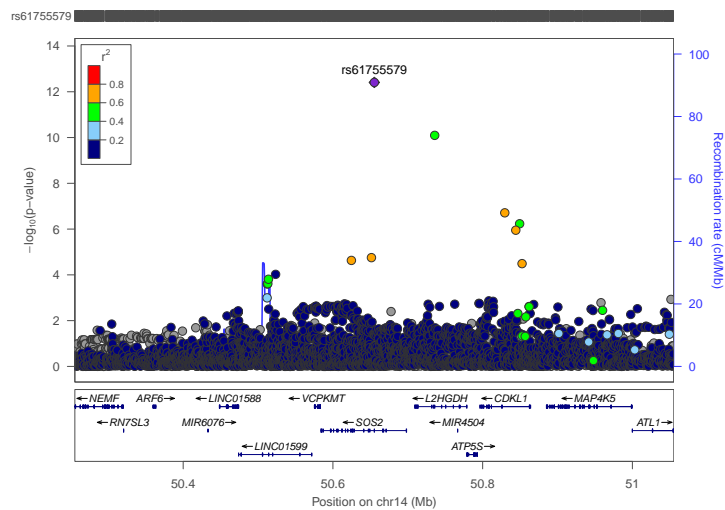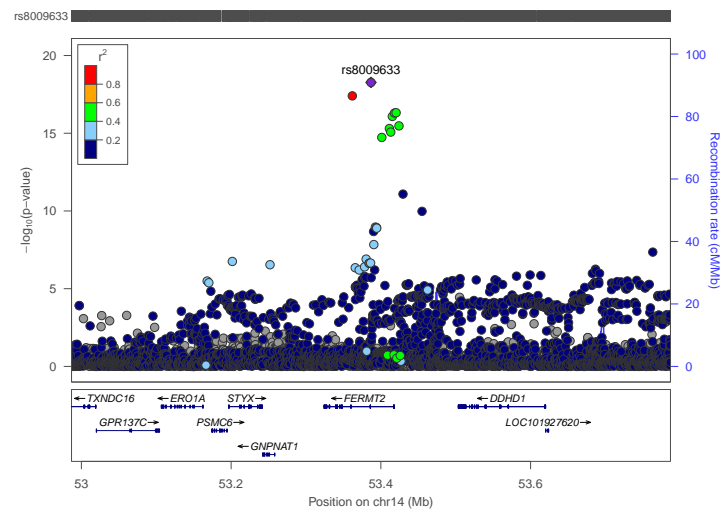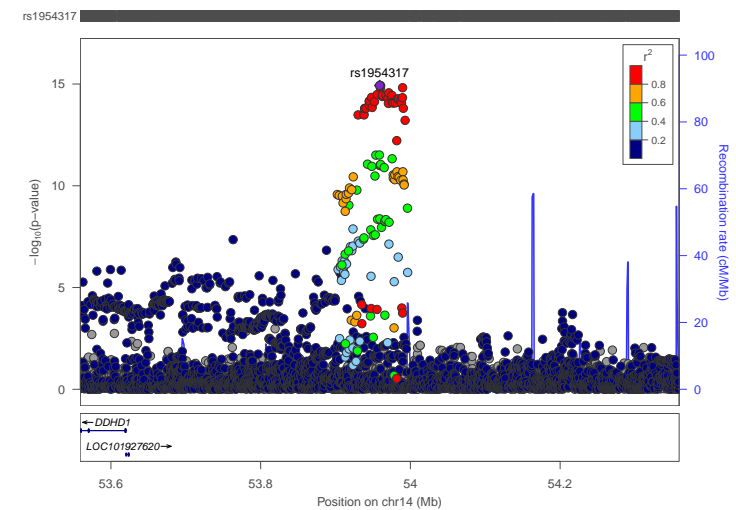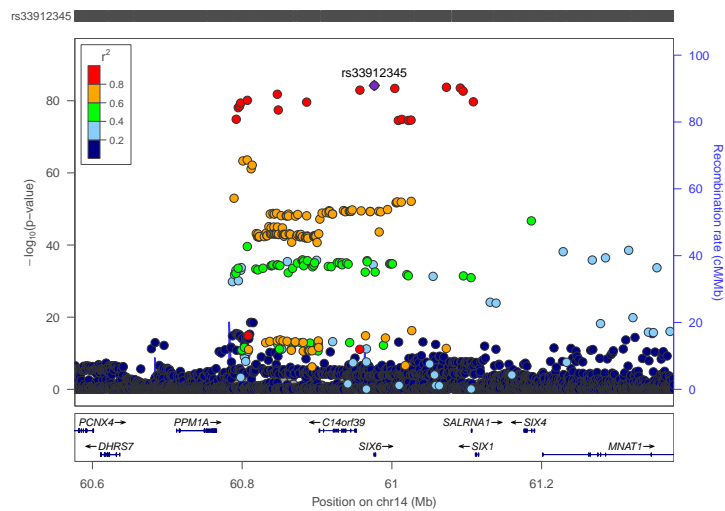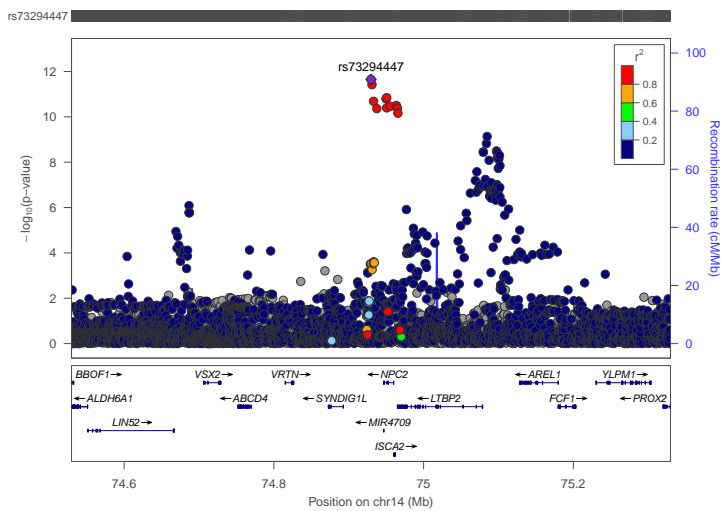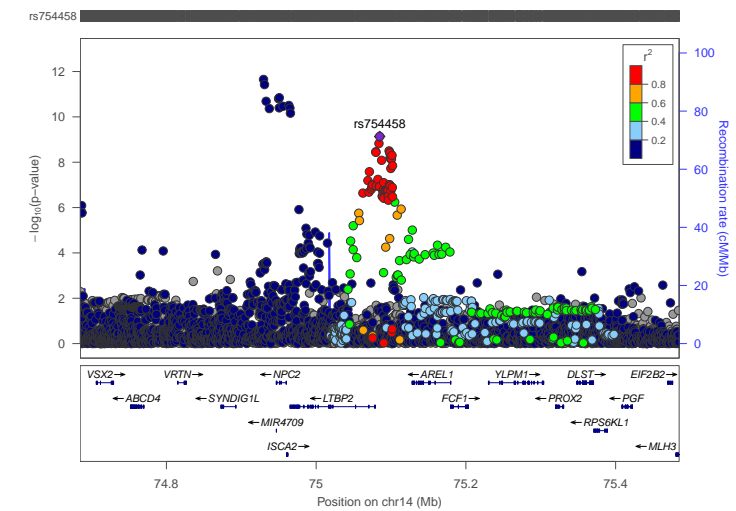

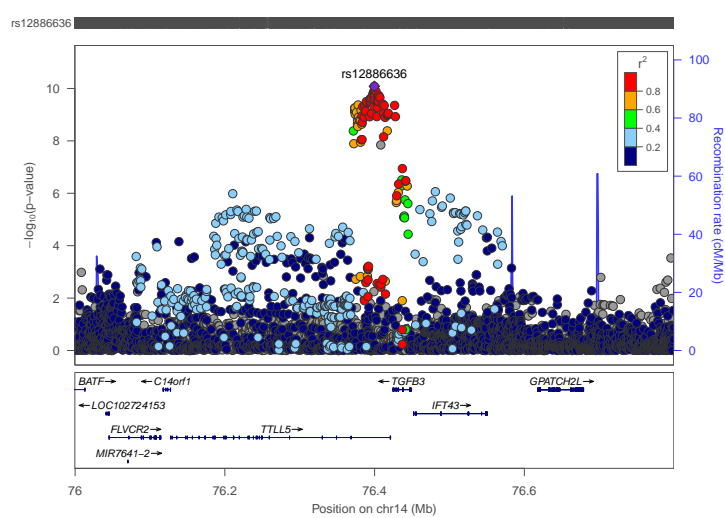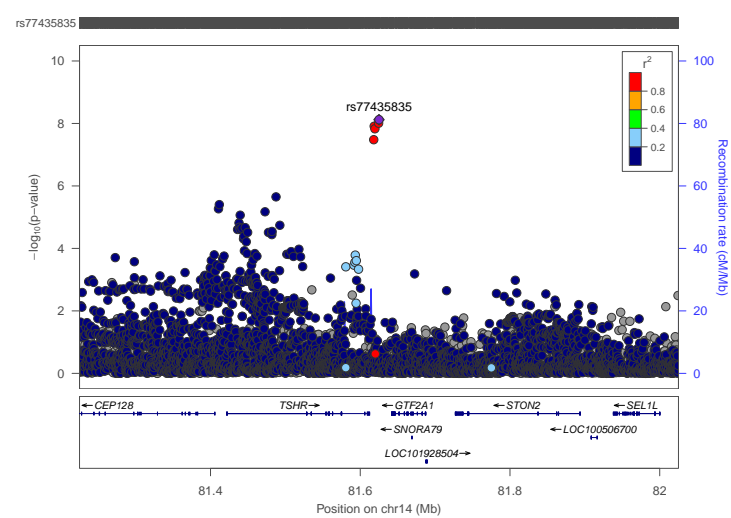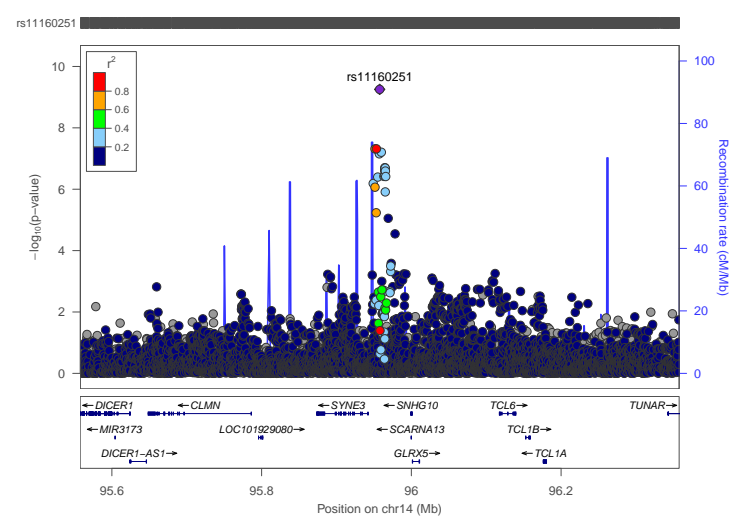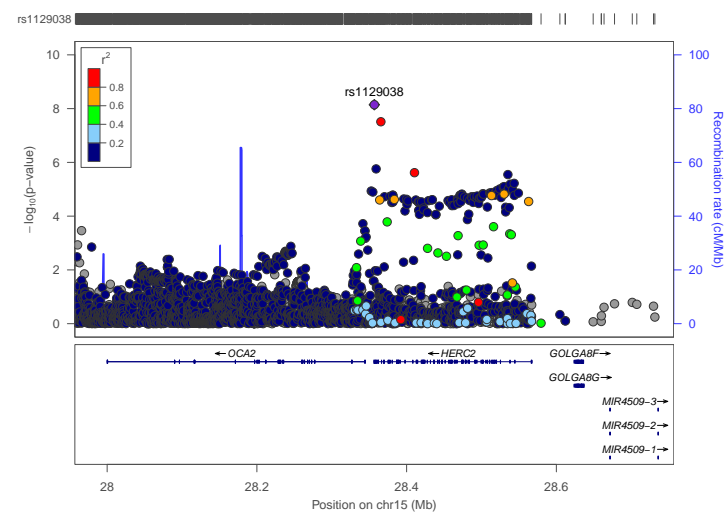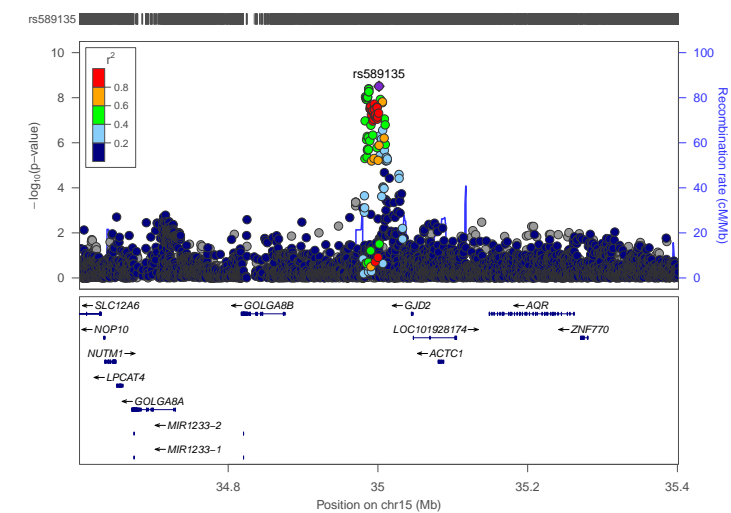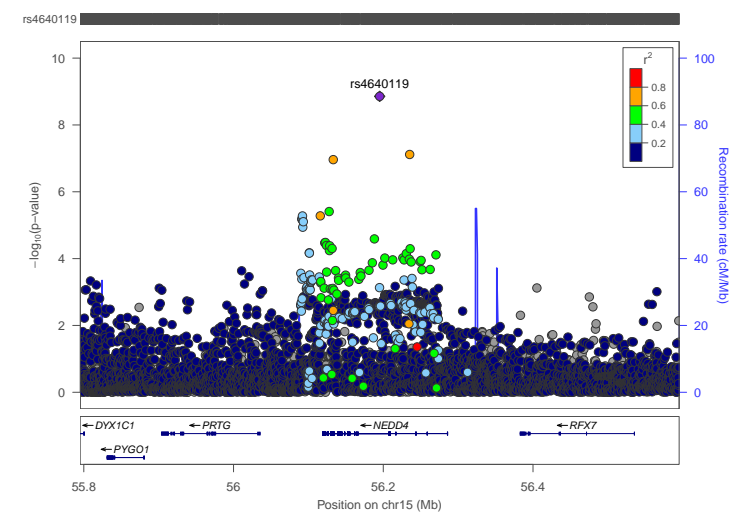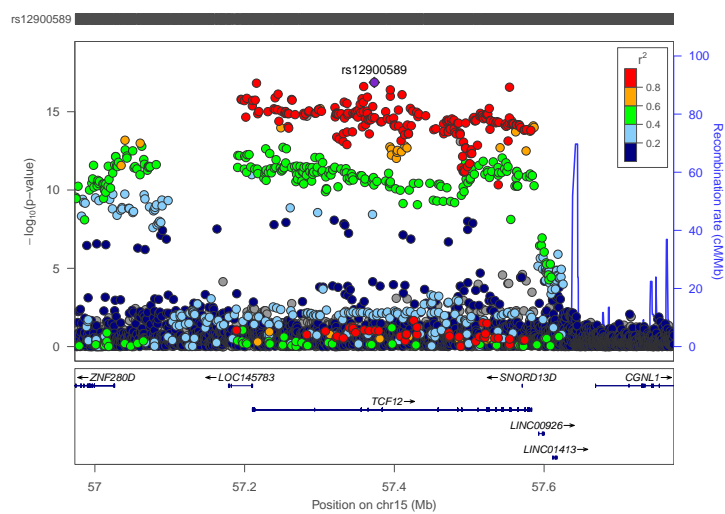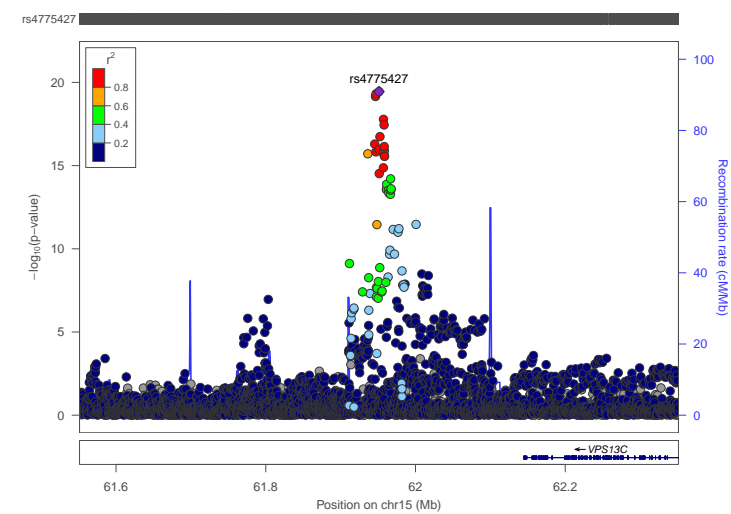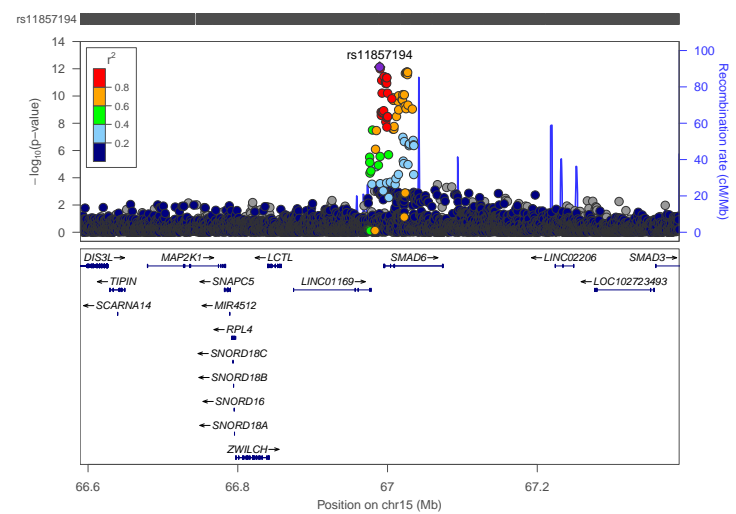

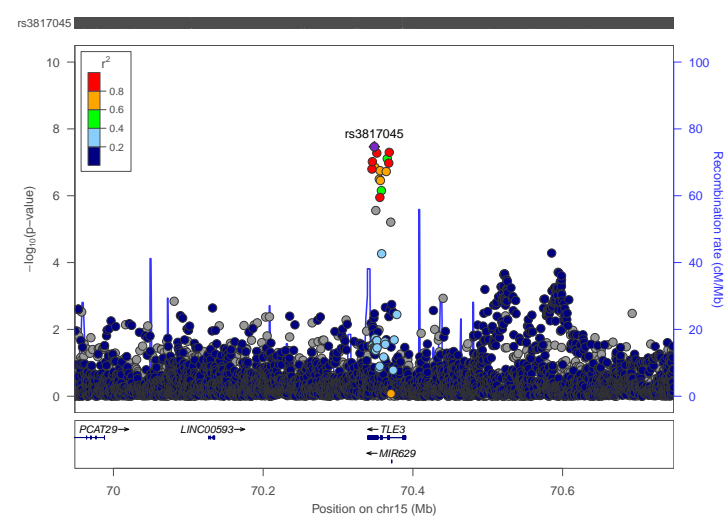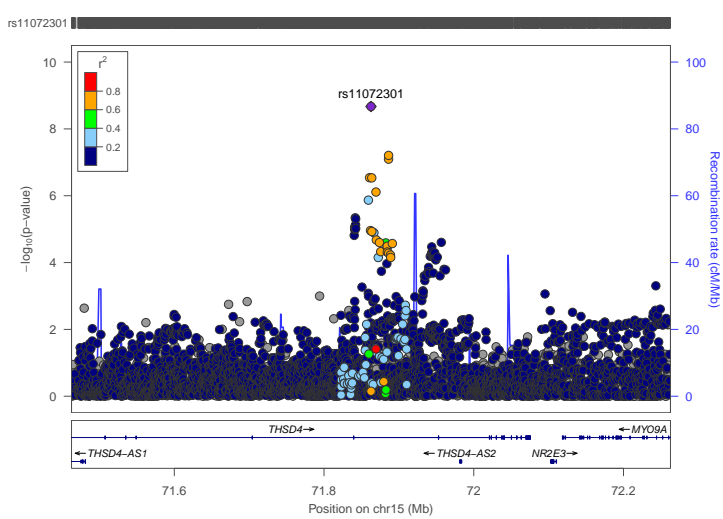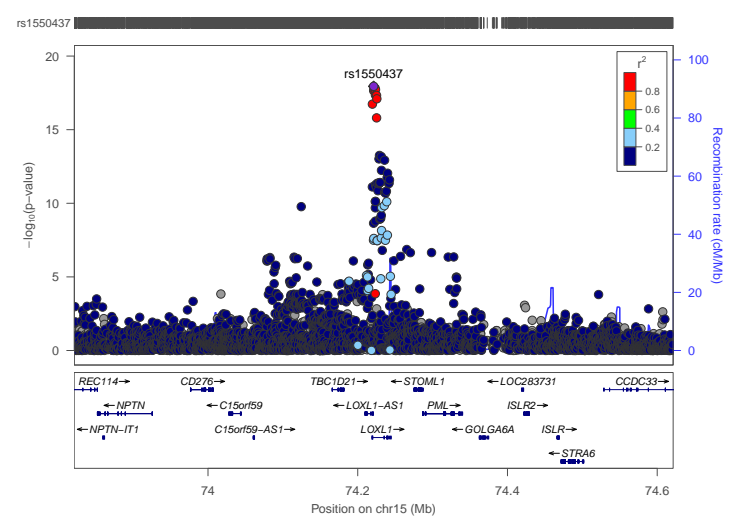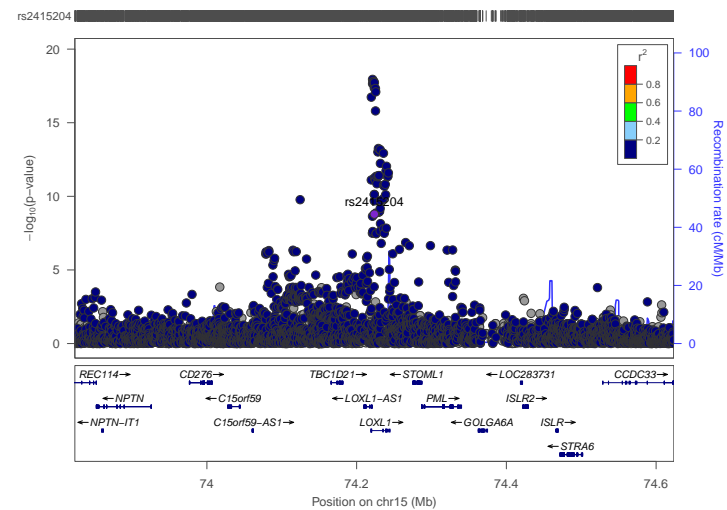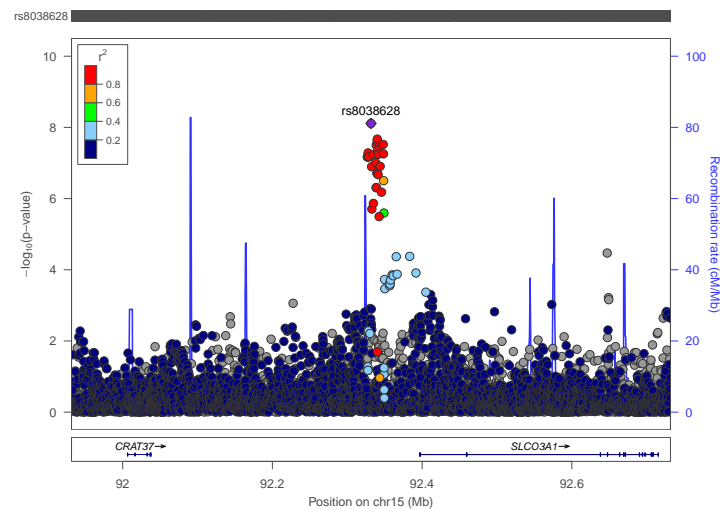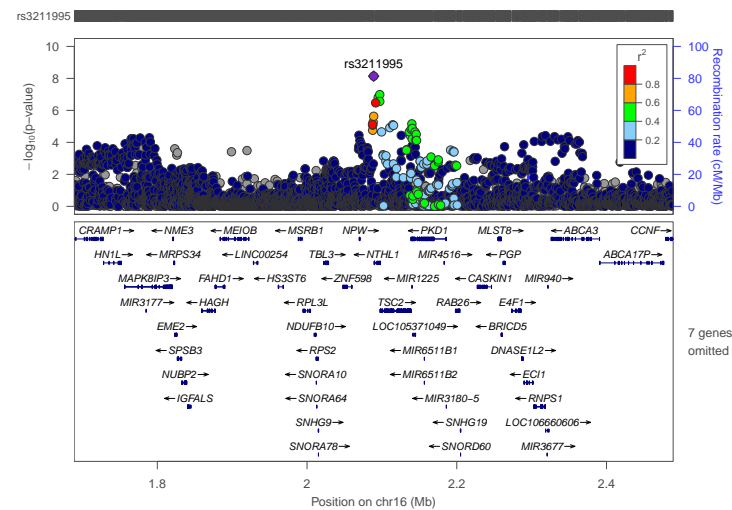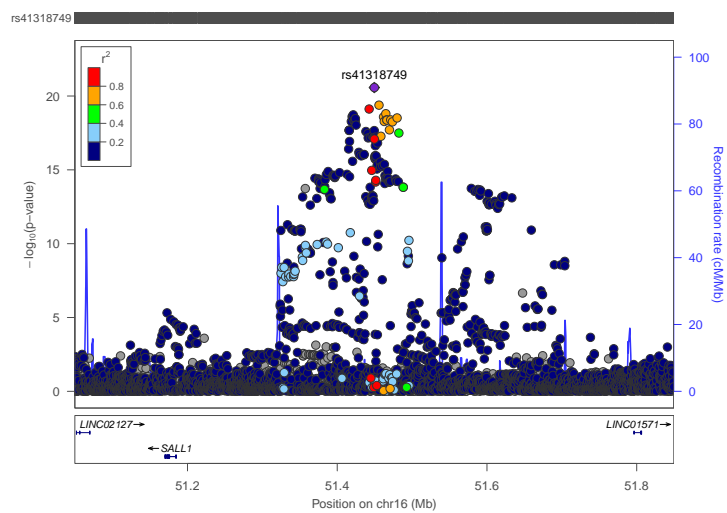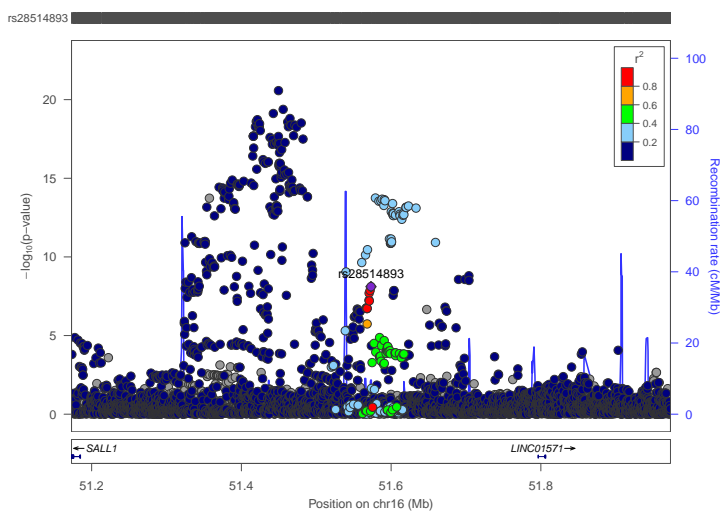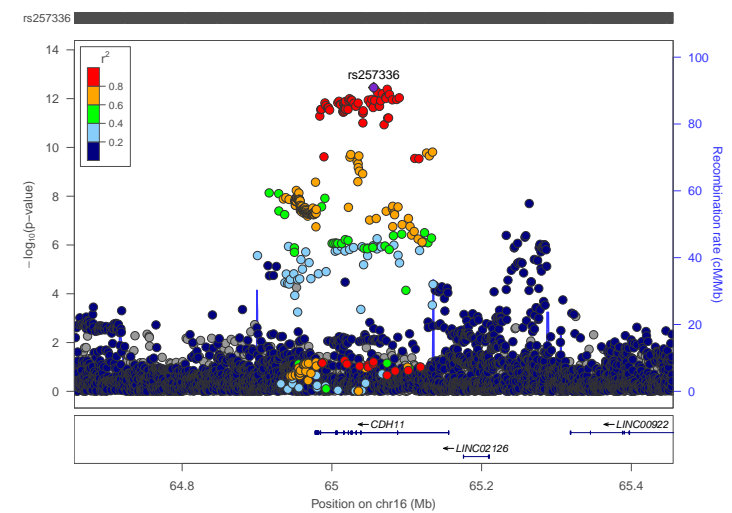

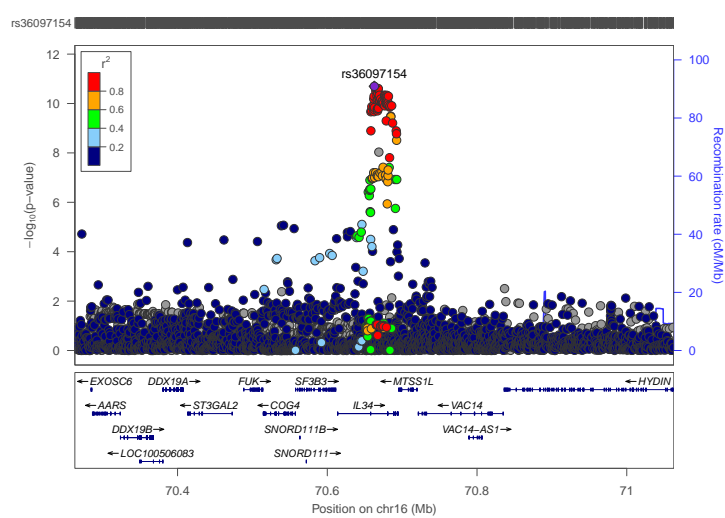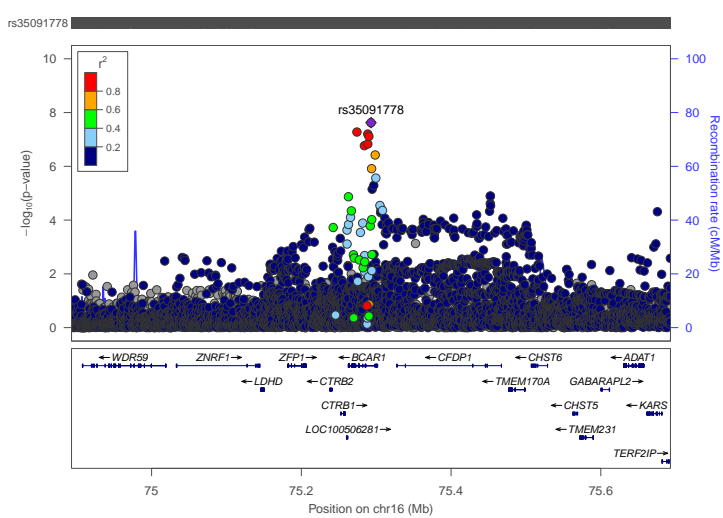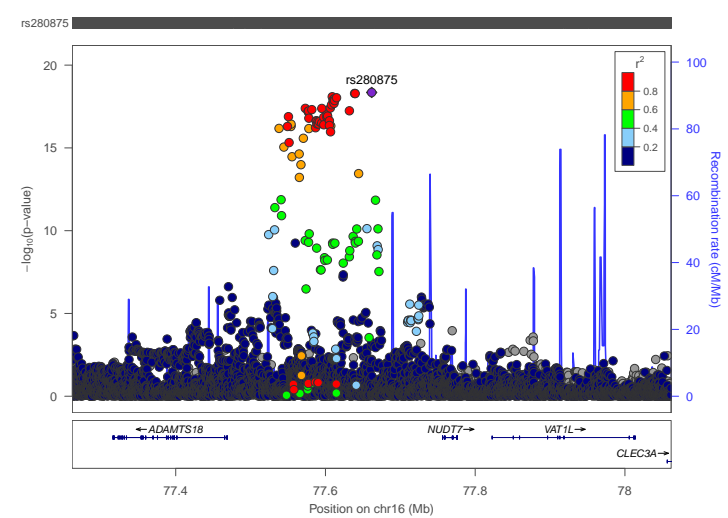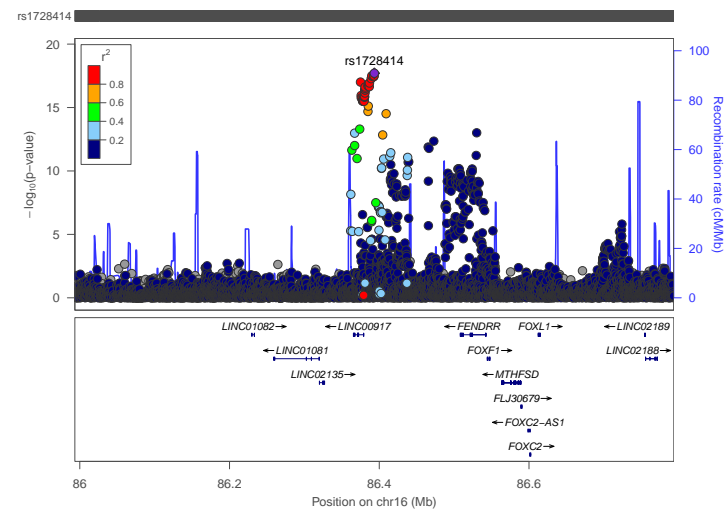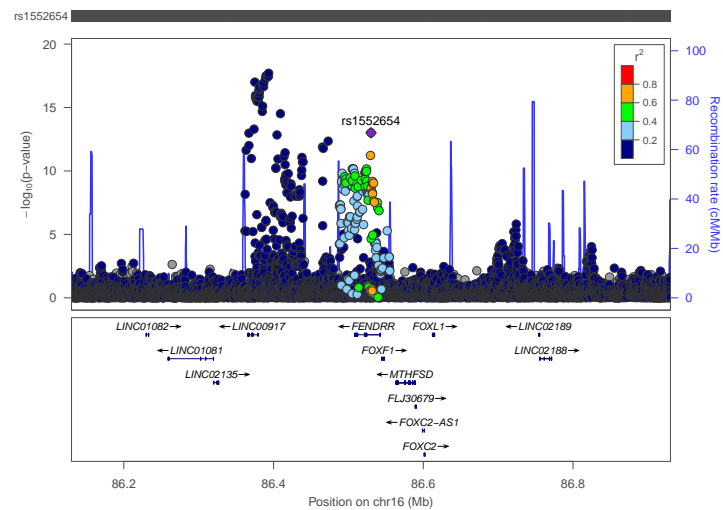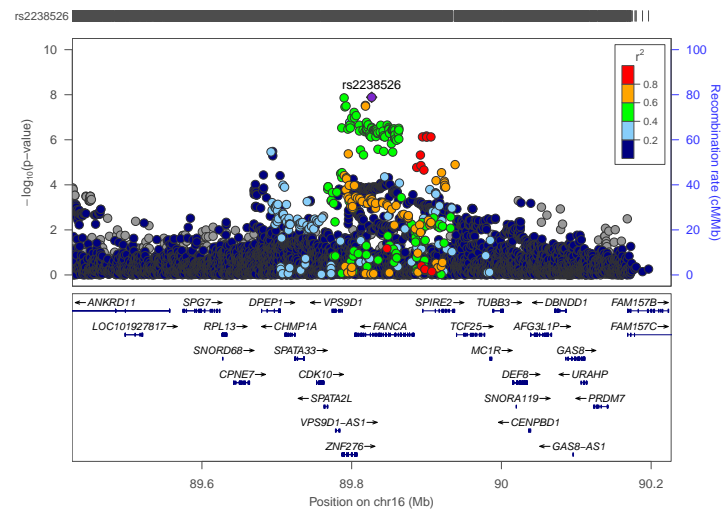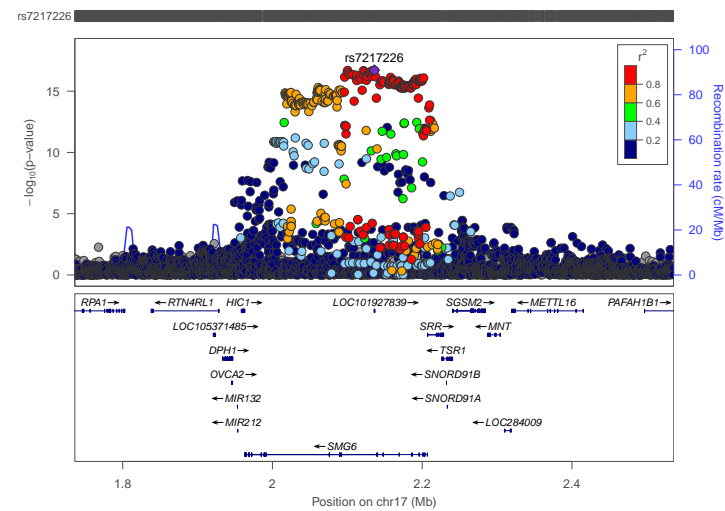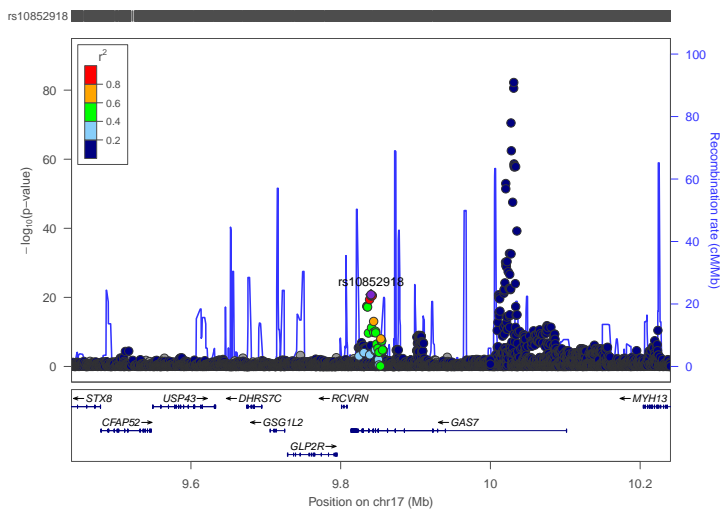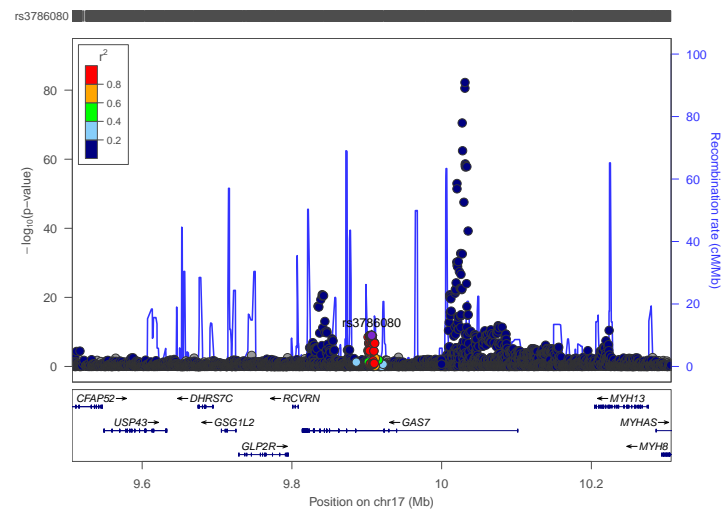

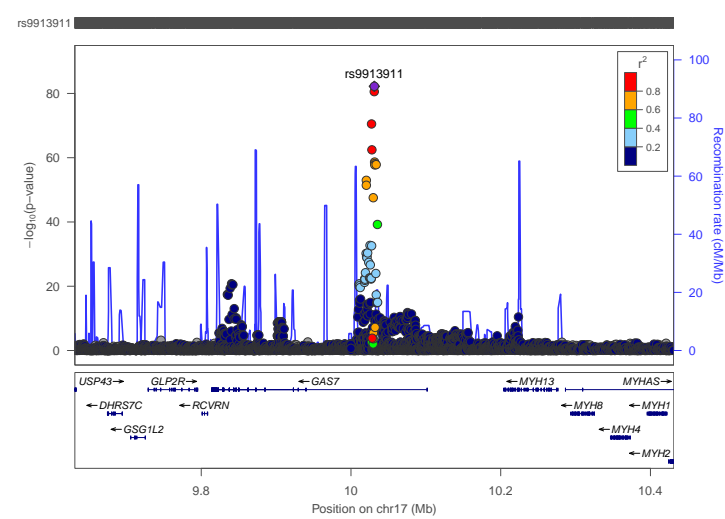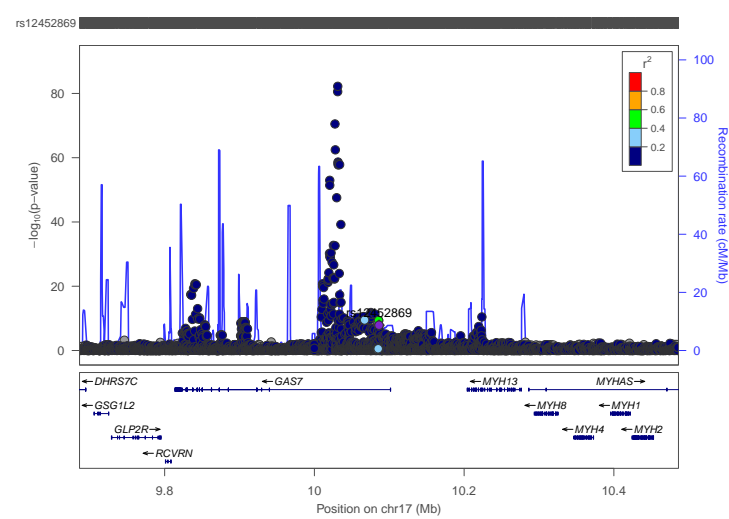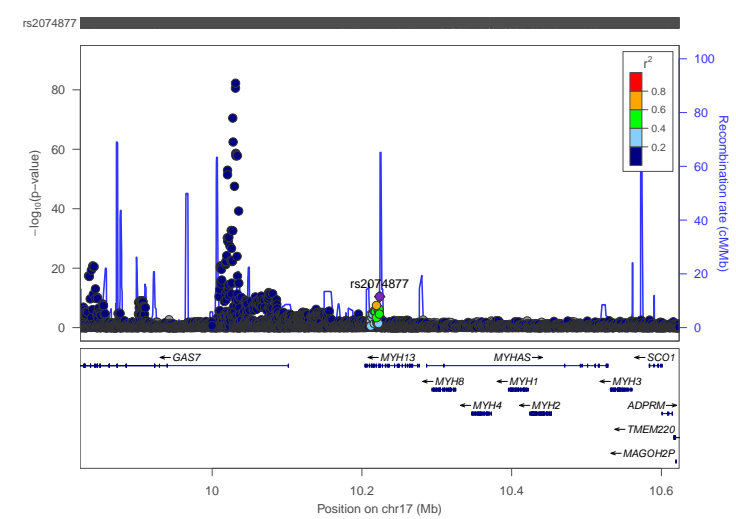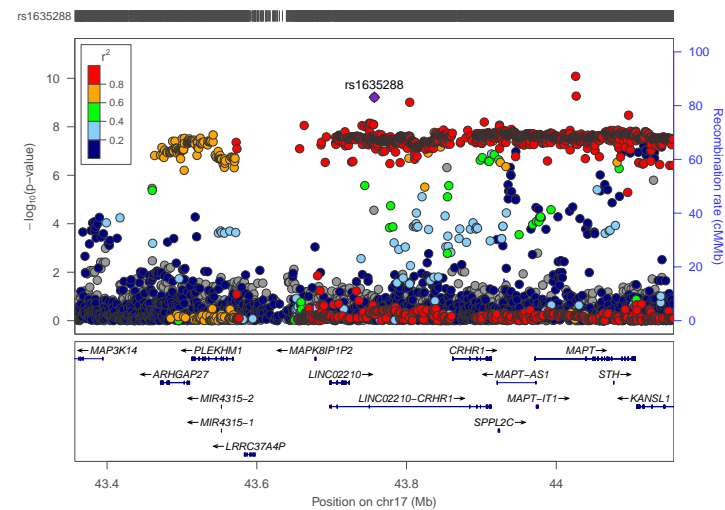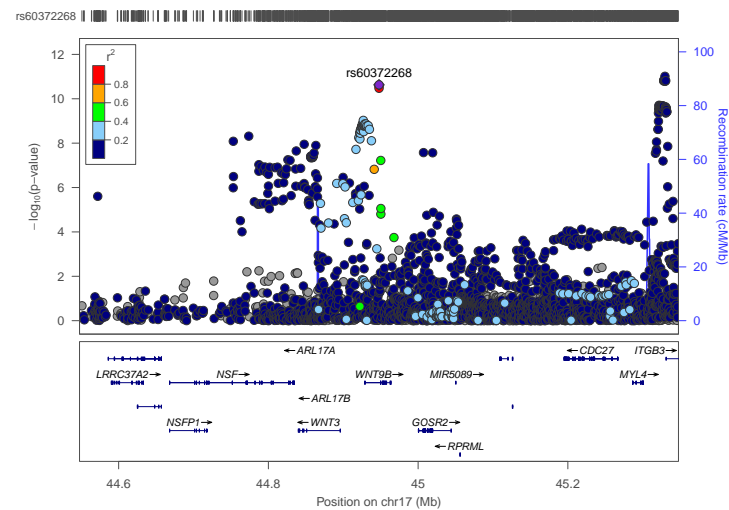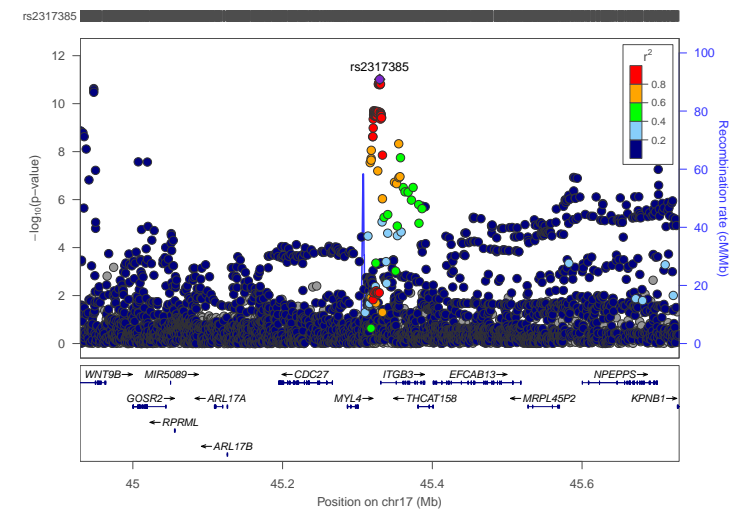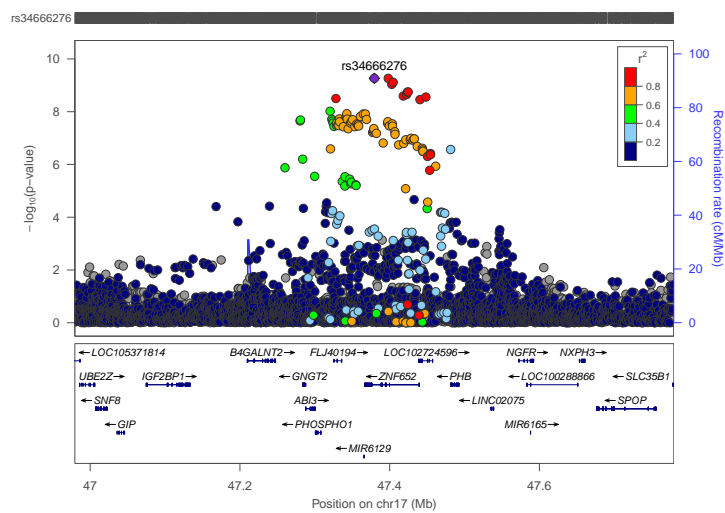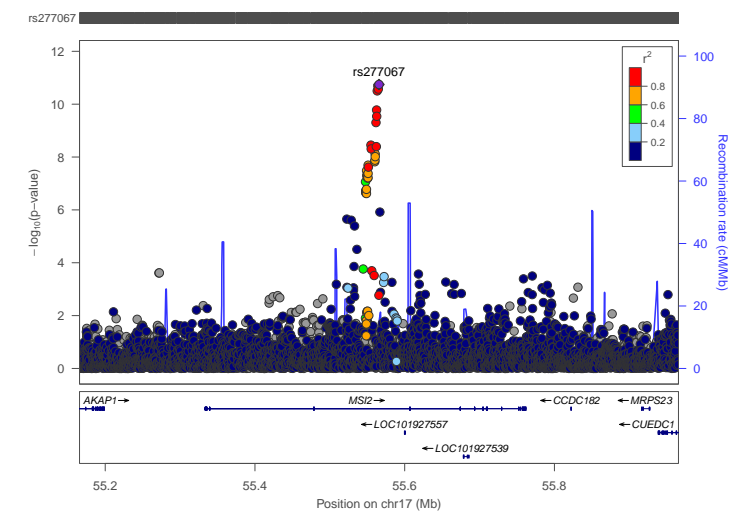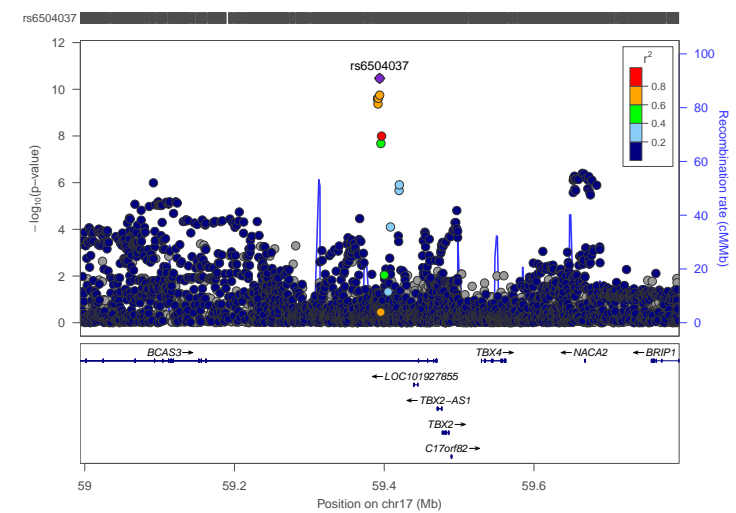

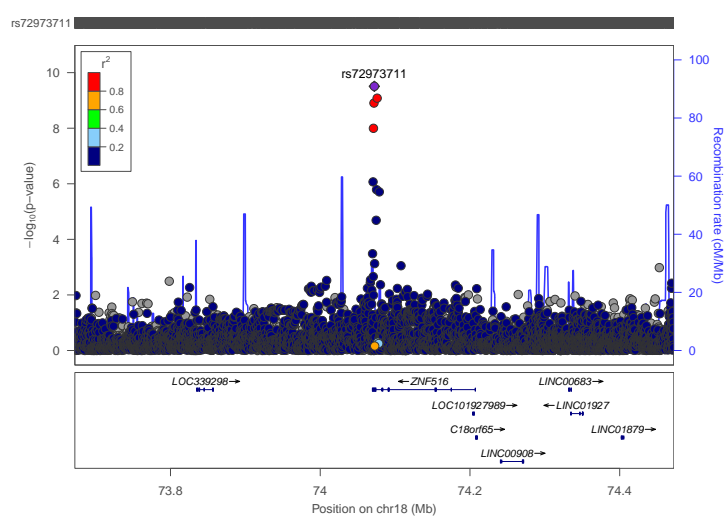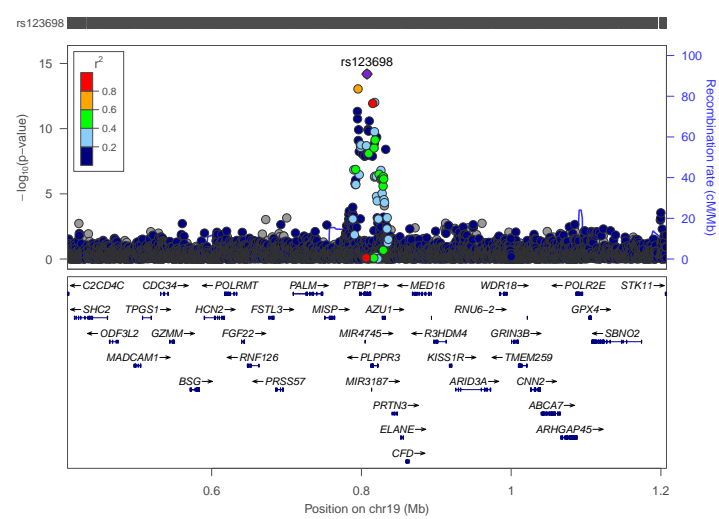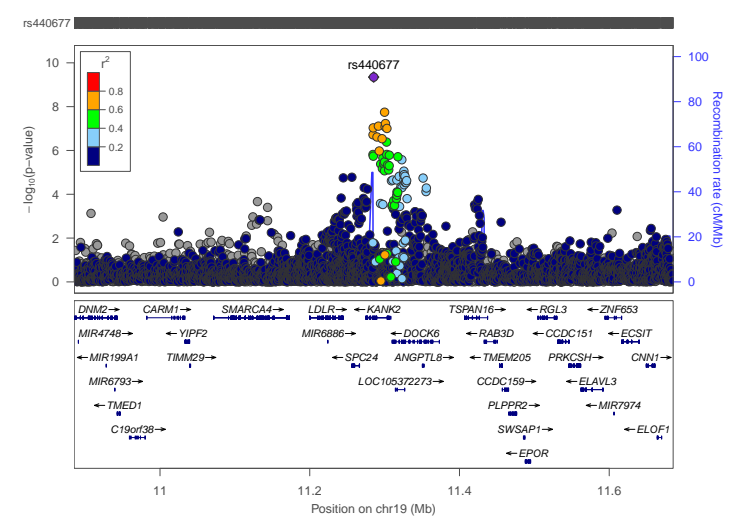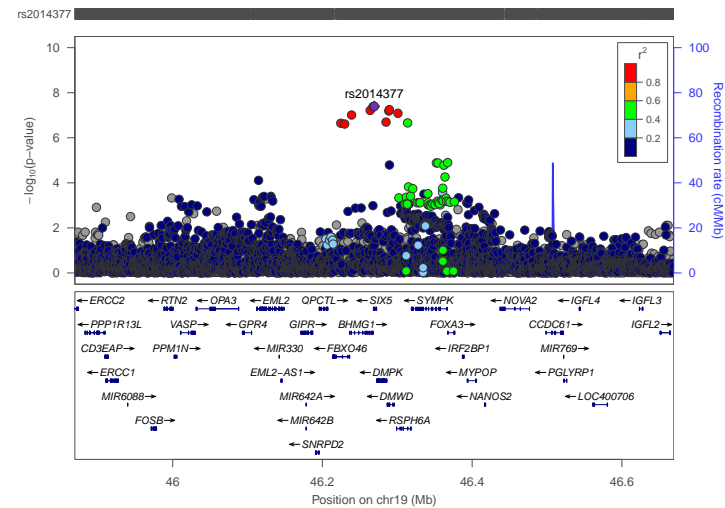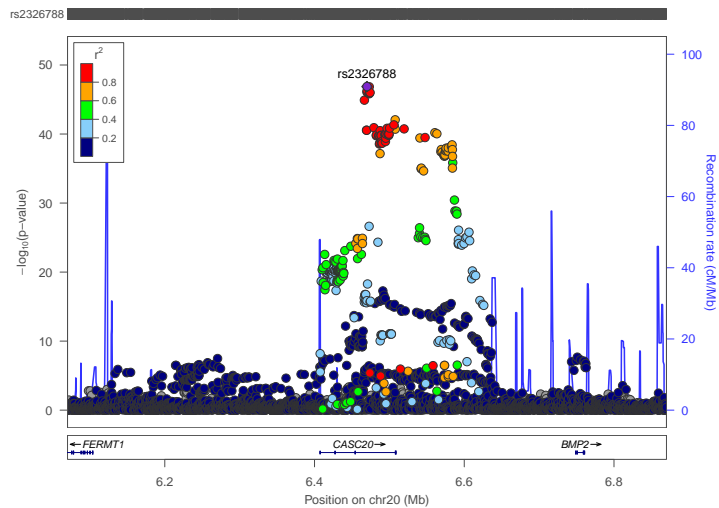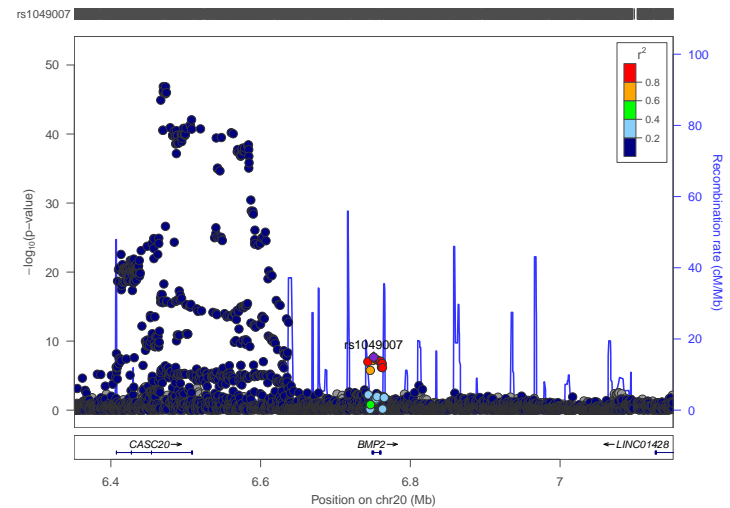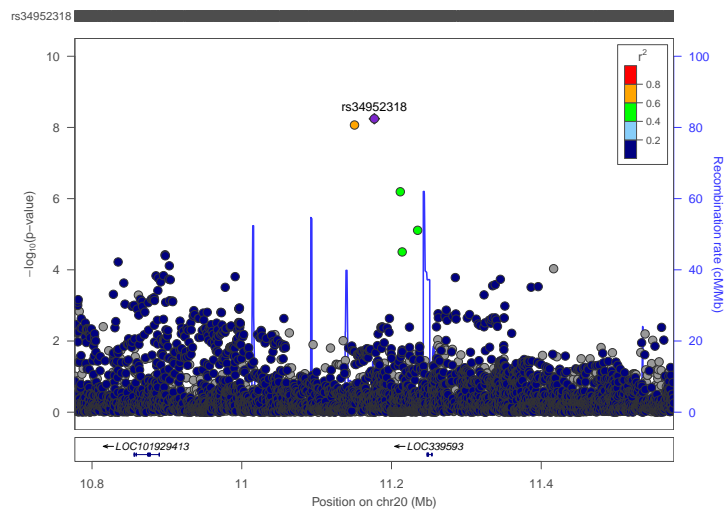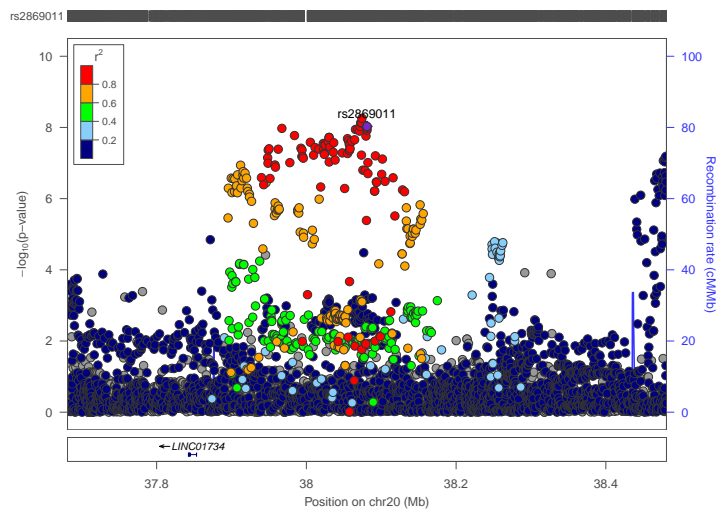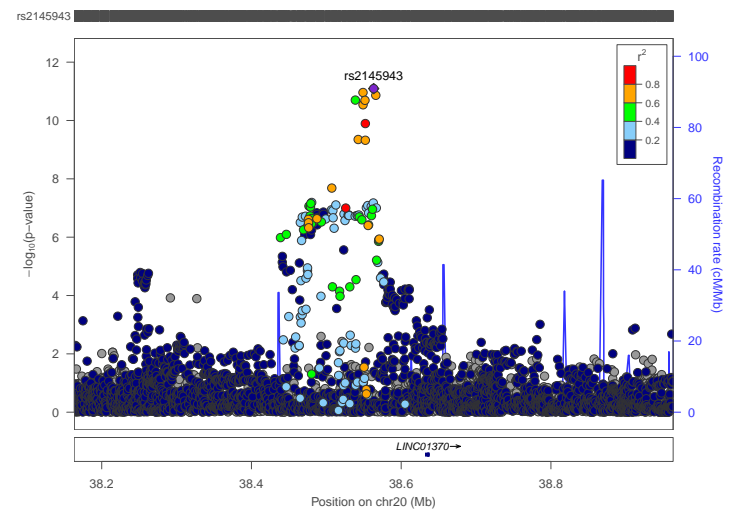

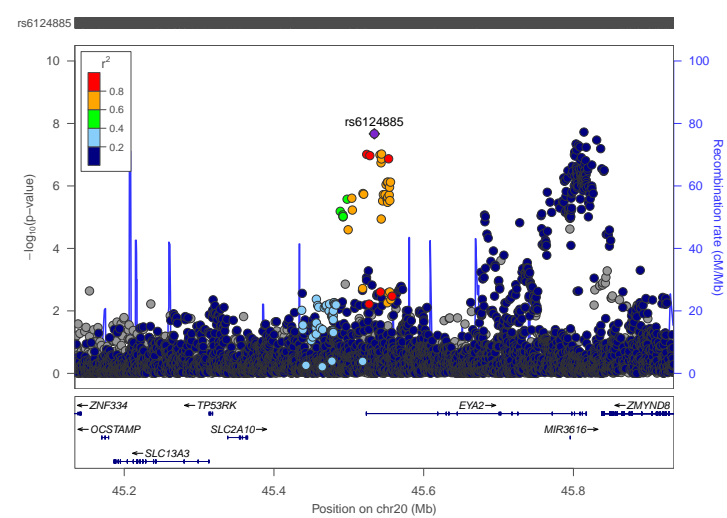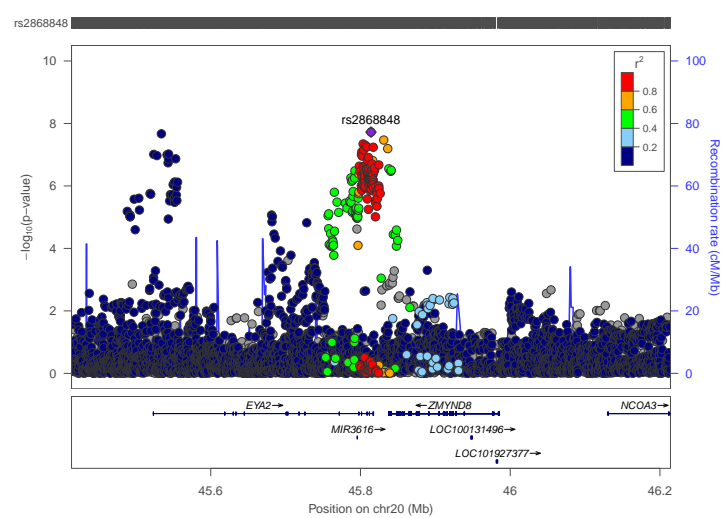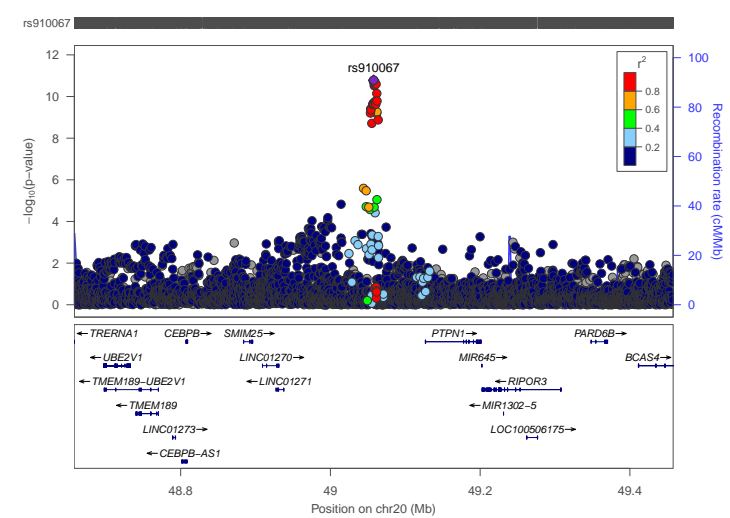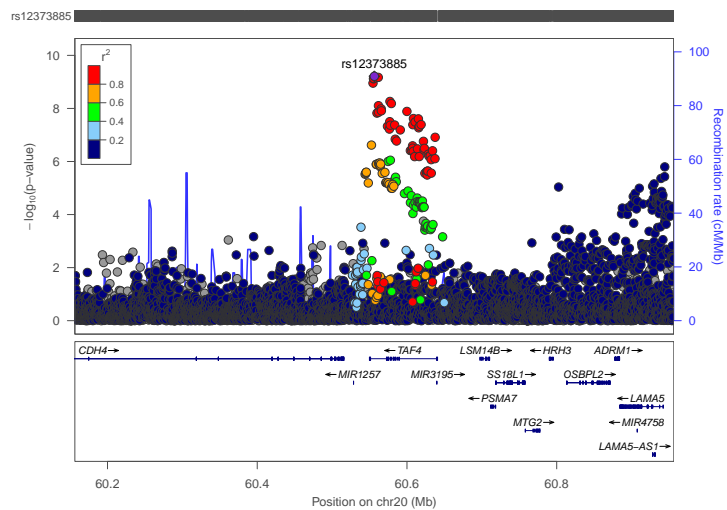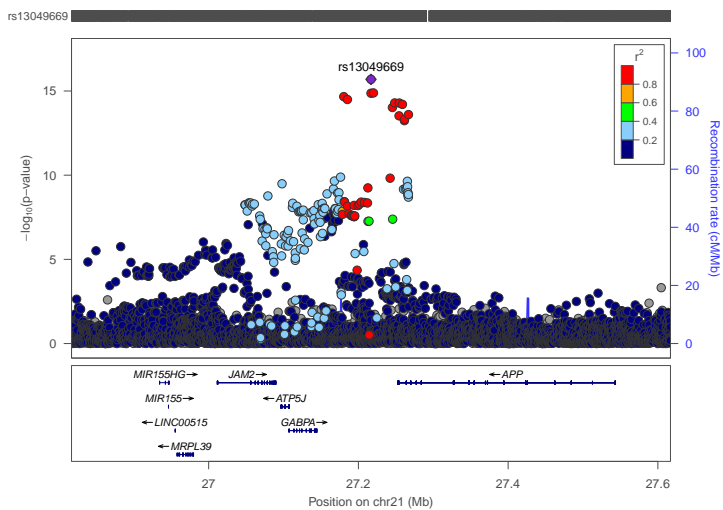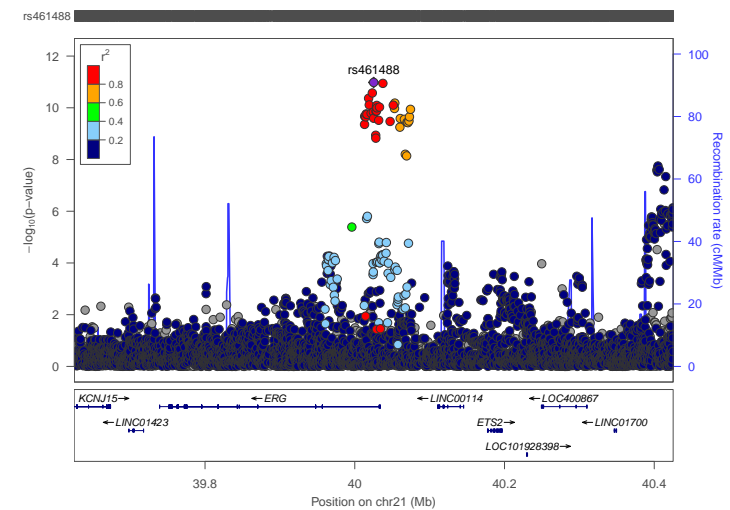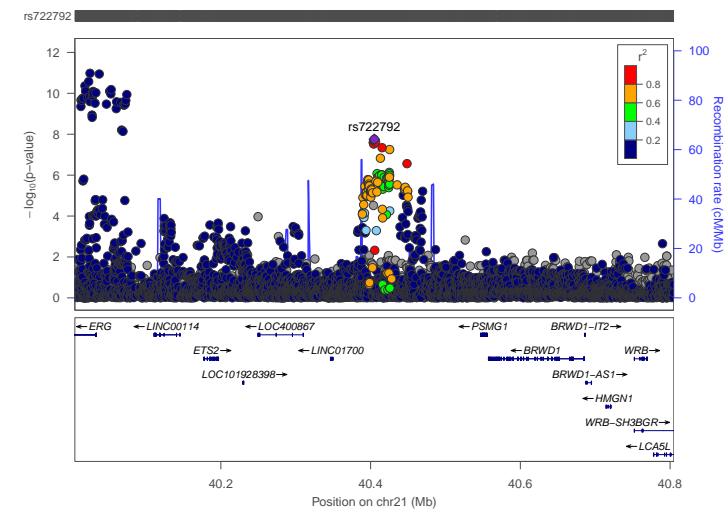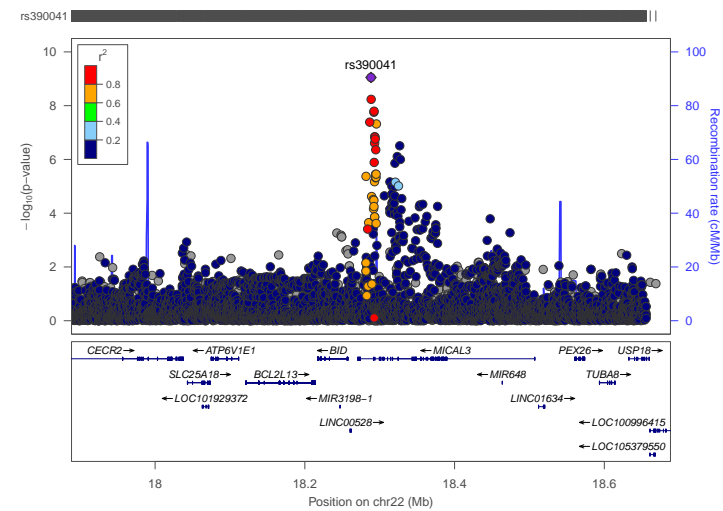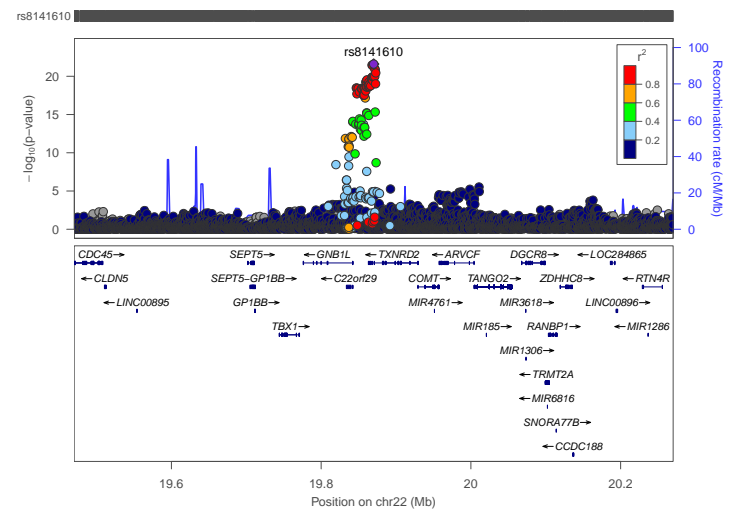

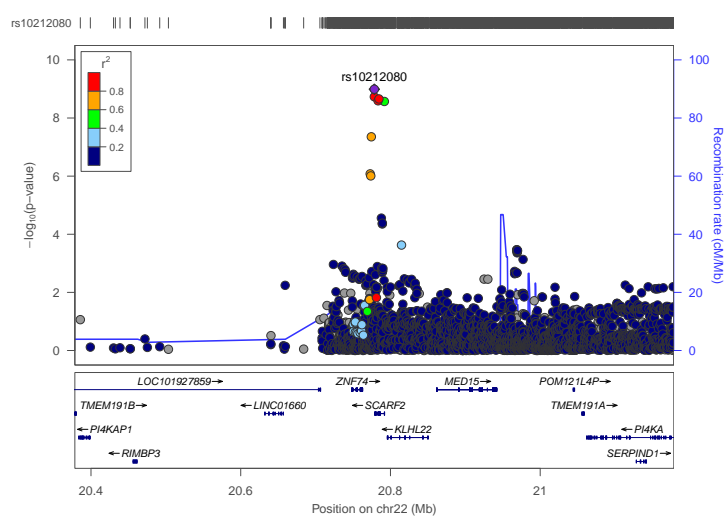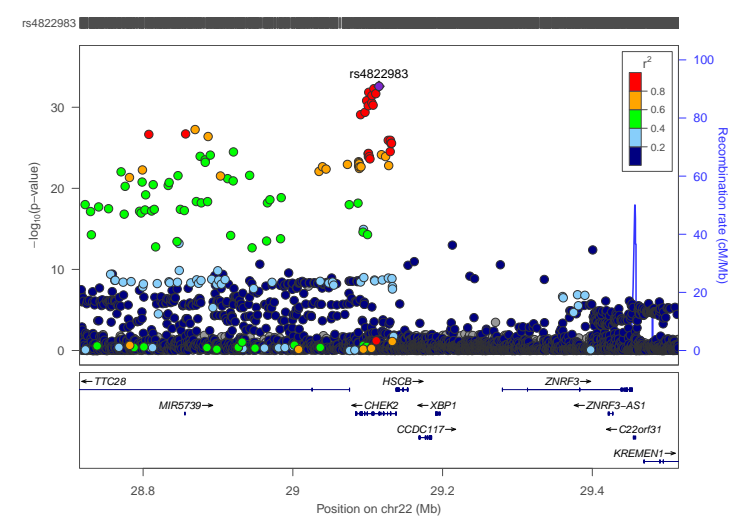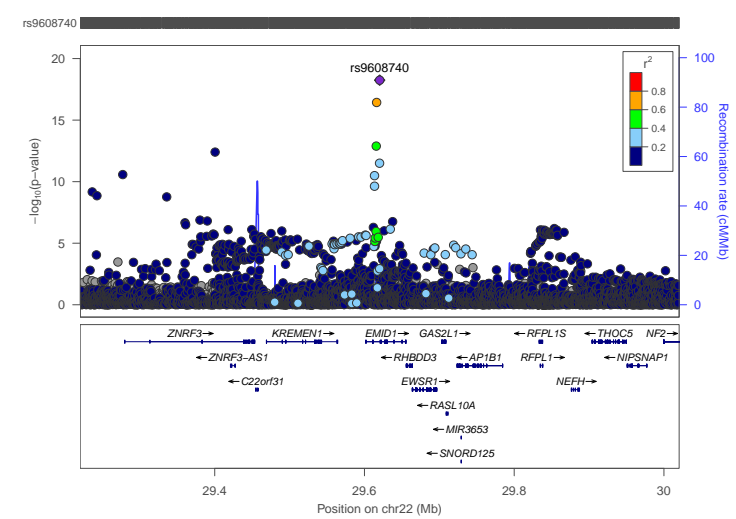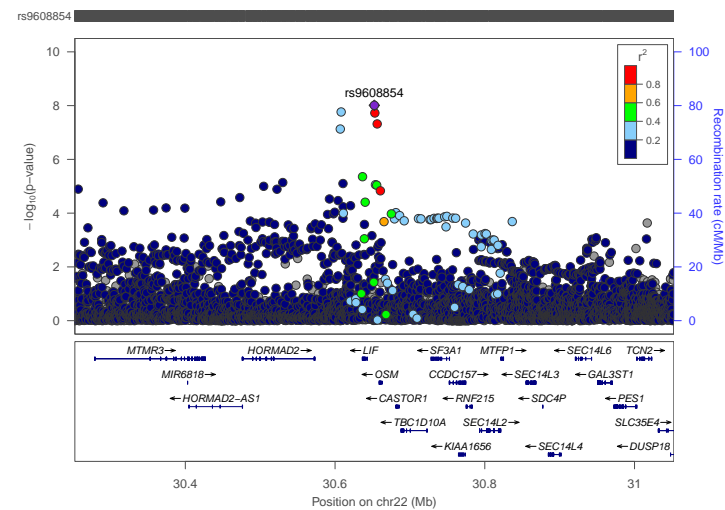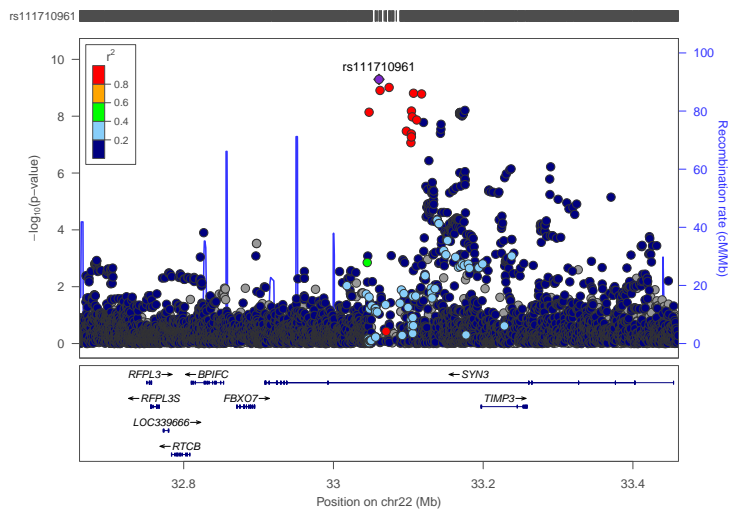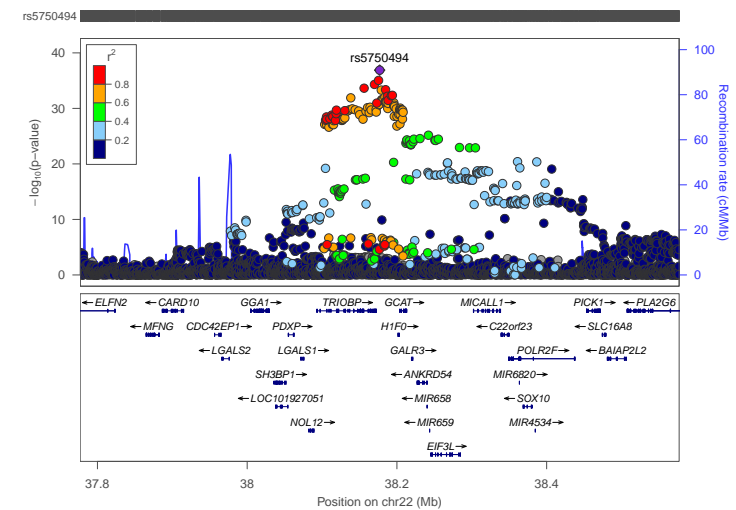

Supplement: Supplementary file 4 — LocusZoom plots for multi-ancestry meta-analysis. [file 41588_2023_1428_MOESM4_ESM.pdf]
