# Supplementary material for: Large-scale multitrait genome-wide association analyses identify hundreds of glaucoma risk loci
Source: Nat Genet. 2023 Jun 29;55(7):1116–25. doi: 10.1038/s41588-023-01428-5 (PMC10335935; doi:10.1038/s41588-023-01428-5)
Supplement: Supplementary file 6 — Consortium members. [file 41588_2023_1428_MOESM6_ESM.pdf]

**Members of the 23andMe Research Team**

Stella Aslibekyan, Adam Auton, Elizabeth Babalola, Robert K. Bell, Jessica Bielenberg, Katarzyna Bryc, Emily Bullis, Daniella Coker, Gabriel Cuellar Partida, Devika Dhamija, Sayantan Das, Sarah L. Elson, Nicholas Eriksson, Teresa Filshtein, Alison Fitch, Kipper Fletez-Brant, Pierre Fontanillas, Will Freyman, Julie M. Granka, Karl Heilbron, Alejandro Hernandez, Barry Hicks, David A. Hinds, Ethan M. Jewett, Yunxuan Jiang, Katelyn Kukar, Alan Kwong, Keng-Han Lin, Bianca A. Llamas, Maya Lowe, Jey C. McCreight, Matthew H. McIntyre, Steven J. Micheletti, Meghan E. Moreno, Priyanka Nandakumar, Dominique T. Nguyen, Elizabeth S. Noblin, Jared O'Connell, Aaron A. Petrakovitz, G. David Poznik, Alexandra Reynoso, Morgan Schumacher, Anjali J. Shastri, Janie F. Shelton, Jingchunzi Shi, Suyash Shringarpure, Qiaojuan Jane Su, Susana A. Tat, Christophe Toukam Tchakouté, Vinh Tran, Joyce Y. Tung, Xin Wang, Wei Wang, Catherine H. Weldon, Peter Wilton, Corinna D. Wong.

**Members of the International Glaucoma Genetics Consortium**

Puya Gharahkhani, Eric Jorgenson, Anthony P. Khawaja, Sarah Pendergrass, Xikun Han, Jue Sheng Ong, Alex W. Hewitt, Ayellet V. Segrè, John M. Rouhana, Andrew R. Hamel, Robert P. Igo Jr, Helene Choquet, Ayub Qassim, Navya S. Josyula, Jessica N. Cooke Bailey, Pieter W. M. Bonnemaijer, Owen M. Siggs, Terri L. Young, Veronique Vitart, Alberta A. H. J. Thiadens, Juha Karjalainen, Steffen Uebe, Ronald B. Melles, K. Saidas Nair, Robert Luben, Mark Simcoe, Nishani Amersinghe, Angela J. Cree, Rene Hohn, Alicia Poplawski, Li Jia Chen, Shi-Song Rong, Tin Aung, Eranga Nishanthie Vithana, Gen Tamiya, Yukihiro Shiga, Masayuki Yamamoto, Toru Nakazawa, Hannah Currant, Ewan Birney, Michelle K. Lupton, Nicholas G. Martin, Adeyinka Ashaye, Olusola Olawoye, Susan E. Williams, Stephen Akafo, Michele Ramsay, Kazuki Hashimoto, Yoichiro Kamatani, Masato Akiyama, Yukihide Momozawa, Paul J. Foster, Peng T. Khaw, James E. Morgan, Nicholas G. Strouthidis, Peter Kraft, Jae H. Kang, Chi Pui Pang, Francesca Pasutto, Paul Mitchell, Andrew J. Lotery, Aarno Palotie, Jonathan L. Haines, Louis R. Pasquale, Caroline C. W. Klaver, Michael Hauser, Chiea Chuen Khor, David A. Mackey, Michiaki Kubo, Ching-Yu Cheng, Jamie E. Craig, Stuart MacGregor, Janey L. Wiggs, Elisabeth M. van Leeuwen, Adriana I. Iglesias, René Höhn, Aslihan Gerhold-Ay, Stefan Nickels, James F. Wilson, Caroline Hayward, Thibaud S. Boutin, Ozren Polašek, Najaf Amin, Pirro G. Hysi, Christopher J. Hammond, Cornelia M. van Duijn, Henriët Springelkamp, Aniket Mishra, Robert Wojciechowski, Abhishek Nag, Ya Xing Wang, Jie Jin Wang, Gabriel Cuellar-Partida, Jane Gibson, Wishal D. Ramdas, Tanja Zeller, Robert N. Luben, Ekaterina Yonova-Doing, Ananth C. Viswanathan, Seyhan Yazar, Jia Yu Koh, Emmanuelle Souzeau, Christian Müller,

Cristina Venturini, Lisa S. Kearns, Jae Hee Kang, Yih Chung Tham, Tiger Zhou, Paul Sanfilippo, Jiemin Liao, Herma van der Linde, Wanting Zhao, Leonieke M. E. van Koolwijk, Li Zheng, Fernando Rivadeneira, Mani Baskaran, Sven J. van der Lee, Shamira Perera, Paulus T. V. M. de Jong, Ben A. Oostra, André G. Uitterlinden, Qiao Fan, Albert Hofman, E.-Shyong Tai, Johannes R. Vingerling, Xueling Sim, Roger C. W. Wolfs, Yik Ying Teo, Hans G. Lemij, Rob Willemsen, Karl J. Lackner, Nomdo M. Jansonius, Grant Montgomery, Philipp S. Wild, Kathryn P. Burdon, Tien Yin Wong, Jost B. Jonas, Norbert Pfeiffer, Alexander Schuster, Caroline Brandl, Fridbert Jonansson, Gudmar Thorliefsson, Joelle Vergroesen, John Fingert, Kari Stefansson, Unnur Thorsteinsdottir, VA de Vries, Sjoerd Driessen
